# Removal of Dust Microelectric Signal Based on Empirical Mode Decomposition and Multifractal Detrended Fluctuation Analysis

**DOI:** 10.1155/2021/5468514

**Published:** 2021-08-06

**Authors:** Jiming Li, Yongji Sun, Xuezhen Cheng

**Affiliations:** College of Electrical Engineering and Automation, Shandong University of Science and Technology, 579 Qianwangang Road, Huangdao District, Qingdao 266590, Shandong, China

## Abstract

Microcharge induction has recently been applied as a dust detection method. However, in complex environments, the detection device can be seriously polluted by noise. To improve the quality of the measured signal, the characteristics of both the signal and the noise should be analyzed so as to determine an effective noise removal method. Traditional removal methods mostly deal with specific noise signals, and it is difficult to consider the correlation of measured signals between adjacent time periods. To overcome this shortcoming, we describe a method in which wavelet decomposition is applied to the measured signal to obtain sub-band components in different frequency ranges. A time-lapse Pearson method is then used to analyze the correlation of the sub-band components and the noise signal. This allows the sub-band component of the measurement signal that has the strongest correlation with the noise to be determined. Based on multifractal detrended fluctuation analysis combined with empirical mode decomposition, the similarity between the signal sub-band components and the noise sub-band components is analyzed and three indices are employed to determine the multifractal characteristics of the sub-band components. The consistency between noise components and signal components is obtained and the main signal components are verified. Finally, the sub-band components are used to reconstruct the signal, giving the noise-free measured (microcharge induction) signal. The filtered signal presents smoother, multifractal features.

## 1. Introduction

One of the notable properties of dust is its electrification during movement. There has been considerable research on the chargeability of dust and how to measure the electrostatic signal [1–6], and it has been widely used in the detection of gas flow velocity in the field of pneumatic conveying. Yan et al. [7–9] conducted extensive research on electrostatic induction for measuring gas-solid two-phase flows and proposed various types of measurement electrodes and measurement methods. In recent years, soft measurement and machine learning [10] have been applied to measure the parameters of gas-solid two-phase flows by the induced charge method. Zheng et al. [11] studied the electrification of sand dust and the spatial variation of the particle concentration along the flow direction by assuming a certain charge-to-mass ratio. Xu et al. [12, 13] proposed a new type of electrostatic sensor system for measuring the pulverized coal speed and relative mass flow, which determines the speed of pulverized coal from the autocorrelation of the output signal of the electrostatic sensor array. The relative concentration and mass flow rate are then obtained from the root mean square value output by two ring electrodes. The present authors [6] have conducted research on the theory of mine dust belt motors, charging modes, charging measurement models, and measuring devices and have identified nonlinear characteristics in the dust electrostatic signal that reflect the multiscale information contained in the signal. Because the measurement environment of the detection device is complex and changeable, the measured dust electrostatic signal always contains noise. The noise that exists when the detection device is working is also called measurement noise (in the remainder of this paper, the electrostatic signal of dust is referred to as the measurement signal). This noise seriously affects the measurement accuracy and must be removed.

The processing of electrostatic signals obtained by electrostatic sensors is mostly achieved by adding anti-aliasing or low-pass filters to the hardware circuit. There are relatively few signal processing methods for electrostatic signals. Wang et al. [14] compared the frequency spectra of the measured signals from external-surface electrostatic sensors and embedded electrostatic sensors and used a harmonic wavelet transform to decompose the measured signal. The frequency spectrum of inner flush-mounted electrostatic sensors was found to contain a critical frequency for decoupling the induced charge signal from the transferred charge signal, thus improving the cross-correlation speed of the signal. Kamel and Yan [15] proposed a hybrid removal method that combines the cut-off frequency method for removing the noise component outside the signal bandwidth with a median filter for smoothing the signal, thus minimizing the effect of noise in the signal. Wang [16] proposed a radial vibration measurement method based on electrostatic sensing and Hilbert–Huang transform (HHT) signal processing, which provides a simple and economical method for rotating-shaft radial vibration measurements. Zhang [17] used the HHT to study the characteristics of different electrodes' electrostatic signals and extracted the features that characterize the changes in solid concentration. Wang et al. [18] proposed an adaptive decomposition method based on the discrete wavelet transform (DWT), which effectively reduces the average relative standard deviation of the solid velocity. The abovementioned studies can be combined with modern signal processing methods to achieve electrostatic signal processing, but the basic characteristics and frequency range of noise signals are preset, and so adaptive noise recognition and processing cannot be performed. To remove noise, it is necessary to separate the signal from the noise, or to identify the obvious differences in their respective characteristics. The multilayer wavelet decomposition transform is an effective signal-noise separation method in both the time domain and frequency domain. Therefore, many studies [19–23] have used the multilayer wavelet decomposition transform to remove noise. Wavelet transforms can decompose the noise and the signal into different frequency bands, but they do not have the ability to adaptively identify which sub-band is the noise.

The fractal theory proposed by Mandelbrot [24] provides the possibility of analyzing the similarity and correlation between the measured signal and the noise signal. In particular, multifractals [25, 26] enable a detailed description of the local characteristics of the signal. Combining the theory of multifractals and detrended fluctuation analysis, Kantelhardt et al. [27] developed multifractal detrended fluctuation analysis (MFDFA). MFDFA has since been widely applied to stock market analysis [28], temperature time series [29], seismic wave signals [30], vibration tomographic diagnosis [31], image processing [32], and voice signal analysis [33]. MFDFA can effectively describe the nonlinear measurement signal, especially the multifractal characteristics of the time series, but the analysis of the time series signal requires a detrending process, which causes pseudo-fluctuation errors to appear. There are two main reasons for this: one is that the sequence is over- or underfitted due to the uncertainty of the order of the fitted polynomial function; the other is that MFDFA uses a uniform sequence for data segmentation, resulting in sequence segmentation points that are not continuous. To solve these problems, Li et al. [34] proposed an MFDFA algorithm based on empirical mode decomposition (EMD) and template movement. This algorithm was used to analyze the multifractal characteristics of harmonic signals, resulting in a new method for determining the characteristics of harmonic signals. This method can be used to analyze the similarity between the noise and signal sub-bands given by wavelet analysis, allowing the signal component and noise component to be identified. The noise component can then be filtered out and the signal component reconstructed, thus realizing signal denoising.

In order to separate noise signal and measurement signal, this article proposes an asymmetric two-electrode measuring device, most signal components of one electrode are electrostatic signals, and the other contains most noise signals. A wavelet transform is used to decompose the electrostatic signals and noise signals, and a time-lapse Pearson correlation analysis method is developed to analyze the correlation between the signal wavelet and the noise sub-bands to confirm which signal components have the most correlation with noise. The result of time-lapse Pearson determines that *d*_3_ is a noise component, but it is impossible to determine which other components are noise and which are signals. So the EMD-MFDFA algorithm is then used to analyze the similarity between the signal sub-band and the noise sub-band and determine the wavelet sub-bands in which the electrostatic signal is located, and the results show that *a*_4_, *a*_5_, *d*_4_, and *d*_5_ are the signal components. Finally, the noise is filtered and the denoised electrostatic signal is reconstructed with *a*_4_, *a*_5_, *d*_4_, and *d*_5_.

## 2. Analysis Method Combining Multifractals and Empirical Mode Decomposition

### 2.1. Generalized Multifractal Detrending Algorithm [27]

(1) Determine the “contour” of *x*_*k*_ as
  (1) $$\begin{array}{c} Y\left(i\right)=\sum_{k=1}^{i}\left(x_{k}-\bar{x}\right), \end{array}$$
where $\bar{x}=\left(1/N\right)\sum_{k=1}^{N}x_{k}$.
(2) Divide the contour *Y*(*i*) into *N*_*s*_=int(*N*/*s*) equal-length nonoverlapping segments with a length of *s*. There may be a segment with a length of less than *s* at the end of the contour. To eliminate the influence of this part, the same process is repeated from the opposite end to obtain 2*N*_*s*_ data segments.
(3) Calculate the local trend of each of the 2*N*_*s*_ segments through the least-squares fitting of the sequence, and then determine the variance as
  (2) $$\begin{array}{c} F^{2}\left(s,v\right)=\frac{1}{s}\sum_{i=1}^{s}{\left\{Y\left[\left(v-1\right)s+i\right]-y_{v}\left(i\right)\right\}}^{2}, \end{array}$$
where each segment is denoted by *v*, *v*=1,…, *N*_*s*_, and
  (3) $$\begin{array}{c} F^{2}\left(s,v\right)=\frac{1}{s}\sum_{i=1}^{s}{\left\{Y\left[N-\left(v-\left|N_{s}\right|\right)s+i\right]-y_{v}\left(i\right)\right\}}^{2}, \end{array}$$
where *v*=*N*_*s*_+1,…, 2*N*_*s*_. *y*_*v*_(*i*) is the fitting polynomial of the data segment *v*. Linear, quadratic, cubic, or higher-order polynomials can be used in the fitting process (generally called DFA1, DFA2, DFA3, and so on). As the time series is detrended by subtracting the polynomial fitting from the contour, the ability of the different-order DFAs to eliminate the trend of the series is different. In m-order MFDFA, the m-order trend (or, equivalently, the (*m* − 1)-order trend in the original sequence) in the contour is eliminated.
(4) Take the average of all line segments to obtain the q-order wave function
  (4) $$\begin{array}{c} F_{q}\left(s\right)={\left\{\frac{1}{2N_{s}}\sum_{v=1}^{2N_{s}}{\left[F^{2}\left(s,v\right)\right]}^{q/2}\right\}}^{1/q}, \end{array}$$
where *F*_*q*_(*s*) is a q-order function of *s*. As *s* increases, *F*_*q*_(*s*) has a power relationship with *s*:
  (5) $$\begin{array}{c} s^{h\left(q\right)}\propto F_{q}\left(s\right), \end{array}$$
where *h*(*q*) is the Hurst index, which is related to the multifractal quality index *τ*(*q*) according to
  (6) $$\begin{array}{c} \tau \left(q\right)=qh\left(q\right)-1. \end{array}$$
(5) According to the Legendre transformation, the relationship between the singular index *α* and the multifractal spectrum *f*(*α*) and *h*(*q*) is
  (7) $$\begin{array}{c} \alpha =h\left(q\right)+qh^{\prime }\left(q\right)=h\left(q\right)+q\frac{\text{d}h\left(q\right)}{\text{d}q},\\ f\left(\alpha \right)=q\left[\alpha -h\left(q\right)\right]+1. \end{array}$$

### 2.2. Empirical Mode Decomposition Algorithm [35, 36]

EMD enables the smoothing of nonstationary signals, with fluctuations and changing trends on different characteristic time scales identified by decomposing the signal. The decomposition result is composed of multiple intrinsic mode functions (IMFs) *c*_1_(*t*), *c*_2_(*t*),…, *c*_*n*_(*t*) and residuals *r*_*n*_(*t*), where *r*_*n*_(*t*) is a monotonic function that is the average trend of the signal.

The EMD process can be described as follows:

(8) $$\begin{array}{c} x\left(t\right)=\sum_{i=0}^{n}c_{i}\left(t\right)+r_{n}\left(t\right). \end{array}$$

### 2.3. Improved Method Based on EMD-MFDFA

Analyzing the third step of the MFDFA algorithm, it can be seen that the order of the fitting polynomial *y*_*v*_(*i*) cannot be determined at the initial stage of signal analysis. *y*_*v*_(*i*) is obtained by curve fitting. If the order is very high, it will take a long time to determine the order, and the determined polynomial may not be optimal. Improper fitting of the volatility trend will introduce erroneous volatility and affect the analysis results. In the EMD result, *r*_*n*_(*t*) reflects the average trend characteristics of the signal. Using *r*_*n*_(*t*) instead of *y*_*v*_(*i*) in the third step of the MFDFA constitutes a new EMD-MFDFA algorithm.

Third step: the local variance of each of the 2*N*_*s*_ segments is

(9) $$\begin{array}{c} F^{2}\left(s,v\right)=\frac{1}{s}\sum_{i=1}^{s}{\left\{Y\left[\left(v-1\right)s+i\right]-r_{v}\left(i\right)\right\}}^{2}, \end{array}$$

where each segment is denoted by *v*, *v*=1,…, *N*_*s*_, and

(10) $$\begin{array}{c} F^{2}\left(s,v\right)=\frac{1}{s}\sum_{i=1}^{s}{\left\{Y\left[N-\left(v-\left|N_{s}\right|\right)s+i\right]-r_{v}\left(i\right)\right\}}^{2}, \end{array}$$

where *v*=*N*_*s*_+1,…, 2*N*_*s*_.

The first, second, and fourth steps of the EMD-MFDFA algorithm are the same as in the MFDFA algorithm. The following research compares the effects of the MFDFA algorithm and the EMD-MFDFA algorithm in multiple analysis applications.

### 2.4. Time-Lapse Pearson Analysis Method

The Pearson correlation coefficient describes the correlation between two variables at the same time and does not consider the time lag of the change between variables. This paper proposes a Pearson correlation coefficient method with time-lapse correlation analysis, which enables the time-lapse correlation between two variables to be analyzed.

The Pearson correlation coefficient between samples *X* and *Y* of a total of *N* samples is given by

(11) $$\begin{array}{c} r=\frac{\sum_{i=1}^{N}\left(x_{i}-\bar{x}\right)\left(y_{i}-\bar{y}\right)}{\sqrt{\sum_{i=1}^{N}{\left(x_{i}-\bar{x}\right)}^{2}\sum_{i=1}^{N}{\left(y_{i}-\bar{y}\right)}^{2}}}, \end{array}$$

where $\bar{x}=\left(1/N\right)\sum_{i=1}^{N}x_{i}$, $\bar{y}=\left(1/N\right)\sum_{i=1}^{N}y_{i}$, *X*=[*x*_1_,…, *x*_*N*−1_, *x*_*N*_], and *Y*=[*y*_1_,…, *y*_*i*−1_, *y*_*i*_].

The range of Pearson's correlation coefficient *r* is [–1, 1]; |*r*| represents the degree of correlation between two variables, with |*r*| closer to 1 indicating that the correlation between the two variables is higher, and the relationship between them is closer.

The time-lapse Pearson correlation between *X* and *Y*_*j*_ is defined as

(12) $$\begin{array}{c} r_{j}=\frac{\sum_{i=1}^{N}\left(x_{i}-\bar{x}\right)\left(y_{i+j}-\bar{y_{j}}\right)}{\sqrt{\sum_{i=1}^{N}{\left(x_{i}-\bar{x}\right)}^{2}\sum_{i=1}^{N}{\left(y_{i+j}-\bar{y_{j}}\right)}^{2}}}, \end{array}$$

where $\bar{x}=\left(1/N\right)\sum_{i=1}^{N}x_{i}$, $\bar{y_{j}}=\left(1/N\right)\sum_{i=1}^{N}y_{i+j}$, *X*=[*x*_1_,…, *x*_*N*−1_, *x*_*N*_], and *Y*_*j*_=[*y*_*j*_, *y*_*j*+1_,…, *y*_*i*+*j*−1_, *y*_*i*+*j*_].

The time-lapse Pearson correlation matrix is

(13) $$\begin{array}{c} R=\left[r_{-j},r_{-j+1},\ldots ,r_{-1},r_{0},r_{1},\ldots ,r_{j-1},r_{j}\right]. \end{array}$$

## 3. Signal Acquisition

The study in [15] describes a number of treatments for weak signal-detection circuit design and noise shielding and finds that the measurement signal is still inevitably subjected to interference by surrounding noise signals. Obviously, in addition to the necessary hardware protection to reduce noise interference, noise removal is required. First, it is necessary to understand the morphological characteristics of the noise signal obtained by the detection circuit, and to distinguish the noise components in the measurement signal. The noise in the measurement signal can then be effectively removed.

### 3.1. Asymmetric Two-Electrode Measuring Device

**Figure 1:**
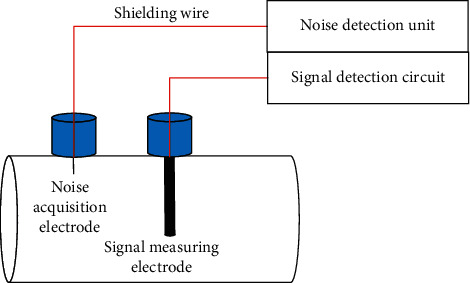
Schematic figure of the structure of the measuring device.

To remove noise, an asymmetric two-electrode measurement unit is developed. One electrode is the noise collection terminal, which mainly collects noise signals in the detection environment, and the other electrode is the signal collection terminal, which mainly collects noise-containing microelectric signals. According to the positive correlation between the induction signal and the size of the electrode [6], it can be assumed that the induction signal obtained by the noise-collecting electrode is much smaller than the induction signal measured by the measuring electrode and that noise is the main component. The noise-collecting electrode is much smaller than the measuring electrode, having a length of 20–30 mm and a diameter of 1–2 mm. The measuring electrode has a length of 400 mm and a diameter of 10 mm. The structure of the measuring device is shown in Figure 1.

### 3.2. Acquisition of Measurement Signal

**Figure 2:**
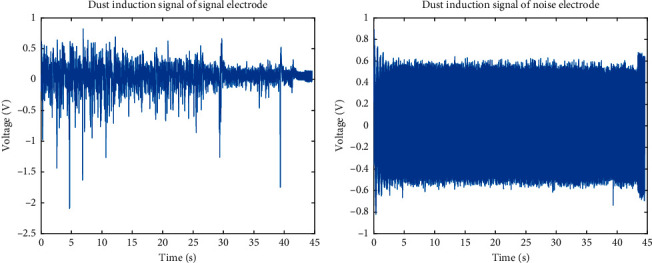
Acquisition of measurement signal. (a) Signal of signal-measuring electrode. (b) Signal of noise-measuring electrode.

Figures 2(a) and 2(b) show the dust microelectric signal collected by the measuring device. It can be seen that the signal induced on the measuring electrode by the movement of the dust is much larger than the signal collected by the noise-collecting electrode. In the measurement signal recorded by the noise electrode, the effective induction signal is almost completely submerged in the noise.

## 4. Characteristic Analysis of Measurement Signal and Measurement Noise Signal

**Figure 3:**
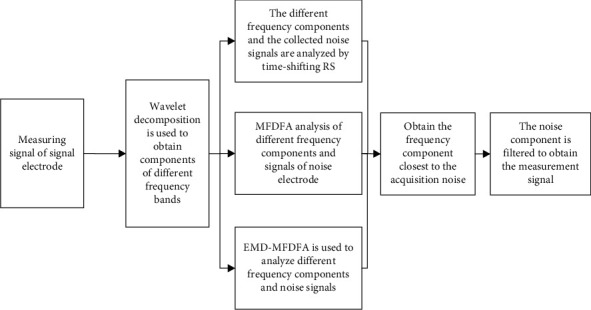
Architecture diagram of feature analysis.

Figure 2 illustrates that the measurement signal exhibits nonlinear and random characteristics, as does the noise in the measurement environment. To effectively filter the noise from the measurement signal, the measurement noise needs to be identified. First, wavelet decomposition is applied to the signal recorded by the measuring electrode to obtain the frequency components in different frequency ranges. Time-lapse Pearson analysis is then performed on the different frequency components and noise signals to identify those components that have the strongest correlation with the noise. Using the MFDFA algorithm and the improved EMD-MFDFA algorithm, the similarity between the different frequency bands of the signal and the measurement noise is then analyzed, and the frequency components closest to the collected noise are removed. This analysis makes it possible to determine the characteristics of the noise that affect the measurement signal, and to obtain the noise signal as the basis for noise filtering. The architecture is shown in Figure 3.

### 4.1. Wavelet Analysis of Measurement Signals

#### 4.1.1. db24 Wavelet Decomposition

**Figure 4:**
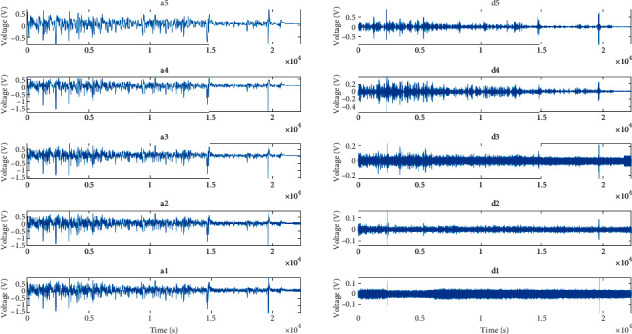
Wavelet sub-band waveforms of measuring electrode's signal.

The application of the db24 wavelet with five-layer decomposition to the measured signal shown in Figure 2(a) gives the signal waveforms of each sub-band, as shown in Figure 4.

The frequency range of each sub-band is listed in Table 1. Analysis shows that each decomposed sub-band contains corresponding frequency components, and the signal amplitudes of the high-frequency signal parts *d*_1_, *d*_2_, and *d*_3_ are small, but not negligible.

### 4.2. Time-Lapse Pearson Correlation Analysis of the Measured Signal Sub-Band and the Noise Signal

#### 4.2.1. Analysis of Time-Lapse Pearson Correlation between the Measured Signal Sub-Band and the Noise Signal

**Figure 5:**
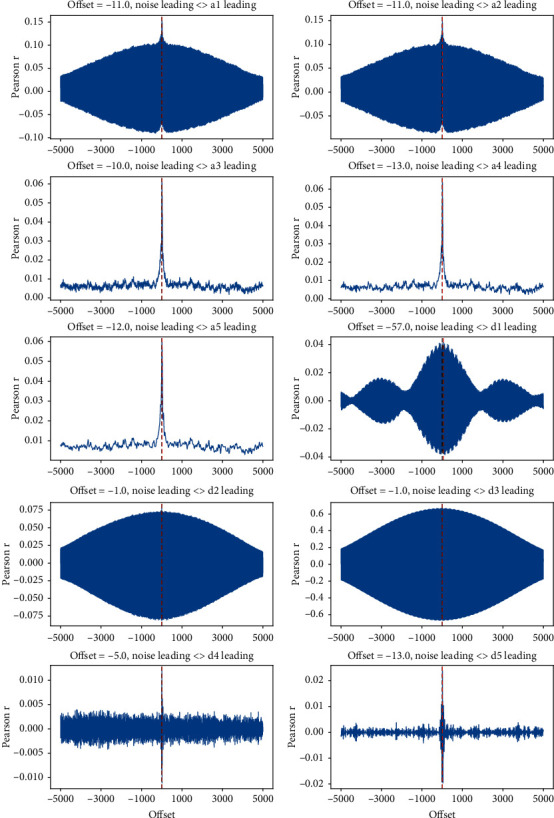
Correlation diagram of each sub-band signal and noise signal.

Figure 5 shows the result of direct analysis of the correlation between the measured signal sub-band and the noise signal for about 10s (5000 data points). The focus is whether the change in noise occurs first, or whether the detection signal leads.

From Figure 5, the following points can be identified: (1) the time-lapse correlation coefficient between component *d*_3_ and the noise is greater than 0.6, indicating a strong correlation between the two; (2) the two signals appear to change simultaneously (only 1-point difference, 0.002 s), and the frequency range of *d*_3_ is 29.75–59.5 Hz.

#### 4.2.2. Wavelet Sub-Band Decomposition of Noise Signal

By analyzing the time-lapse Pearson correlation between the wavelet sub-band of the measured signal and the measured noise, it is clear that there is a strong correlation between *d*_3_ and the noise, although the two are not completely correlated. The noise contains a strong 50 Hz component, which masks the correlation between other components and the noise. First, the same method as for measurement signal analysis is used to decompose the noise signal into its sub-bands; the wavelet sub-band ranges of the noise are the same as in Table 1.

The time-lapse Pearson correlation between the measured signal wavelet sub-bands and the noise wavelet sub-bands is presented in Table 2. The following correlations can be identified:

1. nos_*a*_4_–*a*_4_ and nos_*a*_5_–*a*_5_ are highly correlated and exhibit the characteristics of simultaneous changes
2. nos_*d*_3_–*d*_3_ indicates that, given the strong correlation between *d*_3_ and the overall noise signal, component *d*_3_ can be considered as a noise signal
3. nos_*d*_1_–*d*_1_, nos_*a*_3_–*a*_3_, and nos_*d*_5_–*d*_5_ exhibit a certain correlation, but it is impossible to directly determine which is the noise component and which is the signal

### 4.3. Multifractal Characteristic Analysis of Signal and Noise

The MFDFA algorithm is used to study the multifractal characteristics of the measured signal and the measured noise, which is judged as follows.

Relationship between *q* and *h*(*q*): for *q*=2, the Hurst exponent *h*(*q*) has the following properties [37]: when *h*(*q*) = 0.5, the signal sequence has no correlation; when *h*(*q*)>0.5, the signal sequence has a positive long-range correlation, indicating that the time series is positively correlated; when *h*(*q*)<0.5, the time series is negatively correlated. If *q* is not related to *h*(*q*), the signal is monofractal; if *q* is related to *h*(*q*), the signal is multifractal.

Relationship between *q* and *τ*(*q*): if *τ*(*q*) is a straight line, then the signal is simplex; if *q* and *τ*(*q*) are nonlinear, then the signal is multifractal.

Relationship between *α* and *f*(*α*): if *f*(*α*) is a constant, the signal is a single-shaped signal; if the curve between *α* and *f*(*α*) has a single-peak bell shape, the signal is multifractal.

**Figure 6:**
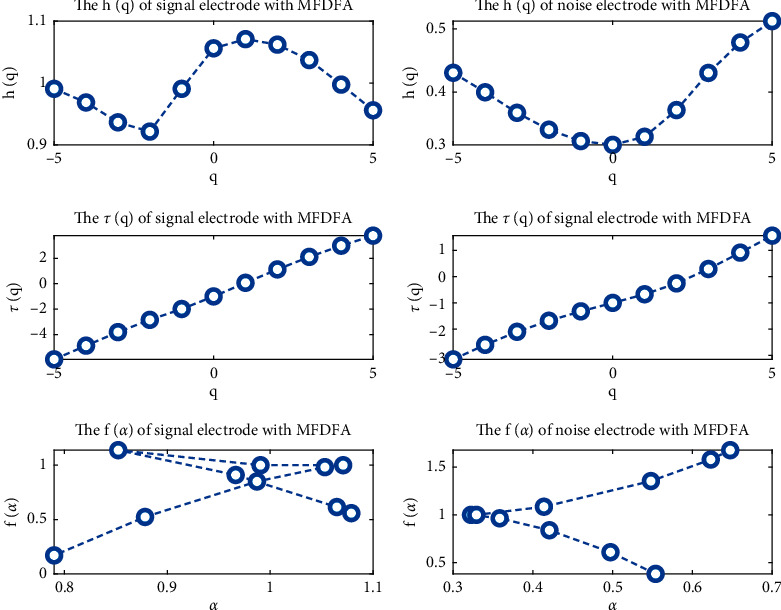
Multifractal characteristic figures of measured signal/noise with MFDFA.

MFDFA analysis was applied to the measured signal and the measured noise signal, and the results are shown in Figure 6. Both *q* − *τ*(*q*) and *α* − *f*(*α*) indicate that the measured signal and measured noise do not have multifractal characteristics.

The multifractal features of the signal are submerged by noise pollution. Next, by analyzing the multifractal features of the measured signal and measured noise on each wavelet sub-band, it is possible to determine which sub-bands have noise signals as their main components and which are mainly measured signals.

#### 4.3.1. Analysis of the Detrending Ability of MFDFA and EMD-MFDFA Algorithms

**Figure 7:**
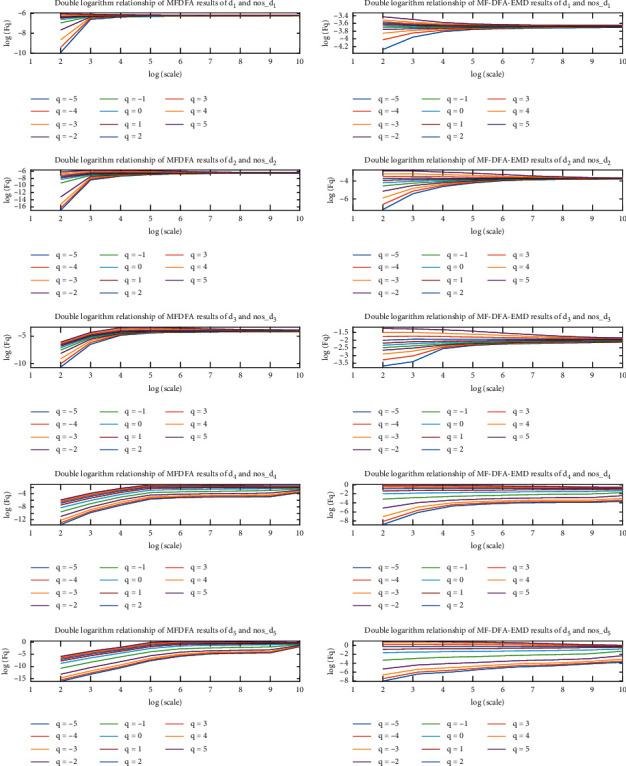
MFDFA and EMD-MFDFA detrending capability curves.

The log(*Fq*) − log(*s*) curve of the wavelet sub-band *d* component of the signal was obtained using MFDFA and EMD-MFDFA. The detrending polynomial of the MFDFA algorithm is linear, and the EMD-MFDFA detrending term is the residual *r*_*n*_(*t*) of EMD. The results are shown in Figure 7. From the value of *Fq* in Figure 7, we find that the absolute value of the result obtained by EMD-MFDFA and its numerical range are smaller than those obtained by MFDFA. This shows that EMD-MFDFA has stronger detrending ability than MFDFA.

#### 4.3.2. Analysis of MFDFA and EMD-MFDFA Characteristics: Wavelet Sub-Bands of Measurement Signals and Wavelet Sub-Bands of Noise Signals

**Figure 8:**
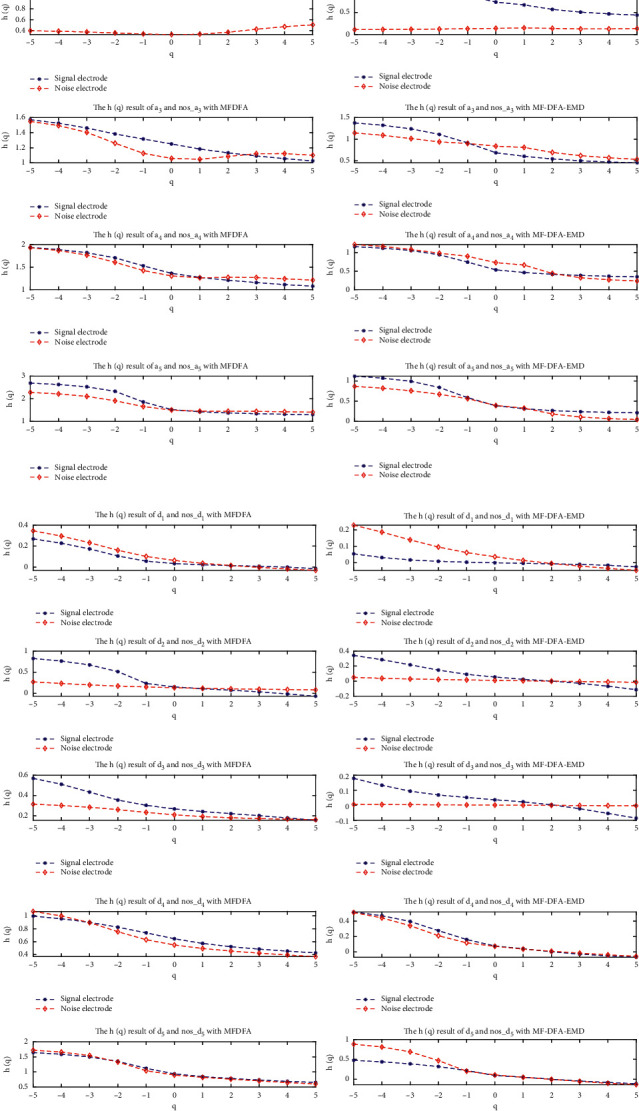
MFDFA and EMD-MFDFA *q*-*h*(*q*) curves of each sub-band of the measured signal and measured noise: (a) MFDFA; (b) EMD-MFDFA.

*(1) q-h(q) Characteristic Analysis*. The wavelet sub-bands of the measured signal and the measured noise obtained from MFDFA and EMD-MFDFA are now analyzed. The results are shown in Figure 8. For *q*=2, the Hurst exponent *h*(*q*) of each wavelet sub-band component of the measured signal and noise and its long-range correlation are presented in Tables 3 and 4. Analysis of the MFDFA and EMD-MFDFA algorithms shows that EMD-MFDFA can effectively eliminate the influence of the trend item, so that the trend characteristic of the affected component is eliminated. This highlights the multifractal characteristics of the component. By comparing the *q* − *τ*(*q*) values of signal components *a*_1_ and *a*_3_ in Figure 8, it can be seen that EMD-MFDFA highlights the multifractal characteristics of the measured signal components. EMD-MFDFA gives smaller *k* values for acoustic components *a*_2_, *d*_2_, and *d*_3_ than MFDFA, which leads to the fact that the measured signal does not have multifractal characteristics.

*(2) *q* − *τ*(*q*) Characteristic Analysis*. Next, MFDFA and EMD-MFDFA were used to obtain the wavelet sub-bands of the measured signal and the measured noise, and the linearity *R* between *q* and *τ*(*q*) was calculated. The results are presented in Tables 5 and 6. Comparing the relationship between *R* and *q* − *τ*(*q*), it is apparent that values of *τ*(*q*) greater than 0.96 indicate multifractal characteristics. EMD-MFDFA effectively reduces the trend characteristics of components and highlights their fractal characteristics.

**Figure 9:**
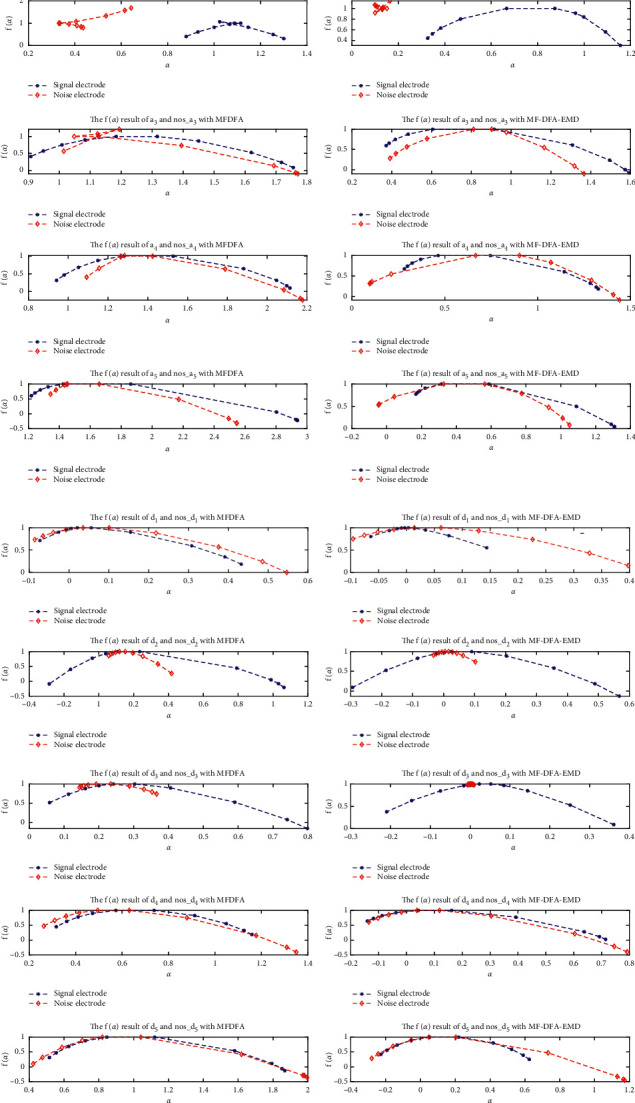
MFDFA and EMD-MFDFA *α* − *f*(*α*) curves of each sub-band of the measured signal and measured noise: (a) MFDFA; (b) EMD-MFDFA.

*(3) *α* − *f*(*α*) Characteristic Analysis*. MFDFA and EMD-MFDFA were then used to obtain the wavelet sub-band signals of the measured signal and the measured noise, and the *α* − *f*(*α*) waveforms were calculated. The waveforms are shown in Figure 9, and the fractal feature judgments are presented in Tables 7 and 8.

**Figure 10:**
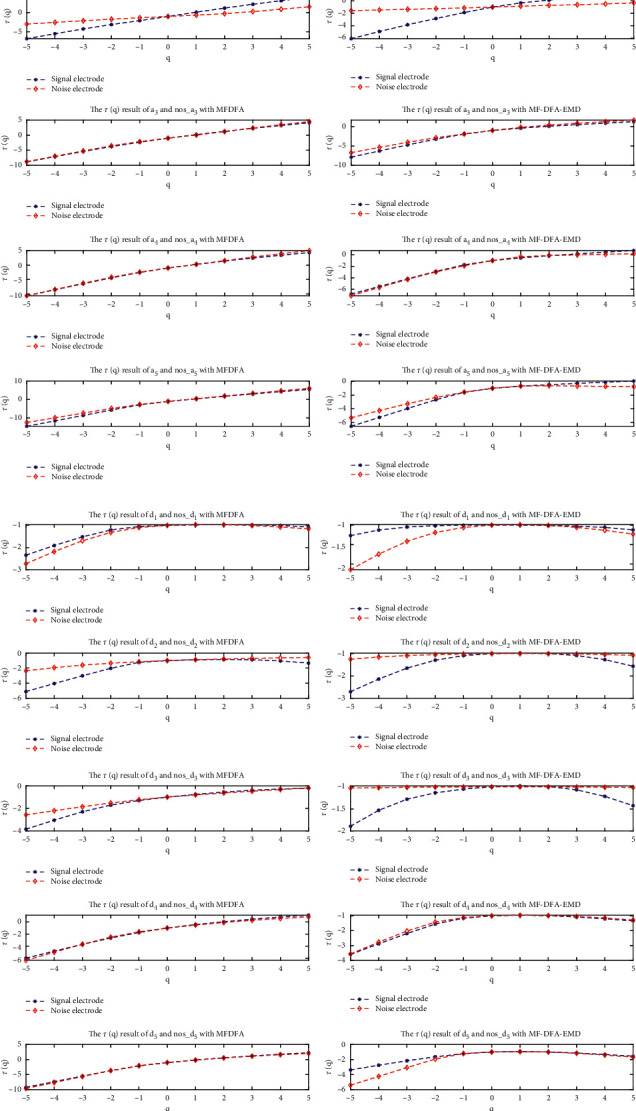
MFDFA and EMD-MFDFA *q* − *τ*(*q*) curves of each sub-band of the measured signal and measured noise: (a) MFDFA; (b) EMD-MFDFA.

*(4). Determination of Signal Wavelet Sub-Band*. The correlation, average error, and standard deviation are now used to evaluate the differences among the EMD-MFDFA analysis results of each sub-band using *h*(*q*), *τ*(*q*), and *f*(*α*). The results are presented in Tables 9–11. Combining the results of Figures 8–10, it can be seen that the multifractal features of *a*_4_ − nos_*a*_4_, *a*_5_ − nos_*a*_5_, *d*_4_ − nos_*d*_4_, and *d*_5_ − nos_*d*_5_ have very high consistency. These components are mainly concentrated at low frequencies, so *a*_4_, *a*_5_, *d*_4_, and *d*_5_ can be considered as real signal components of the measured signal.

## 5. Signal Noise Filtering

Combining the time-lapse Pearson analysis results and the analysis results using the EMD-MFDFA algorithm, it can be seen that component *d*_3_ is highly correlated in the wavelet decomposition of the measured signal and the measured noise. This can be considered as the main noise component, and the results of multifractal analysis show that *a*_4_, *a*_5_, *d*_4_, and *d*_5_ are the main real signal components. The wavelet decomposition components *d*_1_ and *d*_2_ of the two channel acquisition signals exhibit disorder. This indicates that the signal is polluted by disordered noise.

**Figure 11:**
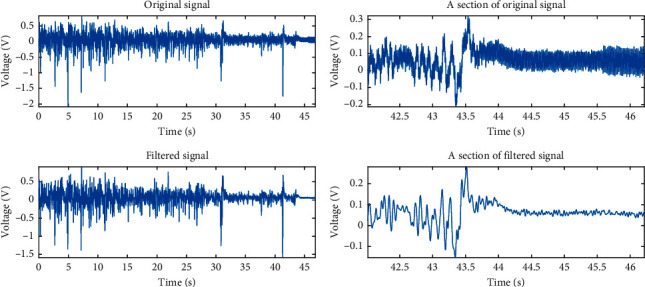
Signal diagram after filtering of the signal-detection electrode.

**Figure 12:**
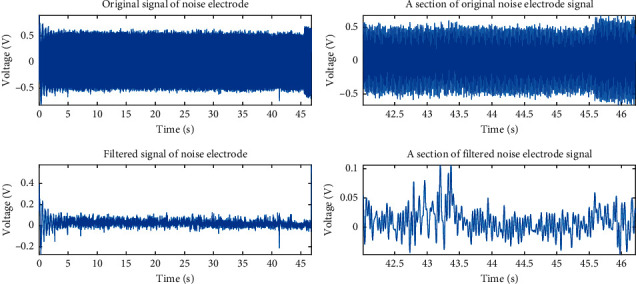
Signal diagram after filtering of the noise-detection electrode.

The *a*_4_, *a*_5_, *d*_4_, and *d*_5_ components can be used to reconstruct a new signal, regarded as an electrostatic signal after noise filtering. The filtered waveform is shown in Figure 11. It can be seen that the filtered signal is smoother than the original signal, as the interference of power frequency noise and disordered noise has been eliminated, and provides a basis for further signal analysis. Even if the measured noise is completely submerged by noise, the real charge-induced signal can be obtained by filtering, as shown in Figure 12.

**Figure 13:**
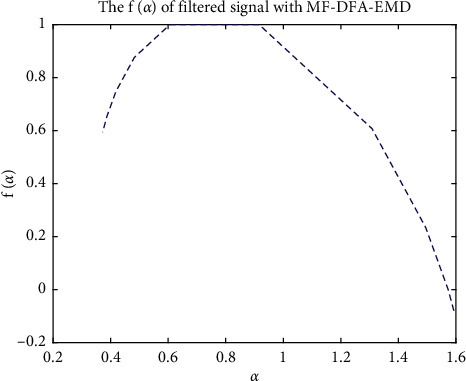
*α* − *f*(*α*) curve of the signal after removing the noise.

The filtered signal was analyzed using EMD-MFDFA, and the *α* − *f*(*α*) curve was found to exhibit multifractal characteristics (see Figure 13). This indicates that the filtered signal has multifractal characteristics.

## 6. Conclusion

1. First, the measurement model was improved, and a signal acquisition method using an asymmetric two-electrode structure was proposed. In this method, large-sized electrodes are used to obtain the induced signals and small-sized electrodes are used to record the noise.
2. A method of combining EMD-MFDFA with time-lapse Pearson correlation analysis has been proposed. Based on the db24 wavelet decomposition method, the measured signal and measured noise were decomposed. The results of the time-lapse Pearson correlation analysis showed that the *d*_3_ component of the induced signal wavelet decomposition had a strong overall correlation with the noise and exhibited the characteristics of simultaneous changes, without any delay or lag.
3. Further, based on the wavelet decomposition of the noise signal, time-lapse Pearson correlation analysis found that nos_*a*_4_–*a*_4_, nos_*a*_5_–*a*_5_, nos_*d*_3_–*d*_3_, nos_*d*_1_–*d*_1_, nos_*a*_3_–*a*_3_, and nos_*d*_5_–*d*_5_ exhibited some correlation. However, it was impossible to directly determine which components were noise and which were part of the signal using this method.
4. Multifractal feature analysis of the measured signal found no obvious multifractal features. MFDFA and EMD-MFDFA analysis of each component after wavelet decomposition did, however, find obvious multifractal features in several components.
5. Comparing the multifractal results of the measured signal wavelet sub-band and the measured noise wavelet sub-band, there is obvious consistency between nos_*a*_4_–*a*_4_, nos_*a*_5_–*a*_5_, nos_*d*_4_–*d*_4_, and nos_*d*_5_–*d*_5_. It was verified that the components of the measured signal were mainly concentrated at low frequencies, and *a*_4_, *a*_5_, *d*_4_, and *d*_5_ were considered to be the main components of the real signal.
6. The signal was reconstructed using components *a*_4_, *a*_5_, *d*_4_, and *d*_5_ to obtain the noise-removed induction signal, which presents smoother characteristics. The filtered signal also presents obvious multifractal characteristics.

In future work, the denoised electrostatic induction signal will be used for the measurement of dust concentration. Simultaneously, by combining Ensemble Empirical Mode Decomposition [36] (EEMD), Variational Mode Decomposition [38] (VMD) with stronger ability to extract trends, MFDFA's de-trending capabilities will be further improved.

## Figures and Tables

**Table 1:** Wavelet sub-band frequency range of detection signal.

Noise signal decomposition layer	Sub-band frequency
*a*_5_	0–7.4375 Hz
*a*_4_	0–14.875 Hz
*a*_3_	0–29.75 Hz
*a*_2_	0–59.5 Hz
*a*_1_	0–119 Hz
*d*_5_	7.4375–15.625 Hz
*d*_4_	14.875–29.75 Hz
*d*_3_	29.75–59.5 Hz
*d*_2_	59.5–119 Hz
*d*_1_	119–238 Hz

**Table 2:** Time-lapse Pearson correlation.

Analysis object	Max value	Offset time (positive value indicates that noise component leads; negative value indicates that signal component leads)
nos_*a*_1_ − *a*_1_	0.160	0
nos_*a*_2_ − *a*_2_	0.160	0
nos_*a*_3_ − *a*_3_	0.450	0
nos_*a*_4_ − *a*_4_	0.667	0
nos_*a*_5_ − *a*_5_	0.710	2
nos_*d*_1_ − *d*_1_	0.497	–57
nos_*d*_2_ − *d*_2_	0.367	0
nos_*d*_3_ − *d*_3_	0.694	0
nos_*d*_4_ − *d*_4_	0.112	0
nos_*d*_5_ − *d*_5_	0.530	0

**Table 3:** For *q*=2, the Hurst exponent of each sub-band component of the measured signal *h*(*q*).

	*a*_1_	*a*_2_	*a*_3_	*a*_4_	*a*_5_	*d*_1_	*d*_2_	*d*_3_	*d*_4_	*d*_5_
MFDFA	1.087	1.091	1.133	1.211	1.376	0.013	0.074	0.221	0.523	0.784
MFDFA-EMD	0.602	0.567	0.544	0.417	0.266	−0.006	0	0.003	0.002	0
Relevance judgment	Negative correlation	Negative correlation	Negative correlation	Positive correlation	Positive correlation	Positive correlation	Positive correlation	Positive correlation	Positive correlation	Positive correlation

**Table 4:** For *q*=2, the Hurst exponent of each sub-band component of measured noise *h*(*q*).

	nos − *a*_1_	nos − *a*_2_	nos − *a*_3_	nos − *a*_4_	nos − *a*_5_	nos − *d*_1_	nos − *d*_2_	nos − *d*_3_	nos − *d*_4_	nos − *d*_5_
MFDFA	0.377	0.369	1.086	1.274	1.450	0.014	0.107	0.181	0.455	0.762
EMD-MFDFA	0.197	0.141	0.694	0.439	0.185	−0.004	0	0	0.009	−0.002
Relevance judgment	Positive correlation	Positive correlation	Negative correlation	Positive correlation	Positive correlation	Positive correlation	Positive correlation	Positive correlation	Positive correlation	Positive correlation

**Table 5:** R value of signal component.

R value	*a*_1_	*a*_2_	*a*_3_	*a*_4_	*a*_5_	*d*_1_	*d*_2_	*d*_3_	*d*_4_	*d*_5_
MFDFA	0.9990	0.9989	0.9930	0.9876	0.9807	0.7952	0.8194	0.9439	0.9755	0.9712
EMD-MFDFA	0.9794	0.9771	0.9609	0.9559	0.9288	0.3954	0.6324	0.4867	0.7733	0.7351
Fractal characteristics	Single fractal	Single fractal	Single fractal	Fractal characteristics	Fractal characteristics	Fractal characteristics	Fractal characteristics	Fractal characteristics	Fractal characteristics	Fractal characteristics

**Table 6:** R value of noise component.

R value	nos − *a*_1_	nos − *a*_2_	nos − *a*_3_	nos − *a*_4_	nos − *a*_5_	nos − *d*_1_	nos − *d*_2_	nos − *d*_3_	nos − *d*_4_	nos − *d*_5_
MFDFA	0.9954	0.9948	0.9945	0.9912	0.9905	0.7823	0.9500	0.9838	0.9600	0.9628
EMD-MFDFA	0.9815	0.9984	0.9788	0.9334	0.8904	0.6997	0.6327	0.4570	0.7656	0.7587
Fractal characteristics	Single fractal	Single fractal	Single fractal	Fractal characteristics	Fractal characteristics	Fractal characteristics	Fractal characteristics	Fractal characteristics	Fractal characteristics	Fractal characteristics

**Table 7:** Fractal judgment between signal components *α* and *f*(*α*).

	*a*_1_	*a*_2_	*a*_3_	*a*_4_	*a*_5_	*d*_1_	*d*_2_	*d*_3_	*d*_4_	*d*_5_
MFDFA	Non-bell shaped	Non-bell shaped	Non-bell shaped	Bell shaped	Bell shaped	Bell shaped	Bell shaped	Bell shaped	Bell shaped	Bell shaped
EMD-MFDFA	Non-bell shaped	Non-bell shaped	Non-bell shaped	Bell shaped	Bell shaped	Bell shaped	Bell shaped	Bell shaped	Bell shaped	Bell shaped
Fractal characteristics	Single fractal	Single fractal	Single fractal	Multifractal	Multifractal	Multifractal	Multifractal	Multifractal	Multifractal	Multifractal

**Table 8:** Fractal judgment between noise component *α* and *f*(*α*).

	nos − *a*_1_	nos − *a*_2_	nos − *a*_3_	nos − *a*_4_	nos − *a*_5_	nos − *d*_1_	nos − *d*_2_	nos − *d*_3_	nos − *d*_4_	nos − *d*_5_
MFDFA	Non-bell shaped	Non-bell shaped	Non-bell shaped	Bell shaped	Bell shaped	Bell shaped	Bell shaped	Bell shaped	Bell shaped	Bell shaped
EMD-MFDFA	Non-bell shaped	Non-bell shaped	Non-bell shaped	Bell shaped	Bell shaped	Bell shaped	Bell shaped	Non-bell -shaped	Bell shaped	Bell shaped
Fractal characteristics	Single fractal	Single fractal	Single fractal	Multifractal	Multifractal	Multifractal	Multifractal	Single fractal	Multifractal	Multifractal

**Table 9:** Difference in Hurst exponent *h* (*q*) between the noise component and signal component analyzed by EMD-MFDFA.

	*a*_1_–, nos_a_1_	*a*_2_–, nos_*a*_2_	*a*_3_–nos_*a*_3_	*a*_4_–nos_*a*_4_	*a*_5_–nos_*a*_5_	*d*_1_–nos_*d*_1_	*d*_2_–nos_*d*_2_	*d*_3_–nos_*d*_3_	*d*_4_–nos_*d*_4_	*d*_5_–nos_*d*_5_
Correlation	−0.970	−0.593	0.958	0.959	0.971	0.977	0.995	0.989	0.994	0.981
Average error	0.591	0.608	0.006	−0.044	0.131	−0.055	0.074	0.037	0.015	−0.106
Standard deviation	0.068	0.052	0.031	0.012	0.010	0.005	0.016	0.006	0.001	0.029

**Table 10:** Difference in *τ*(*q*) between the noise component and signal component analyzed by EMD-MFDFA.

	*a*_1_–nos_a_1_	*a*_2_–nos_*a*_2_	*a*_3_–nos_*a*_3_	*a*_4_–nos_*a*_4_	*a*_5_–nos_*a*_5_	*d*_1_–nos_*d*_1_	*d*_2_–nos_*d*_2_	*d*_3_–nos_*d*_3_	*d*_4_–nos_*d*_4_	*d*_5_–nos_*d*_5_
Correlation	0.924	0.966	0.997	0.997	0.994	0.921	0.997	0.995	0.998	0.997
Average error	−0.775	−0.676	−0.435	0.154	−0.120	0.207	−0.372	−0.225	−0.059	0.450
Standard deviation	3.949	4.306	0.128	0.044	0.425	0.086	0.225	0.076	0.003	0.503

**Table 11:** Difference in *f*(*α*) between the noise component and signal component analyzed by EMD-MFDFA.

	*a*_1_*–*nos_*a*_1_	*a*_2_*–*nos_*a*_2_	*a*_3_*–*nos_*a*_3_	*a*_4_*–*nos_*a*_4_	*a*_5_*–*nos_*a*_5_	*d*_1_*–*nos_*d*_1_	*d*_2_*–*nos_*d*_2_	*d*_3_*–*nos_*d*_3_	*d*_4_*–*nos_*d*_4_	*d*_5_*–*nos_*d*_5_
Correlation	−0.765	−0.488	0.846	0.774	0.798	0.937	0.897	0.979	0.988	0.877
Average error	−0.368	−0.322	0.017	0.174	0.009	0.132	−0.348	−0.269	0.073	0.305
Standard deviation	0.099	0.077	0.046	0.057	0.058	0.023	0.120	0.092	0.027	0.134

## Data Availability

The data used to support the findings of this study are included within the supplementary information file.

## Supplementary Materials

Supplementary Materials “Measured_noise_data.docx” includes the measured noise used in this paper. “Measured_signal_data.docx” includes the measured signal used in this paper.
