# Supplementary material for: Removal of Dust Microelectric Signal Based on Empirical Mode Decomposition and Multifractal Detrended Fluctuation Analysis
Source: Comput Intell Neurosci. 2021 Aug 6;2021:5468514. doi: 10.1155/2021/5468514 (PMC8367589; doi:10.1155/2021/5468514)
Supplement: Supplementary Materials — “Measured_noise_data.docx” includes the measured noise used in this paper. “Measured_signal_data.docx” includes the measured signal used in this paper. [file 5468514.f1.zip › 5468514.f1/measured_noise_data.docx]

The following data is the measured noise signal in this paper, the sampling rate is 500Hz, and the unit is Voltage (V).

0.21973,0.53833,0.55176,0.41626,0.14038,-0.092773,-0.36621,-0.32227,-0.076904,0.18311,0.57251,0.70801,0.67993,0.47852,0.19531,-0.10254,-0.3418,-0.21851,0.1001,0.33691,0.58105,0.69458,0.52979,0.2417,0.036621,-0.22949,-0.28809,0.021973,0.24414,0.5957,0.85205,0.75195,0.47119,0.24658,-0.0073242,-0.15869,-0.043945,0.15015,0.38452,0.81543,0.89111,0.69336,0.3833,0.084229,-0.18921,-0.16724,0.092773,0.31738,0.57373,0.81543,0.81543,0.54199,0.21973,-0.012207,-0.22339,-0.10986,0.16846,0.42969,0.65186,0.73853,0.63477,0.35522,0.098877,-0.13916,-0.29541,-0.091553,0.25391,0.55054,0.74341,0.7251,0.46753,0.17334,-0.053711,-0.27344,-0.15381,0.11108,0.36255,0.63354,0.72388,0.53345,0.18066,0.014648,-0.14893,-0.33203,-0.1709,0.15381,0.39307,0.61279,0.65063,0.40283,0.095215,-0.14038,-0.30762,-0.26123,-0.026855,0.23926,0.52246,0.63477,0.56519,0.2832,-0.05249,-0.33569,-0.41504,-0.23315,0.029297,0.3418,0.56641,0.57129,0.36743,0.070801,-0.20508,-0.41992,-0.34668,-0.11841,0.18433,0.43213,0.47729,0.44922,0.16968,-0.15137,-0.39307,-0.4895,-0.41504,-0.14526,0.15503,0.3833,0.36987,0.15503,-0.15015,-0.49316,-0.66284,-0.57617,-0.44189,-0.15869,0.20874,0.32837,0.21973,-0.021973,-0.38086,-0.68481,-0.74097,-0.52612,-0.22949,0.090332,0.31738,0.33203,0.15015,-0.11475,-0.41382,-0.64819,-0.57983,-0.35156,-0.041504,0.27832,0.34668,0.32715,0.065918,-0.25879,-0.58472,-0.79468,-0.59448,-0.27832,-0.024414,0.27832,0.31128,0.073242,-0.15381,-0.45898,-0.82031,-0.66528,-0.33936,-0.18677,0.16235,0.2771,0.1001,-0.13428,-0.33203,-0.62744,-0.78857,-0.61279,-0.34058,-0.045166,0.24048,0.30273,0.14282,-0.14648,-0.44434,-0.67139,-0.67871,-0.41138,-0.11841,0.18188,0.36133,0.32227,0.056152,-0.33081,-0.58228,-0.75317,-0.5249,-0.22217,0.050049,0.2832,0.40649,0.23926,-0.03418,-0.26733,-0.57617,-0.56885,-0.24414,0.01709,0.30762,0.44189,0.3894,0.14771,-0.1355,-0.37354,-0.63965,-0.55908,-0.21973,0.11841,0.34912,0.46021,0.30762,-0.028076,-0.24902,-0.49561,-0.53101,-0.22339,0.0024414,0.42725,0.54443,0.45654,0.18799,-0.1416,-0.33936,-0.46753,-0.37109,-0.1123,0.15869,0.42358,0.53467,0.44556,0.11475,-0.18311,-0.41992,-0.47607,-0.26611,0.05249,0.32349,0.50781,0.5481,0.40405,0.059814,-0.20386,-0.38574,-0.28931,-0.020752,0.33325,0.60059,0.60425,0.5127,0.26367,-0.042725,-0.27832,-0.29419,-0.14282,0.15625,0.5603,0.71533,0.62012,0.44434,0.1355,-0.12939,-0.24658,-0.23438,-0.041504,0.22095,0.49561,0.63965,0.5127,0.2002,-0.0061035,-0.25024,-0.27466,-0.12085,0.15991,0.48828,0.71045,0.69336,0.47607,0.1355,-0.19287,-0.35889,-0.23926,0.015869,0.28564,0.60913,0.60303,0.49072,0.35278,0.10742,-0.22461,-0.26245,-0.072021,0.18555,0.42847,0.70557,0.65674,0.43091,0.20752,-0.081787,-0.37964,-0.26978,-0.0048828,0.2417,0.57861,0.73242,0.48218,0.22461,-0.024414,-0.29297,-0.3186,-0.15015,0.090332,0.39795,0.66162,0.65186,0.45654,0.16602,-0.15747,-0.31982,-0.27588,-0.091553,0.18188,0.39795,0.59326,0.52246,0.24292,-0.041504,-0.26245,-0.45288,-0.23193,0.089111,0.30029,0.52246,0.59448,0.32715,0.05127,-0.20386,-0.49194,-0.47485,-0.18433,0.1123,0.37964,0.50049,0.40283,0.12817,-0.13672,-0.37354,-0.53223,-0.4187,-0.13428,0.17578,0.42847,0.49316,0.29053,-0.014648,-0.26855,-0.49072,-0.51514,-0.29419,-0.0024414,0.25146,0.41138,0.31494,0.03418,-0.19165,-0.46875,-0.60913,-0.4248,-0.20264,0.087891,0.34912,0.43213,0.33203,-0.023193,-0.37354,-0.5957,-0.54321,-0.29907,0.032959,0.25757,0.39063,0.32837,0.13306,-0.17822,-0.47607,-0.62378,-0.44922,-0.16724,0.15259,0.38574,0.40527,0.30396,0.023193,-0.29541,-0.5957,-0.62744,-0.40405,-0.13428,0.24658,0.34668,0.22705,0.019531,-0.26367,-0.46631,-0.62134,-0.54077,-0.28931,0.029297,0.30151,0.36621,0.23071,-0.080566,-0.41016,-0.54077,-0.50537,-0.33813,-0.10376,0.2063,0.38086,0.34668,0.16602,-0.11597,-0.448,-0.63965,-0.47363,-0.21484,0.043945,0.36621,0.39551,0.26245,-0.0097656,-0.24414,-0.57129,-0.61035,-0.27466,-0.1062,0.2002,0.45898,0.37354,0.14038,-0.096436,-0.34912,-0.5188,-0.354,-0.13062,0.16113,0.47607,0.50537,0.37354,0.12817,-0.15015,-0.42236,-0.48706,-0.29541,-0.072021,0.28564,0.49927,0.47363,0.23438,0.061035,-0.29175,-0.49805,-0.33081,-0.10498,0.1123,0.36377,0.54565,0.41992,0.11597,-0.1416,-0.36133,-0.48462,-0.23804,0.05249,0.39551,0.60181,0.61523,0.36865,0.10986,-0.18066,-0.44678,-0.3479,-0.063477,0.23926,0.54199,0.63354,0.51025,0.26123,0.01709,-0.2356,-0.40161,-0.20996,0.13672,0.40527,0.61279,0.62378,0.32227,0.015869,-0.19043,-0.37231,-0.30151,-0.050049,0.22705,0.47119,0.62744,0.55664,0.25757,-0.050049,-0.23926,-0.36499,-0.23315,0.059814,0.30762,0.57007,0.59082,0.38696,0.061035,-0.23804,-0.44067,-0.36011,-0.12329,0.21973,0.47729,0.62134,0.54565,0.28687,-0.037842,-0.23193,-0.34424,-0.21973,0.075684,0.31982,0.50903,0.5603,0.37354,0.053711,-0.19409,-0.40405,-0.37231,-0.16479,0.17822,0.49805,0.61523,0.54932,0.28442,-0.043945,-0.29785,-0.43213,-0.20874,0.084229,0.35889,0.65186,0.53467,0.32471,0.15137,-0.16968,-0.39429,-0.27344,-0.10254,0.1709,0.50049,0.60791,0.52856,0.31738,-0.021973,-0.34912,-0.38086,-0.2417,-0.050049,0.18921,0.46509,0.52368,0.39795,0.073242,-0.26855,-0.53467,-0.42236,-0.18677,0.092773,0.38696,0.53711,0.36377,0.16602,-0.029297,-0.41504,-0.5542,-0.40039,-0.13062,0.14404,0.42114,0.42847,0.24414,-0.046387,-0.31006,-0.58716,-0.58838,-0.35278,-0.081787,0.26611,0.41748,0.37598,0.10986,-0.14282,-0.38696,-0.61035,-0.41748,-0.090332,0.043945,0.38208,0.49438,0.30518,-0.01709,-0.27954,-0.56885,-0.64941,-0.30518,-0.030518,0.32715,0.48828,0.41992,0.087891,-0.16846,-0.40771,-0.61035,-0.45166,-0.16968,0.087891,0.38086,0.46753,0.32104,0.0097656,-0.26367,-0.54932,-0.63721,-0.38452,-0.11475,0.2002,0.41626,0.42236,0.18311,-0.13794,-0.34912,-0.53955,-0.4248,-0.16724,0.16724,0.37109,0.43701,0.30273,-0.0085449,-0.30396,-0.46753,-0.54443,-0.32593,-0.015869,0.32104,0.49561,0.47119,0.30396,-0.012207,-0.27466,-0.51025,-0.45044,-0.14648,0.17578,0.39063,0.54321,0.354,0.040283,-0.19897,-0.45166,-0.52246,-0.3833,-0.031738,0.27344,0.41626,0.41626,0.22461,-0.11719,-0.34668,-0.50781,-0.43091,-0.21362,0.068359,0.47363,0.46997,0.39917,0.22949,-0.087891,-0.40283,-0.52002,-0.26855,0.065918,0.32715,0.5127,0.47974,0.25879,0.097656,-0.23193,-0.51025,-0.30151,-0.1062,0.177,0.50781,0.57617,0.44189,0.24414,-0.08667,-0.35522,-0.36133,-0.24902,-0.1123,0.2478,0.52246,0.5127,0.33081,0.075684,-0.23193,-0.40405,-0.31372,-0.1416,0.12939,0.448,0.63477,0.53833,0.25513,-0.040283,-0.3186,-0.44678,-0.23315,0.05127,0.35278,0.62012,0.62012,0.39551,0.14648,-0.12207,-0.38696,-0.33691,-0.063477,0.18188,0.5603,0.67017,0.57861,0.35645,0.078125,-0.18799,-0.41748,-0.27466,-0.0073242,0.24536,0.53345,0.61523,0.36133,0.067139,-0.19043,-0.49561,-0.36499,-0.040283,0.21729,0.4834,0.67993,0.56274,0.26978,0.023193,-0.22949,-0.43457,-0.31494,-0.0048828,0.19043,0.44922,0.52979,0.35767,0.097656,-0.19531,-0.41504,-0.4248,-0.16724,0.13794,0.41626,0.51392,0.45532,0.19043,-0.10132,-0.29541,-0.46509,-0.29907,-0.036621,0.23438,0.44922,0.52368,0.36987,0.043945,-0.22339,-0.40405,-0.46021,-0.22339,0.084229,0.36865,0.50537,0.56641,0.30762,-0.058594,-0.30396,-0.49561,-0.41992,-0.12451,0.16113,0.36377,0.47852,0.35034,0.039063,-0.23926,-0.47852,-0.50537,-0.28076,0.0048828,0.30029,0.46753,0.36743,0.177,-0.12451,-0.34546,-0.53955,-0.43579,-0.13794,0.058594,0.44678,0.40771,0.28564,0.059814,-0.22217,-0.50293,-0.52124,-0.32227,-0.012207,0.32227,0.49683,0.4541,0.30762,-0.046387,-0.35645,-0.51514,-0.41016,-0.13672,0.13794,0.43579,0.35278,0.2478,0.0061035,-0.27466,-0.53223,-0.57007,-0.35278,-0.075684,0.26489,0.46143,0.39795,0.2124,-0.045166,-0.35767,-0.55786,-0.43945,-0.16113,0.015869,0.39063,0.49072,0.3479,0.057373,-0.24902,-0.53467,-0.57007,-0.32471,-0.062256,0.27222,0.48096,0.45776,0.22949,-0.043945,-0.33936,-0.56152,-0.44189,-0.18066,0.064697,0.4126,0.52979,0.39551,0.10742,-0.15137,-0.44556,-0.57129,-0.31006,-0.0097656,0.26367,0.54077,0.56396,0.29907,0.01709,-0.23438,-0.50171,-0.39551,-0.11353,0.1123,0.38574,0.52002,0.35889,0.063477,-0.2124,-0.45898,-0.55176,-0.33813,-0.058594,0.22461,0.47852,0.50293,0.30762,0.065918,-0.20508,-0.43945,-0.42358,-0.17212,0.14648,0.40039,0.5835,0.46387,0.18677,-0.11108,-0.29541,-0.44067,-0.26611,0.080566,0.31494,0.54688,0.57129,0.33691,0.014648,-0.22583,-0.40405,-0.39307,-0.14404,0.15137,0.43213,0.56152,0.50415,0.26367,-0.068359,-0.33569,-0.50659,-0.36377,-0.05249,0.24414,0.48828,0.57129,0.41138,0.076904,-0.18433,-0.36621,-0.36987,-0.091553,0.23315,0.45654,0.57495,0.50049,0.25879,-0.067139,-0.25757,-0.39063,-0.30396,-0.039063,0.271,0.55664,0.57373,0.47119,0.1416,-0.19897,-0.43213,-0.39429,-0.21973,0.074463,0.41138,0.60425,0.49072,0.27832,-0.031738,-0.30762,-0.40649,-0.27222,0,0.29175,0.53467,0.55664,0.43213,0.13184,-0.16479,-0.39185,-0.3833,-0.20264,0.085449,0.42847,0.54199,0.47852,0.28076,0.0073242,-0.32593,-0.46387,-0.36621,-0.098877,0.20996,0.50293,0.54443,0.39551,0.097656,-0.16724,-0.43457,-0.45166,-0.16846,0.098877,0.39795,0.59204,0.49316,0.22461,-0.031738,-0.31006,-0.52368,-0.40161,-0.11475,0.10986,0.42236,0.50903,0.35278,0.068359,-0.13184,-0.50049,-0.51025,-0.19287,0.039063,0.31128,0.54565,0.49561,0.25269,-0.020752,-0.37109,-0.58105,-0.42969,-0.16357,0.12329,0.41626,0.4834,0.34912,0.043945,-0.22339,-0.44434,-0.50049,-0.31128,-0.0354,0.25146,0.44189,0.4126,0.29053,-0.05127,-0.33691,-0.54199,-0.46509,-0.19165,0.13184,0.35278,0.5249,0.35767,0.025635,-0.25757,-0.43213,-0.5896,-0.35522,-0.05249,0.24658,0.43945,0.43579,0.14893,-0.15503,-0.39185,-0.50537,-0.45654,-0.19775,0.10132,0.39673,0.5127,0.43579,0.16113,-0.15869,-0.38696,-0.52124,-0.34546,0,0.27466,0.4126,0.4248,0.21729,-0.08667,-0.36377,-0.57861,-0.49072,-0.27588,0.061035,0.32227,0.39673,0.33936,0.097656,-0.24658,-0.47363,-0.54688,-0.41382,-0.085449,0.22461,0.4895,0.46509,0.27222,0.0012207,-0.32593,-0.51758,-0.43823,-0.26367,0.024414,0.30273,0.46387,0.3772,0.14893,-0.16846,-0.40405,-0.44067,-0.25391,-0.050049,0.24292,0.50537,0.50049,0.33203,0.0354,-0.30396,-0.52979,-0.41016,-0.23193,0.065918,0.42236,0.48096,0.42847,0.19287,-0.11108,-0.36865,-0.49561,-0.30762,-0.015869,0.25024,0.49805,0.55786,0.35767,0.079346,-0.19775,-0.45532,-0.4541,-0.22339,0.067139,0.45898,0.52124,0.45532,0.19287,-0.078125,-0.33936,-0.48828,-0.29419,-0.039063,0.20996,0.55664,0.5957,0.36377,0.092773,-0.18677,-0.44678,-0.39795,-0.15869,0.048828,0.31738,0.53711,0.48462,0.2356,-0.03418,-0.28198,-0.43945,-0.26855,0,0.25269,0.53345,0.59204,0.42725,0.14404,-0.1709,-0.40283,-0.4541,-0.25146,0.079346,0.49316,0.5188,0.56396,0.29175,-0.053711,-0.2356,-0.44189,-0.37964,-0.043945,0.23193,0.44189,0.57739,0.4541,0.090332,-0.12451,-0.36133,-0.4834,-0.19409,0.10498,0.37598,0.55664,0.45898,0.20874,-0.089111,-0.31982,-0.48584,-0.38086,-0.095215,0.18677,0.48096,0.55664,0.43945,0.10254,-0.20386,-0.42725,-0.47852,-0.22217,-0.057373,0.27832,0.45532,0.44434,0.25879,-0.079346,-0.33691,-0.46631,-0.37598,-0.16479,0.14893,0.3894,0.46875,0.48584,0.16846,-0.15747,-0.41138,-0.53833,-0.3186,0.0012207,0.30273,0.46387,0.44678,0.28564,0.0048828,-0.30029,-0.5249,-0.42725,-0.21973,0.1001,0.36621,0.46143,0.34668,0.090332,-0.21606,-0.42847,-0.46631,-0.31982,-0.065918,0.26001,0.4541,0.4248,0.2063,-0.084229,-0.38818,-0.57617,-0.40039,-0.16602,0.11963,0.43823,0.48828,0.37109,0.10254,-0.2356,-0.52856,-0.60425,-0.46387,-0.19409,0.061035,0.27588,0.25879,0.048828,-0.17822,-0.42847,-0.64453,-0.52124,-0.25635,0.0354,0.36621,0.51025,0.3479,0.15503,-0.083008,-0.37964,-0.54199,-0.34058,-0.040283,0.14526,0.46509,0.44556,0.21973,-0.0085449,-0.30029,-0.50903,-0.38574,-0.1709,0.10254,0.3894,0.54443,0.46021,0.21729,-0.10376,-0.35645,-0.46753,-0.29663,-0.014648,0.25635,0.47852,0.52368,0.29053,-0.0036621,-0.26855,-0.54688,-0.47852,-0.21118,0.10498,0.3894,0.55298,0.46875,0.15991,-0.092773,-0.32715,-0.48706,-0.36865,-0.056152,0.18188,0.41504,0.5188,0.32471,-0.015869,-0.23804,-0.49805,-0.46997,-0.20508,0.070801,0.34302,0.54199,0.44312,0.14038,-0.13184,-0.34302,-0.44678,-0.30273,0.0036621,0.22217,0.51636,0.56274,0.38696,0.087891,-0.23926,-0.4895,-0.43457,-0.20508,0.1001,0.36865,0.52002,0.51758,0.29907,-0.037842,-0.25391,-0.40039,-0.26978,0.043945,0.32471,0.55664,0.52246,0.40405,0.085449,-0.23804,-0.44922,-0.43701,-0.18799,0.019531,0.47119,0.51758,0.49927,0.25879,-0.078125,-0.28198,-0.45044,-0.33203,-0.08667,0.19531,0.4541,0.52612,0.36621,0.065918,-0.22461,-0.46265,-0.47729,-0.24048,0.045166,0.3479,0.52002,0.47241,0.29053,-0.036621,-0.27466,-0.45776,-0.35767,-0.13672,0.17334,0.448,0.5249,0.39551,0.11108,-0.17578,-0.43823,-0.45654,-0.21973,0.028076,0.31128,0.55298,0.47852,0.27832,-0.0036621,-0.26367,-0.50781,-0.29053,-0.13428,0.14404,0.42358,0.47852,0.32959,0.050049,-0.22217,-0.45654,-0.55176,-0.37231,-0.097656,0.27588,0.54932,0.43579,0.21362,-0.065918,-0.34058,-0.50293,-0.38818,-0.15869,0.10498,0.37598,0.57007,0.4248,0.10254,-0.20142,-0.42847,-0.52979,-0.28564,0.045166,0.26611,0.47119,0.50659,0.23682,-0.067139,-0.26367,-0.53711,-0.39551,-0.15747,0.1001,0.39307,0.50293,0.38818,0.1001,-0.16846,-0.41504,-0.54932,-0.36133,-0.048828,0.25391,0.52612,0.5188,0.26001,-0.070801,-0.29785,-0.53711,-0.45044,-0.21606,0.065918,0.30518,0.46997,0.36865,0.053711,-0.22705,-0.43945,-0.55786,-0.39185,-0.023193,0.23682,0.46265,0.52612,0.26978,0.05127,-0.26611,-0.51636,-0.47363,-0.26245,0.063477,0.30762,0.42969,0.38086,0.16602,-0.17334,-0.42725,-0.54077,-0.41382,-0.11108,0.2063,0.3833,0.41992,0.25146,-0.062256,-0.33325,-0.45166,-0.47119,-0.19287,0.1416,0.43945,0.53467,0.4834,0.20142,-0.13184,-0.31372,-0.46143,-0.31372,-0.12207,0.16113,0.43579,0.50049,0.36133,0.085449,-0.25146,-0.46387,-0.36377,-0.13672,0.10254,0.43823,0.58472,0.46387,0.27588,-0.072021,-0.37354,-0.50293,-0.38574,-0.12939,0.16602,0.52368,0.46021,0.32471,0.1355,-0.16846,-0.44189,-0.41382,-0.20874,0.064697,0.36743,0.58228,0.46143,0.24048,-0.013428,-0.26611,-0.46631,-0.25269,-0.054932,0.15015,0.54199,0.49927,0.31006,0.15015,-0.13916,-0.40527,-0.39551,-0.15381,0.098877,0.43213,0.62622,0.53101,0.31494,0.0048828,-0.29419,-0.49683,-0.36865,-0.12573,0.12695,0.35645,0.4834,0.36133,0.056152,-0.18188,-0.42603,-0.50293,-0.22949,0.08667,0.354,0.55664,0.53467,0.33447,0.053711,-0.20996,-0.46753,-0.36865,-0.10498,0.15503,0.43701,0.53223,0.3833,0.05127,-0.12207,-0.41992,-0.50049,-0.23315,0.067139,0.28076,0.59082,0.55176,0.29053,0.014648,-0.18799,-0.45898,-0.30151,-0.046387,0.23682,0.48584,0.58472,0.37964,0.16357,-0.13672,-0.43213,-0.40283,-0.30273,0,0.31738,0.52002,0.55176,0.36011,0.046387,-0.24414,-0.44922,-0.37109,-0.11597,0.16968,0.3772,0.50293,0.39673,0.10742,-0.17334,-0.38818,-0.48096,-0.27588,0.063477,0.34912,0.51392,0.5127,0.28564,-0.020752,-0.26245,-0.41992,-0.39795,-0.18677,0.15869,0.40039,0.45654,0.40527,0.11475,-0.19409,-0.42725,-0.48096,-0.354,0.0061035,0.28931,0.55786,0.54321,0.3186,0.030518,-0.29053,-0.49927,-0.3833,-0.15991,0.10986,0.40771,0.53223,0.46021,0.10864,-0.19165,-0.40527,-0.5188,-0.33447,-0.037842,0.24902,0.47119,0.45532,0.31128,0.065918,-0.3064,-0.54688,-0.43823,-0.271,0.024414,0.30762,0.48462,0.33936,0.12695,-0.17334,-0.43701,-0.57373,-0.2771,-0.10254,0.19165,0.49316,0.54443,0.33569,0.073242,-0.22461,-0.4834,-0.39795,-0.18799,0.10986,0.42358,0.53101,0.46631,0.22217,-0.13184,-0.38452,-0.53345,-0.38452,-0.098877,0.12329,0.4834,0.5542,0.354,0.10742,-0.17944,-0.47852,-0.45288,-0.14893,0.031738,0.3064,0.51392,0.41016,0.12329,-0.087891,-0.32227,-0.53101,-0.35034,-0.0354,0.20874,0.52979,0.58716,0.41016,0.084229,-0.18311,-0.44556,-0.43823,-0.24292,0.045166,0.26855,0.46875,0.37109,0.1709,-0.11475,-0.33203,-0.49438,-0.38208,-0.10254,0.21973,0.50537,0.58228,0.43945,0.13428,-0.13794,-0.36377,-0.39429,-0.18433,0.1123,0.38574,0.51636,0.53467,0.24536,-0.1123,-0.31372,-0.47485,-0.39673,-0.10254,0.16602,0.46387,0.57617,0.49561,0.14404,-0.14893,-0.33203,-0.4187,-0.18433,0.12329,0.3894,0.50537,0.46997,0.25635,-0.037842,-0.29907,-0.49316,-0.40894,-0.10864,0.18433,0.5249,0.5896,0.46509,0.20386,-0.092773,-0.34546,-0.41016,-0.18555,0.1123,0.41016,0.60791,0.48218,0.2771,-0.046387,-0.29663,-0.46997,-0.39185,-0.080566,0.22339,0.51636,0.57129,0.46387,0.23193,-0.13916,-0.37354,-0.36011,-0.25269,-0.024414,0.35889,0.56519,0.50781,0.34668,0.028076,-0.29785,-0.48096,-0.36865,-0.14648,0.15381,0.41016,0.56274,0.44922,0.21729,-0.14404,-0.36377,-0.45166,-0.25635,0.039063,0.34424,0.53711,0.51758,0.31494,0.020752,-0.26855,-0.47729,-0.37476,-0.14648,0.14038,0.4895,0.59814,0.43945,0.21362,-0.057373,-0.36133,-0.46631,-0.25879,-0.020752,0.20752,0.52002,0.50293,0.25879,-0.0085449,-0.24536,-0.50903,-0.42358,-0.16113,0.12695,0.4187,0.55908,0.44189,0.15869,-0.11841,-0.36499,-0.50293,-0.35645,-0.040283,0.20996,0.50049,0.52734,0.30396,0.010986,-0.31616,-0.58838,-0.49194,-0.25024,0.029297,0.32227,0.52246,0.40527,0.14282,-0.10742,-0.35645,-0.48706,-0.33936,-0.0073242,0.22827,0.43091,0.5127,0.32715,0.0048828,-0.25391,-0.46997,-0.46997,-0.271,0.06958,0.37598,0.47607,0.47363,0.20508,-0.11475,-0.33569,-0.47607,-0.36987,-0.048828,0.22705,0.45044,0.53955,0.3894,0.031738,-0.26245,-0.51514,-0.48828,-0.21484,0.057373,0.3064,0.44434,0.40649,0.16846,-0.10742,-0.33569,-0.5127,-0.38086,-0.096436,0.16602,0.47852,0.50049,0.36865,0.074463,-0.25879,-0.51147,-0.49438,-0.25635,0.03418,0.38696,0.5481,0.36011,0.18066,-0.12207,-0.36621,-0.42969,-0.31738,-0.1123,0.2063,0.50171,0.5188,0.37842,0.092773,-0.23438,-0.46387,-0.45166,-0.26611,-0.028076,0.32715,0.47241,0.44678,0.2356,-0.08667,-0.3418,-0.52246,-0.40771,-0.12695,0.17334,0.40771,0.50537,0.36987,0.098877,-0.17578,-0.43701,-0.4895,-0.25146,-0.031738,0.32593,0.53223,0.41504,0.21362,-0.029297,-0.29785,-0.53955,-0.30762,-0.061035,0.2063,0.50537,0.62988,0.42236,0.13184,-0.090332,-0.44189,-0.43579,-0.19043,-0.026855,0.2002,0.49316,0.43823,0.23438,-0.053711,-0.29419,-0.50537,-0.3418,-0.13184,0.15625,0.46021,0.59326,0.38696,0.11963,-0.075684,-0.37842,-0.48096,-0.25635,0.070801,0.36133,0.61035,0.49316,0.25269,-0.015869,-0.2356,-0.46143,-0.41504,-0.11963,0.16846,0.39185,0.54932,0.46143,0.1123,-0.11719,-0.33691,-0.49805,-0.33447,-0.0012207,0.26367,0.45654,0.49072,0.26978,-0.03418,-0.26855,-0.48462,-0.42114,-0.16479,0.11841,0.42725,0.5542,0.51758,0.2002,-0.12207,-0.38208,-0.48096,-0.32227,-0.024414,0.18799,0.39673,0.47363,0.26489,-0.029297,-0.29541,-0.47241,-0.36621,-0.12451,0.19165,0.44189,0.53223,0.50293,0.26123,-0.078125,-0.29663,-0.41748,-0.32104,-0.0073242,0.19531,0.44434,0.44556,0.25635,-0.020752,-0.31372,-0.48096,-0.39917,-0.18677,0.11963,0.39551,0.56152,0.44067,0.2124,-0.079346,-0.31494,-0.45044,-0.34058,-0.048828,0.27466,0.50171,0.53223,0.37231,0.046387,-0.28442,-0.49194,-0.43335,-0.20874,0.068359,0.39063,0.48218,0.40771,0.17212,-0.12695,-0.40649,-0.5249,-0.36743,-0.10376,0.16235,0.41992,0.51392,0.3418,0.096436,-0.21118,-0.54688,-0.48584,-0.14893,-0.012207,0.33447,0.5127,0.3833,0.16113,-0.075684,-0.35645,-0.56152,-0.36621,-0.079346,0.19165,0.47974,0.49683,0.31006,0.053711,-0.2063,-0.45654,-0.43945,-0.2124,0.0354,0.33936,0.60059,0.42358,0.20142,-0.096436,-0.33325,-0.5542,-0.34058,-0.10742,0.10742,0.36743,0.48584,0.30396,-0.0085449,-0.2417,-0.45654,-0.53223,-0.20508,-0.0085449,0.27588,0.50781,0.4834,0.21729,-0.059814,-0.26855,-0.4834,-0.38452,-0.12085,0.13428,0.36987,0.50537,0.33081,0.018311,-0.19287,-0.42114,-0.49805,-0.26489,0.018311,0.30884,0.5249,0.48462,0.24414,-0.036621,-0.2417,-0.46509,-0.40039,-0.10742,0.16968,0.43457,0.53223,0.41504,0.06958,-0.20386,-0.43701,-0.47852,-0.26855,0.10498,0.32837,0.53833,0.56152,0.35034,0.010986,-0.22583,-0.40283,-0.30273,-0.041504,0.26611,0.49316,0.48584,0.3894,0.093994,-0.19653,-0.41016,-0.50049,-0.29785,-0.0073242,0.33203,0.5249,0.49561,0.30273,-0.020752,-0.29663,-0.46387,-0.34058,-0.096436,0.23071,0.45898,0.55664,0.47119,0.2002,-0.15869,-0.36865,-0.46753,-0.29053,0.0354,0.35278,0.47363,0.48462,0.29297,0.0061035,-0.30273,-0.47852,-0.39673,-0.22461,0.056152,0.46509,0.54932,0.43579,0.11841,-0.19897,-0.448,-0.57129,-0.40527,-0.14648,0.14648,0.44678,0.46143,0.24414,0.018311,-0.28442,-0.48096,-0.33936,-0.12939,0.15625,0.47485,0.56641,0.48462,0.17334,-0.065918,-0.43823,-0.53223,-0.28198,-0.081787,0.19409,0.50171,0.49683,0.31128,0.078125,-0.25635,-0.53101,-0.40161,-0.15503,0.090332,0.33936,0.52734,0.40039,0.12207,-0.16846,-0.40039,-0.55542,-0.37354,-0.14404,0.091553,0.3894,0.43091,0.19287,-0.16357,-0.44922,-0.66528,-0.61035,-0.40039,-0.14771,0.22583,0.44434,0.38574,0.14282,-0.13184,-0.37109,-0.55908,-0.37842,-0.048828,0.20508,0.46753,0.56274,0.26978,0.073242,-0.15381,-0.45288,-0.48828,-0.14893,0.14771,0.39307,0.57861,0.49927,0.096436,-0.039063,-0.33569,-0.5957,-0.39063,-0.14526,0.11475,0.43701,0.50537,0.38696,0.097656,-0.2002,-0.45044,-0.44067,-0.24658,0.05127,0.33691,0.54199,0.50049,0.24902,-0.058594,-0.31982,-0.52246,-0.36011,-0.068359,0.2002,0.41748,0.4834,0.39673,0.062256,-0.25391,-0.45654,-0.52002,-0.32104,0.036621,0.32959,0.44189,0.46875,0.24414,-0.080566,-0.3479,-0.51514,-0.45654,-0.16235,0.14648,0.41138,0.5127,0.40894,0.15747,-0.16357,-0.38818,-0.41748,-0.21606,0.048828,0.36255,0.5542,0.49316,0.28809,-0.062256,-0.31494,-0.47363,-0.37476,-0.15137,0.15503,0.48096,0.56519,0.35889,0.090332,-0.22583,-0.46265,-0.46875,-0.29663,-0.012207,0.34424,0.5481,0.51514,0.31128,0,-0.2771,-0.47729,-0.33691,-0.12451,0.12329,0.44678,0.53711,0.39429,0.13062,-0.1416,-0.38818,-0.48706,-0.29297,-0.030518,0.28564,0.5481,0.50537,0.27832,0.029297,-0.25879,-0.48828,-0.41382,-0.18188,0.075684,0.38696,0.52856,0.36987,0.15869,-0.12207,-0.37231,-0.44922,-0.26978,0.0012207,0.30762,0.54443,0.48828,0.23926,0.042725,-0.24048,-0.48096,-0.41138,-0.16113,0.12085,0.34424,0.49805,0.45898,0.16846,-0.11841,-0.3064,-0.46387,-0.26611,0.078125,0.25269,0.54443,0.61279,0.33325,0.058594,-0.20752,-0.52124,-0.45776,-0.15747,0.096436,0.35767,0.50415,0.41138,0.14648,-0.10742,-0.32959,-0.45654,-0.30029,-0.0048828,0.27588,0.5542,0.56885,0.37109,0.05127,-0.21606,-0.42847,-0.40161,-0.2002,0.064697,0.31494,0.50415,0.44556,0.20508,-0.15137,-0.354,-0.47363,-0.36499,-0.043945,0.26489,0.4248,0.50781,0.35034,0.0048828,-0.22217,-0.48218,-0.47485,-0.20752,0.10986,0.44678,0.49072,0.448,0.12817,-0.13794,-0.36133,-0.48462,-0.37964,-0.090332,0.23438,0.47974,0.55664,0.43579,0.10254,-0.17822,-0.39063,-0.38696,-0.18433,0.089111,0.38574,0.53955,0.47852,0.25879,-0.080566,-0.32349,-0.45654,-0.31494,-0.063477,0.25391,0.52002,0.56274,0.43945,0.13184,-0.17334,-0.43579,-0.43213,-0.22949,0.018311,0.30396,0.50659,0.40283,0.20142,-0.090332,-0.35522,-0.52856,-0.27222,-0.1416,0.14893,0.53955,0.56885,0.43579,0.15869,-0.15137,-0.45898,-0.45288,-0.25757,-0.010986,0.37842,0.53223,0.43457,0.26123,-0.048828,-0.33447,-0.49194,-0.33813,-0.092773,0.17822,0.47119,0.50049,0.354,0.056152,-0.17578,-0.46997,-0.50537,-0.2356,-0.018311,0.29541,0.45166,0.40161,0.17578,-0.013428,-0.37476,-0.52856,-0.41748,-0.18555,0.076904,0.41992,0.53345,0.44189,0.12207,-0.11841,-0.37598,-0.51636,-0.23804,0.043945,0.28687,0.56152,0.53833,0.26611,-0.025635,-0.25391,-0.51147,-0.37231,-0.089111,0.16235,0.42725,0.56641,0.3833,0.12451,-0.1416,-0.3772,-0.4895,-0.24414,-0.05127,0.26978,0.48828,0.50171,0.29175,-0.048828,-0.31372,-0.46265,-0.43945,-0.18311,0.15625,0.37476,0.54321,0.47607,0.14771,-0.15991,-0.32227,-0.48096,-0.29541,0.0048828,0.2417,0.46753,0.50293,0.28931,-0.0061035,-0.25879,-0.47974,-0.42725,-0.20264,0.12085,0.45654,0.55664,0.48828,0.21973,-0.090332,-0.30029,-0.44434,-0.3479,-0.0097656,0.31982,0.53711,0.54932,0.34912,0.029297,-0.24048,-0.46143,-0.38574,-0.177,0.12207,0.42603,0.51636,0.42114,0.19409,-0.12207,-0.354,-0.43091,-0.31372,-0.053711,0.21973,0.4541,0.49072,0.36377,0.025635,-0.21606,-0.52124,-0.41992,-0.19531,0.12207,0.37842,0.53711,0.47852,0.26978,-0.063477,-0.35645,-0.46143,-0.24658,0.019531,0.29175,0.56396,0.52979,0.34912,0.10254,-0.17944,-0.4895,-0.43213,-0.20996,0.067139,0.43823,0.59692,0.44922,0.20996,-0.050049,-0.31616,-0.4248,-0.35522,-0.097656,0.19043,0.46265,0.53467,0.34912,-0.01709,-0.25879,-0.50903,-0.48706,-0.17456,0.085449,0.36133,0.54443,0.47852,0.21118,-0.029297,-0.29541,-0.5127,-0.29785,-0.020752,0.20874,0.47852,0.57617,0.41504,0.12695,-0.10986,-0.47729,-0.55176,-0.25391,0.026855,0.33936,0.54932,0.47119,0.24292,-0.040283,-0.28809,-0.49316,-0.35034,-0.11108,0.19531,0.46875,0.53711,0.35156,0.05249,-0.21729,-0.43579,-0.52979,-0.27344,-0.021973,0.30762,0.46387,0.45166,0.22217,-0.11597,-0.33203,-0.52612,-0.45776,-0.16479,0.15747,0.39307,0.58716,0.44189,0.12695,-0.15869,-0.45776,-0.53589,-0.27588,0.013428,0.31128,0.49072,0.45654,0.27832,-0.085449,-0.34912,-0.51392,-0.36865,-0.12085,0.23682,0.49316,0.55786,0.50537,0.2002,-0.13916,-0.42725,-0.52124,-0.3479,0.0097656,0.31006,0.52979,0.52979,0.33325,-0.018311,-0.33691,-0.448,-0.39795,-0.14648,0.15381,0.42725,0.53589,0.42969,0.15015,-0.15625,-0.39185,-0.48218,-0.31738,-0.075684,0.26978,0.52734,0.45654,0.31616,0.025635,-0.34424,-0.49194,-0.35889,-0.2124,0.072021,0.39429,0.47607,0.40039,0.17944,-0.15503,-0.41992,-0.47974,-0.31372,-0.029297,0.28687,0.51392,0.47241,0.28931,0.014648,-0.26367,-0.47363,-0.41626,-0.15015,0.13306,0.48096,0.53711,0.43213,0.18799,-0.1001,-0.40894,-0.49316,-0.33936,-0.1062,0.19531,0.5188,0.50781,0.29907,0.061035,-0.29785,-0.51514,-0.40527,-0.096436,0.12329,0.43213,0.61279,0.50049,0.19409,-0.041504,-0.30884,-0.51392,-0.30518,-0.036621,0.19409,0.49805,0.5127,0.31128,0.06958,-0.27466,-0.46387,-0.40894,-0.16602,0.093994,0.39917,0.59204,0.46387,0.21851,-0.039063,-0.3833,-0.50049,-0.31982,-0.058594,0.21484,0.43701,0.53955,0.34424,0,-0.22705,-0.41748,-0.52368,-0.15503,0.15137,0.40649,0.57617,0.54077,0.22949,-0.08667,-0.3418,-0.53711,-0.35767,-0.075684,0.21118,0.47241,0.55176,0.39551,0.11475,-0.19043,-0.49805,-0.50415,-0.25635,0.057373,0.32227,0.49072,0.43579,0.18677,-0.11475,-0.28931,-0.45776,-0.41138,-0.16113,0.15381,0.3894,0.46387,0.39551,0.014648,-0.3064,-0.48706,-0.53223,-0.33691,0.01709,0.29297,0.50659,0.44678,0.21729,-0.11475,-0.35034,-0.45654,-0.28931,-0.067139,0.20264,0.47607,0.49561,0.40527,0.015869,-0.16602,-0.48462,-0.44556,-0.30273,0,0.36987,0.55298,0.49561,0.29175,0.031738,-0.30396,-0.47729,-0.42236,-0.21851,0.091553,0.41504,0.44067,0.30762,0.12695,-0.19897,-0.44434,-0.42236,-0.21729,-0.041504,0.3186,0.50049,0.4895,0.25146,-0.018311,-0.31738,-0.49316,-0.36011,-0.12939,0.079346,0.39551,0.50537,0.3894,0.13306,-0.14526,-0.42358,-0.55786,-0.29053,-0.010986,0.26489,0.53223,0.50537,0.23193,0.0073242,-0.25635,-0.52734,-0.3833,-0.13916,0.13306,0.46631,0.53955,0.37354,0.12817,-0.15625,-0.41016,-0.49805,-0.30151,-0.054932,0.26123,0.50293,0.52246,0.28076,0.032959,-0.23926,-0.51392,-0.40527,-0.1709,0.1001,0.32837,0.51025,0.39429,0.065918,-0.24048,-0.35522,-0.51147,-0.28198,0.065918,0.29175,0.51392,0.49316,0.26367,-0.032959,-0.24048,-0.50537,-0.43091,-0.17334,0.10742,0.41504,0.51758,0.42725,0.12329,-0.177,-0.41138,-0.53223,-0.36133,0.018311,0.25879,0.51025,0.5835,0.33569,0.053711,-0.17456,-0.39307,-0.37598,-0.15137,0.19043,0.45898,0.56519,0.51758,0.22705,-0.15381,-0.33203,-0.43823,-0.36377,-0.062256,0.22461,0.4541,0.54199,0.41016,0.063477,-0.23682,-0.46631,-0.37231,-0.13794,0.14648,0.3894,0.56274,0.50415,0.18066,-0.14648,-0.40405,-0.54565,-0.3772,-0.054932,0.25391,0.4834,0.53101,0.39795,0.084229,-0.22705,-0.42603,-0.40405,-0.2417,0.080566,0.46021,0.5127,0.44312,0.25513,-0.058594,-0.34546,-0.4541,-0.38208,-0.11841,0.16113,0.4895,0.54199,0.38818,0.085449,-0.20508,-0.44312,-0.40283,-0.19165,0.0354,0.36621,0.51758,0.45654,0.20996,-0.11841,-0.40894,-0.52124,-0.36621,-0.083008,0.2124,0.53345,0.55664,0.4541,0.087891,-0.11719,-0.46753,-0.45654,-0.22949,0.05127,0.36133,0.54932,0.43457,0.22827,-0.014648,-0.28809,-0.5249,-0.36621,-0.13428,0.13794,0.47852,0.54077,0.33203,0.073242,-0.19653,-0.43823,-0.42114,-0.17456,-0.048828,0.3064,0.51514,0.50903,0.22339,-0.10132,-0.36865,-0.50903,-0.37476,-0.081787,0.084229,0.33936,0.50659,0.33936,0.043945,-0.21973,-0.45044,-0.53467,-0.24658,0.043945,0.32715,0.54565,0.50903,0.23682,-0.047607,-0.27466,-0.56641,-0.47729,-0.2002,0.089111,0.37231,0.5127,0.38452,0.053711,-0.1001,-0.33569,-0.47363,-0.27954,0.096436,0.33203,0.53467,0.54932,0.32959,-0.013428,-0.25879,-0.50171,-0.43213,-0.15625,0.14648,0.38696,0.49805,0.43091,0.13184,-0.13428,-0.36133,-0.46387,-0.2771,0.061035,0.35034,0.47485,0.50415,0.28931,-0.048828,-0.31982,-0.51514,-0.47241,-0.25269,0.092773,0.41992,0.45776,0.40161,0.22339,-0.12695,-0.32471,-0.41138,-0.30762,0.015869,0.35645,0.52124,0.53467,0.38818,0.070801,-0.25757,-0.52979,-0.37476,-0.27344,0.036621,0.3772,0.47363,0.42969,0.19165,-0.11475,-0.40771,-0.4895,-0.34546,-0.030518,0.25513,0.49561,0.5127,0.30273,0.0024414,-0.27954,-0.4895,-0.41748,-0.24902,0.056152,0.3772,0.49316,0.37598,0.13672,-0.1355,-0.39307,-0.45166,-0.34668,-0.12329,0.16479,0.5188,0.52856,0.3479,0.067139,-0.20386,-0.54688,-0.40039,-0.18799,0.079346,0.37354,0.46631,0.37842,0.12939,-0.12939,-0.38086,-0.52612,-0.30518,-0.021973,0.23193,0.46875,0.49805,0.32837,0.098877,-0.16113,-0.45898,-0.44678,-0.20264,0.043945,0.44434,0.5957,0.41504,0.16846,0.030518,-0.2832,-0.48706,-0.34668,-0.067139,0.17578,0.46265,0.5896,0.37231,0.03418,-0.23682,-0.4834,-0.45288,-0.12817,0.16479,0.34668,0.47607,0.4895,0.23071,-0.083008,-0.27832,-0.49316,-0.3772,-0.036621,0.24658,0.48706,0.61279,0.3894,0.059814,-0.092773,-0.4187,-0.49194,-0.22949,0.05127,0.33447,0.56519,0.52246,0.27466,-0.046387,-0.26367,-0.45654,-0.33691,-0.048828,0.23071,0.45898,0.53467,0.38696,0.041504,-0.21973,-0.43213,-0.49805,-0.38086,-0.1123,0.20386,0.35767,0.35156,0.16113,-0.21362,-0.44556,-0.54199,-0.49194,-0.23071,0.12817,0.37598,0.49438,0.46021,0.16846,-0.16846,-0.40649,-0.46631,-0.23315,0.083008,0.41504,0.55786,0.44312,0.21973,-0.1062,-0.32593,-0.48462,-0.40161,-0.15503,0.12695,0.43457,0.47729,0.37842,0.11597,-0.083008,-0.40649,-0.40771,-0.31006,-0.11597,0.25024,0.47607,0.44556,0.28442,-0.023193,-0.32593,-0.50049,-0.32715,-0.11475,0.16602,0.53223,0.6189,0.49072,0.24902,-0.081787,-0.37842,-0.44922,-0.29419,-0.029297,0.26733,0.49072,0.49561,0.28687,0.019531,-0.27588,-0.48828,-0.43213,-0.19165,0.10254,0.37842,0.51758,0.40527,0.17456,-0.087891,-0.38818,-0.50537,-0.2771,-0.041504,0.27588,0.52979,0.48462,0.22461,-0.015869,-0.28076,-0.52979,-0.45288,-0.19653,0.040283,0.3772,0.5188,0.40527,0.13916,-0.13794,-0.40161,-0.49683,-0.35645,-0.11353,0.17578,0.43335,0.50903,0.27588,-0.0061035,-0.31372,-0.46631,-0.38574,-0.11963,0.17456,0.41504,0.57617,0.41992,0.12207,-0.1355,-0.354,-0.5603,-0.35889,-0.032959,0.20508,0.44922,0.50293,0.29175,0.026855,-0.20996,-0.53589,-0.54199,-0.26367,0.06958,0.36011,0.58228,0.46387,0.20752,-0.061035,-0.27954,-0.40894,-0.29785,-0.061035,0.25879,0.52734,0.5603,0.40161,0.030518,-0.27832,-0.41748,-0.4248,-0.24902,0.065918,0.36987,0.47852,0.52979,0.26611,-0.091553,-0.35889,-0.50537,-0.39917,-0.089111,0.16113,0.45898,0.54321,0.43457,0.085449,-0.20874,-0.49561,-0.55908,-0.28076,0.040283,0.31006,0.47363,0.43213,0.19653,-0.067139,-0.3064,-0.47607,-0.37598,-0.10742,0.22461,0.56396,0.58716,0.35767,0.054932,-0.26367,-0.46997,-0.46143,-0.29053,-0.062256,0.27832,0.50659,0.44189,0.23926,0.019531,-0.31372,-0.44067,-0.2771,-0.037842,0.24658,0.51514,0.4834,0.40649,0.1062,-0.20386,-0.46509,-0.51392,-0.28076,0.0354,0.36377,0.53711,0.50659,0.30884,0.059814,-0.27344,-0.45654,-0.32227,-0.096436,0.13794,0.47974,0.54565,0.33203,0.095215,-0.15137,-0.448,-0.48462,-0.25391,-0.047607,0.31006,0.55664,0.48096,0.21973,0.03418,-0.23682,-0.44434,-0.34302,-0.12207,0.17822,0.47974,0.59326,0.5127,0.20264,-0.13306,-0.40039,-0.54565,-0.23926,0.053711,0.26611,0.45166,0.4895,0.23193,-0.015869,-0.25513,-0.55054,-0.41992,-0.074463,0.15503,0.38818,0.52979,0.39551,0.12329,-0.11963,-0.38086,-0.5481,-0.32715,-0.020752,0.24658,0.48096,0.50293,0.26733,-0.043945,-0.28198,-0.48462,-0.47241,-0.22583,0.097656,0.36499,0.5249,0.42725,0.092773,-0.20264,-0.4248,-0.56152,-0.27466,-0.057373,0.21606,0.448,0.49072,0.28809,-0.023193,-0.28809,-0.49683,-0.40283,-0.15381,0.19287,0.49316,0.48828,0.47852,0.25269,-0.1062,-0.3479,-0.48706,-0.38086,-0.021973,0.21973,0.46753,0.51636,0.36377,0.05249,-0.25146,-0.46631,-0.4187,-0.15869,0.15503,0.46387,0.53711,0.46753,0.23926,-0.070801,-0.30029,-0.46875,-0.37842,-0.092773,0.24414,0.52979,0.56885,0.31372,0.13428,-0.20874,-0.40283,-0.37598,-0.26367,0.083008,0.40283,0.53101,0.50537,0.25146,-0.12695,-0.36377,-0.48462,-0.33081,-0.10254,0.17212,0.40039,0.4834,0.3186,0.15381,-0.177,-0.44189,-0.36133,-0.11475,0.1416,0.50293,0.53711,0.43335,0.23193,-0.029297,-0.34302,-0.53711,-0.42725,-0.1709,0.11963,0.52246,0.4834,0.28564,0.11597,-0.16235,-0.4541,-0.4541,-0.18066,0.11841,0.45532,0.53955,0.44189,0.22461,-0.078125,-0.33081,-0.48828,-0.354,-0.11108,0.14404,0.45776,0.55054,0.4248,0.13428,-0.1123,-0.42847,-0.46631,-0.2002,0.05127,0.30273,0.57373,0.56641,0.28687,0.0061035,-0.22461,-0.45044,-0.31372,-0.0097656,0.21606,0.47852,0.54443,0.37354,0.087891,-0.17334,-0.45532,-0.49438,-0.22461,-0.042725,0.31738,0.49316,0.45898,0.2478,-0.043945,-0.31372,-0.50659,-0.39673,-0.075684,0.12207,0.36621,0.53467,0.43945,0.091553,-0.18311,-0.39063,-0.50659,-0.27344,0.0354,0.2832,0.4834,0.5188,0.28687,-0.075684,-0.31494,-0.4541,-0.41748,-0.13428,0.18066,0.44678,0.45532,0.37109,0.083008,-0.18433,-0.42969,-0.46997,-0.29785,0.013428,0.30518,0.57739,0.59814,0.38574,0.046387,-0.25513,-0.44434,-0.35278,-0.10864,0.17456,0.44556,0.52246,0.4541,0.16724,-0.1416,-0.38208,-0.44678,-0.32837,-0.041504,0.26855,0.44067,0.43335,0.32104,0.048828,-0.26611,-0.47119,-0.43945,-0.28076,0.050049,0.45654,0.46753,0.33691,0.18311,-0.1062,-0.36377,-0.4834,-0.23804,0.046387,0.23315,0.5127,0.50781,0.27832,-0.0024414,-0.25513,-0.48706,-0.32104,-0.11963,0.10254,0.46631,0.55298,0.46387,0.26978,-0.05127,-0.37964,-0.47241,-0.27588,-0.026855,0.27588,0.57617,0.50537,0.30273,0.063477,-0.25146,-0.50903,-0.43457,-0.17944,0.10254,0.3894,0.53345,0.39795,0.11475,-0.15991,-0.29785,-0.50903,-0.32715,-0.05127,0.19409,0.51514,0.57617,0.35156,0.1123,-0.13672,-0.46265,-0.42969,-0.18433,0.10376,0.39917,0.52246,0.41748,0.1416,-0.1355,-0.37598,-0.54932,-0.29297,-0.043945,0.17944,0.54321,0.5127,0.41138,0.096436,-0.16846,-0.46387,-0.4834,-0.16968,0.092773,0.36865,0.50903,0.48706,0.271,-0.040283,-0.31738,-0.49316,-0.37109,-0.05127,0.26489,0.45654,0.49561,0.39551,0.010986,-0.1416,-0.34058,-0.46265,-0.17456,0.15625,0.3894,0.52979,0.51758,0.26001,-0.030518,-0.25391,-0.49316,-0.37231,-0.043945,0.21973,0.51392,0.60791,0.3772,0.073242,-0.19653,-0.40405,-0.43213,-0.24902,0.015869,0.33813,0.53711,0.50781,0.2417,-0.12329,-0.41748,-0.52612,-0.41748,-0.19775,0.15259,0.44189,0.47363,0.40894,0.11963,-0.21484,-0.39429,-0.43457,-0.24292,0.021973,0.33447,0.49438,0.48096,0.28687,-0.026855,-0.31372,-0.53467,-0.43823,-0.2002,0.10864,0.38696,0.49927,0.36133,0.12451,-0.18799,-0.41992,-0.46997,-0.29907,-0.03418,0.34546,0.53223,0.46387,0.23682,-0.06958,-0.36377,-0.47852,-0.37842,-0.19287,0.065918,0.40894,0.49316,0.36377,0.11719,-0.18066,-0.4541,-0.50293,-0.28809,-0.021973,0.26611,0.52979,0.55542,0.37109,0.087891,-0.21606,-0.52979,-0.42236,-0.14893,0.13062,0.37231,0.49316,0.37231,0.17456,-0.11963,-0.41504,-0.55054,-0.34058,-0.058594,0.17822,0.50537,0.48584,0.2417,0.010986,-0.26123,-0.57739,-0.42358,-0.2063,0.064697,0.36621,0.54077,0.43335,0.16602,-0.079346,-0.3186,-0.44434,-0.2832,0.012207,0.26001,0.52612,0.57129,0.31982,-0.026855,-0.26855,-0.51025,-0.45898,-0.18799,0.11719,0.44922,0.57129,0.46753,0.16602,-0.091553,-0.34424,-0.48218,-0.34302,-0.0024414,0.21484,0.41626,0.49316,0.34668,0.074463,-0.18188,-0.46631,-0.48706,-0.16602,0.11475,0.39673,0.52734,0.50659,0.25879,-0.031738,-0.35889,-0.49561,-0.36865,-0.046387,0.24048,0.48584,0.51025,0.34424,-0.0024414,-0.29053,-0.50781,-0.49072,-0.26123,0.054932,0.36987,0.53833,0.48584,0.27344,-0.048828,-0.28809,-0.39551,-0.31372,-0.059814,0.25146,0.48096,0.5542,0.42847,0.084229,-0.19897,-0.43701,-0.45166,-0.20264,0.078125,0.42603,0.5249,0.4834,0.28198,-0.046387,-0.33936,-0.48096,-0.35522,-0.11719,0.23193,0.49561,0.51147,0.36743,0.12817,-0.18066,-0.43701,-0.44678,-0.23315,0.012207,0.3418,0.52368,0.43091,0.23926,-0.043945,-0.31006,-0.48828,-0.38818,-0.15503,0.16113,0.44434,0.53711,0.4248,0.080566,-0.1355,-0.4126,-0.44312,-0.21729,0.040283,0.32227,0.54321,0.49561,0.28564,-0.015869,-0.32715,-0.51025,-0.32593,-0.10742,0.10742,0.37354,0.47852,0.30762,0.065918,-0.19531,-0.51514,-0.61035,-0.31372,-0.030518,0.2832,0.52002,0.4834,0.24902,-0.0061035,-0.24658,-0.48584,-0.38208,-0.11353,0.15381,0.46021,0.57373,0.3894,0.10254,-0.15869,-0.42725,-0.44067,-0.24414,-0.028076,0.26978,0.48584,0.46875,0.27832,-0.019531,-0.27832,-0.51514,-0.42969,-0.1355,0.15869,0.45166,0.57617,0.53467,0.11841,-0.13916,-0.39551,-0.56152,-0.35034,0.012207,0.24414,0.46753,0.47729,0.26489,0.0061035,-0.26001,-0.46875,-0.38208,-0.11963,0.16113,0.47607,0.54443,0.46021,0.16602,-0.097656,-0.36987,-0.4834,-0.40283,-0.12329,0.20264,0.42847,0.44556,0.23682,0.020752,-0.28076,-0.4541,-0.44434,-0.18311,0.057373,0.30762,0.48828,0.45654,0.177,-0.13062,-0.34668,-0.49683,-0.29785,-0.014648,0.26367,0.47607,0.5127,0.29175,0.058594,-0.271,-0.46875,-0.40771,-0.19897,0.10132,0.44189,0.49805,0.47363,0.2771,-0.087891,-0.35034,-0.48462,-0.354,-0.068359,0.24414,0.4895,0.53467,0.33203,0.059814,-0.23804,-0.46265,-0.41382,-0.17822,0.093994,0.39917,0.54199,0.44556,0.21973,-0.091553,-0.32349,-0.47241,-0.26367,-0.05127,0.20752,0.51514,0.52002,0.3772,0.13428,-0.21484,-0.50537,-0.4187,-0.22339,0.096436,0.40283,0.57861,0.49316,0.29419,0.0073242,-0.27832,-0.5249,-0.36621,-0.063477,0.18799,0.4541,0.50903,0.32104,0.015869,-0.20996,-0.4834,-0.50781,-0.2417,0.021973,0.32349,0.51025,0.44067,0.18311,0.041504,-0.29785,-0.51025,-0.34058,-0.081787,0.19409,0.49561,0.55054,0.37598,0.10742,-0.15259,-0.44189,-0.46631,-0.16357,0.10742,0.36377,0.53589,0.51025,0.23926,-0.040283,-0.24292,-0.45654,-0.38086,-0.059814,0.15259,0.4248,0.52612,0.4248,0.073242,-0.20386,-0.45166,-0.52979,-0.29541,0.019531,0.31982,0.52979,0.53223,0.30273,0.0048828,-0.25757,-0.42847,-0.32837,-0.058594,0.21973,0.49438,0.57373,0.43579,0.10498,-0.16113,-0.3772,-0.46753,-0.26367,0.05127,0.37476,0.448,0.5249,0.31128,-0.05249,-0.29785,-0.45166,-0.3418,-0.16113,0.11108,0.37231,0.48462,0.40649,0.1123,-0.23682,-0.47852,-0.5249,-0.30029,0.0061035,0.30273,0.49316,0.49072,0.30151,0.043945,-0.24536,-0.4248,-0.35889,-0.10864,0.19043,0.51758,0.56885,0.45776,0.20508,-0.10864,-0.33447,-0.43335,-0.33081,-0.058594,0.2832,0.53223,0.54077,0.33691,0.014648,-0.2832,-0.44312,-0.33813,-0.10986,0.15381,0.45166,0.55176,0.40283,0.19775,-0.12695,-0.40771,-0.49683,-0.29663,-0.041504,0.24414,0.50049,0.5127,0.31128,0.043945,-0.29541,-0.53467,-0.45654,-0.22461,0.065918,0.33691,0.4895,0.38086,0.13672,-0.16235,-0.41138,-0.53223,-0.38208,-0.068359,0.22705,0.48828,0.4895,0.27222,0.0073242,-0.20752,-0.46631,-0.43091,-0.19897,0.046387,0.42114,0.59692,0.4541,0.15869,-0.1062,-0.39917,-0.54932,-0.38696,-0.085449,0.13916,0.42358,0.52979,0.31128,0.01709,-0.23193,-0.49194,-0.46509,-0.177,0.10864,0.36621,0.49561,0.45166,0.18555,-0.11108,-0.354,-0.49927,-0.44189,-0.1123,0.17822,0.44434,0.53223,0.35645,0.0354,-0.19653,-0.47852,-0.49316,-0.19531,0.096436,0.3418,0.59082,0.52246,0.24292,-0.048828,-0.3064,-0.48706,-0.33936,-0.023193,0.17334,0.51025,0.49805,0.35034,0.0097656,-0.18799,-0.44312,-0.4126,-0.22461,-0.013428,0.34546,0.45654,0.44556,0.22949,-0.023193,-0.33325,-0.47363,-0.38574,-0.080566,0.23682,0.47852,0.46631,0.3894,0.063477,-0.23193,-0.41504,-0.49072,-0.19531,0.10498,0.4187,0.50049,0.47852,0.29175,-0.046387,-0.31494,-0.47363,-0.44922,-0.22461,0.084229,0.46997,0.50293,0.42725,0.16479,-0.14404,-0.41504,-0.46265,-0.30762,-0.058594,0.31738,0.56152,0.50171,0.24902,-0.058594,-0.31494,-0.47974,-0.34668,-0.1709,0.19043,0.448,0.53101,0.40039,0.12329,-0.18433,-0.41626,-0.44556,-0.21729,0.050049,0.34668,0.58838,0.46021,0.29419,0.015869,-0.25513,-0.54077,-0.43701,-0.19897,0.096436,0.39429,0.49316,0.33691,0.12207,-0.16357,-0.44067,-0.51025,-0.29785,0.0048828,0.40039,0.53711,0.45288,0.20264,-0.032959,-0.21606,-0.46387,-0.43457,-0.2356,0.014648,0.3833,0.53711,0.43945,0.12085,-0.13916,-0.37598,-0.47119,-0.31006,-0.050049,0.19043,0.50659,0.5603,0.32471,-0.0097656,-0.32837,-0.55908,-0.42236,-0.12451,0.1123,0.35889,0.4187,0.36133,0.12329,-0.13428,-0.38696,-0.53589,-0.38086,-0.046387,0.2002,0.52246,0.57495,0.35522,0.056152,-0.18311,-0.47852,-0.51636,-0.27588,0.050049,0.33203,0.53223,0.43579,0.14038,-0.16113,-0.36377,-0.49438,-0.3186,-0.043945,0.27954,0.52124,0.52246,0.33447,0.029297,-0.23682,-0.43823,-0.37476,-0.17944,0.095215,0.42847,0.448,0.44678,0.22095,-0.10254,-0.33691,-0.49194,-0.36987,-0.076904,0.2002,0.38818,0.5188,0.36621,0.092773,-0.2417,-0.49683,-0.46753,-0.18433,0.14648,0.4541,0.53101,0.37109,0.15503,-0.14038,-0.35889,-0.4541,-0.35278,-0.1062,0.1709,0.47852,0.50659,0.35156,0.078125,-0.19531,-0.45288,-0.43945,-0.25879,0.029297,0.35645,0.55786,0.47729,0.24536,-0.075684,-0.31006,-0.44922,-0.29053,-0.056152,0.2832,0.54932,0.49805,0.39551,0.076904,-0.22827,-0.4541,-0.42114,-0.19287,-0.0073242,0.36255,0.51147,0.43457,0.25513,0.0048828,-0.31494,-0.55176,-0.32715,-0.043945,0.21851,0.47363,0.5957,0.43945,0.15747,-0.08667,-0.41748,-0.46997,-0.17334,0.10864,0.39673,0.50293,0.40161,0.17456,-0.10132,-0.33203,-0.53345,-0.39673,-0.15991,0.10986,0.46143,0.54443,0.4248,0.13184,-0.10742,-0.36011,-0.46997,-0.2771,-0.031738,0.25879,0.53955,0.54932,0.27588,-0.045166,-0.27588,-0.47852,-0.39185,-0.070801,0.19409,0.45288,0.58105,0.49805,0.15381,-0.12451,-0.39673,-0.46753,-0.26611,0.059814,0.31128,0.47363,0.47363,0.29785,0.0024414,-0.29419,-0.5127,-0.48096,-0.2002,0.090332,0.33813,0.5481,0.4248,0.1123,-0.15747,-0.35645,-0.50171,-0.22949,0.073242,0.32959,0.56641,0.58105,0.3064,-0.021973,-0.29175,-0.43823,-0.354,-0.15625,0.15625,0.45898,0.54932,0.52979,0.24902,-0.11475,-0.33081,-0.42358,-0.30029,0.020752,0.31738,0.44434,0.46509,0.28564,-0.01709,-0.31006,-0.54199,-0.46387,-0.21484,0.12695,0.41138,0.51514,0.43945,0.18799,-0.11475,-0.32227,-0.49438,-0.33936,-0.056152,0.2356,0.47363,0.44189,0.35767,0.10986,-0.2478,-0.4895,-0.42236,-0.26245,0.074463,0.3772,0.49805,0.39185,0.17456,-0.14893,-0.42358,-0.48828,-0.3186,-0.090332,0.22461,0.49805,0.54565,0.34546,0.074463,-0.26367,-0.52368,-0.47485,-0.25391,0.040283,0.40771,0.51636,0.48462,0.25879,-0.029297,-0.31006,-0.4895,-0.32104,-0.073242,0.19775,0.46387,0.49927,0.32227,0.074463,-0.25146,-0.54443,-0.50903,-0.28076,0.031738,0.43335,0.52368,0.38696,0.24902,0.0097656,-0.26855,-0.45288,-0.26733,-0.018311,0.23926,0.57739,0.52368,0.33936,0.0354,-0.2417,-0.49316,-0.47119,-0.18799,0.070801,0.34424,0.54932,0.51025,0.23071,-0.073242,-0.3125,-0.53955,-0.35889,-0.063477,0.1709,0.44067,0.56641,0.38574,0.1001,-0.17456,-0.49072,-0.53955,-0.21973,0.081787,0.41504,0.48584,0.46631,0.21606,-0.043945,-0.29053,-0.49194,-0.4187,-0.11963,0.15503,0.42114,0.55664,0.40405,0.054932,-0.20508,-0.43091,-0.50293,-0.26489,0.023193,0.32227,0.51514,0.50415,0.26611,-0.065918,-0.29785,-0.46997,-0.38452,-0.12939,0.13672,0.52368,0.56519,0.49561,0.18799,-0.16357,-0.41504,-0.52002,-0.29175,0.019531,0.28564,0.50537,0.5542,0.3418,0.030518,-0.23193,-0.48706,-0.34424,-0.13184,0.18311,0.42236,0.47852,0.35645,0.10254,-0.18921,-0.39795,-0.50659,-0.40161,-0.10498,0.26001,0.47607,0.46631,0.26855,-0.041504,-0.31006,-0.49316,-0.36743,-0.19653,0.087891,0.42603,0.54199,0.42847,0.15747,-0.19775,-0.43701,-0.48462,-0.30151,0.014648,0.25513,0.46021,0.4834,0.29419,0.01709,-0.2478,-0.47729,-0.40039,-0.16357,0.15625,0.51025,0.56519,0.45898,0.24658,-0.028076,-0.34424,-0.47974,-0.35645,-0.083008,0.23193,0.5127,0.5127,0.24902,0.014648,-0.26978,-0.48096,-0.38208,-0.15747,0.096436,0.41382,0.55176,0.44067,0.15869,-0.15137,-0.36743,-0.48462,-0.35156,-0.064697,0.15381,0.43091,0.49561,0.31738,0.046387,-0.26123,-0.50903,-0.41504,-0.18799,0.065918,0.3479,0.53711,0.44189,0.18799,-0.092773,-0.32471,-0.54077,-0.32349,-0.021973,0.21606,0.46509,0.51514,0.29907,0.010986,-0.23315,-0.48462,-0.50049,-0.2478,0.053711,0.37231,0.5127,0.44556,0.19165,-0.079346,-0.2832,-0.48584,-0.40039,-0.1123,0.1355,0.40283,0.53467,0.32227,-0.010986,-0.27588,-0.47119,-0.39551,-0.1416,0.11841,0.3894,0.57495,0.54443,0.31982,-0.029297,-0.30273,-0.46875,-0.34058,-0.036621,0.2417,0.42969,0.51514,0.44434,0.10376,-0.17456,-0.41748,-0.49805,-0.26123,0,0.30151,0.49561,0.43945,0.2417,-0.068359,-0.2832,-0.43823,-0.37598,-0.15137,0.16235,0.44556,0.50049,0.35156,0.025635,-0.24048,-0.42236,-0.46387,-0.24414,0.012207,0.33691,0.50781,0.49438,0.29419,-0.036621,-0.32471,-0.47852,-0.42114,-0.17334,0.12207,0.38208,0.4834,0.36987,0.12329,-0.19287,-0.45288,-0.46631,-0.25879,-0.0085449,0.32959,0.52368,0.47974,0.26611,0.061035,-0.27588,-0.5249,-0.36621,-0.087891,0.093994,0.39795,0.448,0.33936,0.097656,-0.16113,-0.41382,-0.49683,-0.31372,-0.064697,0.24536,0.47119,0.43701,0.23926,-0.021973,-0.30029,-0.41382,-0.35645,-0.12939,0.14771,0.47852,0.57861,0.40771,0.096436,-0.19531,-0.44189,-0.53223,-0.32104,-0.028076,0.26001,0.52246,0.5542,0.31128,0.043945,-0.23682,-0.47852,-0.36011,-0.090332,0.15747,0.3418,0.55176,0.34424,0.11841,-0.14404,-0.40161,-0.56396,-0.31738,-0.032959,0.25879,0.50171,0.52734,0.28809,0.0048828,-0.24292,-0.47729,-0.41992,-0.22705,0.040283,0.45166,0.50049,0.38086,0.076904,-0.12939,-0.4126,-0.50659,-0.35889,-0.059814,0.18921,0.43457,0.53223,0.30029,-0.0097656,-0.22339,-0.43579,-0.42358,-0.18677,0.11963,0.32837,0.49805,0.43945,0.16479,-0.12939,-0.36987,-0.50171,-0.40039,-0.047607,0.19897,0.47607,0.53101,0.38574,0.020752,-0.24414,-0.41382,-0.39307,-0.16846,0.16357,0.39307,0.53467,0.39917,0.24414,-0.076904,-0.32959,-0.4895,-0.31982,-0.01709,0.25269,0.47607,0.51514,0.39795,0.081787,-0.21973,-0.4248,-0.43701,-0.2356,0.046387,0.41016,0.50049,0.44189,0.22217,-0.13428,-0.4248,-0.5188,-0.4187,-0.1355,0.19165,0.44678,0.48828,0.354,0.070801,-0.23804,-0.42358,-0.41504,-0.20386,0.090332,0.44189,0.5603,0.51514,0.19775,-0.026855,-0.30151,-0.47485,-0.38574,-0.12817,0.2002,0.50537,0.54199,0.4126,0.14648,-0.16846,-0.49194,-0.48584,-0.26733,-0.073242,0.22705,0.44922,0.31494,0.091553,-0.17456,-0.43823,-0.59082,-0.43091,-0.16724,0.13794,0.43945,0.55176,0.42236,0.073242,-0.17944,-0.44434,-0.46875,-0.23193,-0.010986,0.354,0.57251,0.52124,0.28076,-0.0097656,-0.31738,-0.50049,-0.39063,-0.14648,0.12695,0.41016,0.58594,0.40161,0.13184,-0.13916,-0.39917,-0.4541,-0.21973,0.0073242,0.28809,0.4895,0.48462,0.2417,-0.05127,-0.31006,-0.5542,-0.45654,-0.22461,0.076904,0.3833,0.50537,0.40527,0.10742,-0.16357,-0.38574,-0.53467,-0.31982,0.021973,0.28809,0.58716,0.55664,0.32471,-0.029297,-0.26001,-0.48462,-0.35278,-0.11841,0.10376,0.35889,0.53223,0.44189,0.15137,-0.1123,-0.354,-0.45166,-0.28076,0.021973,0.25269,0.49438,0.54077,0.33936,0.036621,-0.25146,-0.49194,-0.47607,-0.26611,0.092773,0.36987,0.50049,0.42603,0.12451,-0.177,-0.38818,-0.45654,-0.31738,-0.0048828,0.29053,0.50049,0.5249,0.30762,-0.010986,-0.2417,-0.45776,-0.4834,-0.22705,0.037842,0.41992,0.52979,0.50293,0.23071,-0.065918,-0.36865,-0.48828,-0.35278,-0.037842,0.27344,0.47363,0.49316,0.34424,0.067139,-0.27832,-0.46875,-0.42847,-0.2356,0.065918,0.39917,0.48584,0.39551,0.19043,-0.091553,-0.32837,-0.42114,-0.30029,-0.043945,0.271,0.53345,0.49194,0.41016,0.1062,-0.21851,-0.47852,-0.50293,-0.25391,0.010986,0.30151,0.49561,0.43213,0.19409,-0.10254,-0.33203,-0.49927,-0.2832,-0.0354,0.16113,0.54199,0.55908,0.36255,0.083008,-0.22583,-0.5127,-0.52856,-0.26001,0.0024414,0.36987,0.54443,0.47729,0.2832,0.05127,-0.25513,-0.50415,-0.37476,-0.10986,0.19531,0.47607,0.55176,0.4126,0.092773,-0.16479,-0.4248,-0.46143,-0.2002,0.075684,0.35156,0.52612,0.46631,0.23438,-0.041504,-0.26367,-0.44189,-0.31616,-0.075684,0.19287,0.48462,0.57617,0.44434,0.097656,-0.16357,-0.43335,-0.51758,-0.2832,-0.029297,0.22217,0.54565,0.54565,0.29663,-0.043945,-0.29297,-0.46753,-0.36621,-0.046387,0.22705,0.4541,0.46631,0.37231,0.050049,-0.20508,-0.36011,-0.46387,-0.29785,0.0061035,0.23926,0.44434,0.42725,0.24048,0.037842,-0.26123,-0.49805,-0.354,-0.10986,0.19653,0.48218,0.50659,0.4248,0.11963,-0.17212,-0.40283,-0.50293,-0.31372,0.0354,0.32715,0.50049,0.44922,0.24292,-0.050049,-0.28442,-0.42847,-0.39185,-0.18921,0.11475,0.45288,0.50537,0.4541,0.17456,-0.177,-0.44678,-0.49316,-0.27344,-0.085449,0.20752,0.44922,0.43701,0.24536,-0.0085449,-0.33325,-0.53223,-0.31494,-0.083008,0.19775,0.5127,0.53345,0.46631,0.23193,-0.089111,-0.29541,-0.42969,-0.39551,-0.065918,0.25635,0.48584,0.49927,0.29297,0.029297,-0.28564,-0.50537,-0.4248,-0.14771,0.12695,0.4187,0.57251,0.43945,0.20996,-0.11841,-0.4187,-0.51636,-0.28076,-0.073242,0.177,0.43213,0.47363,0.26855,0.045166,-0.2356,-0.56152,-0.46265,-0.22949,0.045166,0.39063,0.55664,0.47729,0.26123,-0.023193,-0.28198,-0.44678,-0.27588,-0.014648,0.23438,0.53711,0.53833,0.30151,0.03418,-0.24292,-0.53467,-0.47241,-0.23926,0.072021,0.354,0.53467,0.40405,0.11841,-0.11719,-0.29663,-0.5188,-0.41382,-0.092773,0.12085,0.39429,0.4834,0.33325,0.0073242,-0.26367,-0.53467,-0.54932,-0.25879,0.046387,0.29663,0.51392,0.50781,0.25635,-0.03418,-0.28687,-0.48828,-0.30273,0.0024414,0.22949,0.47363,0.49805,0.37354,0.076904,-0.18555,-0.39307,-0.43457,-0.2002,0.13916,0.40039,0.52856,0.55298,0.27588,-0.037842,-0.23193,-0.43213,-0.41992,-0.12939,0.15137,0.40161,0.51514,0.36743,0.0097656,-0.2832,-0.46265,-0.49072,-0.26367,0.014648,0.31982,0.50659,0.46875,0.26001,-0.074463,-0.31372,-0.46021,-0.39307,-0.14404,0.12451,0.43457,0.48706,0.38574,0.096436,-0.21729,-0.44312,-0.47607,-0.28076,0.026855,0.35645,0.53955,0.51758,0.33691,-0.014648,-0.29053,-0.42969,-0.36865,-0.12207,0.21362,0.45532,0.46753,0.38086,0.097656,-0.23926,-0.44434,-0.55176,-0.32959,-0.063477,0.29175,0.49805,0.45288,0.25146,-0.0024414,-0.28442,-0.50415,-0.38696,-0.16602,0.081787,0.39063,0.52979,0.40039,0.11963,-0.17822,-0.44189,-0.51147,-0.28564,-0.031738,0.27222,0.49561,0.49316,0.29053,-0.031738,-0.35889,-0.47852,-0.31738,-0.11719,0.11963,0.43457,0.50659,0.37231,0.16846,-0.12939,-0.44067,-0.57495,-0.32471,0.014648,0.24658,0.54688,0.57129,0.33936,0.098877,-0.14526,-0.50293,-0.37842,-0.17578,0.1416,0.44189,0.5542,0.40039,0.10254,-0.092773,-0.31372,-0.46753,-0.3418,-0.0036621,0.25391,0.55542,0.60181,0.29541,0.10742,-0.16846,-0.47852,-0.41016,-0.16602,0.039063,0.27832,0.52002,0.46753,0.16357,-0.12939,-0.40039,-0.56152,-0.35034,-0.01709,0.21973,0.43457,0.4834,0.31006,0.010986,-0.25269,-0.46143,-0.48706,-0.21729,0.093994,0.44189,0.4834,0.40283,0.24658,-0.043945,-0.27832,-0.44434,-0.38574,-0.065918,0.24902,0.5188,0.53711,0.34912,0.037842,-0.22461,-0.43091,-0.43457,-0.22339,0.087891,0.35034,0.49927,0.46509,0.2002,-0.1355,-0.3894,-0.50293,-0.39185,-0.13062,0.17944,0.40649,0.4541,0.35645,0.053711,-0.2063,-0.4126,-0.35645,-0.18311,0.12451,0.41504,0.56519,0.46387,0.25024,0.0024414,-0.28809,-0.4834,-0.30518,-0.042725,0.17456,0.54565,0.49438,0.33203,0.019531,-0.15503,-0.48828,-0.4126,-0.27344,-0.0073242,0.37598,0.52246,0.43823,0.2356,-0.053711,-0.33325,-0.4541,-0.33447,-0.090332,0.16357,0.44434,0.43701,0.29907,0.18677,-0.12207,-0.47607,-0.44556,-0.15625,0.041504,0.42236,0.61035,0.39795,0.28931,0.036621,-0.31738,-0.5127,-0.37354,-0.10742,0.15503,0.46265,0.52856,0.40649,0.10742,-0.15991,-0.43213,-0.50049,-0.26123,-0.012207,0.28076,0.48584,0.49072,0.30029,0,-0.26733,-0.50537,-0.3772,-0.095215,0.1416,0.41016,0.54565,0.38574,0.1001,-0.11719,-0.39917,-0.54321,-0.24902,0.042725,0.29175,0.56274,0.5542,0.25879,-0.0097656,-0.22461,-0.47729,-0.42114,-0.16724,0.064697,0.3125,0.47607,0.37354,0.085449,-0.18066,-0.39795,-0.45776,-0.26123,0.043945,0.33447,0.55664,0.54199,0.31738,-0.010986,-0.31616,-0.52246,-0.39795,-0.095215,0.10254,0.47852,0.52979,0.49194,0.20874,-0.083008,-0.31006,-0.42603,-0.31738,0.046387,0.29785,0.53589,0.53955,0.30518,0.062256,-0.18555,-0.40283,-0.37109,-0.13184,0.16602,0.44189,0.49438,0.44434,0.19409,-0.15259,-0.37109,-0.45776,-0.30273,-0.050049,0.26489,0.51514,0.47119,0.3186,0.045166,-0.2771,-0.46021,-0.40894,-0.22583,0.067139,0.35767,0.51758,0.43091,0.20386,-0.1001,-0.31372,-0.42725,-0.30396,-0.026855,0.28931,0.49438,0.57007,0.40527,0.097656,-0.2124,-0.44312,-0.39917,-0.18921,0.084229,0.41748,0.53223,0.4248,0.21729,-0.085449,-0.42358,-0.47974,-0.26611,-0.079346,0.19653,0.48584,0.49927,0.31494,0.080566,-0.2002,-0.46387,-0.43945,-0.18066,0.085449,0.40894,0.4834,0.38574,0.16479,-0.11475,-0.354,-0.5127,-0.39795,-0.12207,0.18311,0.4834,0.5127,0.38818,0.06958,-0.2124,-0.45776,-0.448,-0.18311,0.043945,0.29053,0.54199,0.47363,0.20996,-0.023193,-0.29175,-0.49805,-0.31006,0.0012207,0.22461,0.50537,0.55542,0.37598,0.0354,-0.1001,-0.38452,-0.50903,-0.17334,0.097656,0.36621,0.52612,0.49316,0.271,0.0012207,-0.29053,-0.48584,-0.41626,-0.15869,0.14893,0.38696,0.53467,0.42603,0.11841,-0.14526,-0.38208,-0.45898,-0.20386,0.064697,0.32715,0.54565,0.57373,0.33447,-0.024414,-0.24048,-0.43701,-0.35889,-0.068359,0.22461,0.47119,0.56396,0.53101,0.19287,-0.10986,-0.31738,-0.45898,-0.30029,0.040283,0.32349,0.47607,0.4541,0.23926,-0.083008,-0.32593,-0.51392,-0.42114,-0.18311,0.16113,0.41504,0.48096,0.38818,0.093994,-0.22949,-0.3772,-0.4541,-0.30151,-0.0024414,0.3125,0.51758,0.5188,0.34058,0.014648,-0.31372,-0.5127,-0.40039,-0.2356,0.1123,0.36865,0.50049,0.448,0.20752,-0.14893,-0.36011,-0.42358,-0.27588,0.019531,0.30762,0.54199,0.50415,0.29419,0.0354,-0.2771,-0.4834,-0.38696,-0.21729,0.090332,0.46631,0.55298,0.42847,0.21606,-0.080566,-0.36987,-0.4541,-0.30762,-0.063477,0.22339,0.50659,0.48828,0.29907,0.0061035,-0.29663,-0.5249,-0.40283,-0.20508,0.11475,0.42847,0.52856,0.46631,0.23315,-0.081787,-0.36255,-0.48828,-0.31128,-0.040283,0.20386,0.49438,0.52246,0.29907,0.053711,-0.23438,-0.51514,-0.49316,-0.23071,0.032959,0.31494,0.53711,0.44922,0.12939,-0.11719,-0.33447,-0.52246,-0.25635,-0.063477,0.16357,0.53955,0.51025,0.27344,0.10986,-0.19775,-0.53711,-0.44189,-0.14893,-0.0024414,0.35645,0.50659,0.45166,0.18555,-0.1062,-0.33936,-0.5249,-0.36499,-0.073242,0.18799,0.44312,0.54321,0.35156,0.042725,-0.22217,-0.448,-0.51025,-0.25879,0.03418,0.32715,0.48462,0.46631,0.22217,-0.092773,-0.30273,-0.48218,-0.38696,-0.092773,0.20264,0.43213,0.50293,0.41748,0.063477,-0.21851,-0.46143,-0.45898,-0.19653,0,0.36743,0.4834,0.43945,0.23438,-0.10986,-0.33203,-0.45166,-0.27588,-0.0036621,0.2002,0.52246,0.47974,0.33081,0.11719,-0.19287,-0.45288,-0.50049,-0.37354,-0.058594,0.33569,0.45898,0.45654,0.29541,-0.065918,-0.28564,-0.43945,-0.34912,-0.12817,0.2002,0.42847,0.5542,0.44434,0.15381,-0.16602,-0.45654,-0.49927,-0.2478,0.015869,0.29419,0.50293,0.42114,0.23193,-0.041504,-0.30029,-0.4834,-0.32959,-0.20264,0.12451,0.46997,0.49805,0.39307,0.15137,-0.18188,-0.44922,-0.40039,-0.28809,-0.025635,0.20264,0.47363,0.45044,0.23438,-0.068359,-0.31494,-0.50537,-0.39185,-0.16113,0.091553,0.41138,0.56152,0.40649,0.12207,-0.18677,-0.4248,-0.5127,-0.3186,-0.0061035,0.21606,0.44556,0.45532,0.27222,0.020752,-0.27832,-0.52124,-0.3894,-0.092773,0.048828,0.42847,0.57373,0.43457,0.19775,-0.010986,-0.33691,-0.50537,-0.25391,-0.057373,0.24048,0.5249,0.52979,0.24414,-0.014648,-0.24414,-0.45776,-0.41748,-0.1709,0.073242,0.42847,0.5542,0.47607,0.177,-0.12695,-0.35278,-0.50049,-0.29419,0.012207,0.2063,0.44434,0.57373,0.36377,0.065918,-0.23438,-0.49072,-0.448,-0.12695,0.15381,0.37598,0.54443,0.46875,0.19653,-0.084229,-0.30762,-0.49561,-0.32471,-0.01709,0.12817,0.46753,0.44922,0.29419,-0.03418,-0.2478,-0.40283,-0.4541,-0.19653,0.15747,0.39307,0.57007,0.53101,0.26367,-0.061035,-0.23315,-0.44189,-0.34058,-0.048828,0.26001,0.49927,0.57861,0.32227,0.11108,-0.20752,-0.40771,-0.41992,-0.22949,0.10498,0.41382,0.5249,0.4834,0.26123,-0.092773,-0.3479,-0.47852,-0.37598,-0.12817,0.16479,0.40894,0.48828,0.40527,0.093994,-0.2002,-0.42969,-0.41748,-0.18066,0.13428,0.43457,0.46509,0.41382,0.25879,-0.05127,-0.32715,-0.48096,-0.3833,-0.13428,0.19653,0.50415,0.47485,0.36987,0.1001,-0.18311,-0.43945,-0.46387,-0.29053,-0.0085449,0.33325,0.53955,0.46265,0.23193,-0.065918,-0.31738,-0.47852,-0.35278,-0.15503,0.098877,0.40649,0.46387,0.32471,0.11719,-0.1709,-0.47852,-0.50537,-0.25757,0.0061035,0.32715,0.56274,0.5249,0.354,0.084229,-0.29785,-0.50293,-0.32959,-0.037842,0.090332,0.46753,0.48828,0.34546,0.097656,-0.13184,-0.43335,-0.49927,-0.25635,0.020752,0.30762,0.5249,0.52612,0.26123,-0.012207,-0.22217,-0.43457,-0.41992,-0.19653,0.064697,0.43091,0.5603,0.41992,0.085449,-0.22461,-0.448,-0.50171,-0.30151,-0.045166,0.23926,0.51025,0.56763,0.271,-0.021973,-0.26123,-0.5127,-0.34424,-0.037842,0.18433,0.37109,0.50293,0.42725,0.19775,-0.076904,-0.30273,-0.52612,-0.35034,0.025635,0.30273,0.52368,0.56152,0.38818,0.089111,-0.22339,-0.49805,-0.43457,-0.19897,0.14893,0.3894,0.52612,0.38086,0.12329,-0.17212,-0.33569,-0.47119,-0.33325,0.0036621,0.23193,0.4541,0.55054,0.35645,0.0097656,-0.18921,-0.39673,-0.43823,-0.24048,0.058594,0.39307,0.54199,0.49805,0.23682,-0.072021,-0.38574,-0.50293,-0.33569,-0.036621,0.27588,0.50537,0.51514,0.38574,0.092773,-0.23315,-0.46021,-0.35645,-0.13672,0.18921,0.4541,0.51514,0.47607,0.2832,-0.058594,-0.33203,-0.47363,-0.41382,-0.090332,0.13184,0.5127,0.46143,0.34424,0.048828,-0.16846,-0.40405,-0.39185,-0.18311,0.13428,0.41626,0.60425,0.49316,0.22583,-0.053711,-0.32715,-0.46143,-0.29541,-0.029297,0.14282,0.44434,0.48706,0.32959,0.090332,-0.19531,-0.47241,-0.43091,-0.22583,-0.024414,0.34912,0.52979,0.45044,0.23315,-0.070801,-0.42236,-0.58716,-0.41748,-0.14893,0.13672,0.41504,0.50049,0.36133,0.090332,-0.21729,-0.46509,-0.47485,-0.20874,0.040283,0.43457,0.48584,0.35034,0.21606,-0.0048828,-0.27954,-0.49683,-0.40771,-0.13672,0.15625,0.48584,0.64087,0.39673,0.061035,-0.063477,-0.40161,-0.4834,-0.2478,0.0061035,0.2478,0.52979,0.52979,0.22217,-0.11353,-0.36133,-0.5542,-0.41504,-0.14526,0.11475,0.35645,0.48462,0.39185,0.097656,-0.17822,-0.37476,-0.4834,-0.26123,0.03418,0.32349,0.54443,0.56396,0.33447,0.036621,-0.24414,-0.46143,-0.43457,-0.17334,0.19287,0.4126,0.57983,0.45654,0.12939,-0.17456,-0.34668,-0.49561,-0.33203,-0.0073242,0.30396,0.4834,0.50171,0.26489,-0.063477,-0.28687,-0.49316,-0.43457,-0.25757,0.03418,0.34424,0.46265,0.39795,0.13184,-0.080566,-0.43945,-0.448,-0.26611,0.037842,0.29785,0.47241,0.45898,0.30273,-0.0012207,-0.32593,-0.49805,-0.41748,-0.24658,0.11108,0.39551,0.42114,0.41504,0.13794,-0.19775,-0.37842,-0.47241,-0.29541,-0.074463,0.23071,0.47974,0.50415,0.29663,0.093994,-0.20996,-0.46387,-0.4248,-0.19165,0.061035,0.34302,0.56519,0.47607,0.20996,-0.05249,-0.31738,-0.46021,-0.23193,-0.058594,0.21362,0.50293,0.47119,0.29419,0.046387,-0.29419,-0.51514,-0.41748,-0.25391,0.03418,0.43335,0.5249,0.46509,0.24658,-0.01709,-0.29419,-0.46387,-0.29785,-0.054932,0.22705,0.51025,0.57617,0.3479,0.074463,-0.24414,-0.47119,-0.4895,-0.22949,0.037842,0.31128,0.48828,0.42114,0.1709,-0.12573,-0.35645,-0.50293,-0.31372,-0.01709,0.22949,0.49194,0.57129,0.40039,0.097656,-0.1416,-0.43823,-0.46997,-0.25513,0.020752,0.31128,0.56396,0.41504,0.16235,-0.1001,-0.30518,-0.49683,-0.34668,-0.075684,0.19531,0.45166,0.53711,0.3772,0.037842,-0.15991,-0.46265,-0.50293,-0.21118,0.083008,0.34302,0.46875,0.48828,0.27222,-0.040283,-0.2478,-0.47363,-0.3894,-0.064697,0.23193,0.45288,0.50415,0.40161,0.096436,-0.15625,-0.41382,-0.53955,-0.2832,-0.0036621,0.28809,0.49927,0.46387,0.18311,-0.029297,-0.31982,-0.43213,-0.31372,-0.10132,0.20142,0.50903,0.53833,0.49805,0.17578,-0.15747,-0.34668,-0.37598,-0.27344,0.023193,0.30884,0.49316,0.49072,0.32471,-0.029297,-0.29785,-0.47729,-0.39185,-0.12817,0.20142,0.45898,0.54443,0.46753,0.18311,-0.10254,-0.37964,-0.48828,-0.32104,0.021973,0.30518,0.49316,0.4834,0.27588,0.0024414,-0.25635,-0.46997,-0.35645,-0.12207,0.13184,0.44189,0.57861,0.34912,0.22095,-0.10742,-0.40527,-0.43945,-0.29663,-0.054932,0.27222,0.48828,0.46021,0.26123,0.0048828,-0.35522,-0.56152,-0.36621,-0.11108,0.065918,0.4541,0.50903,0.44556,0.2002,-0.087891,-0.34058,-0.45532,-0.32104,-0.026855,0.23438,0.47852,0.52246,0.29297,0.024414,-0.22949,-0.44922,-0.43091,-0.20874,0.064697,0.38208,0.53345,0.38208,0.12451,-0.028076,-0.40649,-0.47485,-0.24902,-0.079346,0.15747,0.48218,0.52002,0.30273,0.041504,-0.21606,-0.48706,-0.41504,-0.17212,0.096436,0.3479,0.57007,0.52246,0.25635,-0.042725,-0.28076,-0.47852,-0.32349,-0.0048828,0.21729,0.45044,0.54199,0.31128,-0.0024414,-0.26123,-0.48584,-0.45898,-0.16235,0.025635,0.39307,0.54199,0.47607,0.20874,-0.042725,-0.27832,-0.44434,-0.33569,-0.070801,0.17212,0.44922,0.51758,0.3772,0.042725,-0.22461,-0.47119,-0.51514,-0.28076,0.041504,0.32471,0.47241,0.45044,0.21362,-0.12573,-0.33325,-0.44922,-0.39307,-0.1123,0.22461,0.48462,0.49316,0.39551,0.065918,-0.22461,-0.42725,-0.48096,-0.25879,0.036621,0.2771,0.5127,0.50293,0.2771,-0.053711,-0.28809,-0.50171,-0.38574,-0.10254,0.15381,0.48828,0.54199,0.42358,0.097656,-0.17334,-0.47363,-0.5127,-0.26855,0.029297,0.39185,0.56519,0.39063,0.23071,-0.05127,-0.34424,-0.44067,-0.34668,-0.21362,0.095215,0.43945,0.53101,0.38208,0.11597,-0.21484,-0.45166,-0.47119,-0.2417,-0.0024414,0.33447,0.5127,0.51758,0.29053,-0.028076,-0.34058,-0.46387,-0.39185,-0.15381,0.11719,0.4248,0.44922,0.38818,0.14526,-0.073242,-0.4541,-0.49072,-0.28076,-0.10498,0.22095,0.57129,0.45166,0.22705,0.090332,-0.17578,-0.51025,-0.31128,-0.15747,0.10376,0.40894,0.56885,0.36377,0.075684,-0.20996,-0.41382,-0.52734,-0.32593,-0.05127,0.20752,0.50171,0.52002,0.30762,0.01709,-0.2832,-0.47485,-0.39185,-0.15137,0.087891,0.37354,0.55054,0.45166,0.20386,-0.085449,-0.40283,-0.55542,-0.31616,0.03418,0.25269,0.50537,0.55664,0.29297,0.031738,-0.2063,-0.48828,-0.448,-0.15137,0.12939,0.42603,0.52856,0.43579,0.14893,-0.12695,-0.3418,-0.47852,-0.39673,-0.10132,0.17822,0.43457,0.54443,0.32104,0.020752,-0.26001,-0.42969,-0.43823,-0.18311,0.1123,0.40039,0.55664,0.4895,0.16846,-0.15137,-0.37354,-0.52246,-0.40527,-0.080566,0.18188,0.40771,0.47119,0.32471,0.0097656,-0.24902,-0.44067,-0.48584,-0.2832,0.050049,0.31494,0.49805,0.49072,0.22461,-0.14038,-0.35645,-0.52002,-0.39429,-0.14038,0.17334,0.41504,0.4834,0.33813,0.13184,-0.1709,-0.42603,-0.39307,-0.14771,0.12939,0.45654,0.57373,0.47119,0.2832,0.0048828,-0.29541,-0.47607,-0.36499,-0.19409,0.1416,0.37109,0.45288,0.38452,0.076904,-0.24902,-0.44312,-0.45654,-0.28198,0.014648,0.38452,0.50537,0.44678,0.25269,-0.12085,-0.35034,-0.47363,-0.36133,-0.13428,0.12939,0.4126,0.50903,0.39307,0.096436,-0.15503,-0.43945,-0.49072,-0.2417,-0.018311,0.2832,0.51392,0.47852,0.26367,-0.015869,-0.28564,-0.46387,-0.37231,-0.15869,0.15381,0.42603,0.52612,0.36987,0.078125,-0.16113,-0.48096,-0.50537,-0.23193,0.014648,0.35889,0.59326,0.52734,0.28076,0.058594,-0.20386,-0.45166,-0.34668,-0.089111,0.063477,0.31982,0.50171,0.36377,0.090332,-0.16724,-0.448,-0.47119,-0.22217,0.057373,0.3186,0.54932,0.56641,0.35034,0.045166,-0.25879,-0.4834,-0.40161,-0.21484,0.093994,0.36865,0.45166,0.39063,0.08667,-0.16357,-0.36987,-0.49683,-0.31372,-0.01709,0.24048,0.49316,0.51636,0.26367,-0.014648,-0.23438,-0.48218,-0.45532,-0.21606,0.045166,0.40771,0.4834,0.4126,0.177,-0.11841,-0.32104,-0.43945,-0.31738,0.041504,0.3418,0.46265,0.53345,0.39307,0.05127,-0.25635,-0.42603,-0.41992,-0.20386,0.1123,0.29907,0.43579,0.39551,0.12451,-0.13428,-0.33203,-0.51758,-0.35889,-0.064697,0.19043,0.40771,0.48828,0.30151,0.024414,-0.25391,-0.47607,-0.46875,-0.24536,0.078125,0.3833,0.48096,0.40894,0.17334,-0.13672,-0.39063,-0.46997,-0.36621,-0.16602,0.18311,0.44556,0.448,0.33081,0.054932,-0.29053,-0.46875,-0.43701,-0.25879,0.05127,0.36621,0.49805,0.43091,0.18677,-0.10742,-0.34302,-0.49438,-0.37598,-0.1001,0.19043,0.50171,0.53833,0.4248,0.11475,-0.20508,-0.43701,-0.42969,-0.21973,0.059814,0.39063,0.46021,0.46143,0.26123,-0.021973,-0.33203,-0.49316,-0.32104,-0.062256,0.23926,0.57861,0.50781,0.33691,0.047607,-0.21851,-0.4541,-0.49438,-0.19287,-0.025635,0.32227,0.48706,0.4541,0.16113,-0.0085449,-0.34912,-0.50049,-0.33325,-0.057373,0.10742,0.49194,0.52002,0.37109,0.084229,-0.13794,-0.43091,-0.39063,-0.13794,-0.015869,0.29541,0.45166,0.42114,0.19165,0.018311,-0.27222,-0.56885,-0.37842,-0.056152,0.1123,0.46875,0.49805,0.35645,0.05249,-0.078125,-0.42236,-0.52246,-0.24292,0.0085449,0.26367,0.48706,0.48706,0.2002,-0.10376,-0.31982,-0.5127,-0.43457,-0.19043,0.13428,0.41016,0.53345,0.36865,0.068359,-0.15625,-0.34302,-0.44312,-0.24902,0.028076,0.29907,0.54199,0.48706,0.27222,-0.056152,-0.31738,-0.53101,-0.46509,-0.22827,0.091553,0.33203,0.50293,0.45166,0.15015,-0.177,-0.33447,-0.48096,-0.29785,0.053711,0.32593,0.48584,0.46143,0.26245,0.0048828,-0.28198,-0.4895,-0.39917,-0.20752,0.1123,0.48462,0.50903,0.42969,0.22461,-0.087891,-0.35034,-0.45898,-0.38574,-0.092773,0.19043,0.45898,0.49316,0.28076,-0.021973,-0.32471,-0.49927,-0.39307,-0.12573,0.10498,0.47485,0.46387,0.40527,0.16724,-0.16479,-0.40283,-0.4187,-0.29541,-0.095215,0.18921,0.47241,0.45654,0.29907,0.05127,-0.28687,-0.54932,-0.49316,-0.26978,-0.010986,0.31372,0.52734,0.41138,0.14893,-0.10864,-0.39917,-0.52979,-0.32715,-0.025635,0.23071,0.50659,0.50049,0.28076,0.026855,-0.30884,-0.53955,-0.39917,-0.18799,0.080566,0.45044,0.54199,0.48096,0.28076,0.024414,-0.28564,-0.4541,-0.32471,-0.053711,0.19165,0.52246,0.62256,0.39795,0.075684,-0.20996,-0.44312,-0.47852,-0.1416,0.063477,0.3125,0.49683,0.45166,0.16113,-0.096436,-0.28931,-0.43213,-0.29297,-0.012207,0.15137,0.40039,0.50659,0.39551,0.090332,-0.10498,-0.46387,-0.5188,-0.22461,0.08667,0.35278,0.58105,0.47485,0.19043,-0.0061035,-0.29663,-0.51147,-0.31616,-0.087891,0.18555,0.4541,0.5542,0.3479,0.029297,-0.22339,-0.42847,-0.49927,-0.22827,-0.063477,0.24536,0.44312,0.45166,0.25391,-0.091553,-0.32593,-0.46997,-0.40649,-0.18066,0.16846,0.41016,0.51025,0.42114,0.12817,-0.21362,-0.43335,-0.4834,-0.29663,0.0073242,0.31494,0.49561,0.44922,0.2832,-0.05127,-0.30518,-0.46509,-0.38452,-0.16357,0.1355,0.45166,0.47974,0.44678,0.17822,-0.16846,-0.41138,-0.47119,-0.36987,-0.087891,0.23682,0.46875,0.46387,0.2832,-0.048828,-0.32959,-0.47607,-0.37231,-0.14404,0.15747,0.47241,0.52734,0.38574,0.12329,-0.17822,-0.38818,-0.44556,-0.24902,0.024414,0.29297,0.55542,0.47363,0.25146,0.12817,-0.21851,-0.49561,-0.35645,-0.11475,0.057373,0.48096,0.52734,0.37476,0.15747,-0.12451,-0.41992,-0.46021,-0.23315,0.0097656,0.28564,0.56396,0.52368,0.29785,0.062256,-0.26611,-0.49194,-0.3479,-0.15137,0.092773,0.43213,0.55542,0.50293,0.25391,-0.056152,-0.35034,-0.54443,-0.36133,-0.043945,0.2124,0.45166,0.54321,0.29541,0.028076,-0.20142,-0.46875,-0.47363,-0.15015,0.13794,0.42114,0.6311,0.49194,0.20264,-0.042725,-0.26123,-0.47729,-0.30151,-0.064697,0.18555,0.5127,0.50903,0.28809,0.01709,-0.19653,-0.51514,-0.42725,-0.17334,0.014648,0.35645,0.58472,0.46753,0.29785,-0.041504,-0.26733,-0.53101,-0.35645,-0.021973,0.25146,0.45166,0.52734,0.36865,0.068359,-0.1709,-0.41016,-0.40771,-0.17822,0.11597,0.4248,0.57007,0.52246,0.31738,0.0048828,-0.27832,-0.50049,-0.36987,-0.17822,0.11353,0.37598,0.49561,0.35156,0.024414,-0.25635,-0.47729,-0.48706,-0.26733,0.029297,0.32593,0.46387,0.42969,0.24048,-0.1123,-0.33936,-0.43091,-0.38696,-0.10498,0.19531,0.47852,0.53467,0.3772,0.18066,-0.1123,-0.47119,-0.46753,-0.32227,-0.024414,0.32227,0.52246,0.46997,0.27344,-0.031738,-0.26611,-0.42847,-0.35522,-0.085449,0.16846,0.43701,0.49561,0.35645,0.084229,-0.18066,-0.43335,-0.47119,-0.2771,-0.046387,0.33203,0.54077,0.4834,0.34424,0.064697,-0.26855,-0.47852,-0.34424,-0.12939,0.098877,0.42236,0.52246,0.354,0.13062,-0.14893,-0.42725,-0.49805,-0.27344,0.0048828,0.32349,0.51514,0.49805,0.26978,0.0097656,-0.24048,-0.44312,-0.40527,-0.16235,0.068359,0.40649,0.54077,0.41748,0.15015,-0.14893,-0.42358,-0.54688,-0.27466,0.0012207,0.25146,0.55908,0.55542,0.25879,-0.014648,-0.24536,-0.53955,-0.3772,-0.13184,0.11353,0.44556,0.50781,0.37842,0.087891,-0.056152,-0.31494,-0.49194,-0.30029,0.012207,0.25269,0.53833,0.58716,0.29785,0.10376,-0.15137,-0.48828,-0.4126,-0.25635,0.041504,0.31616,0.52002,0.4187,0.12207,-0.19653,-0.40405,-0.52368,-0.31006,0.0024414,0.26611,0.46875,0.56885,0.38086,0.026855,-0.19409,-0.41504,-0.42358,-0.17578,0.11719,0.41504,0.54443,0.50537,0.26001,-0.05249,-0.32715,-0.49805,-0.3833,-0.031738,0.19531,0.41992,0.47607,0.36621,0.030518,-0.27222,-0.46997,-0.46997,-0.16479,0.092773,0.44434,0.55176,0.48828,0.25269,-0.067139,-0.31372,-0.48096,-0.38208,-0.079346,0.22583,0.48462,0.52002,0.41992,0.11353,-0.14648,-0.36865,-0.42114,-0.19775,0.0048828,0.31616,0.50049,0.43579,0.21973,-0.095215,-0.35156,-0.46143,-0.3418,-0.12085,0.21362,0.51758,0.53711,0.39063,0.092773,-0.22217,-0.45532,-0.42725,-0.22461,-0.0036621,0.34302,0.50293,0.448,0.2832,-0.01709,-0.36011,-0.51514,-0.39795,-0.18555,0.1355,0.3894,0.49438,0.38208,0.1123,-0.23071,-0.44189,-0.49927,-0.25879,-0.0024414,0.36133,0.51758,0.41626,0.19653,-0.053711,-0.32227,-0.52612,-0.41504,-0.16846,0.10254,0.43701,0.57373,0.41016,0.12817,-0.10742,-0.38086,-0.46265,-0.24536,-0.024414,0.24658,0.5481,0.52979,0.24658,-0.065918,-0.37109,-0.5542,-0.38574,-0.090332,0.10742,0.45776,0.47852,0.36865,0.076904,-0.091553,-0.4248,-0.46387,-0.19775,0.030518,0.35889,0.50903,0.50537,0.24048,0.078125,-0.16235,-0.47852,-0.41992,-0.15015,0.15503,0.40283,0.58716,0.50659,0.18066,-0.084229,-0.28809,-0.44434,-0.25879,0.093994,0.32837,0.55786,0.5249,0.27222,-0.05127,-0.29785,-0.51636,-0.38574,-0.16968,0.12695,0.45044,0.52612,0.49561,0.22339,-0.10864,-0.31128,-0.44922,-0.30518,0.021973,0.28809,0.52734,0.58472,0.40405,0.063477,-0.26489,-0.4541,-0.43213,-0.20874,0.1416,0.40771,0.50049,0.48584,0.23438,-0.070801,-0.27344,-0.43701,-0.3833,-0.075684,0.23193,0.48584,0.49927,0.31372,0.047607,-0.25635,-0.50171,-0.448,-0.2771,0.0048828,0.32715,0.49194,0.40405,0.19287,-0.10254,-0.34668,-0.45776,-0.31006,-0.083008,0.2356,0.51392,0.52246,0.37964,0.057373,-0.27588,-0.50293,-0.38086,-0.20142,0.070801,0.44067,0.54565,0.50049,0.32227,0.018311,-0.31616,-0.43335,-0.26855,-0.026855,0.14526,0.52124,0.49316,0.3418,0.08667,-0.22583,-0.51514,-0.53711,-0.26855,0.0048828,0.29175,0.47974,0.44067,0.15869,-0.10986,-0.34912,-0.50659,-0.31494,-0.05127,0.23438,0.5481,0.50903,0.31006,0.056152,-0.19043,-0.45898,-0.49561,-0.25513,-0.05127,0.26489,0.5481,0.43091,0.1709,-0.064697,-0.29785,-0.50903,-0.32349,-0.092773,0.13916,0.45166,0.59326,0.44922,0.1123,-0.18555,-0.45166,-0.50171,-0.26855,-0.0024414,0.25879,0.43213,0.43823,0.22583,-0.06958,-0.30762,-0.54688,-0.46631,-0.17944,0.10864,0.35156,0.50903,0.33936,0.078125,-0.15625,-0.39551,-0.53589,-0.31616,-0.013428,0.25024,0.49072,0.50903,0.2478,-0.063477,-0.27466,-0.46509,-0.40894,-0.1355,0.14771,0.41992,0.54077,0.47607,0.14282,-0.16724,-0.38086,-0.43823,-0.30518,0.043945,0.28931,0.4895,0.51392,0.3479,0.0354,-0.25024,-0.41992,-0.39551,-0.12817,0.2002,0.44678,0.55908,0.47119,0.20996,-0.12329,-0.33691,-0.51758,-0.3418,-0.012207,0.28076,0.5127,0.55298,0.24536,0.075684,-0.22461,-0.49194,-0.41748,-0.2417,0.073242,0.36743,0.49316,0.39917,0.20264,-0.14282,-0.38086,-0.4126,-0.2832,-0.070801,0.26367,0.51636,0.4834,0.32227,0.070801,-0.29175,-0.48096,-0.39551,-0.23315,0.10864,0.45898,0.53833,0.50415,0.27344,-0.043945,-0.31982,-0.48584,-0.36865,-0.073242,0.19653,0.43579,0.4834,0.31372,0.026855,-0.26733,-0.49805,-0.4248,-0.2002,0.053711,0.38086,0.52124,0.39185,0.17456,-0.081787,-0.3833,-0.54688,-0.34424,-0.11475,0.10498,0.45776,0.47119,0.27954,0.0354,-0.25269,-0.53711,-0.50415,-0.23682,0.0097656,0.30273,0.52856,0.44067,0.18677,-0.065918,-0.31128,-0.49072,-0.30273,-0.13672,0.1416,0.53467,0.5249,0.31006,0.040283,-0.1355,-0.48462,-0.42725,-0.16479,0.067139,0.40649,0.60303,0.55298,0.28931,0.031738,-0.25635,-0.5249,-0.36011,-0.068359,0.18188,0.38086,0.50049,0.34546,0.087891,-0.18555,-0.40771,-0.51514,-0.22705,0.093994,0.34912,0.51025,0.53711,0.30029,-0.024414,-0.2356,-0.4541,-0.36255,-0.080566,0.10986,0.50659,0.53345,0.41626,0.1001,-0.18555,-0.42358,-0.5127,-0.29419,0.026855,0.25879,0.49927,0.51514,0.2417,-0.090332,-0.26367,-0.4834,-0.42358,-0.042725,0.19897,0.44067,0.54199,0.41138,0.13672,-0.16479,-0.42114,-0.50659,-0.33081,-0.039063,0.30762,0.5188,0.51392,0.36743,0.047607,-0.27588,-0.44312,-0.36011,-0.14526,0.15625,0.43945,0.52734,0.45532,0.21118,-0.15869,-0.37598,-0.44678,-0.26611,-0.024414,0.30396,0.44678,0.50415,0.33447,-0.012207,-0.25879,-0.45532,-0.43945,-0.2002,0.12329,0.45654,0.51147,0.45654,0.21973,-0.11597,-0.4187,-0.50537,-0.36621,-0.056152,0.21851,0.48828,0.48828,0.24902,0.010986,-0.24658,-0.48828,-0.39307,-0.15503,0.083008,0.40283,0.53345,0.39551,0.14038,-0.11475,-0.36377,-0.47363,-0.33569,-0.089111,0.17822,0.48584,0.52124,0.34668,0.074463,-0.29175,-0.53589,-0.43335,-0.21973,0.029297,0.35889,0.55542,0.42847,0.18188,-0.095215,-0.36133,-0.54199,-0.31616,-0.048828,0.20996,0.47241,0.52368,0.30273,0.03418,-0.22461,-0.45654,-0.45898,-0.22217,0.046387,0.37354,0.5127,0.50293,0.23193,-0.074463,-0.31006,-0.48828,-0.41016,-0.12329,0.20142,0.43701,0.56885,0.3894,0.043945,-0.19653,-0.4187,-0.45044,-0.16235,0.11841,0.35889,0.55664,0.47974,0.19409,-0.11597,-0.29297,-0.51392,-0.35645,-0.15991,0.10986,0.3772,0.48706,0.39673,0.070801,-0.19043,-0.42236,-0.46631,-0.26367,0.050049,0.30518,0.49805,0.49072,0.28442,-0.072021,-0.35767,-0.54077,-0.40405,-0.13062,0.2002,0.42603,0.47119,0.37598,0.090332,-0.177,-0.39429,-0.42725,-0.28564,0.012207,0.3418,0.49561,0.47485,0.27344,-0.084229,-0.35645,-0.47729,-0.47607,-0.25513,0.093994,0.35034,0.47363,0.4187,0.11353,-0.22827,-0.4248,-0.45166,-0.30518,0.0073242,0.31128,0.49683,0.47607,0.36743,0.037842,-0.29785,-0.4895,-0.36011,-0.1123,0.068359,0.50293,0.47241,0.38574,0.1416,-0.14526,-0.44189,-0.51636,-0.30762,-0.072021,0.26245,0.51392,0.45044,0.26855,0.029297,-0.30273,-0.49683,-0.34424,-0.2417,0.070801,0.49438,0.48828,0.32593,0.18066,-0.12207,-0.39185,-0.47852,-0.26489,-0.064697,0.25635,0.56641,0.57373,0.32593,0.074463,-0.26611,-0.50659,-0.35767,-0.13794,0.11597,0.36133,0.56641,0.42847,0.17334,-0.14404,-0.39795,-0.54443,-0.2478,-0.01709,0.21606,0.46753,0.4834,0.25146,0.025635,-0.18311,-0.50903,-0.48828,-0.26978,0.029297,0.35645,0.4895,0.40161,0.13916,-0.090332,-0.30762,-0.50537,-0.35034,-0.053711,0.18433,0.47485,0.5957,0.32715,-0.014648,-0.2417,-0.45654,-0.44922,-0.21606,0.092773,0.32104,0.50293,0.45532,0.14282,-0.041504,-0.34058,-0.45166,-0.29297,-0.08667,0.1416,0.4541,0.53101,0.36621,0.065918,-0.20386,-0.47241,-0.50415,-0.26245,0.037842,0.27466,0.47363,0.4126,0.2002,-0.098877,-0.33691,-0.49805,-0.39917,-0.12207,0.21851,0.47607,0.47852,0.37354,0.03418,-0.271,-0.44434,-0.43701,-0.24902,0.11963,0.44189,0.55176,0.53589,0.28076,-0.047607,-0.30029,-0.44922,-0.36255,-0.092773,0.15869,0.41626,0.55176,0.46753,0.16235,-0.14526,-0.41748,-0.4248,-0.18555,-0.0073242,0.28931,0.45532,0.42114,0.22461,-0.087891,-0.3894,-0.47852,-0.33081,-0.065918,0.14404,0.46143,0.48096,0.36133,0.14771,-0.14648,-0.40283,-0.46753,-0.30518,-0.046387,0.27222,0.49072,0.44678,0.2124,-0.061035,-0.3125,-0.49072,-0.37964,-0.1709,0.1123,0.43091,0.57129,0.40039,0.1062,-0.19409,-0.43945,-0.47852,-0.28564,-0.06958,0.23926,0.50659,0.46021,0.25146,-0.040283,-0.33936,-0.53467,-0.38452,-0.17334,0.078125,0.35645,0.53711,0.41626,0.16235,-0.12573,-0.41138,-0.52368,-0.31372,-0.0036621,0.25879,0.45776,0.45898,0.25024,-0.036621,-0.24414,-0.49927,-0.46265,-0.17456,0.13306,0.40649,0.53589,0.43945,0.1416,-0.08667,-0.31616,-0.49316,-0.34058,-0.021973,0.18188,0.48584,0.51758,0.24414,-0.053711,-0.29785,-0.5542,-0.41138,-0.10986,0.058594,0.42236,0.51025,0.44556,0.12329,-0.046387,-0.37354,-0.45776,-0.29785,0.03418,0.28809,0.4248,0.45654,0.3064,0.015869,-0.20752,-0.47607,-0.42725,-0.2832,0.050049,0.31982,0.47485,0.44922,0.2124,-0.11841,-0.3125,-0.44556,-0.35278,-0.040283,0.26123,0.46631,0.52002,0.32715,-0.0073242,-0.29541,-0.45898,-0.50537,-0.30518,0.057373,0.448,0.47852,0.46143,0.21729,-0.12451,-0.32593,-0.48096,-0.28809,-0.10986,0.17578,0.46387,0.47607,0.35889,0.075684,-0.25635,-0.52002,-0.50659,-0.26001,0.036621,0.45654,0.47729,0.37598,0.2832,-0.0073242,-0.29541,-0.45776,-0.33691,-0.10742,0.21362,0.53345,0.53101,0.41992,0.13672,-0.19409,-0.5249,-0.44678,-0.32837,-0.058594,0.2771,0.50903,0.43457,0.23926,-0.042725,-0.33813,-0.4834,-0.28564,-0.074463,0.16602,0.47729,0.51636,0.34302,0.10864,-0.21362,-0.49438,-0.51514,-0.28931,-0.037842,0.31128,0.54199,0.51025,0.28442,-0.013428,-0.31982,-0.48584,-0.36865,-0.12451,0.12207,0.38086,0.50659,0.35522,0.070801,-0.23926,-0.46265,-0.5603,-0.30029,-0.047607,0.25879,0.49805,0.54443,0.21484,-0.03418,-0.25146,-0.51025,-0.41992,-0.10986,0.085449,0.40771,0.55908,0.40283,0.075684,-0.15503,-0.39795,-0.54077,-0.32471,-0.014648,0.20142,0.49316,0.51025,0.24414,-0.018311,-0.25391,-0.49072,-0.4126,-0.17334,0.11719,0.4126,0.54199,0.43945,0.17334,-0.15747,-0.40649,-0.52979,-0.39063,-0.026855,0.23926,0.4248,0.49072,0.28198,-0.057373,-0.2771,-0.45044,-0.44067,-0.16357,0.15137,0.36255,0.50781,0.45654,0.18066,-0.12695,-0.31738,-0.46265,-0.32104,0.019531,0.29785,0.42847,0.49805,0.31738,-0.021973,-0.29541,-0.51025,-0.42969,-0.23804,0.093994,0.4541,0.50537,0.4834,0.18433,-0.14893,-0.36987,-0.47974,-0.40283,-0.079346,0.18188,0.40039,0.49072,0.31616,0.019531,-0.16846,-0.43457,-0.37842,-0.12817,0.16602,0.47852,0.58472,0.3894,0.29053,-0.021973,-0.31006,-0.45654,-0.31372,-0.075684,0.26611,0.57495,0.54443,0.2832,0.15747,-0.15747,-0.47119,-0.34058,-0.27588,-0.012207,0.33691,0.5188,0.4248,0.26367,-0.056152,-0.35645,-0.48096,-0.32959,-0.10376,0.12329,0.40039,0.48096,0.35278,0.076904,-0.21729,-0.48706,-0.43945,-0.15381,0.098877,0.46753,0.51392,0.41016,0.22949,-0.023193,-0.31738,-0.49683,-0.35645,-0.079346,0.19531,0.46021,0.57617,0.3833,0.1001,-0.13428,-0.39551,-0.4834,-0.14648,0.12207,0.37354,0.52612,0.48462,0.21973,-0.089111,-0.29297,-0.51514,-0.41382,-0.16846,0.083008,0.44189,0.4834,0.42847,0.14771,-0.14648,-0.44189,-0.48096,-0.21118,0.03418,0.29175,0.60547,0.57129,0.22583,0.040283,-0.21973,-0.52002,-0.3479,-0.058594,0.12207,0.3894,0.51392,0.3833,0.10498,-0.15137,-0.35278,-0.46387,-0.33325,0.020752,0.32227,0.49927,0.51392,0.33691,-0.036621,-0.26611,-0.44312,-0.44312,-0.18433,0.098877,0.30762,0.46387,0.38574,0.087891,-0.19043,-0.38208,-0.52856,-0.31006,0.048828,0.24414,0.46631,0.52002,0.28809,-0.041504,-0.25879,-0.45532,-0.4541,-0.18311,0.096436,0.41626,0.49072,0.46509,0.19653,-0.12573,-0.38452,-0.45776,-0.34546,-0.056152,0.28076,0.52002,0.4895,0.30518,0.059814,-0.28198,-0.44678,-0.38818,-0.21362,0.046387,0.38574,0.49683,0.40771,0.17212,-0.15503,-0.37109,-0.46997,-0.28809,-0.023193,0.28076,0.5188,0.57007,0.43701,0.14526,-0.20996,-0.41382,-0.3833,-0.18799,0.031738,0.4187,0.51514,0.3894,0.16968,-0.12817,-0.38086,-0.50659,-0.21484,-0.075684,0.19043,0.5481,0.57495,0.37598,0.12573,-0.17334,-0.45898,-0.42236,-0.19653,0.091553,0.43945,0.53955,0.50049,0.271,-0.021973,-0.354,-0.54565,-0.41748,-0.11597,0.16113,0.43213,0.54443,0.34912,0.070801,-0.18555,-0.47363,-0.4834,-0.2063,0.024414,0.39551,0.53955,0.40771,0.12573,-0.0024414,-0.34912,-0.52856,-0.31372,-0.14038,0.084229,0.44189,0.53223,0.38208,0.089111,-0.15747,-0.44678,-0.4834,-0.2417,0.029297,0.28198,0.49927,0.43457,0.19043,-0.12939,-0.37231,-0.55908,-0.35156,-0.15015,0.16357,0.51758,0.59814,0.49927,0.15015,-0.12207,-0.34058,-0.42725,-0.16602,0.021973,0.26611,0.45898,0.51392,0.24414,-0.091553,-0.30029,-0.52002,-0.46997,-0.16113,0.12451,0.34912,0.5127,0.40283,0.053711,-0.093994,-0.39307,-0.46753,-0.20996,0.026855,0.38208,0.45898,0.42603,0.21973,-0.1001,-0.34668,-0.46265,-0.40771,-0.12695,0.1709,0.46631,0.52612,0.48584,0.19775,-0.1123,-0.35645,-0.50171,-0.32837,0,0.27832,0.55664,0.50659,0.271,-0.048828,-0.32349,-0.46387,-0.38818,-0.16846,0.12695,0.44434,0.51025,0.40039,0.13794,-0.20508,-0.39551,-0.42969,-0.33081,-0.085449,0.2002,0.4541,0.44312,0.36499,0.097656,-0.2771,-0.53589,-0.45898,-0.22827,0.090332,0.36377,0.52979,0.44556,0.22217,-0.10986,-0.34546,-0.49194,-0.28442,-0.058594,0.26001,0.49561,0.49805,0.26123,-0.025635,-0.2832,-0.52368,-0.45654,-0.24902,0.021973,0.38086,0.50415,0.40283,0.17822,0.025635,-0.30762,-0.4895,-0.28564,-0.020752,0.23682,0.51392,0.58228,0.29907,0.12207,-0.16724,-0.53711,-0.50415,-0.21851,0.079346,0.36865,0.52979,0.4248,0.16846,-0.075684,-0.34424,-0.46021,-0.32959,-0.078125,0.16113,0.54688,0.57739,0.41992,0.11353,-0.17212,-0.46875,-0.46997,-0.19897,0.092773,0.34302,0.58472,0.49316,0.18066,-0.020752,-0.37964,-0.4895,-0.32104,0.0354,0.26855,0.40649,0.49805,0.37109,0.031738,-0.2356,-0.41504,-0.50903,-0.25879,0.021973,0.28931,0.46875,0.47119,0.21973,-0.067139,-0.29053,-0.47729,-0.35889,-0.076904,0.21362,0.46631,0.57617,0.4248,0.074463,-0.23438,-0.42725,-0.52246,-0.24536,0.05127,0.34424,0.52124,0.43091,0.23926,-0.074463,-0.28809,-0.42725,-0.34546,-0.12207,0.20386,0.46875,0.50659,0.46387,0.16357,-0.15869,-0.39307,-0.51025,-0.354,-0.03418,0.30273,0.49805,0.50049,0.32471,0.0012207,-0.28687,-0.44312,-0.36255,-0.14404,0.20996,0.47607,0.53223,0.41016,0.10254,-0.20264,-0.41504,-0.48218,-0.33325,-0.056152,0.22583,0.4834,0.50049,0.33203,0.020752,-0.32349,-0.49805,-0.3833,-0.18799,0.10742,0.40527,0.51025,0.41504,0.20508,-0.15137,-0.42358,-0.47607,-0.29785,-0.042725,0.24536,0.46387,0.4541,0.22461,-0.014648,-0.29297,-0.50903,-0.38574,-0.1416,0.12207,0.46753,0.59692,0.50659,0.29297,-0.0048828,-0.31738,-0.47119,-0.28442,-0.0024414,0.23315,0.55664,0.5542,0.3125,0.084229,-0.23682,-0.46509,-0.35034,-0.1062,0.11475,0.47241,0.60547,0.46143,0.18799,-0.068359,-0.31372,-0.49805,-0.36621,-0.065918,0.18066,0.46509,0.53223,0.31494,0.048828,-0.2417,-0.51392,-0.4834,-0.21606,0.12817,0.38208,0.57129,0.5188,0.2356,-0.081787,-0.30396,-0.54565,-0.36865,-0.062256,0.16113,0.41504,0.48828,0.30396,0.019531,-0.21606,-0.43945,-0.4895,-0.21973,0.087891,0.37109,0.52612,0.54443,0.26978,-0.0085449,-0.24658,-0.47974,-0.41382,-0.090332,0.2124,0.46021,0.56641,0.40039,0.089111,-0.20752,-0.45166,-0.41992,-0.23071,0.037842,0.354,0.5127,0.47974,0.24536,-0.092773,-0.31372,-0.45044,-0.36865,-0.12451,0.13916,0.43457,0.55176,0.48096,0.17578,-0.17456,-0.4126,-0.44434,-0.26245,0.078125,0.37598,0.52002,0.51758,0.36255,-0.012207,-0.29297,-0.41992,-0.30884,-0.089111,0.2417,0.49805,0.48096,0.3833,0.1123,-0.20264,-0.43091,-0.46997,-0.29053,-0.024414,0.31128,0.51392,0.47974,0.30396,0.0024414,-0.31616,-0.46143,-0.40039,-0.18066,0.10742,0.39185,0.49072,0.39063,0.1123,-0.2002,-0.47974,-0.49316,-0.2478,0.031738,0.31982,0.53467,0.5188,0.28687,0.046387,-0.2478,-0.46997,-0.36377,-0.11719,0.076904,0.43579,0.54199,0.39307,0.15625,-0.087891,-0.40771,-0.49072,-0.28931,-0.041504,0.25513,0.59692,0.53345,0.30029,0.031738,-0.28809,-0.49194,-0.33691,-0.12939,0.14404,0.40039,0.50781,0.39917,0.18188,-0.13672,-0.32227,-0.46387,-0.33936,-0.0048828,0.2478,0.48706,0.56763,0.31738,0.0012207,-0.26123,-0.50903,-0.43335,-0.10986,0.12817,0.36377,0.61035,0.45532,0.11841,-0.13062,-0.36255,-0.57129,-0.32959,-0.046387,0.18188,0.43945,0.49927,0.27832,0.0012207,-0.23682,-0.47241,-0.44678,-0.18921,0.13428,0.43945,0.55298,0.43579,0.18555,-0.12573,-0.35034,-0.53589,-0.41504,-0.087891,0.16113,0.41504,0.5127,0.32104,0.10986,-0.16235,-0.42603,-0.46143,-0.14648,0.070801,0.3479,0.48096,0.43945,0.13794,-0.16846,-0.3894,-0.52368,-0.3894,-0.18066,0.12329,0.39673,0.48828,0.41748,0.089111,-0.25269,-0.41382,-0.41504,-0.2063,0.065918,0.32715,0.50049,0.46387,0.23315,-0.10254,-0.36865,-0.52002,-0.37109,-0.089111,0.2124,0.43945,0.49683,0.37842,0.065918,-0.15381,-0.40161,-0.42725,-0.22461,0.078125,0.41748,0.54688,0.49072,0.31372,-0.01709,-0.39551,-0.52002,-0.4126,-0.18799,0.13916,0.43213,0.49438,0.36133,0.096436,-0.19043,-0.43823,-0.4541,-0.23926,-0.023193,0.27954,0.4834,0.45166,0.22949,-0.045166,-0.35645,-0.55054,-0.35034,-0.18311,0.095215,0.43701,0.49316,0.36865,0.13916,-0.177,-0.49561,-0.49805,-0.2771,-0.020752,0.29419,0.51392,0.42725,0.22583,-0.056152,-0.32471,-0.52979,-0.33447,-0.091553,0.085449,0.49072,0.49561,0.35645,0.076904,-0.11963,-0.4248,-0.49316,-0.27588,0.0073242,0.26123,0.54932,0.51758,0.23193,0.030518,-0.24658,-0.53223,-0.40283,-0.18311,0.063477,0.33569,0.52002,0.41504,0.11963,-0.13062,-0.33936,-0.49927,-0.19531,-0.012207,0.22827,0.55664,0.60791,0.32837,0.063477,-0.21118,-0.52979,-0.44312,-0.2002,0.085449,0.4248,0.56152,0.49194,0.23315,-0.075684,-0.33325,-0.46387,-0.3186,0.03418,0.27954,0.50659,0.57495,0.35034,0.054932,-0.18799,-0.47607,-0.44189,-0.18311,0.14282,0.44434,0.45166,0.43213,0.177,-0.14648,-0.354,-0.45166,-0.38574,-0.081787,0.22827,0.42847,0.48462,0.37354,0.0012207,-0.21729,-0.41748,-0.46387,-0.2063,0.10498,0.35034,0.41992,0.43213,0.16968,-0.11841,-0.33691,-0.48584,-0.33447,-0.0354,0.24414,0.448,0.51758,0.36621,0.080566,-0.22949,-0.46753,-0.48218,-0.26855,0.014648,0.36743,0.52002,0.46021,0.27344,-0.026855,-0.33936,-0.48706,-0.36865,-0.18433,0.12695,0.52246,0.47607,0.31006,0.10986,-0.16968,-0.38452,-0.40527,-0.19775,0.037842,0.37109,0.54199,0.38574,0.2002,-0.0024414,-0.32837,-0.46631,-0.27832,-0.16113,0.15015,0.50293,0.5481,0.40649,0.12695,-0.18433,-0.50537,-0.51636,-0.26611,-0.029297,0.271,0.52002,0.40527,0.21362,-0.046387,-0.32715,-0.53955,-0.39429,-0.13184,0.15259,0.43335,0.53955,0.39307,0.10254,-0.15625,-0.41138,-0.48462,-0.32227,-0.046387,0.25757,0.46753,0.49438,0.25391,-0.043945,-0.32349,-0.4895,-0.39551,-0.12451,0.092773,0.34546,0.55908,0.4248,0.095215,-0.1416,-0.41748,-0.51147,-0.18677,-0.0024414,0.22949,0.45654,0.47363,0.22949,-0.047607,-0.26367,-0.47363,-0.41748,-0.15259,0.11841,0.45166,0.54443,0.44678,0.16724,-0.12939,-0.37354,-0.48828,-0.28687,-0.098877,0.18555,0.4541,0.47485,0.25879,-0.046387,-0.3064,-0.47852,-0.37842,-0.11963,0.1062,0.42725,0.54932,0.52734,0.20752,-0.12085,-0.30273,-0.4541,-0.36255,-0.014648,0.28076,0.50537,0.48462,0.30762,0.015869,-0.29419,-0.47729,-0.46387,-0.22705,0.079346,0.41016,0.55176,0.49927,0.24292,-0.024414,-0.2771,-0.44678,-0.28564,-0.0061035,0.25024,0.49561,0.53833,0.34668,0.10376,-0.22339,-0.47485,-0.40894,-0.22339,0.029297,0.42725,0.51514,0.46265,0.25024,-0.081787,-0.31494,-0.41504,-0.36377,-0.11108,0.21973,0.44678,0.50415,0.35889,0.073242,-0.21484,-0.43701,-0.45288,-0.20752,0.054932,0.35767,0.52002,0.43457,0.21973,-0.063477,-0.36133,-0.49072,-0.29297,-0.026855,0.11841,0.5127,0.56396,0.43457,0.14648,-0.19043,-0.46875,-0.43945,-0.18433,0.079346,0.36621,0.50781,0.46387,0.2771,-0.012207,-0.30518,-0.48706,-0.30762,-0.11353,0.18066,0.46143,0.50659,0.36255,0.080566,-0.17944,-0.44922,-0.49194,-0.23804,0.03418,0.28564,0.54443,0.52246,0.2417,-0.012207,-0.25757,-0.4834,-0.40161,-0.08667,0.12207,0.37964,0.54199,0.36987,0.084229,-0.16357,-0.44312,-0.51392,-0.23682,0.0085449,0.32349,0.51758,0.49561,0.27588,0.01709,-0.26245,-0.47729,-0.40161,-0.12573,0.14893,0.44678,0.53833,0.41382,0.11597,-0.16968,-0.35034,-0.52734,-0.37476,-0.021973,0.25513,0.46753,0.56152,0.28076,-0.076904,-0.26367,-0.45044,-0.36621,-0.20508,0.092773,0.44434,0.48706,0.41626,0.11963,-0.23682,-0.41748,-0.54321,-0.38818,-0.067139,0.2063,0.4541,0.50903,0.3064,-0.019531,-0.25146,-0.47607,-0.46387,-0.19409,0.096436,0.30884,0.47974,0.38818,0.11353,-0.11475,-0.35034,-0.49194,-0.32593,-0.012207,0.30762,0.55908,0.51147,0.39795,0.12451,-0.21973,-0.43213,-0.354,-0.20996,0.12939,0.52612,0.55176,0.51025,0.27588,-0.070801,-0.37476,-0.47852,-0.33081,-0.046387,0.2356,0.50903,0.45654,0.28809,0.013428,-0.17944,-0.46753,-0.35889,-0.18311,0.057373,0.34668,0.46997,0.40039,0.17822,-0.11353,-0.40405,-0.5249,-0.38086,-0.1062,0.18311,0.5127,0.51758,0.354,0.095215,-0.23315,-0.50537,-0.48218,-0.24048,0.01709,0.32349,0.52734,0.46021,0.17456,-0.10132,-0.3418,-0.54932,-0.38574,-0.070801,0.13672,0.41504,0.55176,0.35645,0.068359,-0.18433,-0.45776,-0.49805,-0.18921,0.0073242,0.32471,0.52246,0.46143,0.20508,-0.05127,-0.354,-0.54688,-0.32104,-0.05249,0.19165,0.46509,0.57129,0.40283,0.13184,-0.15259,-0.43701,-0.47485,-0.16968,0.10254,0.3772,0.55176,0.5249,0.29297,-0.030518,-0.29541,-0.48584,-0.42725,-0.13184,0.1123,0.45654,0.45532,0.39795,0.053711,-0.13428,-0.36621,-0.46875,-0.25635,0.10132,0.32715,0.55542,0.48828,0.33447,-0.024414,-0.25269,-0.4541,-0.33447,-0.090332,0.068359,0.45166,0.52612,0.41016,0.085449,-0.20508,-0.41504,-0.50293,-0.31982,-0.01709,0.28442,0.45898,0.50049,0.3479,-0.0097656,-0.32715,-0.50171,-0.42725,-0.21118,0.12939,0.40039,0.4541,0.38696,0.20142,-0.15869,-0.4126,-0.4541,-0.31372,-0.021973,0.32471,0.51392,0.48218,0.26123,-0.018311,-0.26123,-0.49805,-0.44067,-0.26733,0.026855,0.48828,0.49316,0.39795,0.13916,-0.10254,-0.36133,-0.39795,-0.30273,-0.030518,0.28564,0.53467,0.5127,0.36133,0.081787,-0.27222,-0.50415,-0.38696,-0.177,0.076904,0.41992,0.51514,0.40527,0.19653,-0.056152,-0.39063,-0.51758,-0.28076,-0.041504,0.2124,0.50903,0.51392,0.33447,0.095215,-0.25146,-0.53223,-0.5127,-0.29175,0.0048828,0.31982,0.49072,0.41016,0.18799,-0.072021,-0.29663,-0.47119,-0.28076,0,0.25269,0.50659,0.5896,0.31006,0.046387,-0.20752,-0.49194,-0.46631,-0.19043,-0.043945,0.30273,0.5127,0.39673,0.1416,-0.092773,-0.35278,-0.5249,-0.36133,-0.095215,0.18066,0.40649,0.53955,0.38086,0.098877,-0.22461,-0.44189,-0.49194,-0.25757,0.015869,0.3064,0.47485,0.4248,0.17578,-0.046387,-0.25391,-0.47119,-0.34912,-0.013428,0.21973,0.45898,0.60791,0.38208,0.054932,-0.072021,-0.43457,-0.54565,-0.29053,0.0048828,0.23071,0.47119,0.44189,0.22217,-0.10742,-0.32715,-0.50293,-0.39673,-0.12573,0.19409,0.46143,0.53955,0.40527,0.090332,-0.22217,-0.41016,-0.43457,-0.28198,-0.024414,0.31128,0.49316,0.45532,0.29175,-0.053711,-0.33936,-0.46631,-0.35645,-0.079346,0.1123,0.43945,0.54199,0.51514,0.22339,-0.093994,-0.354,-0.49927,-0.30518,0.046387,0.3418,0.50659,0.47363,0.28076,0.014648,-0.30518,-0.448,-0.43823,-0.19897,0.10254,0.46753,0.47607,0.38696,0.15381,-0.13062,-0.35767,-0.41016,-0.27588,-0.076904,0.25635,0.52612,0.52246,0.33325,0.041504,-0.271,-0.52979,-0.44312,-0.17578,0.091553,0.40527,0.54199,0.41138,0.14893,-0.098877,-0.39795,-0.47852,-0.29297,-0.036621,0.24048,0.46875,0.47852,0.32959,0.11353,-0.21606,-0.54077,-0.39551,-0.21606,0.040283,0.42358,0.52002,0.36743,0.15869,0.031738,-0.28809,-0.50415,-0.27222,0.036621,0.29419,0.48584,0.50171,0.25024,-0.028076,-0.25146,-0.48584,-0.49072,-0.177,0.040283,0.46753,0.61279,0.52612,0.23926,-0.040283,-0.31982,-0.46265,-0.29541,-0.053711,0.18188,0.48706,0.48706,0.31494,0.028076,-0.26855,-0.51758,-0.49316,-0.18433,0.11597,0.36621,0.58105,0.58105,0.28564,-0.0048828,-0.23315,-0.45898,-0.35645,-0.0085449,0.24902,0.46997,0.53223,0.42847,0.083008,-0.17822,-0.39307,-0.44067,-0.19897,0.074463,0.32227,0.53955,0.5127,0.26978,-0.014648,-0.25879,-0.46143,-0.33936,-0.058594,0.2124,0.49927,0.5542,0.37231,0.024414,-0.24658,-0.48218,-0.52368,-0.28809,0.010986,0.32593,0.45532,0.46387,0.26001,-0.076904,-0.32471,-0.45532,-0.33691,-0.089111,0.2063,0.43579,0.53223,0.47607,0.19409,-0.14893,-0.42236,-0.47729,-0.2124,0.10498,0.41504,0.46753,0.46509,0.24902,-0.01709,-0.30762,-0.48218,-0.39185,-0.15869,0.13306,0.46631,0.50659,0.37109,0.11963,-0.16846,-0.42847,-0.48706,-0.36499,-0.11719,0.22949,0.54443,0.50659,0.30762,-0.0024414,-0.26855,-0.42969,-0.31982,-0.12695,0.14893,0.49072,0.61768,0.51514,0.22583,-0.12329,-0.46387,-0.49561,-0.19775,-0.026855,0.20264,0.50415,0.49805,0.27222,0.037842,-0.25879,-0.52368,-0.40405,-0.1355,0.11597,0.36377,0.52856,0.36865,0.15991,-0.12207,-0.39063,-0.52368,-0.35156,-0.087891,0.24292,0.49316,0.49561,0.26123,0,-0.22217,-0.47852,-0.4126,-0.1709,0.061035,0.39429,0.56152,0.45898,0.14648,-0.1123,-0.36255,-0.52979,-0.37476,-0.05249,0.15747,0.43335,0.51636,0.33447,-0.0061035,-0.23438,-0.47363,-0.44678,-0.19897,0.13428,0.36377,0.47852,0.46387,0.19653,-0.10376,-0.33691,-0.53589,-0.41016,-0.057373,0.17212,0.448,0.54443,0.36865,0.06958,-0.19409,-0.44434,-0.4834,-0.19775,0.1001,0.34302,0.52734,0.48096,0.17822,-0.097656,-0.31372,-0.46143,-0.36377,-0.073242,0.20508,0.45532,0.52734,0.4126,0.068359,-0.27466,-0.44189,-0.45288,-0.26978,0.025635,0.38452,0.50415,0.4834,0.2832,-0.081787,-0.35767,-0.50049,-0.41504,-0.18677,0.14282,0.34912,0.43701,0.33813,0.031738,-0.26001,-0.42969,-0.44067,-0.18799,0.0354,0.40771,0.46143,0.43213,0.23438,-0.090332,-0.33203,-0.49683,-0.38696,-0.2417,0.096436,0.54443,0.48218,0.36865,0.095215,-0.11719,-0.47485,-0.39185,-0.29053,-0.058594,0.2832,0.50903,0.44189,0.25146,-0.05127,-0.34058,-0.50903,-0.30884,-0.19531,0.085449,0.35278,0.43945,0.3479,0.10742,-0.20996,-0.44556,-0.44434,-0.26001,-0.0085449,0.32593,0.56152,0.51025,0.30029,0.019531,-0.36133,-0.58472,-0.43213,-0.16602,0.073242,0.41138,0.52246,0.38818,0.18311,-0.090332,-0.37476,-0.46509,-0.25391,0.03418,0.30273,0.53711,0.52124,0.29785,0.045166,-0.19897,-0.51147,-0.42358,-0.27588,0.03418,0.36743,0.51514,0.41748,0.12817,-0.15503,-0.40161,-0.49805,-0.31372,-0.032959,0.16113,0.4834,0.53833,0.29785,0.025635,-0.26367,-0.53711,-0.43701,-0.15991,0.098877,0.31738,0.47363,0.41626,0.17334,-0.10986,-0.31372,-0.52246,-0.33325,-0.0036621,0.24414,0.49316,0.55298,0.36987,0.048828,-0.19775,-0.4187,-0.45532,-0.25269,0.036621,0.31738,0.51147,0.42847,0.12207,-0.15991,-0.3186,-0.46753,-0.33569,-0.053711,0.19043,0.42969,0.54443,0.32227,0.03418,-0.27466,-0.49805,-0.46631,-0.25146,0.05249,0.36865,0.44922,0.45654,0.24658,-0.091553,-0.31738,-0.47974,-0.38818,-0.081787,0.20508,0.41748,0.4834,0.40527,0.10132,-0.21729,-0.43457,-0.45776,-0.23926,0.037842,0.32349,0.47607,0.42358,0.17334,-0.14526,-0.33203,-0.49683,-0.39307,-0.13672,0.13916,0.46753,0.54932,0.39429,0.097656,-0.18555,-0.42603,-0.42847,-0.27222,-0.01709,0.29297,0.47241,0.42603,0.26611,-0.061035,-0.3418,-0.51758,-0.33081,-0.1062,0.16724,0.46387,0.51758,0.38818,0.14282,-0.15015,-0.45898,-0.50903,-0.26367,-0.019531,0.30884,0.49927,0.41504,0.18311,0.053711,-0.25391,-0.47363,-0.36621,-0.10376,0.18066,0.46509,0.5957,0.41016,0.1416,-0.092773,-0.39063,-0.41626,-0.22339,0.021973,0.30151,0.51758,0.47852,0.25146,-0.037842,-0.26245,-0.54077,-0.43335,-0.12207,0.10986,0.42725,0.60303,0.49683,0.18555,-0.085449,-0.40894,-0.5481,-0.32959,0,0.19653,0.44556,0.51025,0.26001,-0.054932,-0.27466,-0.52246,-0.42114,-0.13062,0.13184,0.39551,0.54321,0.44312,0.17578,-0.076904,-0.32227,-0.52246,-0.34058,-0.023193,0.24048,0.48462,0.53711,0.31494,0.010986,-0.22217,-0.45532,-0.42725,-0.14526,0.18799,0.33691,0.50171,0.41992,0.15747,-0.13916,-0.32593,-0.44434,-0.34058,-0.056152,0.22095,0.44922,0.49561,0.33447,0.0012207,-0.29053,-0.50293,-0.48218,-0.26855,0.083008,0.38818,0.48096,0.48828,0.23071,-0.1001,-0.34912,-0.49561,-0.37598,-0.058594,0.22949,0.44067,0.49194,0.35034,0.059814,-0.22461,-0.43579,-0.44678,-0.22583,0.093994,0.38574,0.49805,0.44556,0.23315,-0.089111,-0.3418,-0.45898,-0.39673,-0.18433,0.15259,0.53711,0.48462,0.32959,0.11353,-0.22949,-0.51758,-0.47852,-0.18188,-0.023193,0.31006,0.49438,0.42847,0.21606,-0.076904,-0.33691,-0.47974,-0.33691,-0.12207,0.17456,0.50537,0.53345,0.42603,0.16357,-0.15747,-0.42358,-0.44556,-0.23804,-0.021973,0.35645,0.52002,0.47363,0.28076,-0.0048828,-0.30273,-0.51636,-0.37109,-0.18188,0.10376,0.47852,0.57251,0.39551,0.084229,-0.18066,-0.4187,-0.46997,-0.20752,0.075684,0.3833,0.52246,0.46509,0.20996,-0.065918,-0.29053,-0.50171,-0.33203,-0.08667,0.074463,0.49072,0.50903,0.39185,0.093994,-0.10498,-0.46997,-0.54688,-0.28809,0.0012207,0.23682,0.52979,0.49072,0.27222,0.0048828,-0.28809,-0.50049,-0.39429,-0.083008,0.16968,0.41626,0.5127,0.41016,0.11841,-0.14526,-0.38818,-0.51514,-0.38818,-0.048828,0.2124,0.43091,0.48706,0.24658,0.0354,-0.2417,-0.46143,-0.41504,-0.10742,0.1709,0.45288,0.61157,0.39551,0.18799,-0.12085,-0.35034,-0.48584,-0.28564,-0.014648,0.2832,0.46021,0.53467,0.33691,0.05127,-0.2356,-0.45898,-0.43457,-0.21851,0.12451,0.43091,0.50903,0.5188,0.24902,-0.1355,-0.40039,-0.49072,-0.36621,-0.056152,0.23438,0.44189,0.49316,0.32227,0.057373,-0.25146,-0.48218,-0.41138,-0.15625,0.13306,0.41138,0.51392,0.4126,0.16113,-0.10986,-0.31616,-0.47363,-0.35645,-0.090332,0.24414,0.50537,0.55298,0.36621,0.087891,-0.23315,-0.45776,-0.4126,-0.26733,0.0097656,0.354,0.54443,0.43213,0.19043,-0.11719,-0.39917,-0.51025,-0.30518,-0.076904,0.17212,0.44556,0.47607,0.35889,0.070801,-0.2356,-0.48706,-0.48584,-0.22949,0.047607,0.37354,0.52002,0.44067,0.26001,0.0048828,-0.29541,-0.48828,-0.39917,-0.1416,0.19653,0.46753,0.55664,0.354,0.042725,-0.19531,-0.48706,-0.48584,-0.21729,0.0073242,0.40161,0.58716,0.51147,0.26367,0.0024414,-0.25391,-0.4541,-0.37109,-0.10742,0.14893,0.41748,0.56396,0.34912,0.12207,-0.1123,-0.47363,-0.47363,-0.19653,-0.029297,0.2417,0.53467,0.52368,0.26123,0.041504,-0.26855,-0.47241,-0.30273,-0.043945,0.23315,0.49927,0.56885,0.42969,0.14893,-0.10132,-0.36621,-0.46143,-0.20752,0.085449,0.37842,0.56519,0.57129,0.2356,0.092773,-0.177,-0.48096,-0.44434,-0.22827,0.075684,0.31128,0.48096,0.40039,0.064697,-0.12939,-0.3772,-0.51392,-0.27588,0.048828,0.36987,0.55298,0.48706,0.26489,0.0354,-0.24536,-0.47241,-0.35522,-0.20264,0.068359,0.33325,0.44678,0.4248,0.14404,-0.14893,-0.36621,-0.5481,-0.37964,-0.064697,0.22583,0.47119,0.50659,0.29053,-0.020752,-0.26367,-0.4541,-0.38574,-0.13062,0.18311,0.43457,0.52246,0.40405,0.20264,-0.11108,-0.34546,-0.43213,-0.30518,-0.042725,0.2002,0.47852,0.46875,0.33447,0.05127,-0.20264,-0.44189,-0.40039,-0.22949,0.10986,0.37842,0.57739,0.40039,0.14282,-0.15381,-0.37598,-0.47363,-0.30029,-0.021973,0.23804,0.53711,0.52734,0.38818,0.080566,-0.26001,-0.44678,-0.38574,-0.18799,0.053711,0.37476,0.44678,0.4126,0.19043,-0.081787,-0.35156,-0.5127,-0.38696,-0.12207,0.12207,0.4541,0.53345,0.36377,0.1001,-0.20508,-0.46753,-0.51147,-0.21484,0.087891,0.38574,0.54932,0.45654,0.16113,-0.11841,-0.35278,-0.47241,-0.34058,-0.15381,0.12207,0.44189,0.50415,0.37109,0.10376,-0.19897,-0.46021,-0.48828,-0.24536,-0.0048828,0.2832,0.52368,0.46021,0.1709,-0.1001,-0.36499,-0.59082,-0.38086,-0.073242,0.22705,0.45898,0.54932,0.43457,0.177,-0.1416,-0.36133,-0.45044,-0.23315,0.085449,0.40039,0.53223,0.48706,0.25757,-0.057373,-0.33325,-0.5188,-0.42969,-0.20752,0.087891,0.36987,0.53955,0.41138,0.096436,-0.21606,-0.38452,-0.4834,-0.26489,0.026855,0.27222,0.47852,0.49316,0.28809,-0.043945,-0.31006,-0.52002,-0.4248,-0.1709,0.17578,0.44556,0.5127,0.45532,0.20874,-0.074463,-0.34668,-0.4126,-0.25635,0.036621,0.3186,0.50049,0.45532,0.24902,0.0024414,-0.27466,-0.49683,-0.36865,-0.15381,0.14038,0.47852,0.56274,0.40771,0.13184,-0.18066,-0.39429,-0.46387,-0.26978,-0.068359,0.23438,0.49683,0.4895,0.29175,0.0085449,-0.30518,-0.53101,-0.43213,-0.22339,0.063477,0.37598,0.53223,0.39185,0.18188,-0.10986,-0.39917,-0.47241,-0.271,-0.047607,0.20874,0.50537,0.50903,0.29419,0.040283,-0.28809,-0.5188,-0.43457,-0.23804,0.058594,0.39185,0.49316,0.42725,0.15747,-0.1123,-0.34668,-0.46875,-0.34424,-0.026855,0.24414,0.4895,0.53223,0.30151,0.037842,-0.23682,-0.49683,-0.49072,-0.18433,0.050049,0.33936,0.54321,0.45532,0.1709,-0.070801,-0.32227,-0.52246,-0.31372,-0.046387,0.18066,0.47607,0.55786,0.37476,0.080566,-0.23926,-0.46997,-0.49683,-0.23071,0.050049,0.31128,0.50659,0.46997,0.2417,-0.06958,-0.28442,-0.47974,-0.34302,-0.097656,0.19897,0.44922,0.50781,0.32959,0.11841,-0.17334,-0.47241,-0.54077,-0.30029,0.0048828,0.33081,0.50903,0.51758,0.2478,-0.08667,-0.26611,-0.46997,-0.38818,-0.1123,0.20264,0.41016,0.57007,0.43091,0.078125,-0.22705,-0.39307,-0.44556,-0.23926,0.05127,0.30518,0.51758,0.46631,0.30273,-0.020752,-0.30029,-0.4834,-0.36621,-0.14038,0.12329,0.42114,0.48706,0.41138,0.11963,-0.10254,-0.46997,-0.49194,-0.30273,0.042725,0.31494,0.50293,0.46875,0.29053,-0.029297,-0.27344,-0.4187,-0.35767,-0.12939,0.19897,0.52856,0.55176,0.43213,0.16357,-0.16479,-0.40039,-0.47241,-0.31494,-0.05127,0.25635,0.49805,0.53955,0.33447,0.083008,-0.25146,-0.46753,-0.32715,-0.098877,0.03418,0.44434,0.52124,0.41992,0.16113,-0.048828,-0.36377,-0.48462,-0.271,0.039063,0.30518,0.50049,0.51758,0.32227,0.10986,-0.21729,-0.52002,-0.36987,-0.2002,0.097656,0.45532,0.52002,0.41016,0.19043,-0.073242,-0.33447,-0.4834,-0.29175,-0.054932,0.18188,0.48828,0.56519,0.28931,0.047607,-0.23926,-0.49316,-0.4187,-0.18677,0.073242,0.34912,0.53467,0.42847,0.14038,-0.14893,-0.33936,-0.50171,-0.36011,-0.10254,0.13306,0.44067,0.47852,0.3064,0.0354,-0.25879,-0.52856,-0.4895,-0.24414,0.053711,0.30884,0.50171,0.41138,0.18311,-0.083008,-0.30762,-0.50537,-0.36865,-0.063477,0.17212,0.42847,0.50293,0.35767,0.018311,-0.26123,-0.46509,-0.48096,-0.27466,0.070801,0.37598,0.47119,0.48828,0.2063,-0.021973,-0.33081,-0.45898,-0.40771,-0.073242,0.087891,0.46143,0.49438,0.3418,0.0097656,-0.13672,-0.45654,-0.49805,-0.23193,0.050049,0.31738,0.52856,0.49072,0.2417,-0.065918,-0.32715,-0.47852,-0.39307,-0.13672,0.177,0.46265,0.46387,0.38574,0.15137,-0.18066,-0.39063,-0.44067,-0.32227,-0.025635,0.33325,0.47607,0.45166,0.26489,-0.061035,-0.31006,-0.47241,-0.37109,-0.092773,0.20874,0.48096,0.56641,0.39551,0.10498,-0.17334,-0.42236,-0.46875,-0.26733,-0.018311,0.35278,0.55664,0.53223,0.38696,0.070801,-0.26855,-0.41504,-0.30518,-0.11841,0.070801,0.45898,0.45288,0.35278,0.13428,-0.11475,-0.46753,-0.448,-0.21973,0.024414,0.32471,0.57251,0.5542,0.34668,0.064697,-0.22217,-0.48584,-0.36865,-0.17456,0.083008,0.42847,0.52368,0.37842,0.12451,-0.15137,-0.39551,-0.4834,-0.33569,-0.079346,0.21729,0.52246,0.50415,0.27588,0.028076,-0.177,-0.44434,-0.354,-0.14893,0.10742,0.41992,0.59082,0.3479,0.097656,-0.063477,-0.42847,-0.54443,-0.3064,-0.041504,0.1709,0.49805,0.5542,0.32227,0.053711,-0.20874,-0.46753,-0.4187,-0.177,0.083008,0.30273,0.48218,0.39307,0.1709,-0.10742,-0.36499,-0.51514,-0.35645,-0.050049,0.2478,0.50415,0.55664,0.35645,0.065918,-0.177,-0.43457,-0.46631,-0.20386,0.097656,0.4126,0.56396,0.5127,0.20142,-0.085449,-0.29297,-0.49805,-0.39795,-0.083008,0.177,0.4126,0.53711,0.37598,0.023193,-0.22583,-0.42969,-0.47363,-0.22827,0.048828,0.33325,0.49805,0.43823,0.17212,-0.12085,-0.37964,-0.52856,-0.4126,-0.13916,0.15991,0.45166,0.51758,0.41016,0.087891,-0.21484,-0.40527,-0.44678,-0.22705,0.026855,0.27832,0.46143,0.45654,0.23193,-0.08667,-0.33081,-0.50537,-0.41992,-0.16602,0.16968,0.43945,0.51514,0.38452,0.092773,-0.13184,-0.40771,-0.38818,-0.19653,-0.03418,0.36743,0.54688,0.44556,0.26489,-0.059814,-0.36377,-0.45288,-0.35156,-0.13306,0.177,0.42603,0.54199,0.41138,0.14404,-0.12939,-0.34912,-0.42114,-0.20508,0.083008,0.34912,0.54932,0.54688,0.36133,0.03418,-0.2832,-0.5249,-0.36865,-0.12573,0.15869,0.48584,0.51392,0.39185,0.19165,-0.10376,-0.44434,-0.4834,-0.28198,-0.01709,0.27344,0.53345,0.48096,0.27344,0.0073242,-0.24658,-0.48096,-0.41382,-0.15137,0.1355,0.42236,0.59326,0.47729,0.17334,-0.10254,-0.35278,-0.46387,-0.29175,-0.032959,0.22583,0.55786,0.5835,0.35034,0.098877,-0.24902,-0.50049,-0.37354,-0.092773,0.074463,0.35278,0.5127,0.42236,0.15747,-0.11353,-0.36133,-0.49438,-0.22583,0.024414,0.23804,0.47485,0.51758,0.32227,0.031738,-0.2417,-0.48706,-0.50781,-0.2771,0.05127,0.47607,0.48584,0.4541,0.29175,0.0048828,-0.2417,-0.42725,-0.3064,0.013428,0.30273,0.48218,0.52979,0.28564,0.075684,-0.16968,-0.39917,-0.39185,-0.1355,0.13794,0.41016,0.50293,0.46753,0.18188,-0.14648,-0.33447,-0.35645,-0.29907,0.019531,0.27222,0.42358,0.49438,0.41748,0.063477,-0.18433,-0.52368,-0.46509,-0.21729,0.091553,0.36621,0.54199,0.49805,0.2832,-0.040283,-0.2771,-0.43213,-0.29541,0.012207,0.32471,0.45654,0.5188,0.38208,0.10376,-0.18799,-0.4187,-0.50171,-0.29785,-0.031738,0.35034,0.48096,0.41626,0.2478,-0.061035,-0.354,-0.43091,-0.36133,-0.16479,0.20508,0.47607,0.56519,0.42603,0.10498,-0.20996,-0.40405,-0.43213,-0.22705,0.0012207,0.271,0.48584,0.4248,0.21729,-0.032959,-0.34912,-0.52246,-0.36377,-0.16846,0.12329,0.45166,0.52002,0.41626,0.19653,-0.14648,-0.47119,-0.53223,-0.33447,-0.057373,0.28809,0.51392,0.44434,0.22705,0.064697,-0.22827,-0.45898,-0.33081,-0.062256,0.12695,0.43213,0.54443,0.36865,0.10742,-0.090332,-0.34912,-0.45532,-0.31738,-0.063477,0.21973,0.52124,0.52856,0.28564,-0.0048828,-0.29053,-0.51636,-0.40527,-0.16846,0.092773,0.36743,0.54932,0.44922,0.15381,-0.177,-0.45044,-0.53223,-0.22705,-0.03418,0.23315,0.44434,0.47363,0.26001,-0.0097656,-0.25391,-0.50293,-0.4541,-0.19653,0.065918,0.37842,0.51758,0.45532,0.16479,-0.083008,-0.33936,-0.56519,-0.41504,-0.093994,0.20508,0.43945,0.49072,0.27954,-0.020752,-0.26367,-0.46265,-0.44922,-0.22461,0.092773,0.38574,0.52734,0.46265,0.17578,-0.092773,-0.29053,-0.43091,-0.3186,-0.090332,0.16235,0.46265,0.50781,0.39429,0.087891,-0.25146,-0.47607,-0.47607,-0.24902,0.10742,0.34424,0.51758,0.52246,0.26611,-0.079346,-0.32471,-0.48584,-0.3418,-0.073242,0.21362,0.43701,0.41626,0.28198,0.0097656,-0.21118,-0.41992,-0.41504,-0.22095,0.093994,0.44678,0.47852,0.45532,0.25757,-0.024414,-0.28198,-0.49072,-0.34912,-0.11841,0.096436,0.51392,0.4895,0.32593,0.0354,-0.17822,-0.47729,-0.40771,-0.15015,0.030518,0.35645,0.52368,0.448,0.21973,-0.073242,-0.35034,-0.46387,-0.28198,-0.037842,0.075684,0.4895,0.4895,0.3479,0.13794,-0.17456,-0.50049,-0.53955,-0.29907,-0.058594,0.26123,0.5188,0.44922,0.23926,-0.039063,-0.32227,-0.54199,-0.34058,-0.14526,0.14038,0.5127,0.49438,0.28687,0.095215,-0.13672,-0.3772,-0.46387,-0.27588,-0.014648,0.25024,0.51025,0.47485,0.18066,-0.058594,-0.28564,-0.53589,-0.4248,-0.20264,0.05127,0.36255,0.5896,0.47363,0.15137,-0.15747,-0.47241,-0.56519,-0.31982,0.043945,0.26245,0.50659,0.54932,0.32471,0.039063,-0.21729,-0.47119,-0.41748,-0.12207,0.15381,0.4126,0.47363,0.41626,0.13794,-0.10254,-0.354,-0.51758,-0.39551,-0.048828,0.21973,0.45044,0.52979,0.29785,0.06958,-0.16479,-0.41992,-0.38208,-0.11475,0.15625,0.44678,0.50049,0.43701,0.1001,-0.17334,-0.4248,-0.47607,-0.32349,0.029297,0.28442,0.41138,0.51025,0.35278,0.024414,-0.2832,-0.44067,-0.43457,-0.23926,0.081787,0.33447,0.47607,0.44067,0.2002,-0.13794,-0.34546,-0.51392,-0.40527,-0.063477,0.26245,0.49561,0.54565,0.38574,0.089111,-0.19775,-0.44189,-0.42236,-0.19653,0.13184,0.50415,0.5542,0.46509,0.28198,-0.048828,-0.33325,-0.46387,-0.36255,-0.15747,0.19653,0.4541,0.49683,0.37598,0.087891,-0.22949,-0.41382,-0.40161,-0.21484,0.076904,0.39673,0.5835,0.50415,0.26001,-0.087891,-0.37231,-0.52246,-0.36011,-0.11963,0.14771,0.48218,0.50781,0.36621,0.17456,-0.12207,-0.44189,-0.43945,-0.18921,0.047607,0.33936,0.53711,0.46021,0.26855,-0.0073242,-0.31616,-0.49927,-0.35278,-0.072021,0.22705,0.43213,0.54443,0.36743,0.068359,-0.16113,-0.43335,-0.49561,-0.22827,-0.013428,0.31738,0.50781,0.46631,0.20264,0.0061035,-0.29785,-0.48706,-0.36621,-0.080566,0.18433,0.43457,0.48584,0.39551,0.12207,-0.054932,-0.40894,-0.48584,-0.271,0.01709,0.28687,0.48584,0.5188,0.27222,-0.048828,-0.28687,-0.5481,-0.47241,-0.17212,0.12207,0.39307,0.52368,0.41504,0.12451,-0.13916,-0.34424,-0.49927,-0.33691,0.018311,0.26489,0.5127,0.52612,0.28564,-0.012207,-0.24902,-0.45776,-0.39917,-0.18921,0.12939,0.41992,0.49805,0.43457,0.13916,-0.21851,-0.3894,-0.48218,-0.27832,0.061035,0.32471,0.53589,0.50903,0.33813,-0.0073242,-0.31372,-0.5127,-0.43091,-0.26611,0.080566,0.30396,0.43457,0.40649,0.15869,-0.19287,-0.40283,-0.45166,-0.30029,0.0097656,0.30762,0.50537,0.51758,0.33569,0.063477,-0.24902,-0.49927,-0.4126,-0.24658,0.073242,0.44678,0.52612,0.48706,0.27588,-0.062256,-0.41504,-0.45532,-0.38574,-0.10742,0.19287,0.45776,0.48218,0.28076,0.0073242,-0.32471,-0.53345,-0.45776,-0.26855,0.014648,0.33569,0.44312,0.36987,0.18433,-0.11475,-0.38574,-0.51636,-0.37598,-0.12695,0.14771,0.46509,0.50293,0.33447,0.095215,-0.18555,-0.54077,-0.52856,-0.2771,-0.037842,0.29053,0.50415,0.4126,0.18555,-0.095215,-0.35278,-0.48584,-0.36987,-0.097656,0.15381,0.44434,0.52002,0.34912,0.050049,-0.23682,-0.50293,-0.53589,-0.28564,-0.05249,0.27832,0.46021,0.43945,0.17578,-0.070801,-0.33691,-0.56763,-0.43823,-0.13184,0.10254,0.3418,0.55176,0.36865,0.018311,-0.22705,-0.46143,-0.53467,-0.18799,-0.01709,0.26245,0.43213,0.43213,0.22095,-0.1001,-0.32593,-0.50293,-0.38086,-0.090332,0.2002,0.48096,0.54199,0.38086,0.12085,-0.067139,-0.42603,-0.49194,-0.24536,-0.05127,0.23315,0.42847,0.43091,0.23193,-0.13672,-0.32471,-0.48096,-0.44434,-0.18066,0.18188,0.38696,0.53955,0.4541,0.10986,-0.22461,-0.3772,-0.47974,-0.39185,-0.063477,0.22339,0.45532,0.48828,0.30762,-0.0354,-0.30762,-0.53223,-0.45898,-0.21362,0.057373,0.31982,0.48828,0.4248,0.16602,-0.13062,-0.41016,-0.49438,-0.34424,-0.018311,0.29541,0.45898,0.43945,0.29053,0.014648,-0.27222,-0.45776,-0.4187,-0.25757,0.080566,0.47241,0.52979,0.47363,0.23071,-0.073242,-0.34302,-0.43579,-0.33691,-0.084229,0.21973,0.52856,0.5542,0.32349,0.045166,-0.26123,-0.45654,-0.39673,-0.18066,0.085449,0.3772,0.53833,0.46265,0.19775,-0.0036621,-0.30151,-0.45288,-0.28564,-0.028076,0.24414,0.49683,0.55176,0.40649,0.15503,-0.18799,-0.49805,-0.46509,-0.18066,0.08667,0.40039,0.60791,0.39551,0.29053,0.061035,-0.2356,-0.41504,-0.271,-0.023193,0.28809,0.57251,0.61035,0.40527,0.075684,-0.20874,-0.43457,-0.46875,-0.20508,0.068359,0.36621,0.59937,0.56152,0.27466,-0.031738,-0.28442,-0.49072,-0.37109,-0.076904,0.14893,0.39063,0.51147,0.36743,0.087891,-0.19165,-0.47729,-0.53955,-0.20386,0.085449,0.36377,0.56519,0.50781,0.29541,0.01709,-0.23438,-0.46509,-0.36133,-0.064697,0.21729,0.47852,0.57373,0.47119,0.12695,-0.14038,-0.36743,-0.46387,-0.28564,0.098877,0.35278,0.57617,0.55298,0.33203,-0.0036621,-0.22705,-0.40771,-0.29907,-0.14404,0.15869,0.43457,0.53345,0.40771,0.072021,-0.15137,-0.448,-0.50537,-0.27344,0.080566,0.37109,0.51514,0.55786,0.33447,0.014648,-0.27344,-0.44434,-0.3894,-0.1355,0.16357,0.40527,0.5249,0.45044,0.19653,-0.16113,-0.36133,-0.42847,-0.31372,0.014648,0.33447,0.51392,0.49438,0.33447,0.029297,-0.29053,-0.47363,-0.42847,-0.19287,0.10498,0.47241,0.52734,0.4248,0.12573,-0.16968,-0.41138,-0.53589,-0.37598,-0.091553,0.16846,0.44189,0.47974,0.24902,0.058594,-0.26611,-0.46875,-0.39673,-0.18311,0.1123,0.48462,0.50537,0.39795,0.15259,-0.19775,-0.46997,-0.54199,-0.34302,-0.1001,0.2124,0.50659,0.5188,0.31006,0.096436,-0.23315,-0.53223,-0.44312,-0.18921,0.096436,0.33813,0.47852,0.41016,0.20264,-0.098877,-0.34424,-0.53223,-0.38818,-0.083008,0.24658,0.51025,0.55176,0.36011,0.087891,-0.21973,-0.46631,-0.4541,-0.22705,0.056152,0.39673,0.57373,0.46875,0.14526,-0.14648,-0.38452,-0.57007,-0.3833,-0.1123,0.12695,0.40527,0.53955,0.35522,0.021973,-0.22949,-0.49316,-0.51147,-0.26001,0.0061035,0.38574,0.43213,0.49316,0.28931,-0.073242,-0.32715,-0.55908,-0.50537,-0.15381,0.15869,0.45898,0.53467,0.4248,0.10498,-0.12695,-0.4126,-0.48706,-0.19775,0.12573,0.38086,0.55786,0.52368,0.24414,-0.05127,-0.25757,-0.44922,-0.38696,-0.1062,0.13916,0.42847,0.53467,0.42847,0.073242,-0.23315,-0.46387,-0.51636,-0.36255,-0.076904,0.29785,0.42847,0.448,0.2478,-0.1355,-0.43335,-0.53833,-0.49561,-0.21973,0.091553,0.33203,0.46753,0.40527,0.16113,-0.18066,-0.45776,-0.50781,-0.32471,-0.020752,0.31738,0.45288,0.46387,0.28076,-0.053711,-0.32959,-0.53101,-0.48828,-0.24658,0.090332,0.39795,0.45532,0.37109,0.12451,-0.20386,-0.46143,-0.53833,-0.3418,-0.079346,0.24902,0.52124,0.46143,0.24414,0.025635,-0.32959,-0.52856,-0.40894,-0.23193,0.03418,0.35889,0.48584,0.35278,0.11353,-0.19897,-0.46631,-0.54565,-0.35889,-0.074463,0.22461,0.46021,0.47485,0.31738,0.018311,-0.33203,-0.55176,-0.47852,-0.27466,0.041504,0.29785,0.47852,0.3479,0.20752,-0.057373,-0.36255,-0.52734,-0.25879,0.0097656,0.23926,0.5603,0.47607,0.22827,0.031738,-0.2417,-0.52612,-0.47852,-0.24658,0.047607,0.40771,0.54199,0.4834,0.19409,-0.1062,-0.33203,-0.50903,-0.31494,-0.05249,0.2063,0.4834,0.56152,0.36987,0.065918,-0.23193,-0.46509,-0.42358,-0.15869,0.10254,0.37598,0.57983,0.46997,0.20264,-0.068359,-0.26733,-0.46387,-0.30762,0.01709,0.20142,0.51514,0.62378,0.46631,0.10986,-0.13672,-0.41748,-0.52124,-0.26611,0.05127,0.29175,0.47119,0.42603,0.1709,-0.093994,-0.31738,-0.50537,-0.39063,-0.15381,0.16113,0.45654,0.49805,0.43213,0.12695,-0.20264,-0.44189,-0.4895,-0.27222,-0.0036621,0.34668,0.45898,0.49316,0.28809,-0.05249,-0.31982,-0.5249,-0.45044,-0.092773,0.093994,0.31738,0.46875,0.39673,0.084229,-0.26123,-0.46143,-0.53223,-0.32715,-0.037842,0.27832,0.4187,0.36987,0.17456,-0.11963,-0.41016,-0.56152,-0.41748,-0.14893,0.059814,0.4834,0.4541,0.3418,0.11353,-0.18799,-0.43701,-0.54688,-0.28809,-0.096436,0.20508,0.53223,0.44312,0.22827,0.03418,-0.28198,-0.48828,-0.36987,-0.11963,0.18311,0.50903,0.48828,0.33203,0.1123,-0.096436,-0.41748,-0.39429,-0.31006,-0.070801,0.23071,0.52002,0.52856,0.30518,0.0085449,-0.25269,-0.50659,-0.3894,-0.14648,0.10376,0.37842,0.58472,0.46753,0.20508,-0.06958,-0.35278,-0.47363,-0.27222,0.019531,0.25635,0.5249,0.50537,0.27588,0.039063,-0.23926,-0.49194,-0.42725,-0.18066,0.089111,0.46997,0.57983,0.48706,0.2417,-0.024414,-0.29297,-0.45776,-0.33691,-0.063477,0.20264,0.46875,0.57007,0.32227,-0.021973,-0.24292,-0.46387,-0.48828,-0.16602,0.068359,0.34912,0.53833,0.47974,0.15869,-0.13916,-0.34424,-0.45166,-0.28076,0.025635,0.24536,0.49683,0.57129,0.43457,0.12817,-0.16113,-0.4834,-0.46509,-0.18433,0.11841,0.39551,0.61523,0.43823,0.19409,-0.085449,-0.32593,-0.54565,-0.3894,-0.062256,0.23682,0.50049,0.52979,0.35522,0.010986,-0.25879,-0.44312,-0.49316,-0.29297,0.010986,0.33936,0.43457,0.4187,0.32471,-0.036621,-0.2771,-0.38818,-0.32593,-0.093994,0.20752,0.45166,0.58105,0.46997,0.13184,-0.16846,-0.43335,-0.4834,-0.18555,0.12207,0.40771,0.58594,0.44678,0.31494,0.0048828,-0.28564,-0.4541,-0.35156,-0.068359,0.27588,0.5481,0.56396,0.4895,0.21362,-0.14038,-0.41016,-0.47363,-0.28687,-0.0024414,0.33691,0.57983,0.52002,0.32715,0.063477,-0.28442,-0.46265,-0.32471,-0.068359,0.22461,0.53345,0.61279,0.4541,0.16357,-0.13672,-0.42603,-0.47607,-0.23804,0.043945,0.37109,0.49927,0.44922,0.27954,0,-0.29785,-0.47119,-0.38696,-0.20386,0.1001,0.35522,0.50903,0.40161,0.11719,-0.177,-0.44189,-0.55786,-0.31372,-0.024414,0.27466,0.58228,0.58228,0.3186,0.045166,-0.25757,-0.4895,-0.36621,-0.12817,0.15259,0.45532,0.53589,0.44189,0.16235,-0.1355,-0.38086,-0.53955,-0.3894,-0.087891,0.17578,0.43457,0.49438,0.27832,-0.015869,-0.25146,-0.50537,-0.47363,-0.19531,0.11963,0.39185,0.54565,0.42847,0.10864,-0.15137,-0.37964,-0.54443,-0.37598,-0.059814,0.18555,0.45898,0.50415,0.29785,-0.020752,-0.28198,-0.52002,-0.50781,-0.16113,0.057373,0.354,0.51636,0.47241,0.20996,-0.096436,-0.36499,-0.5481,-0.4187,-0.11108,0.20996,0.45044,0.50537,0.36621,0.073242,-0.24902,-0.46631,-0.3894,-0.16113,0.065918,0.40039,0.47729,0.4541,0.19287,-0.13916,-0.34058,-0.49316,-0.41382,-0.16968,0.13428,0.38574,0.4541,0.36621,0.031738,-0.25269,-0.45532,-0.44189,-0.18311,0.16113,0.4834,0.60547,0.46143,0.21973,-0.0012207,-0.42358,-0.52124,-0.37598,-0.11475,0.20996,0.54565,0.53711,0.42969,0.17212,-0.14893,-0.38086,-0.34668,-0.18433,0.083008,0.4126,0.54199,0.49561,0.28931,-0.048828,-0.32471,-0.50049,-0.36987,-0.15137,0.12451,0.44189,0.51392,0.38086,0.11475,-0.19409,-0.46265,-0.42725,-0.24902,0.0073242,0.39307,0.60669,0.50293,0.28931,0.013428,-0.29663,-0.4895,-0.38574,-0.13428,0.10132,0.39063,0.56641,0.39429,0.11353,-0.11108,-0.40649,-0.52002,-0.24414,0,0.30029,0.58105,0.54565,0.29053,0.014648,-0.29907,-0.53711,-0.40894,-0.18433,0.058594,0.3894,0.47607,0.37842,0.13916,-0.15137,-0.41992,-0.49805,-0.30518,-0.0061035,0.24048,0.49438,0.57373,0.32837,0.012207,-0.21729,-0.4834,-0.45166,-0.14038,0.13916,0.36377,0.54199,0.4248,0.10376,-0.12695,-0.32715,-0.47974,-0.30273,0.0354,0.24902,0.56641,0.5957,0.34302,0.075684,-0.20508,-0.44678,-0.41382,-0.2002,0.037842,0.33691,0.49072,0.40161,0.14282,-0.12695,-0.35645,-0.50171,-0.36255,-0.039063,0.25879,0.4541,0.55176,0.36621,0.0085449,-0.27588,-0.47852,-0.53711,-0.30029,0.03418,0.41748,0.52612,0.51392,0.23193,-0.084229,-0.3064,-0.46631,-0.34058,-0.041504,0.23315,0.47363,0.5542,0.39063,0.10376,-0.21118,-0.45166,-0.40527,-0.26611,0.026855,0.31738,0.46143,0.42725,0.20386,-0.11597,-0.39307,-0.50293,-0.40283,-0.15747,0.21973,0.46021,0.48462,0.40039,0.067139,-0.25879,-0.4541,-0.50415,-0.33813,-0.01709,0.27222,0.46509,0.40894,0.18555,-0.092773,-0.36133,-0.54565,-0.38208,-0.15137,0.13672,0.46143,0.49561,0.33081,0.083008,-0.21484,-0.49561,-0.46387,-0.2478,-0.019531,0.29907,0.48462,0.41748,0.22461,-0.065918,-0.3833,-0.56519,-0.41504,-0.15991,0.13306,0.44922,0.5127,0.40039,0.12817,-0.18311,-0.4248,-0.49683,-0.29053,-0.036621,0.28931,0.51758,0.44434,0.17456,-0.068359,-0.33813,-0.55176,-0.33936,-0.15991,0.070801,0.43701,0.53955,0.39185,0.12695,-0.15015,-0.39917,-0.5127,-0.32715,-0.053711,0.22705,0.47852,0.47729,0.22705,-0.067139,-0.31128,-0.53223,-0.41748,-0.15625,0.096436,0.41016,0.5481,0.39185,0.13428,-0.1416,-0.40161,-0.55054,-0.31616,-0.0061035,0.21484,0.48828,0.53345,0.24902,-0.057373,-0.27832,-0.52734,-0.47852,-0.21362,0.076904,0.31128,0.4834,0.42969,0.13428,-0.18188,-0.35278,-0.51758,-0.27588,-0.040283,0.20996,0.46143,0.50903,0.25146,-0.024414,-0.29419,-0.54443,-0.4834,-0.22461,0.05127,0.40039,0.50781,0.47852,0.19531,-0.11353,-0.30151,-0.48462,-0.3418,-0.020752,0.24658,0.46997,0.52734,0.32715,0.031738,-0.24658,-0.46631,-0.4248,-0.21973,0.12939,0.42114,0.54443,0.50049,0.25757,-0.10132,-0.33325,-0.45898,-0.36621,-0.047607,0.23193,0.4834,0.52002,0.36133,0.047607,-0.24658,-0.4895,-0.48462,-0.271,0.03418,0.40161,0.49072,0.42236,0.29785,-0.032959,-0.27344,-0.43457,-0.28687,0.0024414,0.26855,0.54565,0.5127,0.33813,0.031738,-0.1709,-0.49438,-0.39429,-0.19287,-0.032959,0.3772,0.52979,0.42725,0.30396,-0.0073242,-0.32715,-0.45288,-0.29297,-0.067139,0.22339,0.51392,0.54688,0.39307,0.079346,-0.2002,-0.49194,-0.53711,-0.25024,-0.0097656,0.30273,0.46143,0.42114,0.21606,-0.046387,-0.33081,-0.53711,-0.35645,-0.14893,0.12329,0.56763,0.49805,0.32471,0.1416,-0.12451,-0.43945,-0.51025,-0.28198,0.01709,0.28809,0.56396,0.54565,0.26001,-0.029297,-0.24048,-0.46509,-0.3418,-0.061035,0.16113,0.43823,0.61035,0.47974,0.1416,-0.2002,-0.46265,-0.57495,-0.31372,0.01709,0.32227,0.54199,0.47363,0.25513,-0.0354,-0.30396,-0.51758,-0.44678,-0.1416,0.12573,0.3479,0.50049,0.36499,0.1001,-0.17578,-0.36865,-0.52002,-0.33813,-0.053711,0.27588,0.47241,0.51025,0.3064,-0.054932,-0.25146,-0.46021,-0.45288,-0.2356,0.12695,0.43945,0.52368,0.46875,0.15869,-0.19043,-0.38574,-0.48828,-0.36865,-0.078125,0.20386,0.44189,0.48706,0.29907,-0.031738,-0.30762,-0.49683,-0.42847,-0.19897,0.10864,0.37231,0.50903,0.47363,0.20874,-0.14282,-0.3772,-0.50171,-0.37476,-0.098877,0.20264,0.4895,0.46509,0.33936,0.074463,-0.24414,-0.48096,-0.43213,-0.27832,0.079346,0.42358,0.52124,0.40894,0.17578,-0.11475,-0.35645,-0.50293,-0.3772,-0.12207,0.18066,0.43335,0.46021,0.27832,0.11841,-0.17822,-0.43457,-0.40283,-0.15991,0.078125,0.47119,0.53101,0.49561,0.22461,-0.0024414,-0.44189,-0.49927,-0.32959,-0.06958,0.19043,0.47485,0.55664,0.42114,0.15259,-0.19409,-0.44922,-0.41992,-0.1355,0.010986,0.42114,0.49072,0.40527,0.21362,0.018311,-0.29053,-0.53589,-0.38574,-0.089111,0.22705,0.49438,0.57617,0.31738,0.14771,-0.11475,-0.43213,-0.49561,-0.19531,0.092773,0.36865,0.60059,0.52002,0.25879,-0.029297,-0.25391,-0.43945,-0.33081,-0.083008,0.177,0.46753,0.62378,0.38452,0.043945,-0.20996,-0.44678,-0.5127,-0.26611,-0.024414,0.26855,0.50903,0.54199,0.32471,-0.0036621,-0.30029,-0.55664,-0.45166,-0.13428,0.13916,0.34668,0.45532,0.34424,0.096436,-0.18555,-0.41626,-0.54077,-0.34668,0.01709,0.33447,0.49438,0.47974,0.26123,-0.042725,-0.27832,-0.44922,-0.39185,-0.17822,0.048828,0.46997,0.49561,0.4248,0.097656,-0.22949,-0.40527,-0.48096,-0.33325,-0.01709,0.23193,0.48706,0.55664,0.32715,-0.026855,-0.25635,-0.4834,-0.39551,-0.18799,0.083008,0.36987,0.44922,0.35156,0.12817,-0.1709,-0.4541,-0.47607,-0.31006,0.0073242,0.32959,0.55664,0.45776,0.31006,0.14893,-0.19897,-0.51147,-0.4126,-0.24658,0.070801,0.37231,0.48462,0.40771,0.15381,-0.15869,-0.38086,-0.48706,-0.34424,-0.056152,0.2417,0.47852,0.49805,0.29053,0.012207,-0.2124,-0.4834,-0.3894,-0.15137,-0.024414,0.38574,0.53223,0.40527,0.13672,-0.040283,-0.46997,-0.54199,-0.29053,-0.063477,0.13306,0.44556,0.50781,0.32349,0.070801,-0.271,-0.50903,-0.47241,-0.21118,0.059814,0.35767,0.47607,0.40283,0.18921,-0.08667,-0.36987,-0.55176,-0.4248,-0.14282,0.16846,0.45166,0.54077,0.32959,0.056152,-0.1062,-0.47852,-0.44556,-0.15503,0.093994,0.37109,0.53467,0.4126,0.15747,-0.11841,-0.34546,-0.53101,-0.36865,-0.11963,0.15381,0.45654,0.58105,0.41138,0.070801,-0.18066,-0.43213,-0.47363,-0.16113,-0.0036621,0.30273,0.49561,0.45532,0.24414,-0.085449,-0.33936,-0.50171,-0.41382,-0.12573,0.16968,0.38696,0.55298,0.43701,0.087891,-0.16602,-0.41504,-0.50781,-0.26733,0.048828,0.34424,0.50537,0.47363,0.23438,-0.063477,-0.28809,-0.49194,-0.45898,-0.17334,0.13428,0.43457,0.52246,0.45898,0.10864,-0.17822,-0.38574,-0.46997,-0.31738,-0.03418,0.29785,0.52368,0.54199,0.32349,-0.026855,-0.33325,-0.47363,-0.41138,-0.16846,0.13672,0.38086,0.49316,0.43457,0.15259,-0.18921,-0.41016,-0.49316,-0.32227,-0.030518,0.27222,0.46753,0.44922,0.28076,-0.029297,-0.32593,-0.4895,-0.39673,-0.28564,0.054932,0.35767,0.48828,0.41504,0.16724,-0.12939,-0.35889,-0.4834,-0.31372,-0.05249,0.28076,0.54565,0.52246,0.26611,0.0048828,-0.33325,-0.53589,-0.43091,-0.22705,0.054932,0.43457,0.53955,0.44678,0.21973,-0.11108,-0.36133,-0.46387,-0.20264,-0.081787,0.19531,0.45898,0.46631,0.29785,0.037842,-0.22461,-0.51758,-0.41382,-0.2356,0.065918,0.38574,0.54077,0.44067,0.17456,-0.10132,-0.33691,-0.52368,-0.34302,-0.050049,0.18677,0.4895,0.50659,0.30762,0.0354,-0.22705,-0.48706,-0.50415,-0.23804,0.010986,0.27954,0.53955,0.44067,0.16602,-0.10376,-0.33447,-0.4834,-0.27954,-0.013428,0.15869,0.55542,0.53345,0.3479,0.12451,-0.20874,-0.44678,-0.45898,-0.21606,0.0048828,0.40039,0.47119,0.44434,0.20264,-0.1001,-0.31372,-0.52979,-0.33936,0.045166,0.22583,0.4834,0.51758,0.38086,0.048828,-0.13916,-0.43335,-0.50415,-0.2063,0.12939,0.41016,0.47607,0.46509,0.23682,-0.092773,-0.34424,-0.51392,-0.37598,-0.16479,0.14404,0.45898,0.5188,0.40039,0.045166,-0.15015,-0.43457,-0.48706,-0.26123,0.037842,0.31982,0.4541,0.46021,0.22705,-0.093994,-0.32471,-0.46387,-0.40527,-0.13672,0.1416,0.43701,0.4834,0.41016,0.10254,-0.24414,-0.46387,-0.51758,-0.33691,-0.025635,0.30029,0.49561,0.4834,0.29541,-0.01709,-0.29541,-0.50903,-0.42236,-0.18066,0.12695,0.40649,0.4834,0.38696,0.13428,-0.16968,-0.39307,-0.5249,-0.34302,-0.092773,0.28076,0.49072,0.50415,0.25391,-0.0085449,-0.32227,-0.49316,-0.40527,-0.21851,0.073242,0.44189,0.55054,0.45166,0.17822,-0.1709,-0.42847,-0.51392,-0.26367,-0.11108,0.16235,0.52246,0.45532,0.26367,0.10864,-0.22095,-0.53467,-0.36621,-0.1062,0.10376,0.49561,0.50537,0.36133,0.13916,-0.015869,-0.37842,-0.52246,-0.36133,-0.013428,0.14893,0.4834,0.50537,0.26489,0.015869,-0.16968,-0.5249,-0.40527,-0.17334,-0.023193,0.31128,0.54443,0.45044,0.16357,0.0024414,-0.33325,-0.50293,-0.25635,0.01709,0.25024,0.52612,0.50293,0.31006,0.10254,-0.17456,-0.448,-0.44922,-0.15625,0.15503,0.42603,0.57129,0.50049,0.2002,-0.0024414,-0.3064,-0.4541,-0.35645,-0.12695,0.12817,0.38696,0.49316,0.29907,-0.014648,-0.26245,-0.47729,-0.50903,-0.18311,0.12207,0.43091,0.60303,0.53345,0.26978,-0.0012207,-0.2478,-0.46509,-0.38452,-0.097656,0.2417,0.47852,0.54443,0.36621,0.053711,-0.14648,-0.43213,-0.45654,-0.22705,0.12207,0.39917,0.55664,0.45898,0.24902,-0.0097656,-0.34302,-0.45166,-0.32715,-0.070801,0.23071,0.47607,0.53711,0.40771,0.1062,-0.19043,-0.45044,-0.46753,-0.25879,0.029297,0.37231,0.55054,0.57861,0.2832,-0.047607,-0.34424,-0.49805,-0.40649,-0.2124,0.096436,0.34424,0.46997,0.36865,0.14404,-0.19531,-0.41992,-0.47363,-0.29419,-0.0061035,0.33203,0.52856,0.44678,0.28564,0.012207,-0.29053,-0.4834,-0.37354,-0.19043,0.1001,0.448,0.54321,0.41626,0.15869,-0.16357,-0.42236,-0.48218,-0.31372,-0.021973,0.26123,0.52856,0.50537,0.29785,0.020752,-0.28076,-0.52368,-0.36621,-0.15015,0.11963,0.45288,0.52368,0.36255,0.12085,-0.15381,-0.43823,-0.53467,-0.38086,-0.084229,0.16602,0.49805,0.52124,0.26489,0.015869,-0.16235,-0.52124,-0.39917,-0.1355,0.014648,0.43701,0.52734,0.4126,0.10498,-0.19043,-0.42603,-0.52612,-0.36743,-0.081787,0.14648,0.42847,0.49316,0.25391,0.10254,-0.16602,-0.46021,-0.40283,-0.13672,0.15015,0.4834,0.56396,0.42725,0.24048,-0.056152,-0.31372,-0.54199,-0.39795,0.0048828,0.22949,0.49805,0.62988,0.38574,0.089111,-0.16846,-0.44922,-0.44678,-0.13428,0.12939,0.4126,0.54688,0.40161,0.14893,-0.058594,-0.38574,-0.55786,-0.39307,-0.17212,0.1416,0.41138,0.50659,0.38818,0.053711,-0.15381,-0.46143,-0.42725,-0.20996,0.11719,0.36743,0.44434,0.42236,0.21484,-0.1416,-0.3479,-0.51514,-0.38452,-0.087891,0.24902,0.45776,0.55176,0.41748,0.1001,-0.19409,-0.4248,-0.46387,-0.29663,0.025635,0.39185,0.53833,0.4834,0.27832,-0.079346,-0.37354,-0.49805,-0.40649,-0.18311,0.10742,0.40161,0.50293,0.36987,0.068359,-0.21973,-0.47852,-0.47729,-0.271,0.0012207,0.40039,0.49438,0.42847,0.25146,-0.081787,-0.32959,-0.52856,-0.44678,-0.21484,0.085449,0.43457,0.50537,0.38574,0.10742,-0.17456,-0.46631,-0.46875,-0.30396,-0.043945,0.27832,0.53955,0.49927,0.24414,-0.073242,-0.35645,-0.57007,-0.35278,-0.15137,0.056152,0.37964,0.50293,0.38818,0.12573,-0.12695,-0.39551,-0.50903,-0.29053,-0.029297,0.29175,0.5249,0.51392,0.29053,-0.021973,-0.29907,-0.52612,-0.47974,-0.19531,0.084229,0.36743,0.5542,0.40405,0.12207,-0.1123,-0.37231,-0.5188,-0.2832,0,0.23071,0.49072,0.54688,0.26367,-0.03418,-0.28076,-0.52368,-0.44678,-0.13184,0.045166,0.44434,0.49316,0.44922,0.22827,-0.072021,-0.34668,-0.50171,-0.33203,0.014648,0.28198,0.5127,0.57739,0.38696,0.085449,-0.2002,-0.46387,-0.46143,-0.15747,0.1416,0.41992,0.55908,0.49805,0.2124,-0.062256,-0.29541,-0.48584,-0.35034,-0.024414,0.28564,0.5188,0.5835,0.32715,0.10376,-0.17944,-0.49316,-0.448,-0.30029,0.031738,0.31372,0.47607,0.4248,0.19775,-0.14404,-0.35889,-0.46997,-0.35767,-0.10132,0.22461,0.48706,0.52002,0.40527,0.065918,-0.25146,-0.49438,-0.5127,-0.30762,-0.0024414,0.34912,0.47974,0.43701,0.2356,-0.1001,-0.3418,-0.49438,-0.41626,-0.15991,0.19165,0.41138,0.5127,0.39917,0.064697,-0.24292,-0.4541,-0.48584,-0.26611,-0.036621,0.29175,0.50537,0.40161,0.21484,-0.070801,-0.3894,-0.50415,-0.37598,-0.15015,0.16602,0.51025,0.54443,0.40039,0.10376,-0.16846,-0.46753,-0.50415,-0.31372,-0.018311,0.26733,0.50537,0.47974,0.2417,-0.023193,-0.31128,-0.47729,-0.37231,-0.15991,0.11353,0.53711,0.51514,0.39185,0.19897,-0.10132,-0.43945,-0.50537,-0.26367,-0.021973,0.21729,0.54688,0.47119,0.21973,-0.036621,-0.33813,-0.55176,-0.4541,-0.16602,0.087891,0.34668,0.52246,0.40771,0.098877,-0.16113,-0.35278,-0.52734,-0.33691,-0.03418,0.25635,0.48706,0.53711,0.2417,-0.050049,-0.29053,-0.53589,-0.448,-0.20386,0.085449,0.42114,0.56152,0.44434,0.18311,-0.13428,-0.37598,-0.47852,-0.28076,0.0354,0.26855,0.4834,0.46143,0.26489,-0.037842,-0.25635,-0.50293,-0.41138,-0.13794,0.1416,0.46631,0.52734,0.47852,0.177,-0.11963,-0.34424,-0.4895,-0.40527,-0.095215,0.20264,0.47607,0.49927,0.32471,0.010986,-0.27222,-0.46143,-0.45654,-0.2478,0.073242,0.35522,0.52979,0.46875,0.177,-0.17456,-0.35889,-0.4248,-0.32104,-0.0085449,0.17578,0.42847,0.47852,0.33691,0.023193,-0.29419,-0.49561,-0.46875,-0.31616,0.010986,0.40283,0.48828,0.48584,0.24536,0.015869,-0.32593,-0.46143,-0.34424,-0.072021,0.23193,0.45288,0.49805,0.34058,0.048828,-0.25146,-0.48706,-0.49072,-0.27832,0.0073242,0.32471,0.46753,0.41504,0.19531,-0.089111,-0.35645,-0.50049,-0.38452,-0.15137,0.17822,0.47119,0.51147,0.39063,0.1123,-0.22095,-0.49805,-0.47852,-0.2124,0.021973,0.31982,0.5481,0.4834,0.23682,-0.057373,-0.38086,-0.56396,-0.41016,-0.19897,0.043945,0.32837,0.41992,0.2478,-0.023193,-0.30273,-0.54932,-0.61523,-0.38818,-0.098877,0.2356,0.49316,0.45776,0.26123,-0.01709,-0.31006,-0.5127,-0.40405,-0.13184,0.17334,0.43457,0.53955,0.42847,0.14404,-0.11475,-0.3833,-0.4895,-0.2002,0.097656,0.36621,0.59326,0.56396,0.32715,0.03418,-0.2002,-0.46021,-0.39795,-0.13916,0.15503,0.47119,0.57617,0.50537,0.1709,-0.16235,-0.36499,-0.48706,-0.38086,-0.024414,0.22705,0.4834,0.5481,0.3064,-0.0073242,-0.27588,-0.48584,-0.40527,-0.15137,0.12451,0.35767,0.45654,0.46387,0.21729,-0.083008,-0.36987,-0.5188,-0.33691,-0.083008,0.19653,0.49316,0.50049,0.33813,0.050049,-0.25391,-0.448,-0.43701,-0.25513,0.14893,0.42236,0.5542,0.46875,0.17334,-0.22949,-0.39185,-0.4541,-0.30029,-0.032959,0.25146,0.50781,0.56519,0.39307,0.067139,-0.23193,-0.44067,-0.37109,-0.15625,0.096436,0.37598,0.49561,0.47974,0.20996,-0.093994,-0.42969,-0.59814,-0.43823,-0.11597,0.17822,0.43579,0.47852,0.33936,0.10376,-0.21606,-0.45044,-0.41504,-0.22705,0.072021,0.43213,0.52979,0.41748,0.22217,-0.032959,-0.3064,-0.46143,-0.34424,-0.11597,0.20752,0.55176,0.61401,0.42236,0.15869,-0.14648,-0.4248,-0.44434,-0.21973,0.037842,0.32471,0.59082,0.54199,0.25391,-0.080566,-0.33081,-0.51636,-0.32593,-0.084229,0.1709,0.448,0.49438,0.35767,0.1001,-0.2063,-0.47485,-0.45898,-0.22583,0.047607,0.32959,0.50903,0.46631,0.25513,-0.048828,-0.32104,-0.59326,-0.46021,-0.15137,0.13916,0.39551,0.53467,0.39307,0.06958,-0.18066,-0.39063,-0.49316,-0.24414,0.073242,0.36499,0.59082,0.59204,0.29663,-0.0036621,-0.24658,-0.47119,-0.39185,-0.15503,0.1001,0.44189,0.5481,0.46021,0.15747,-0.13062,-0.40039,-0.46753,-0.27344,0.03418,0.29541,0.50049,0.54688,0.30518,-0.029297,-0.29419,-0.49072,-0.40161,-0.1416,0.15381,0.41016,0.47607,0.42725,0.16235,-0.15503,-0.35645,-0.4541,-0.33447,-0.0036621,0.32227,0.51514,0.51147,0.33081,0.010986,-0.28687,-0.50171,-0.46631,-0.22949,0.073242,0.37354,0.49072,0.43945,0.16357,-0.19775,-0.4126,-0.48218,-0.32959,-0.013428,0.31006,0.50293,0.5542,0.31738,0.0073242,-0.31372,-0.48584,-0.4248,-0.24048,0.037842,0.37842,0.4834,0.46387,0.23926,-0.13916,-0.37842,-0.45654,-0.271,0.0024414,0.29663,0.53711,0.55908,0.37598,0.12695,-0.15991,-0.49561,-0.45776,-0.16479,0.06958,0.37598,0.49683,0.36499,0.15137,-0.098877,-0.354,-0.52368,-0.37964,-0.12085,0.19775,0.52979,0.54932,0.3833,0.068359,-0.25024,-0.48584,-0.44922,-0.27588,0,0.29053,0.53467,0.46387,0.21118,-0.090332,-0.33203,-0.50293,-0.30273,-0.059814,0.18921,0.49316,0.58228,0.38574,0.073242,-0.2124,-0.49316,-0.52124,-0.2478,0.0073242,0.33447,0.48584,0.42236,0.21484,-0.05127,-0.32471,-0.49805,-0.42114,-0.13184,0.16602,0.47363,0.53955,0.37354,0.058594,-0.21362,-0.46509,-0.48706,-0.20386,-0.024414,0.29541,0.52612,0.50659,0.23193,-0.061035,-0.28076,-0.4895,-0.39185,-0.084229,0.15259,0.44922,0.57739,0.48828,0.15625,-0.15869,-0.40527,-0.52124,-0.32349,-0.043945,0.22583,0.448,0.45898,0.20874,-0.085449,-0.35522,-0.45288,-0.34058,-0.067139,0.10742,0.4126,0.50293,0.4126,0.11719,-0.16113,-0.40405,-0.49438,-0.32593,-0.018311,0.28687,0.46387,0.49561,0.30396,-0.029297,-0.29053,-0.47119,-0.43701,-0.19043,0.15625,0.40405,0.51514,0.42725,0.1416,-0.15625,-0.36255,-0.46875,-0.29785,-0.05127,0.26367,0.47607,0.47363,0.26367,-0.0024414,-0.29541,-0.51147,-0.42603,-0.18799,0.10742,0.44922,0.52856,0.46021,0.21729,-0.1123,-0.36865,-0.47852,-0.37109,-0.070801,0.22461,0.44922,0.4834,0.28442,-0.014648,-0.26978,-0.49316,-0.45166,-0.19897,0.092773,0.41138,0.5249,0.40894,0.18555,-0.097656,-0.39063,-0.51636,-0.28687,-0.10986,0.14893,0.4834,0.50171,0.3064,0.031738,-0.25024,-0.52124,-0.45898,-0.2417,0.021973,0.33081,0.52979,0.43823,0.16235,-0.11475,-0.354,-0.48096,-0.33936,-0.1123,0.16602,0.45166,0.49927,0.32349,0.042725,-0.25879,-0.50293,-0.43091,-0.24048,0.010986,0.44434,0.52979,0.46265,0.2124,0.037842,-0.31006,-0.51758,-0.34424,-0.0097656,0.15015,0.47852,0.47485,0.28076,-0.026855,-0.26367,-0.50659,-0.54443,-0.27466,0.015869,0.32227,0.49683,0.49805,0.23315,-0.068359,-0.30151,-0.46997,-0.41626,-0.14038,0.12939,0.44434,0.55176,0.41748,0.075684,-0.22461,-0.46631,-0.5481,-0.2832,0.0048828,0.26123,0.47974,0.48828,0.25024,-0.073242,-0.30273,-0.49683,-0.4126,-0.11841,0.2063,0.4187,0.51392,0.37598,0.084229,-0.20874,-0.44434,-0.5127,-0.36255,-0.048828,0.32227,0.48828,0.48096,0.34424,-0.041504,-0.31372,-0.4834,-0.42358,-0.1709,0.1416,0.36133,0.52002,0.40527,0.10742,-0.19287,-0.44067,-0.51025,-0.29785,-0.0061035,0.29053,0.48218,0.47363,0.28442,-0.015869,-0.29297,-0.45776,-0.42603,-0.2417,0.095215,0.45898,0.52246,0.41992,0.16724,-0.18433,-0.46143,-0.48828,-0.38818,-0.11353,0.2417,0.50781,0.47363,0.2771,-0.036621,-0.30273,-0.47363,-0.39307,-0.15381,0.12573,0.40039,0.51636,0.40405,0.15137,-0.13184,-0.4248,-0.53467,-0.39063,-0.11597,0.19775,0.47852,0.50659,0.32349,0.074463,-0.28198,-0.5127,-0.38086,-0.10132,0.053711,0.45898,0.52246,0.37598,0.22949,-0.032959,-0.33691,-0.53101,-0.2832,0.0036621,0.26611,0.48828,0.52734,0.27588,0.0036621,-0.21973,-0.4895,-0.46143,-0.19653,0.047607,0.37476,0.54199,0.47729,0.21606,-0.10742,-0.39673,-0.54565,-0.33936,-0.11963,0.17334,0.4187,0.52368,0.34424,0.0061035,-0.15381,-0.50659,-0.46143,-0.11719,0.075684,0.42725,0.54199,0.43091,0.14404,-0.083008,-0.32227,-0.56152,-0.43457,-0.15137,0.12573,0.40405,0.50171,0.3833,0.062256,-0.21973,-0.43945,-0.52002,-0.29175,0.037842,0.30884,0.51514,0.45898,0.22461,-0.029297,-0.31372,-0.48706,-0.36865,-0.083008,0.25024,0.48462,0.56152,0.3479,0.10986,-0.17456,-0.38574,-0.46875,-0.21729,-0.021973,0.33081,0.49438,0.51147,0.271,-0.095215,-0.3418,-0.45898,-0.41138,-0.1416,0.078125,0.4126,0.448,0.36621,0.048828,-0.15259,-0.44067,-0.44189,-0.20508,0.12451,0.43579,0.53101,0.46753,0.26855,-0.031738,-0.35034,-0.5481,-0.42358,-0.2124,0.1001,0.45044,0.50293,0.40527,0.15137,-0.15869,-0.39917,-0.46021,-0.3418,-0.040283,0.2832,0.51147,0.50415,0.30518,-0.063477,-0.35278,-0.5249,-0.43457,-0.22583,0.070801,0.37598,0.49072,0.38574,0.15137,-0.16602,-0.43823,-0.47974,-0.30273,-0.05127,0.2356,0.53711,0.49683,0.26855,0.041504,-0.31738,-0.5542,-0.448,-0.20752,0.053711,0.35034,0.52612,0.40039,0.14771,-0.12695,-0.39673,-0.52002,-0.32715,-0.028076,0.27832,0.53345,0.49805,0.25513,0.018311,-0.25513,-0.50171,-0.44678,-0.25146,0.0012207,0.37476,0.53467,0.40405,0.15747,-0.10742,-0.38818,-0.56641,-0.2771,-0.076904,0.14771,0.45044,0.56152,0.31616,0.0012207,-0.2002,-0.49072,-0.46143,-0.15137,0.15625,0.41016,0.54443,0.47485,0.23438,-0.05249,-0.28809,-0.47241,-0.31372,-0.024414,0.16846,0.54199,0.52856,0.35278,0.041504,-0.1416,-0.47607,-0.44678,-0.20264,-0.015869,0.25024,0.48462,0.44678,0.15503,-0.15137,-0.35522,-0.5188,-0.40039,-0.068359,0.19409,0.41504,0.51758,0.38574,0.0354,-0.14038,-0.4541,-0.46143,-0.21362,0.087891,0.40771,0.54688,0.43579,0.23438,0.05127,-0.25635,-0.50537,-0.34058,-0.15503,0.15381,0.42358,0.50781,0.3833,0.075684,-0.22339,-0.42725,-0.49316,-0.27832,0.0097656,0.40039,0.48706,0.43945,0.19043,-0.12817,-0.37231,-0.50293,-0.40039,-0.18066,0.14526,0.43457,0.47974,0.43457,0.13306,-0.19287,-0.448,-0.48096,-0.32715,-0.072021,0.28564,0.4895,0.42236,0.25879,-0.043945,-0.33325,-0.51758,-0.42358,-0.16357,0.16113,0.41504,0.51514,0.40161,0.12939,-0.16479,-0.38696,-0.52856,-0.33203,-0.026855,0.25879,0.48096,0.46143,0.24414,-0.040283,-0.30884,-0.51758,-0.42114,-0.23438,0.039063,0.48462,0.53711,0.40039,0.15625,-0.15015,-0.44189,-0.45898,-0.21118,0.061035,0.30518,0.49316,0.48218,0.23315,-0.019531,-0.27832,-0.53345,-0.39307,-0.2002,0.05249,0.42114,0.51758,0.448,0.17334,-0.13184,-0.37231,-0.44922,-0.31616,-0.0061035,0.18921,0.55176,0.49561,0.25513,0.076904,-0.21973,-0.46509,-0.40527,-0.13672,0.028076,0.43823,0.51025,0.42725,0.12207,-0.12695,-0.35522,-0.5542,-0.33569,-0.058594,0.20874,0.44922,0.50293,0.29053,-0.01709,-0.28809,-0.46631,-0.47363,-0.21606,0.12695,0.38818,0.55176,0.52979,0.2832,-0.041504,-0.2832,-0.46387,-0.28564,0.012207,0.28564,0.44556,0.5249,0.34668,0.013428,-0.28687,-0.53955,-0.54932,-0.2063,0.010986,0.32959,0.50293,0.48218,0.22827,-0.074463,-0.29785,-0.47607,-0.39307,-0.11963,0.19043,0.43457,0.46387,0.3418,0.03418,-0.19775,-0.47241,-0.41626,-0.2356,-0.0085449,0.36621,0.52124,0.44678,0.22949,-0.080566,-0.33569,-0.47974,-0.39063,-0.16113,0.11963,0.46509,0.52246,0.39795,0.075684,-0.26855,-0.53223,-0.4834,-0.31616,-0.01709,0.31982,0.4834,0.45166,0.27222,-0.063477,-0.31616,-0.45776,-0.36133,-0.13794,0.16724,0.44556,0.48584,0.36133,0.1001,-0.19287,-0.48584,-0.54077,-0.32104,-0.061035,0.31128,0.56641,0.50537,0.23926,0.0048828,-0.31128,-0.53711,-0.4126,-0.18311,0.085449,0.39917,0.56152,0.38086,0.1123,-0.19165,-0.45166,-0.52124,-0.32227,-0.079346,0.24902,0.47607,0.47607,0.25024,0.05249,-0.28564,-0.50781,-0.354,-0.1123,0.16357,0.49438,0.60059,0.40039,0.12695,-0.079346,-0.41382,-0.55786,-0.38452,-0.042725,0.19165,0.448,0.53833,0.26855,-0.012207,-0.2356,-0.48584,-0.4126,-0.16113,0.13184,0.40894,0.53955,0.43945,0.14282,-0.16479,-0.39551,-0.54321,-0.41138,-0.1123,0.17456,0.45044,0.51758,0.31128,-0.0097656,-0.26733,-0.4895,-0.45776,-0.21484,0.1001,0.35645,0.55664,0.44556,0.16235,-0.177,-0.37842,-0.53345,-0.33936,-0.10254,0.18433,0.41016,0.52612,0.32715,0.018311,-0.2478,-0.47119,-0.46021,-0.26855,0.063477,0.41748,0.50781,0.46021,0.2356,-0.1123,-0.39429,-0.52246,-0.41992,-0.15625,0.16113,0.41626,0.46143,0.354,0.053711,-0.24414,-0.47363,-0.47363,-0.25146,0.065918,0.36621,0.53345,0.44678,0.20264,-0.14526,-0.39185,-0.49561,-0.35645,-0.2002,0.14893,0.44434,0.47363,0.38574,0.075684,-0.16846,-0.48584,-0.37842,-0.18799,-0.043945,0.30273,0.53467,0.45044,0.23315,0.019531,-0.32715,-0.52002,-0.31372,-0.05127,0.11475,0.49927,0.48096,0.35034,0.083008,-0.21973,-0.47363,-0.52612,-0.27222,-0.01709,0.3772,0.51514,0.448,0.23926,-0.032959,-0.32959,-0.49316,-0.36987,-0.12207,0.06958,0.51636,0.5481,0.33325,0.074463,-0.17822,-0.44678,-0.52246,-0.26611,-0.041504,0.28564,0.53589,0.50049,0.26123,-0.030518,-0.29785,-0.52368,-0.42236,-0.18066,0.091553,0.37109,0.47363,0.3894,0.13306,-0.17822,-0.45166,-0.56641,-0.26611,-0.0085449,0.23193,0.50049,0.48828,0.25269,-0.020752,-0.25146,-0.51514,-0.42969,-0.18433,0.10864,0.38574,0.53955,0.3833,0.084229,-0.17334,-0.36621,-0.50781,-0.354,-0.025635,0.25635,0.51636,0.54321,0.31128,-0.014648,-0.26978,-0.4895,-0.44922,-0.23438,0.063477,0.33081,0.47363,0.43579,0.12451,-0.18555,-0.39429,-0.50659,-0.38574,-0.032959,0.21973,0.45898,0.55176,0.34302,0.023193,-0.26855,-0.51758,-0.49072,-0.25024,0.014648,0.37842,0.48584,0.44678,0.16113,-0.14648,-0.3833,-0.4541,-0.32349,-0.025635,0.28809,0.5249,0.57617,0.4126,0.11597,-0.22705,-0.43091,-0.39063,-0.17822,0.03418,0.46875,0.44434,0.39063,0.15747,-0.15015,-0.36987,-0.51636,-0.37964,-0.15137,0.18188,0.4541,0.54932,0.36133,0.018311,-0.14648,-0.46509,-0.46997,-0.17944,0.032959,0.28687,0.50781,0.43213,0.19409,-0.11475,-0.37842,-0.55908,-0.2771,-0.1355,0.13794,0.41626,0.46631,0.3186,0.083008,-0.1355,-0.53711,-0.46631,-0.26611,-0.012207,0.33081,0.51025,0.39917,0.19531,-0.070801,-0.37231,-0.53711,-0.41016,-0.16479,0.13306,0.4187,0.51636,0.38818,0.078125,-0.19897,-0.41748,-0.46753,-0.27954,-0.010986,0.31738,0.52856,0.46387,0.21973,-0.10742,-0.35522,-0.52124,-0.42725,-0.14648,0.12207,0.44189,0.53833,0.43335,0.12695,-0.1709,-0.43091,-0.50781,-0.26978,0.01709,0.21118,0.47852,0.45166,0.19653,-0.081787,-0.3064,-0.55786,-0.39307,-0.091553,0.12207,0.4541,0.49072,0.38452,0.080566,-0.058594,-0.40161,-0.52612,-0.37598,-0.054932,0.26611,0.52856,0.50903,0.24902,-0.11108,-0.35278,-0.49072,-0.40283,-0.10498,0.15747,0.41016,0.59204,0.51514,0.1709,-0.1001,-0.29907,-0.43945,-0.26367,-0.032959,0.2478,0.448,0.52856,0.31982,-0.024414,-0.32837,-0.53955,-0.47119,-0.21729,0.025635,0.50171,0.50293,0.51025,0.24414,-0.085449,-0.32959,-0.4541,-0.26245,0.029297,0.29419,0.5249,0.44922,0.26855,0.080566,-0.28564,-0.46021,-0.41626,-0.18555,0.090332,0.38086,0.45166,0.46143,0.22705,-0.15381,-0.37354,-0.4895,-0.41748,-0.072021,0.2417,0.47974,0.48584,0.29053,-0.014648,-0.27832,-0.49805,-0.49072,-0.19043,0.092773,0.3894,0.58105,0.46631,0.2478,-0.020752,-0.27222,-0.42236,-0.23193,-0.010986,0.14893,0.48096,0.52612,0.33691,0.068359,-0.20996,-0.53955,-0.44312,-0.20386,0.10132,0.47119,0.53955,0.4248,0.23438,-0.085449,-0.38574,-0.52979,-0.26855,-0.12207,0.18066,0.45166,0.47363,0.31738,0.036621,-0.20752,-0.47852,-0.5249,-0.27832,0.0036621,0.29785,0.53223,0.4541,0.21973,-0.046387,-0.30029,-0.51758,-0.42969,-0.14648,0.1123,0.46753,0.58228,0.38574,0.039063,-0.1416,-0.49805,-0.46021,-0.19287,0.059814,0.33203,0.57617,0.45654,0.29663,0.03418,-0.2832,-0.44189,-0.28564,-0.010986,0.12451,0.39795,0.51025,0.41626,0.089111,-0.19775,-0.448,-0.58472,-0.36621,-0.0036621,0.30518,0.4834,0.52002,0.25269,-0.048828,-0.30029,-0.5127,-0.45776,-0.14771,0.16235,0.39917,0.51758,0.38208,0.087891,-0.2124,-0.4126,-0.53223,-0.32227,-0.057373,0.23926,0.45654,0.51758,0.27222,-0.043945,-0.31006,-0.49194,-0.44067,-0.19409,0.12451,0.43213,0.52856,0.46753,0.16602,-0.18799,-0.39429,-0.51147,-0.37354,-0.075684,0.23071,0.44312,0.4834,0.30762,-0.01709,-0.28687,-0.46997,-0.38696,-0.16357,0.15991,0.42725,0.51758,0.43701,0.18555,-0.14893,-0.38818,-0.51758,-0.39917,-0.092773,0.21729,0.46387,0.49316,0.3186,0.021973,-0.25513,-0.5127,-0.44922,-0.23682,0.065918,0.38696,0.53711,0.39551,0.17822,-0.12939,-0.39551,-0.49683,-0.36133,-0.15869,0.16846,0.49316,0.44678,0.26367,0.053711,-0.26123,-0.5127,-0.43213,-0.25635,0.026855,0.41016,0.56274,0.47852,0.2124,-0.10132,-0.3833,-0.51514,-0.35889,-0.079346,0.17456,0.43701,0.50659,0.32471,0.05249,-0.21729,-0.47607,-0.48096,-0.21362,0.037842,0.3418,0.54077,0.46509,0.18677,0.043945,-0.26123,-0.54199,-0.37476,-0.042725,0.096436,0.52246,0.50659,0.29541,0.10498,-0.14526,-0.43945,-0.48706,-0.19165,0.06958,0.33447,0.57373,0.46875,0.31006,0.0012207,-0.25391,-0.46631,-0.28809,-0.020752,0.13916,0.47363,0.52856,0.41016,0.065918,-0.1416,-0.46753,-0.50659,-0.18188,0.12695,0.4248,0.59082,0.51392,0.27466,0.068359,-0.22095,-0.49683,-0.41626,-0.08667,0.10376,0.35034,0.49072,0.31982,0.03418,-0.25146,-0.43945,-0.50049,-0.31616,-0.0061035,0.3418,0.48584,0.50293,0.28931,-0.089111,-0.31372,-0.42847,-0.41748,-0.21973,0.10864,0.38818,0.48096,0.43823,0.11597,-0.19653,-0.4126,-0.53223,-0.33936,-0.01709,0.26855,0.47607,0.46875,0.28564,-0.025635,-0.27466,-0.49805,-0.42847,-0.19043,0.12817,0.38818,0.47241,0.37231,0.11841,-0.18066,-0.39063,-0.45532,-0.32104,0.020752,0.33936,0.53711,0.50049,0.32349,0.024414,-0.21606,-0.52002,-0.33447,-0.15015,0.048828,0.46021,0.51147,0.3894,0.14282,-0.15625,-0.41504,-0.48218,-0.31128,-0.059814,0.22583,0.50293,0.48706,0.3186,0.046387,-0.29297,-0.50049,-0.41138,-0.2356,0.063477,0.42725,0.4834,0.44067,0.1709,-0.14038,-0.42114,-0.53955,-0.37598,-0.10742,0.16846,0.48218,0.52002,0.29053,0.063477,-0.22339,-0.5249,-0.4541,-0.17944,0.041504,0.3833,0.50049,0.40039,0.1416,-0.1355,-0.37598,-0.54932,-0.32471,-0.10254,0.15503,0.4248,0.5127,0.33813,0.06958,-0.19897,-0.4834,-0.49316,-0.24536,-0.0085449,0.29419,0.5188,0.40771,0.1355,-0.13184,-0.36499,-0.57739,-0.31372,-0.10986,0.17212,0.44189,0.53955,0.33203,0.013428,-0.21729,-0.42114,-0.47729,-0.24292,0.012207,0.41138,0.46631,0.44922,0.22949,0.014648,-0.25513,-0.49683,-0.37598,-0.057373,0.25391,0.51025,0.61035,0.40039,0.16968,-0.10498,-0.42725,-0.4541,-0.17944,0.036621,0.34912,0.5188,0.45898,0.21484,-0.14282,-0.31006,-0.50171,-0.43701,-0.18066,0.12207,0.38696,0.48828,0.42847,0.097656,-0.15991,-0.4187,-0.50049,-0.22949,-0.0097656,0.26367,0.47241,0.48584,0.271,-0.070801,-0.37354,-0.5603,-0.36011,-0.16846,0.13672,0.45044,0.50171,0.37109,0.11963,-0.18311,-0.41992,-0.45776,-0.35034,-0.073242,0.31372,0.51514,0.5127,0.28076,-0.05249,-0.35645,-0.48584,-0.4248,-0.177,0.12695,0.41992,0.56152,0.49316,0.21973,-0.10254,-0.38086,-0.44434,-0.23193,0.073242,0.40161,0.52979,0.47241,0.24902,0.080566,-0.24414,-0.56152,-0.4126,-0.14893,0.053711,0.5188,0.53467,0.40894,0.16113,-0.024414,-0.40771,-0.44434,-0.22339,-0.043945,0.20508,0.46509,0.51025,0.27222,0.0097656,-0.29541,-0.54565,-0.48218,-0.24292,0.042725,0.36255,0.51392,0.41748,0.14893,-0.028076,-0.37476,-0.49561,-0.29053,0.01709,0.23804,0.54199,0.51514,0.271,0.076904,-0.1709,-0.53589,-0.49316,-0.15747,0.030518,0.27588,0.54199,0.46631,0.17212,-0.079346,-0.32959,-0.52002,-0.35278,-0.083008,0.21973,0.49316,0.5542,0.34668,0.037842,-0.28809,-0.52246,-0.51514,-0.30029,-0.0048828,0.35156,0.50049,0.47363,0.20752,-0.14771,-0.32959,-0.49316,-0.41138,-0.087891,0.17578,0.35889,0.50415,0.40649,0.026855,-0.21973,-0.46753,-0.56152,-0.31738,0.045166,0.31738,0.46021,0.43091,0.23193,-0.10254,-0.33447,-0.5249,-0.3833,-0.080566,0.18555,0.58472,0.52734,0.39795,0.041504,-0.24414,-0.42847,-0.47363,-0.31616,-0.025635,0.24414,0.47607,0.47363,0.24902,-0.056152,-0.32959,-0.45898,-0.37842,-0.12695,0.18066,0.41992,0.50903,0.4248,0.13794,-0.18921,-0.47852,-0.54688,-0.2478,-0.03418,0.30273,0.49805,0.43945,0.25024,-0.026855,-0.36133,-0.45532,-0.31494,-0.087891,0.1123,0.39551,0.47119,0.35156,0.12451,-0.19287,-0.44312,-0.50293,-0.30151,-0.089111,0.26489,0.50049,0.45898,0.26611,-0.043945,-0.34546,-0.5127,-0.42358,-0.20264,0.12451,0.44067,0.53223,0.42847,0.1709,-0.15503,-0.40771,-0.53711,-0.34668,-0.095215,0.2002,0.48706,0.46875,0.20996,-0.021973,-0.23682,-0.53833,-0.41382,-0.16113,0.056152,0.39795,0.55054,0.39185,0.13184,-0.11719,-0.39551,-0.54077,-0.354,-0.092773,0.16113,0.46143,0.47974,0.26489,-0.021973,-0.29907,-0.5249,-0.40405,-0.15015,0.053711,0.4248,0.55176,0.41992,0.16113,-0.11719,-0.38086,-0.55176,-0.28687,-0.074463,0.19165,0.48584,0.5127,0.29419,0.014648,-0.22827,-0.48096,-0.50903,-0.27588,0.083008,0.40405,0.5542,0.49927,0.16479,-0.11963,-0.32837,-0.46875,-0.38818,-0.1123,0.15625,0.37354,0.49316,0.31006,-0.010986,-0.27344,-0.4834,-0.4541,-0.13672,0.046387,0.41138,0.49316,0.44434,0.19409,-0.078125,-0.35645,-0.5481,-0.36255,-0.12085,0.19043,0.41626,0.45166,0.34424,0.046387,-0.26367,-0.45044,-0.47363,-0.22339,0.036621,0.39063,0.48096,0.42847,0.24536,-0.098877,-0.33691,-0.48584,-0.32104,-0.1709,0.12207,0.41138,0.47852,0.33691,0.043945,-0.26611,-0.49072,-0.49194,-0.31616,-0.028076,0.34424,0.52734,0.47852,0.26978,-0.053711,-0.32471,-0.45776,-0.4187,-0.16724,0.1416,0.38208,0.47363,0.34912,0.063477,-0.2124,-0.46631,-0.4834,-0.28442,-0.020752,0.33203,0.52246,0.46509,0.271,-0.01709,-0.32959,-0.56152,-0.36255,-0.2124,0.085449,0.49316,0.49316,0.37964,0.073242,-0.093994,-0.39917,-0.54077,-0.27222,0.05127,0.31494,0.60059,0.46997,0.22461,0.098877,-0.19287,-0.46509,-0.34546,-0.12451,0.16479,0.4834,0.59082,0.42358,0.16113,-0.08667,-0.37476,-0.48096,-0.25513,0.0012207,0.28198,0.61523,0.52979,0.28076,-0.013428,-0.28442,-0.45288,-0.37354,-0.11963,0.070801,0.39185,0.57617,0.42358,0.18066,-0.11963,-0.39185,-0.54932,-0.29297,0.056152,0.29541,0.5249,0.57739,0.28809,0.12207,-0.1355,-0.54199,-0.44434,-0.15625,0.083008,0.50171,0.50293,0.41016,0.10498,-0.062256,-0.35278,-0.46631,-0.33936,0.0085449,0.26123,0.54199,0.53711,0.2832,0.05127,-0.18555,-0.43701,-0.35278,-0.11597,0.073242,0.43091,0.4834,0.45776,0.16846,-0.17822,-0.4187,-0.5542,-0.39551,-0.10254,0.19043,0.44434,0.50171,0.39307,0.10498,-0.23315,-0.45532,-0.45898,-0.24414,0.075684,0.35645,0.52979,0.4541,0.21729,-0.1001,-0.32104,-0.51147,-0.3772,-0.11963,0.22217,0.50293,0.51514,0.39063,0.083008,-0.24658,-0.40649,-0.44434,-0.29053,0.014648,0.39429,0.49561,0.45898,0.25513,-0.076904,-0.39429,-0.5542,-0.43213,-0.17456,0.11963,0.40527,0.49072,0.35645,0.063477,-0.22827,-0.48584,-0.50049,-0.22827,0.028076,0.32959,0.51025,0.42847,0.21973,-0.095215,-0.36255,-0.53833,-0.32959,-0.20264,0.095215,0.42969,0.51025,0.40405,0.15137,-0.16846,-0.46631,-0.46875,-0.29541,-0.019531,0.28442,0.53589,0.47607,0.19043,-0.062256,-0.32227,-0.57495,-0.43213,-0.15381,0.081787,0.41748,0.51758,0.35767,0.097656,-0.16602,-0.41504,-0.47607,-0.28076,-0.024414,0.28442,0.52368,0.51025,0.25269,-0.031738,-0.29785,-0.52856,-0.45288,-0.2063,0.081787,0.36865,0.53955,0.41626,0.13428,-0.18311,-0.39917,-0.50049,-0.27832,0.014648,0.25635,0.48462,0.54321,0.28076,-0.037842,-0.26367,-0.52002,-0.48218,-0.21362,0.084229,0.34912,0.46143,0.45166,0.13672,-0.053711,-0.34912,-0.47241,-0.33569,-0.046387,0.23315,0.49316,0.5127,0.24658,-0.040283,-0.28564,-0.51147,-0.40527,-0.15625,0.054932,0.39185,0.51758,0.40771,0.1355,-0.13062,-0.354,-0.44556,-0.37231,-0.070801,0.19409,0.41748,0.52246,0.36133,0.029297,-0.30273,-0.51392,-0.45532,-0.23682,0.046387,0.41016,0.42358,0.42847,0.18555,-0.16357,-0.39551,-0.47607,-0.32471,-0.053711,0.23193,0.46631,0.48096,0.31494,0.063477,-0.26489,-0.50293,-0.48218,-0.27344,-0.0097656,0.4126,0.50293,0.45044,0.21729,-0.10254,-0.36255,-0.47485,-0.4248,-0.14771,0.21606,0.46387,0.53467,0.37231,0.05249,-0.28076,-0.46265,-0.45776,-0.2356,0.012207,0.32227,0.47363,0.40527,0.21973,-0.058594,-0.3418,-0.51025,-0.27588,-0.14893,0.11475,0.47852,0.5542,0.40039,0.12451,-0.16113,-0.45898,-0.48462,-0.27588,-0.029297,0.26855,0.50415,0.42969,0.22339,-0.056152,-0.33936,-0.51392,-0.34424,-0.12939,0.17944,0.47363,0.49927,0.34058,0.073242,-0.19653,-0.46143,-0.48828,-0.31372,-0.043945,0.27222,0.49561,0.44434,0.21118,-0.073242,-0.32227,-0.54443,-0.40527,-0.17822,0.06958,0.34912,0.59082,0.42725,0.05249,-0.18799,-0.40771,-0.51392,-0.21729,-0.023193,0.24536,0.44434,0.46021,0.23926,0.043945,-0.21851,-0.49194,-0.34058,-0.15747,0.073242,0.4248,0.5127,0.43091,0.15259,-0.13062,-0.42358,-0.58105,-0.37109,-0.036621,0.26123,0.448,0.47485,0.21484,-0.091553,-0.27832,-0.48218,-0.41138,-0.21973,0.10986,0.39673,0.51636,0.4248,0.12939,-0.20874,-0.39551,-0.48462,-0.32959,-0.12573,0.18433,0.45654,0.49805,0.32593,0,-0.25391,-0.51758,-0.44678,-0.21606,0.096436,0.32715,0.50293,0.46509,0.20386,-0.12207,-0.35156,-0.49194,-0.34668,-0.026855,0.271,0.43457,0.42847,0.26367,0.023193,-0.28198,-0.49805,-0.45898,-0.27588,0.050049,0.46021,0.50537,0.4248,0.22461,-0.097656,-0.36621,-0.47852,-0.33569,-0.1709,0.15503,0.43945,0.4895,0.29785,-0.046387,-0.30762,-0.52246,-0.44434,-0.15625,0.03418,0.31738,0.50659,0.43091,0.16846,-0.1355,-0.35889,-0.48584,-0.30029,-0.079346,0.18677,0.50171,0.5249,0.36133,0.12817,-0.21362,-0.52124,-0.44312,-0.20996,0.0354,0.40039,0.5896,0.38574,0.21118,0.050049,-0.26123,-0.49561,-0.26978,-0.10864,0.16235,0.52246,0.5249,0.34668,0.087891,-0.16846,-0.44189,-0.47852,-0.19653,0.085449,0.40161,0.60303,0.50415,0.23193,-0.046387,-0.30029,-0.5249,-0.37598,-0.12329,0.1123,0.38452,0.52734,0.35889,0.06958,-0.19653,-0.49683,-0.5481,-0.24414,0.026855,0.28931,0.48584,0.4834,0.21484,-0.040283,-0.26489,-0.51636,-0.43823,-0.12573,0.1416,0.40894,0.51514,0.40283,0.06958,-0.19043,-0.42236,-0.5249,-0.3418,-0.043945,0.27344,0.48218,0.51636,0.28687,-0.092773,-0.32227,-0.39673,-0.34668,-0.058594,0.14526,0.47974,0.5249,0.40894,0.063477,-0.13062,-0.4187,-0.51147,-0.31738,-0.061035,0.25635,0.44678,0.52002,0.28198,-0.041504,-0.26367,-0.47852,-0.35156,-0.21118,0.13916,0.354,0.48828,0.39795,0.15869,-0.21729,-0.41138,-0.49316,-0.26001,-0.039063,0.29297,0.47607,0.47607,0.29785,0,-0.29541,-0.47729,-0.40039,-0.21973,0.10254,0.42603,0.49805,0.40771,0.1709,-0.15137,-0.40649,-0.45044,-0.35889,-0.032959,0.21606,0.49072,0.49805,0.31982,0.021973,-0.20996,-0.47119,-0.32959,-0.2063,0.046387,0.40405,0.55298,0.41748,0.2002,-0.083008,-0.40283,-0.52246,-0.32715,-0.10498,0.19043,0.51758,0.50049,0.34058,0.10376,-0.2417,-0.5249,-0.46875,-0.20508,0.053711,0.354,0.51392,0.39551,0.19775,-0.11841,-0.41382,-0.53223,-0.40161,-0.1416,0.17822,0.43213,0.49927,0.3479,0.030518,-0.23193,-0.48584,-0.50537,-0.22827,0.010986,0.3479,0.5542,0.48462,0.17822,-0.068359,-0.34546,-0.57617,-0.41138,-0.12451,0.10254,0.37842,0.55176,0.3418,0.031738,-0.19653,-0.44189,-0.50049,-0.19897,0.053711,0.30518,0.51025,0.46753,0.2063,-0.081787,-0.37598,-0.55176,-0.43701,-0.19043,0.10742,0.43457,0.51392,0.3772,0.070801,-0.2002,-0.44067,-0.52124,-0.2771,0.025635,0.25879,0.5249,0.50293,0.23193,-0.08667,-0.28564,-0.50903,-0.43091,-0.14404,0.14404,0.38086,0.50903,0.42236,0.084229,-0.2063,-0.36865,-0.50781,-0.2832,-0.019531,0.27466,0.46387,0.50781,0.27588,-0.059814,-0.33936,-0.45898,-0.37598,-0.14404,0.089111,0.4248,0.448,0.38208,0.078125,-0.13794,-0.41016,-0.49072,-0.2478,0.093994,0.37231,0.54321,0.47241,0.26001,-0.070801,-0.29663,-0.51025,-0.40039,-0.2478,0.1001,0.41138,0.46875,0.4126,0.16724,-0.1416,-0.4248,-0.50049,-0.39917,-0.096436,0.26367,0.5249,0.49072,0.27344,0.0085449,-0.22949,-0.5127,-0.33691,-0.2478,0.03418,0.43701,0.45776,0.36133,0.11841,-0.084229,-0.43701,-0.44434,-0.26855,0.012207,0.271,0.57861,0.48218,0.35522,0.068359,-0.271,-0.55908,-0.38452,-0.25879,0.048828,0.36865,0.46997,0.41016,0.17822,-0.097656,-0.37354,-0.5249,-0.34546,-0.037842,0.21362,0.50903,0.53345,0.26611,0.021973,-0.11963,-0.46631,-0.44678,-0.19653,0.078125,0.44189,0.50659,0.46265,0.15991,-0.0073242,-0.35889,-0.4834,-0.29419,-0.026855,0.14771,0.5481,0.54321,0.33447,0.056152,-0.23926,-0.47852,-0.47241,-0.17822,0.064697,0.31128,0.44434,0.4187,0.17578,-0.068359,-0.28198,-0.54077,-0.4187,-0.092773,0.18066,0.41382,0.55176,0.39795,0.083008,-0.15991,-0.44189,-0.55176,-0.31738,-0.023193,0.28809,0.51025,0.4248,0.18555,-0.14893,-0.38086,-0.49316,-0.36743,-0.14038,0.17944,0.43091,0.51758,0.39307,0.08667,-0.21973,-0.39551,-0.46265,-0.33936,-0.014648,0.28076,0.43945,0.46875,0.27832,-0.078125,-0.32471,-0.51147,-0.42969,-0.15869,0.16235,0.3772,0.46387,0.41504,0.10376,-0.12573,-0.42725,-0.42236,-0.23315,0.085449,0.37598,0.47852,0.40161,0.21973,-0.1062,-0.34546,-0.50293,-0.3772,-0.22217,0.070801,0.47241,0.5127,0.44189,0.15625,-0.15869,-0.43091,-0.50049,-0.30029,-0.092773,0.24414,0.49316,0.448,0.24414,-0.03418,-0.34424,-0.48584,-0.38208,-0.19897,0.08667,0.4126,0.47363,0.36621,0.14648,-0.1709,-0.40527,-0.48706,-0.32471,-0.096436,0.20264,0.50293,0.46875,0.24414,0.018311,-0.35889,-0.56152,-0.4541,-0.22949,0.048828,0.34668,0.52612,0.40771,0.177,-0.12207,-0.39551,-0.51025,-0.31494,-0.012207,0.25024,0.47363,0.44189,0.20264,-0.013428,-0.26123,-0.54565,-0.45166,-0.16113,0.0061035,0.46997,0.53955,0.42236,0.15015,0.026855,-0.23926,-0.55176,-0.27466,-0.1062,0.10254,0.44556,0.52246,0.24414,-0.039063,-0.30273,-0.54932,-0.43457,-0.23682,0.068359,0.33447,0.50537,0.44678,0.18677,-0.090332,-0.29541,-0.48706,-0.36987,-0.085449,0.14282,0.4187,0.50415,0.34424,0.046387,-0.2356,-0.49683,-0.53223,-0.26855,0.057373,0.30396,0.52734,0.4834,0.19653,-0.11963,-0.29663,-0.49927,-0.41504,-0.1001,0.16846,0.49561,0.49805,0.36255,0.085449,-0.19653,-0.42725,-0.46387,-0.22949,-0.01709,0.29785,0.44922,0.48218,0.25757,-0.087891,-0.36377,-0.51514,-0.3418,-0.13428,0.15991,0.38086,0.46753,0.37476,0.039063,-0.16602,-0.4834,-0.49438,-0.24414,0.1123,0.39185,0.43945,0.42725,0.21973,-0.10742,-0.33081,-0.43579,-0.39551,-0.12085,0.10742,0.53589,0.49316,0.38086,0.054932,-0.16602,-0.50659,-0.47119,-0.28076,-0.10254,0.24658,0.50049,0.45898,0.22949,-0.061035,-0.32715,-0.50171,-0.33081,-0.1416,0.11963,0.52246,0.47119,0.44556,0.18677,-0.14404,-0.42969,-0.51514,-0.30273,0.0097656,0.3479,0.55176,0.50659,0.28687,0.014648,-0.30762,-0.52246,-0.38086,-0.17944,0.11353,0.3833,0.49683,0.36499,0.13672,-0.15625,-0.41748,-0.53589,-0.34668,-0.061035,0.20996,0.47363,0.51758,0.21606,-0.040283,-0.21606,-0.53467,-0.37598,-0.1062,0.053711,0.5127,0.58716,0.41626,0.12939,-0.032959,-0.38574,-0.47607,-0.30151,-0.0012207,0.19165,0.59204,0.49805,0.26123,-0.040283,-0.26123,-0.53223,-0.37354,-0.083008,0.084229,0.43945,0.50781,0.43213,0.16602,-0.059814,-0.38208,-0.52246,-0.38086,0.019531,0.22949,0.36987,0.50293,0.33203,-0.031738,-0.24292,-0.52612,-0.52246,-0.24414,0.080566,0.33691,0.50171,0.44189,0.17578,-0.10498,-0.31738,-0.48584,-0.39429,-0.092773,0.18066,0.40771,0.49805,0.32227,-0.024414,-0.23804,-0.42847,-0.47729,-0.25146,0.048828,0.39795,0.53955,0.55176,0.22949,-0.12939,-0.37354,-0.50537,-0.40894,-0.14404,0.14282,0.38574,0.48462,0.3894,0.065918,-0.2771,-0.46631,-0.3894,-0.1355,0.057373,0.44067,0.448,0.37842,0.34424,0.025635,-0.26611,-0.46265,-0.37354,-0.12695,0.22217,0.51758,0.56763,0.32715,0.17334,-0.13428,-0.41016,-0.41504,-0.21484,0.053711,0.40527,0.58105,0.49072,0.29297,-0.072021,-0.34424,-0.45898,-0.34912,-0.17334,0.12817,0.41504,0.4834,0.3833,0.10254,-0.18921,-0.46021,-0.47241,-0.2417,-0.023193,0.33691,0.59448,0.53955,0.3479,0.05127,-0.28564,-0.54199,-0.37964,-0.097656,0.16846,0.45166,0.51392,0.35889,0.067139,-0.14526,-0.42358,-0.51514,-0.29053,-0.0073242,0.26123,0.50049,0.4834,0.23438,0.0073242,-0.23193,-0.53101,-0.44678,-0.23193,0.029297,0.38696,0.53345,0.37964,0.093994,-0.12939,-0.40527,-0.53467,-0.32593,-0.036621,0.24048,0.55664,0.57129,0.271,-0.026855,-0.26245,-0.47852,-0.3894,-0.16968,0.12451,0.37231,0.49805,0.43335,0.14893,-0.16357,-0.34424,-0.4895,-0.34546,-0.018311,0.24658,0.46143,0.56152,0.35889,0.043945,-0.22461,-0.47974,-0.52124,-0.20996,0.090332,0.29419,0.45776,0.41504,0.11353,-0.15869,-0.3418,-0.51514,-0.35767,0.0097656,0.25024,0.45898,0.50903,0.31128,0.024414,-0.22583,-0.46143,-0.47241,-0.26855,0.042725,0.38086,0.50903,0.46021,0.19653,-0.10254,-0.37231,-0.47607,-0.36499,-0.11597,0.22095,0.46143,0.53955,0.4126,0.084229,-0.31006,-0.47241,-0.42236,-0.22339,0.075684,0.33203,0.43701,0.43091,0.20264,-0.14648,-0.3833,-0.50293,-0.34546,-0.13184,0.18555,0.448,0.45776,0.37109,0.10986,-0.19531,-0.42847,-0.47119,-0.32715,-0.036621,0.34668,0.53467,0.42114,0.19531,-0.091553,-0.3418,-0.49561,-0.34546,-0.11108,0.19409,0.50659,0.5249,0.36011,0.074463,-0.24658,-0.48706,-0.45288,-0.26611,-0.040283,0.29907,0.46143,0.40527,0.22705,-0.073242,-0.39429,-0.57495,-0.39307,-0.18799,0.068359,0.48706,0.5249,0.32593,0.16235,-0.1062,-0.45898,-0.51636,-0.18433,0.039063,0.35889,0.59326,0.5249,0.27466,0.0024414,-0.25635,-0.4834,-0.35889,-0.063477,0.079346,0.40527,0.55664,0.40039,0.1123,-0.11475,-0.39063,-0.47974,-0.29663,-0.041504,0.26001,0.52124,0.50537,0.23926,-0.054932,-0.33325,-0.55786,-0.43579,-0.1416,0.11963,0.37354,0.52734,0.42847,0.089111,-0.13428,-0.35034,-0.51147,-0.33447,-0.0048828,0.22461,0.39795,0.49561,0.26978,0.029297,-0.2002,-0.45044,-0.45776,-0.1416,0.17578,0.3833,0.5835,0.47852,0.18555,-0.072021,-0.30029,-0.47607,-0.25879,0.058594,0.32593,0.45898,0.48706,0.26001,-0.068359,-0.31372,-0.52002,-0.43335,-0.2417,0.12451,0.40649,0.48828,0.47852,0.20264,-0.12817,-0.31372,-0.42236,-0.36499,-0.092773,0.2417,0.47363,0.50537,0.34912,0.0048828,-0.30762,-0.4834,-0.43579,-0.2417,0.063477,0.38574,0.52856,0.46753,0.19409,-0.14771,-0.39307,-0.49805,-0.38452,-0.10742,0.18066,0.4248,0.44556,0.34546,0.03418,-0.28809,-0.52368,-0.5127,-0.29175,0.0073242,0.31006,0.47119,0.40771,0.21484,-0.090332,-0.3833,-0.47974,-0.31372,-0.097656,0.18799,0.49438,0.5249,0.36621,0.056152,-0.24658,-0.49194,-0.48828,-0.24536,0.012207,0.35889,0.54443,0.45532,0.21729,-0.026855,-0.33081,-0.50293,-0.36499,-0.13672,0.14526,0.46387,0.52612,0.34912,0.063477,-0.24414,-0.51025,-0.51025,-0.25635,-0.015869,0.31006,0.50293,0.41992,0.20996,-0.037842,-0.32837,-0.51636,-0.28076,-0.13428,0.11963,0.45898,0.55298,0.40161,0.075684,-0.16602,-0.42847,-0.53711,-0.27222,0.010986,0.22827,0.51758,0.52246,0.25391,-0.050049,-0.2832,-0.50171,-0.38574,-0.10498,0.15625,0.39795,0.49194,0.36987,0.090332,-0.087891,-0.42236,-0.5127,-0.28076,0.048828,0.32715,0.54932,0.50537,0.20508,0.036621,-0.20264,-0.47485,-0.41748,-0.081787,0.089111,0.44922,0.55176,0.40527,0.046387,-0.21729,-0.39063,-0.51025,-0.31006,-0.03418,0.26245,0.47729,0.49316,0.29785,-0.019531,-0.31128,-0.46631,-0.41504,-0.20752,0.11475,0.43091,0.49805,0.48828,0.21973,-0.15503,-0.38452,-0.55176,-0.41016,-0.050049,0.21973,0.41748,0.47729,0.25879,-0.048828,-0.29541,-0.48218,-0.4126,-0.19653,0.14771,0.42358,0.50659,0.42847,0.18066,-0.16113,-0.35767,-0.47363,-0.36133,-0.13184,0.18555,0.47119,0.46997,0.32715,0.053711,-0.30151,-0.52246,-0.43701,-0.25024,0.067139,0.38696,0.53223,0.43457,0.17334,-0.1355,-0.35156,-0.49194,-0.36011,-0.081787,0.19409,0.44678,0.4541,0.29663,0.03418,-0.25757,-0.50293,-0.448,-0.25146,0.0024414,0.4126,0.53711,0.46753,0.22583,-0.043945,-0.36865,-0.53223,-0.354,-0.1062,0.085449,0.43945,0.53589,0.31128,0.073242,-0.21362,-0.48706,-0.44189,-0.19043,0.031738,0.35889,0.49927,0.40771,0.2002,-0.083008,-0.33813,-0.47852,-0.36011,-0.20508,0.12939,0.4248,0.48828,0.33936,0.020752,-0.25879,-0.47607,-0.5249,-0.28442,-0.0061035,0.25391,0.52368,0.52246,0.2124,-0.10254,-0.33691,-0.53589,-0.33447,0.010986,0.2063,0.46631,0.49805,0.36133,0.03418,-0.11719,-0.45654,-0.47729,-0.19043,0.0085449,0.38818,0.48462,0.50659,0.28198,-0.0012207,-0.271,-0.46509,-0.37476,-0.10498,0.18555,0.43457,0.55298,0.40283,0.12207,-0.14526,-0.38452,-0.48462,-0.22827,0.053711,0.38208,0.58716,0.56519,0.34302,0.0097656,-0.25024,-0.41016,-0.37109,-0.12451,0.18066,0.448,0.53223,0.48828,0.17334,-0.15991,-0.37476,-0.45898,-0.32104,0.0073242,0.29541,0.51392,0.5603,0.37598,0.050049,-0.25757,-0.48218,-0.36743,-0.087891,0.11597,0.48096,0.46509,0.33325,0.087891,-0.13428,-0.41992,-0.48706,-0.31738,-0.10132,0.2417,0.50659,0.48828,0.31494,0.040283,-0.28931,-0.45898,-0.43091,-0.26245,0.078125,0.3479,0.4834,0.41504,0.14404,-0.18066,-0.41992,-0.49316,-0.32104,-0.075684,0.21973,0.50537,0.51025,0.31372,0.056152,-0.27466,-0.52246,-0.40405,-0.2124,0.050049,0.39063,0.51758,0.41626,0.18066,-0.092773,-0.37109,-0.5249,-0.36865,-0.098877,0.15259,0.47363,0.49683,0.32715,0.16113,-0.177,-0.44189,-0.38574,-0.14648,0.15991,0.45166,0.54321,0.49072,0.25391,-0.0073242,-0.28687,-0.48218,-0.31982,0.0012207,0.25635,0.57495,0.53711,0.3479,0.037842,-0.18433,-0.46387,-0.54077,-0.1709,0.021973,0.26489,0.51514,0.48584,0.13916,-0.076904,-0.37598,-0.4834,-0.34912,-0.026855,0.23926,0.51636,0.58716,0.43335,0.14771,-0.13306,-0.39429,-0.39429,-0.15137,0.098877,0.40039,0.49683,0.47607,0.20996,-0.073242,-0.30151,-0.5249,-0.35645,-0.023193,0.13916,0.49683,0.52246,0.37109,0.048828,-0.16846,-0.35645,-0.47241,-0.23804,0.13672,0.43701,0.54688,0.51392,0.22461,-0.040283,-0.3772,-0.43701,-0.38574,-0.21362,0.11963,0.41504,0.47729,0.42236,0.11475,-0.17822,-0.42603,-0.50293,-0.22461,-0.020752,0.25391,0.45898,0.47363,0.26367,0.0073242,-0.29663,-0.51025,-0.38696,-0.10742,0.11963,0.4895,0.46875,0.40649,0.15747,-0.19409,-0.40405,-0.44312,-0.29785,-0.024414,0.32593,0.54443,0.48096,0.30762,0.025635,-0.30151,-0.5249,-0.41138,-0.20874,0.096436,0.42236,0.55176,0.40283,0.16602,-0.12695,-0.38696,-0.43701,-0.27466,-0.0024414,0.29541,0.53711,0.52368,0.29663,0.019531,-0.30029,-0.53711,-0.39307,-0.19897,0.06958,0.43945,0.51025,0.41992,0.22095,-0.10498,-0.40527,-0.5188,-0.354,-0.063477,0.20996,0.53223,0.52002,0.25146,0.023193,-0.24902,-0.5481,-0.4541,-0.19775,0.05127,0.36987,0.53345,0.38452,0.13672,-0.10986,-0.37964,-0.5127,-0.29297,-0.073242,0.20264,0.4895,0.53711,0.31738,0.029297,-0.15625,-0.52246,-0.46387,-0.16968,0.026855,0.41016,0.53345,0.44189,0.16113,0.0024414,-0.27588,-0.49927,-0.27222,0.043945,0.25391,0.53223,0.60425,0.36987,0.058594,-0.19653,-0.47363,-0.48706,-0.20142,0.043945,0.3894,0.55908,0.45166,0.19287,-0.1001,-0.34546,-0.49927,-0.39795,-0.1001,0.20142,0.41016,0.50171,0.39307,0.043945,-0.25757,-0.46631,-0.53101,-0.27466,0.037842,0.30762,0.49561,0.45654,0.23071,-0.092773,-0.29053,-0.4834,-0.43335,-0.1123,0.19531,0.4541,0.53223,0.41138,0.062256,-0.22949,-0.41504,-0.47974,-0.33203,-0.040283,0.28076,0.47485,0.45654,0.2417,-0.13672,-0.36133,-0.48096,-0.27954,-0.039063,0.13794,0.48828,0.43701,0.33325,0.16724,-0.12085,-0.46997,-0.48828,-0.24902,-0.043945,0.31738,0.46021,0.40039,0.23193,-0.040283,-0.33325,-0.50171,-0.41992,-0.20874,0.12817,0.4187,0.51025,0.36621,0.11353,-0.16602,-0.4248,-0.4541,-0.28809,-0.056152,0.28076,0.48706,0.42847,0.25269,-0.013428,-0.31738,-0.49805,-0.35889,-0.18188,0.097656,0.44434,0.53467,0.43701,0.16968,-0.15137,-0.4248,-0.50049,-0.31738,-0.048828,0.20264,0.448,0.448,0.24658,-0.0048828,-0.27466,-0.47119,-0.37354,-0.11841,0.14282,0.40283,0.52368,0.39551,0.14771,-0.085449,-0.36499,-0.54688,-0.34424,-0.092773,0.17334,0.47852,0.48096,0.2356,-0.012207,-0.25269,-0.52124,-0.45044,-0.18677,0.05249,0.31006,0.52979,0.42114,0.14282,-0.15869,-0.31738,-0.54077,-0.27832,-0.072021,0.16113,0.39551,0.47485,0.25879,0.012207,-0.25269,-0.49072,-0.45654,-0.24048,0.05127,0.38086,0.53467,0.46265,0.16602,-0.093994,-0.30518,-0.51147,-0.37964,-0.084229,0.14893,0.40039,0.46143,0.30273,-0.03418,-0.28931,-0.48096,-0.44678,-0.19043,0.19531,0.42358,0.52734,0.42847,0.18555,-0.039063,-0.24902,-0.4248,-0.38086,-0.037842,0.24414,0.49561,0.43457,0.3479,0.13794,-0.13184,-0.40283,-0.45166,-0.20996,0.1062,0.37842,0.4834,0.43945,0.20508,-0.1123,-0.32227,-0.37598,-0.27466,-0.0085449,0.15137,0.47607,0.44189,0.32593,0.03418,-0.18921,-0.4248,-0.46875,-0.30273,-0.025635,0.36865,0.46143,0.44067,0.29785,-0.020752,-0.35522,-0.47363,-0.35889,-0.15015,0.17212,0.43213,0.4541,0.32227,0.070801,-0.21362,-0.43945,-0.47119,-0.23926,0.0048828,0.31494,0.48828,0.45044,0.21362,-0.043945,-0.29785,-0.50537,-0.32349,-0.24048,0.048828,0.40039,0.45166,0.30762,0.10376,-0.21362,-0.47607,-0.47729,-0.32227,-0.076904,0.25635,0.50903,0.45288,0.23926,-0.0354,-0.31738,-0.53711,-0.37476,-0.10986,0.11597,0.36865,0.48828,0.354,0.085449,-0.16602,-0.39429,-0.52856,-0.25146,-0.061035,0.22583,0.47607,0.49805,0.2478,0.076904,-0.19165,-0.4895,-0.33325,-0.10986,0.05249,0.32959,0.53711,0.38818,0.10254,-0.18799,-0.4126,-0.55054,-0.29907,-0.021973,0.21851,0.46143,0.49561,0.24536,-0.0097656,-0.22461,-0.4834,-0.45654,-0.22095,0.070801,0.38574,0.48096,0.41992,0.11719,-0.14526,-0.36011,-0.47852,-0.40649,-0.093994,0.19775,0.43945,0.48706,0.31738,-0.041504,-0.24902,-0.43457,-0.45776,-0.20874,0.10986,0.33936,0.46875,0.43579,0.13306,-0.076904,-0.30396,-0.50415,-0.35034,0.010986,0.23193,0.47485,0.45898,0.34668,0.061035,-0.2002,-0.41748,-0.45532,-0.25879,0.067139,0.31006,0.46997,0.40649,0.15625,-0.14038,-0.38452,-0.49438,-0.26611,-0.11963,0.18799,0.47852,0.46875,0.31982,0.054932,-0.25513,-0.42603,-0.4248,-0.26855,-0.019531,0.33203,0.46753,0.42725,0.21362,-0.079346,-0.39551,-0.51025,-0.41992,-0.21606,0.11597,0.36621,0.45654,0.35645,0.073242,-0.24902,-0.43457,-0.44312,-0.21362,0.025635,0.34058,0.48828,0.39551,0.18799,-0.080566,-0.32227,-0.51025,-0.37231,-0.15747,0.10986,0.44067,0.49805,0.34302,0.10254,-0.12207,-0.40894,-0.44189,-0.24902,-0.013428,0.25391,0.5249,0.43457,0.21729,-0.090332,-0.34424,-0.47729,-0.32715,-0.068359,0.10254,0.47729,0.47119,0.32837,0.05127,-0.090332,-0.44678,-0.41504,-0.19653,-0.018311,0.23804,0.47852,0.45532,0.21484,-0.031738,-0.26855,-0.54932,-0.39429,-0.18677,0.05127,0.41992,0.48828,0.36865,0.078125,-0.079346,-0.39673,-0.43701,-0.22095,0.1001,0.32471,0.54199,0.54199,0.32349,0.024414,-0.25269,-0.45776,-0.36743,-0.15015,0.13184,0.47363,0.50537,0.50537,0.20752,-0.080566,-0.26978,-0.44434,-0.28809,0.058594,0.28931,0.53711,0.52979,0.27954,-0.067139,-0.271,-0.46631,-0.42603,-0.21729,0.08667,0.33569,0.46753,0.43701,0.13184,-0.15137,-0.31982,-0.46875,-0.33936,-0.045166,0.2002,0.46021,0.47363,0.28442,0.031738,-0.26245,-0.50049,-0.45654,-0.26123,0.0097656,0.41992,0.43945,0.36865,0.26367,-0.073242,-0.31006,-0.40039,-0.27466,0.026855,0.34912,0.55176,0.45654,0.30029,0.014648,-0.18921,-0.46753,-0.37476,-0.19409,0,0.45654,0.4541,0.40527,0.21729,-0.11108,-0.36499,-0.3833,-0.26123,-0.029297,0.14893,0.5127,0.47363,0.33447,0.05249,-0.17578,-0.48584,-0.41504,-0.23682,0.032959,0.29175,0.45044,0.38574,0.15503,-0.12573,-0.34424,-0.53711,-0.29175,-0.13794,0.1416,0.44678,0.46387,0.32227,0.073242,-0.20508,-0.45654,-0.47119,-0.29053,-0.072021,0.26978,0.49072,0.37842,0.18066,-0.089111,-0.32715,-0.51514,-0.27832,-0.1123,0.10254,0.44312,0.53711,0.354,0.048828,-0.083008,-0.4541,-0.44434,-0.19653,-0.021973,0.26367,0.46387,0.43091,0.22949,-0.056152,-0.31372,-0.50781,-0.3833,-0.13428,0.10254,0.39551,0.49805,0.37598,0.11475,-0.12573,-0.39917,-0.46631,-0.25513,-0.0048828,0.29663,0.46997,0.47363,0.23926,-0.046387,-0.26978,-0.44434,-0.41748,-0.12939,0.15747,0.42969,0.54199,0.45898,0.1355,-0.14404,-0.36621,-0.51147,-0.36621,-0.058594,0.20996,0.37842,0.42603,0.22217,-0.073242,-0.26611,-0.4834,-0.38086,-0.05127,0.095215,0.44678,0.47485,0.36133,0.090332,-0.062256,-0.28687,-0.47363,-0.26611,0.0012207,0.26001,0.51758,0.44678,0.24902,-0.026855,-0.3064,-0.46631,-0.33203,-0.22705,0.096436,0.42725,0.46875,0.41382,0.16113,-0.17456,-0.37109,-0.38574,-0.25146,0.028076,0.33203,0.43457,0.44556,0.28076,0.083008,-0.2002,-0.40283,-0.38818,-0.16846,0.13916,0.448,0.50049,0.43701,0.20508,-0.079346,-0.34424,-0.47485,-0.33447,-0.065918,0.19287,0.46509,0.46021,0.30884,0.015869,-0.24658,-0.45654,-0.40039,-0.22217,0.048828,0.32593,0.47974,0.40894,0.20264,-0.091553,-0.34668,-0.49072,-0.34058,-0.098877,0.16113,0.44678,0.45898,0.31128,0.037842,-0.24658,-0.50781,-0.50415,-0.25879,0.019531,0.28809,0.48584,0.42236,0.17944,-0.058594,-0.28564,-0.45898,-0.34668,-0.10254,0.14771,0.42236,0.50781,0.34302,0.021973,-0.22949,-0.46021,-0.46387,-0.22949,-0.014648,0.32593,0.51514,0.49316,0.23438,-0.037842,-0.2832,-0.50781,-0.36133,-0.12207,0.11963,0.37354,0.46997,0.31738,0.024414,-0.22705,-0.43091,-0.50171,-0.28076,0.03418,0.29785,0.43945,0.46143,0.24536,-0.079346,-0.26855,-0.45166,-0.42725,-0.15015,0.14648,0.38208,0.47363,0.38208,0.050049,-0.10742,-0.43335,-0.47852,-0.2417,0.070801,0.32593,0.46143,0.46631,0.22217,-0.062256,-0.25024,-0.44922,-0.36377,-0.10986,0.15137,0.36987,0.50049,0.40039,0.10254,-0.2002,-0.39185,-0.49805,-0.354,-0.065918,0.28442,0.46265,0.47119,0.31372,-0.014648,-0.28564,-0.43091,-0.35156,-0.19043,0.10376,0.35767,0.46875,0.39551,0.15259,-0.20752,-0.41016,-0.47729,-0.35278,-0.043945,0.28931,0.42236,0.46875,0.30518,-0.03418,-0.28809,-0.46509,-0.35034,-0.21973,0.087891,0.4834,0.44067,0.37598,0.13794,-0.17334,-0.43091,-0.46509,-0.28564,-0.12695,0.15503,0.54932,0.43945,0.24658,0.12695,-0.18555,-0.43091,-0.29785,-0.1123,0.14893,0.46753,0.57617,0.44434,0.21729,-0.075684,-0.34424,-0.4541,-0.31372,-0.025635,0.23926,0.54932,0.55908,0.42847,0.15259,-0.16357,-0.46021,-0.39185,-0.11597,0.098877,0.3894,0.57373,0.42847,0.21484,-0.024414,-0.31982,-0.50049,-0.27222,-0.010986,0.1355,0.50049,0.46997,0.27832,0.048828,-0.20874,-0.448,-0.45898,-0.21729,0.030518,0.35278,0.51514,0.47363,0.21973,-0.090332,-0.33936,-0.48584,-0.36499,-0.11475,0.15869,0.40527,0.5127,0.3479,0.0048828,-0.21729,-0.41992,-0.46997,-0.13794,0.092773,0.32715,0.51025,0.4834,0.23193,-0.070801,-0.25391,-0.47363,-0.42358,-0.12939,0.13916,0.38086,0.53223,0.40527,0.070801,-0.15015,-0.41382,-0.49561,-0.25269,0.045166,0.30273,0.50903,0.46753,0.23193,-0.062256,-0.31738,-0.48462,-0.34668,-0.13916,0.15625,0.37231,0.43457,0.32959,0.059814,-0.22339,-0.40039,-0.46387,-0.33325,-0.010986,0.31128,0.47485,0.46509,0.28564,-0.061035,-0.32959,-0.47607,-0.44922,-0.23682,0.10132,0.36743,0.44556,0.39063,0.095215,-0.2002,-0.37598,-0.43091,-0.26855,0.012207,0.3186,0.50659,0.47363,0.28687,-0.0097656,-0.30762,-0.49927,-0.4126,-0.21484,0.065918,0.45776,0.43213,0.38818,0.12939,-0.036621,-0.39185,-0.43579,-0.24414,0.043945,0.30762,0.55176,0.53955,0.30029,0.048828,-0.28564,-0.49927,-0.38818,-0.2478,0,0.26367,0.32715,0.15869,-0.12329,-0.39795,-0.64209,-0.7373,-0.57129,-0.35278,-0.070801,0.28564,0.33936,0.10864,-0.13306,-0.44922,-0.58228,-0.4541,-0.19653,0.073242,0.36377,0.54199,0.39063,0.19409,-0.090332,-0.33936,-0.44312,-0.2356,0.0354,0.26367,0.52124,0.51758,0.31128,0.10254,-0.16846,-0.39063,-0.3833,-0.21484,0.063477,0.38696,0.51636,0.46021,0.18066,-0.061035,-0.27588,-0.49072,-0.33691,-0.029297,0.19775,0.49194,0.56885,0.33691,0.0073242,-0.19897,-0.4248,-0.45044,-0.19409,0.11841,0.35645,0.49072,0.44922,0.21973,-0.040283,-0.24414,-0.43945,-0.32593,-0.030518,0.19287,0.47363,0.56152,0.43213,0.12451,-0.13184,-0.42114,-0.49438,-0.20874,0.093994,0.31494,0.50049,0.43945,0.19897,-0.061035,-0.27588,-0.45898,-0.32715,-0.070801,0.22217,0.46631,0.51025,0.40527,0.10254,-0.20996,-0.38208,-0.41504,-0.28809,0.023193,0.36987,0.49805,0.50659,0.28564,-0.059814,-0.32349,-0.46997,-0.38574,-0.1416,0.16968,0.4126,0.5542,0.4187,0.1123,-0.19043,-0.41748,-0.44922,-0.27466,0.024414,0.32471,0.47974,0.46509,0.2478,-0.070801,-0.32227,-0.50171,-0.41382,-0.17944,0.12329,0.46387,0.5249,0.43945,0.17578,-0.15503,-0.4248,-0.47485,-0.27344,-0.014648,0.2771,0.49316,0.46387,0.23193,-0.024414,-0.31616,-0.51025,-0.38208,-0.15503,0.13306,0.44189,0.53223,0.40894,0.15137,-0.12695,-0.37109,-0.4541,-0.33325,-0.083008,0.26367,0.52979,0.52246,0.28198,-0.021973,-0.31494,-0.55664,-0.43213,-0.2002,0.053711,0.36865,0.53955,0.40649,0.15625,-0.13672,-0.40283,-0.44556,-0.2063,-0.048828,0.21484,0.45044,0.45898,0.26123,-0.014648,-0.25513,-0.49927,-0.44434,-0.18799,0.1123,0.38208,0.50537,0.43945,0.16357,-0.074463,-0.34546,-0.51025,-0.37476,-0.056152,0.21606,0.48462,0.54565,0.2771,-0.023193,-0.26855,-0.52002,-0.47119,-0.22949,0.061035,0.34668,0.53955,0.44189,0.13794,-0.12207,-0.32593,-0.50537,-0.3064,-0.065918,0.16357,0.44189,0.52368,0.31128,0.0097656,-0.2832,-0.51392,-0.5188,-0.28564,0.024414,0.31982,0.4834,0.448,0.20142,-0.10864,-0.34668,-0.49194,-0.25024,-0.072021,0.21973,0.4187,0.47363,0.32715,0.0024414,-0.22339,-0.53101,-0.49438,-0.23926,0.0073242,0.43701,0.48828,0.47852,0.23193,-0.10498,-0.32837,-0.46387,-0.39917,-0.10498,0.20264,0.43945,0.49805,0.35767,0.063477,-0.23926,-0.46997,-0.48096,-0.26611,0.0061035,0.31738,0.50293,0.44556,0.24658,-0.076904,-0.354,-0.49805,-0.36133,-0.14648,0.15259,0.44434,0.5188,0.41992,0.11108,-0.23438,-0.47852,-0.52124,-0.30518,-0.03418,0.26611,0.48828,0.44189,0.24902,-0.042725,-0.35767,-0.50293,-0.33081,-0.1355,0.14648,0.50171,0.50659,0.32349,0.11841,-0.15991,-0.45776,-0.51514,-0.33936,-0.067139,0.30762,0.53955,0.48828,0.26367,-0.030518,-0.26978,-0.49194,-0.448,-0.18799,0.073242,0.39063,0.55176,0.3894,0.092773,-0.20752,-0.46143,-0.52124,-0.31616,-0.058594,0.22217,0.45776,0.49927,0.24536,-0.024414,-0.2832,-0.55054,-0.42969,-0.13306,0.12329,0.40527,0.50293,0.39551,0.15137,-0.029297,-0.41504,-0.54199,-0.33081,0.048828,0.30396,0.56885,0.52124,0.23682,0.073242,-0.16968,-0.41992,-0.37964,-0.080566,0.12207,0.49805,0.57373,0.44067,0.11841,-0.19043,-0.38574,-0.45898,-0.29053,-0.10986,0.13184,0.448,0.5481,0.31006,0.0097656,-0.29541,-0.49805,-0.42725,-0.20264,0.08667,0.3418,0.50659,0.47241,0.19531,-0.13428,-0.37842,-0.52002,-0.30029,-0.078125,0.23193,0.43823,0.47852,0.33936,0.020752,-0.26978,-0.47363,-0.3894,-0.26855,0.076904,0.53711,0.52246,0.43457,0.18555,-0.030518,-0.35156,-0.45654,-0.3894,-0.063477,0.14404,0.50537,0.50537,0.3125,0.0048828,-0.14771,-0.47852,-0.41504,-0.22949,0.05127,0.41748,0.53955,0.45654,0.21973,-0.1062,-0.37964,-0.49683,-0.38574,-0.16602,0.16602,0.43823,0.45654,0.3418,0.079346,-0.22949,-0.51147,-0.46143,-0.24902,-0.019531,0.29175,0.51758,0.41504,0.22339,-0.063477,-0.37964,-0.55664,-0.36133,-0.068359,0.13428,0.50293,0.49316,0.33936,0.03418,-0.15015,-0.45898,-0.49194,-0.30273,-0.030518,0.36133,0.5249,0.47852,0.27954,-0.05127,-0.31372,-0.48096,-0.39429,-0.15137,0.083008,0.36377,0.57007,0.3833,0.083008,-0.16113,-0.44189,-0.55908,-0.22705,0.0048828,0.271,0.5542,0.52612,0.24414,-0.065918,-0.31494,-0.52734,-0.4248,-0.15137,0.10498,0.40527,0.47729,0.37354,0.080566,-0.16846,-0.43335,-0.5249,-0.32715,-0.059814,0.22339,0.51514,0.4895,0.25146,-0.029297,-0.26123,-0.4834,-0.43335,-0.10498,0.078125,0.42969,0.50415,0.40649,0.092773,-0.19043,-0.42236,-0.50903,-0.36621,-0.0085449,0.25635,0.46143,0.52368,0.32715,-0.019531,-0.29297,-0.448,-0.41138,-0.19043,0.072021,0.29907,0.42236,0.36987,0.13916,-0.18066,-0.41626,-0.47974,-0.28076,0.058594,0.35156,0.45044,0.47607,0.31128,0.0048828,-0.18555,-0.49072,-0.49561,-0.20752,0.076904,0.40283,0.47852,0.41748,0.12207,-0.17578,-0.35645,-0.45044,-0.37964,-0.12085,0.18433,0.48096,0.52979,0.3418,0.03418,-0.26611,-0.46997,-0.41382,-0.20752,0.05127,0.33447,0.49316,0.41992,0.16235,-0.12939,-0.40039,-0.5249,-0.29297,-0.10254,0.19531,0.51025,0.50049,0.36377,0.093994,-0.25635,-0.46875,-0.44556,-0.26367,0.05127,0.35645,0.5127,0.40161,0.18066,-0.093994,-0.3772,-0.50903,-0.33447,-0.12085,0.17578,0.4834,0.50415,0.30884,0.058594,-0.20508,-0.48828,-0.51514,-0.29053,-0.0085449,0.36865,0.51514,0.42969,0.177,-0.13428,-0.39185,-0.56763,-0.4126,-0.14648,0.10254,0.3894,0.5127,0.35034,0.065918,-0.20752,-0.48584,-0.4834,-0.20386,0.0097656,0.271,0.50049,0.42969,0.21118,-0.076904,-0.35522,-0.57617,-0.37476,-0.14771,0.13062,0.45044,0.54077,0.41016,0.068359,-0.14771,-0.40527,-0.51025,-0.26855,0.024414,0.2478,0.4834,0.45654,0.2124,-0.092773,-0.32837,-0.54199,-0.43701,-0.18066,0.095215,0.43213,0.5249,0.42847,0.10254,-0.17822,-0.41138,-0.49805,-0.31372,-0.065918,0.22949,0.4834,0.48218,0.25146,-0.063477,-0.31372,-0.49561,-0.45776,-0.17578,0.14648,0.3479,0.4834,0.41626,0.10864,-0.18433,-0.39307,-0.46875,-0.2356,-0.0036621,0.30762,0.46387,0.44678,0.25635,-0.048828,-0.32715,-0.52246,-0.45898,-0.15503,0.05249,0.49927,0.51025,0.40283,0.1001,-0.084229,-0.31738,-0.46265,-0.31372,-0.0048828,0.18433,0.57739,0.54932,0.25391,-0.05127,-0.33325,-0.52002,-0.41626,-0.21484,0.057373,0.36499,0.48828,0.40039,0.18311,-0.16968,-0.43213,-0.50293,-0.31616,-0.092773,0.20996,0.46265,0.448,0.29785,0.041504,-0.33447,-0.5835,-0.50171,-0.22217,0.029297,0.40649,0.4834,0.36133,0.10986,-0.050049,-0.34912,-0.44312,-0.26367,0.048828,0.34302,0.48096,0.51025,0.31128,-0.025635,-0.271,-0.49072,-0.45898,-0.271,0.028076,0.37964,0.52002,0.46875,0.19287,-0.074463,-0.35889,-0.49561,-0.33081,-0.076904,0.13306,0.43701,0.54565,0.35034,0.026855,-0.26123,-0.5188,-0.55908,-0.15137,0.063477,0.29907,0.47241,0.42969,0.17822,-0.084229,-0.35278,-0.50537,-0.31738,-0.029297,0.19043,0.53955,0.48218,0.34668,0.12817,-0.14404,-0.41748,-0.50537,-0.22583,0.057373,0.35889,0.53345,0.45654,0.16113,-0.10498,-0.34424,-0.52002,-0.31372,-0.12207,0.16113,0.4541,0.53101,0.38086,0.067139,-0.24902,-0.45654,-0.49805,-0.32959,-0.031738,0.30762,0.42358,0.43701,0.28809,-0.064697,-0.36865,-0.44189,-0.37109,-0.092773,0.16846,0.48828,0.47974,0.48096,0.1709,-0.15137,-0.37354,-0.49683,-0.22949,0.065918,0.35645,0.56274,0.47119,0.22217,0.036621,-0.2124,-0.51392,-0.37231,-0.081787,0.1416,0.52979,0.50903,0.38086,0.090332,-0.081787,-0.42969,-0.47363,-0.26733,-0.024414,0.20386,0.47607,0.42969,0.23071,-0.013428,-0.36499,-0.56396,-0.35889,-0.16968,0.096436,0.42603,0.51636,0.39551,0.10376,-0.19897,-0.43945,-0.5188,-0.24414,-0.029297,0.22705,0.44434,0.46143,0.30518,-0.019531,-0.30029,-0.41992,-0.37231,-0.14648,0.068359,0.4248,0.46143,0.36987,0.22217,-0.046387,-0.32471,-0.53589,-0.30396,-0.0048828,0.29053,0.56519,0.48096,0.20508,0.032959,-0.2002,-0.50903,-0.41626,-0.14893,0.1355,0.46509,0.50415,0.43457,0.10742,-0.073242,-0.35278,-0.5127,-0.31616,-0.1062,0.11719,0.38818,0.49927,0.29907,0.043945,-0.2771,-0.53467,-0.45532,-0.13916,0.087891,0.39673,0.50537,0.45654,0.17334,-0.092773,-0.35645,-0.5603,-0.3894,-0.083008,0.17822,0.42725,0.47607,0.30273,0.0024414,-0.18555,-0.40894,-0.47241,-0.21729,0.12451,0.41748,0.5835,0.54199,0.28076,-0.030518,-0.23926,-0.4126,-0.29297,-0.041504,0.16846,0.54199,0.50293,0.31738,0.14282,-0.22217,-0.42725,-0.41504,-0.19043,0.098877,0.44434,0.49561,0.48218,0.25391,-0.1001,-0.37598,-0.51514,-0.354,-0.14404,0.16113,0.35156,0.46265,0.3418,0.0354,-0.24292,-0.4541,-0.51758,-0.26733,0.020752,0.32349,0.48584,0.43701,0.26611,-0.043945,-0.29175,-0.52979,-0.39551,-0.19409,0.11108,0.42847,0.48706,0.31738,0.079346,-0.1355,-0.50171,-0.45898,-0.21973,0.054932,0.33203,0.60425,0.44678,0.33936,0.03418,-0.23438,-0.42847,-0.28564,-0.1001,0.16357,0.44434,0.49316,0.38208,0.12939,-0.17822,-0.4541,-0.47607,-0.26245,0.024414,0.34912,0.57373,0.5542,0.37598,0.089111,-0.25146,-0.52002,-0.37842,-0.081787,0.15381,0.41504,0.56274,0.43457,0.13916,-0.081787,-0.35278,-0.47852,-0.21851,0.083008,0.33325,0.49316,0.49805,0.26855,0.11108,-0.18555,-0.44556,-0.3894,-0.16602,0.1355,0.48828,0.57373,0.37842,0.1001,-0.10254,-0.39673,-0.5249,-0.354,-0.056152,0.2002,0.53345,0.5835,0.27832,-0.0024414,-0.27588,-0.47852,-0.38086,-0.11841,0.10742,0.34302,0.47607,0.43945,0.15625,-0.16113,-0.35889,-0.51514,-0.38086,-0.075684,0.20508,0.44189,0.53833,0.3479,0.059814,-0.2124,-0.50171,-0.49194,-0.24658,0.084229,0.36865,0.52246,0.38818,0.12207,-0.18311,-0.35522,-0.50415,-0.35156,-0.056152,0.23193,0.44678,0.52979,0.31738,0.018311,-0.24414,-0.39795,-0.39551,-0.23926,0.072021,0.39551,0.49072,0.50293,0.25757,-0.11841,-0.38086,-0.54443,-0.42725,-0.087891,0.18677,0.44922,0.51758,0.34058,0.048828,-0.24902,-0.48584,-0.46631,-0.18799,0.089111,0.3418,0.49561,0.40649,0.18188,-0.083008,-0.34424,-0.54199,-0.39795,-0.16846,0.1416,0.47607,0.50293,0.37598,0.12329,-0.19897,-0.45044,-0.45166,-0.30762,-0.031738,0.33325,0.52734,0.4248,0.2417,-0.11719,-0.37842,-0.5188,-0.36987,-0.14282,0.14893,0.43579,0.4834,0.34668,0.037842,-0.20996,-0.45044,-0.4834,-0.25757,-0.0048828,0.30029,0.49316,0.51147,0.26489,-0.074463,-0.37109,-0.57739,-0.4895,-0.17822,0.10254,0.36865,0.49316,0.34302,0.056152,-0.19653,-0.46997,-0.50171,-0.24902,-0.014648,0.31494,0.52124,0.45044,0.18921,-0.040283,-0.31494,-0.52612,-0.43701,-0.17456,0.067139,0.41138,0.52246,0.40771,0.1123,-0.16479,-0.39429,-0.48828,-0.3186,-0.0073242,0.28076,0.50293,0.52002,0.25269,-0.092773,-0.33203,-0.51514,-0.42358,-0.16968,0.092773,0.38086,0.51514,0.38086,0.13184,-0.17822,-0.36621,-0.47607,-0.30396,-0.029297,0.24292,0.4834,0.53467,0.32349,-0.014648,-0.31006,-0.53223,-0.47607,-0.22217,0.10742,0.35034,0.47363,0.40405,0.14404,-0.14648,-0.34302,-0.48096,-0.36255,-0.025635,0.29663,0.49805,0.4834,0.271,-0.037842,-0.2478,-0.4541,-0.46265,-0.29297,0.015869,0.40283,0.52734,0.42236,0.177,-0.13428,-0.38452,-0.47119,-0.34302,-0.070801,0.19409,0.45776,0.50781,0.32471,0.015869,-0.27344,-0.52246,-0.43701,-0.21484,0.073242,0.39185,0.48584,0.39551,0.20996,-0.11353,-0.41748,-0.46997,-0.36255,-0.10254,0.1709,0.49927,0.47852,0.30029,0.070801,-0.23071,-0.52002,-0.48706,-0.27832,-0.029297,0.31982,0.47852,0.39917,0.16479,-0.12207,-0.34912,-0.4834,-0.37598,-0.085449,0.25024,0.50781,0.53955,0.34302,0.013428,-0.23438,-0.46265,-0.49438,-0.26855,-0.029297,0.35034,0.52368,0.46143,0.22339,-0.045166,-0.31982,-0.51147,-0.35156,-0.070801,0.17212,0.44556,0.55176,0.34668,0.070801,-0.19409,-0.46509,-0.52856,-0.24658,0.0085449,0.27832,0.46753,0.41504,0.1709,-0.08667,-0.31372,-0.52124,-0.41748,-0.15991,0.15015,0.44922,0.51392,0.36377,0.053711,-0.19653,-0.45898,-0.52124,-0.29419,-0.061035,0.26001,0.52734,0.46997,0.24292,-0.080566,-0.32715,-0.49316,-0.38818,-0.11108,0.1355,0.38574,0.51514,0.41626,0.096436,-0.18799,-0.42114,-0.5127,-0.33813,-0.045166,0.29785,0.45654,0.47119,0.25879,-0.089111,-0.32593,-0.50293,-0.45898,-0.20142,0.085449,0.33447,0.49316,0.41626,0.10498,-0.1709,-0.39673,-0.55176,-0.32959,-0.0061035,0.27588,0.45898,0.49316,0.23193,-0.075684,-0.28687,-0.51758,-0.44678,-0.23193,0.075684,0.41138,0.50415,0.43091,0.16113,-0.16602,-0.40894,-0.4834,-0.38574,-0.11108,0.21484,0.45044,0.44067,0.25879,-0.05127,-0.31128,-0.50903,-0.44312,-0.20996,0.085449,0.38818,0.52002,0.44678,0.18188,-0.095215,-0.37598,-0.52979,-0.3894,-0.098877,0.22705,0.48096,0.448,0.2832,0.020752,-0.31738,-0.48096,-0.42236,-0.23926,0.061035,0.36743,0.47974,0.42725,0.177,-0.11719,-0.38818,-0.48218,-0.34424,-0.089111,0.18677,0.43701,0.48462,0.28442,-0.013428,-0.27954,-0.52002,-0.50415,-0.25757,0.0073242,0.354,0.5249,0.44189,0.18677,-0.083008,-0.3418,-0.52246,-0.36621,-0.10742,0.14648,0.40039,0.47363,0.26611,0.021973,-0.2478,-0.49438,-0.50049,-0.26855,0.01709,0.35034,0.50537,0.42603,0.19531,-0.098877,-0.33203,-0.51514,-0.38818,-0.1355,0.1355,0.41382,0.49561,0.35522,0.010986,-0.26123,-0.52246,-0.54199,-0.177,0.024414,0.25879,0.48706,0.46631,0.23804,-0.080566,-0.29785,-0.49683,-0.38818,-0.12939,0.16113,0.44067,0.49438,0.34424,0.054932,-0.20996,-0.47119,-0.50415,-0.30884,-0.0036621,0.35034,0.55786,0.48584,0.24902,-0.073242,-0.30762,-0.47363,-0.4248,-0.14648,0.1123,0.34424,0.50049,0.38574,0.031738,-0.25635,-0.43823,-0.54932,-0.20142,-0.028076,0.28564,0.47119,0.45288,0.25024,-0.080566,-0.34668,-0.49316,-0.40649,-0.17334,0.10498,0.43579,0.48828,0.37964,0.083008,-0.18433,-0.44678,-0.51636,-0.38574,-0.084229,0.26611,0.46509,0.46631,0.26245,-0.073242,-0.31738,-0.48096,-0.41626,-0.18921,0.14282,0.42725,0.48584,0.39551,0.11841,-0.22461,-0.46143,-0.54199,-0.2771,-0.096436,0.22217,0.4541,0.46631,0.26611,0,-0.30029,-0.49561,-0.32715,-0.19287,0.087891,0.3833,0.52002,0.36255,0.11963,-0.20264,-0.42236,-0.54443,-0.28809,-0.065918,0.26855,0.47852,0.50293,0.28809,-0.0024414,-0.2356,-0.46997,-0.43213,-0.22705,0.061035,0.38818,0.51636,0.42603,0.15137,-0.13062,-0.42358,-0.56274,-0.30884,-0.096436,0.14771,0.50293,0.50659,0.27588,0.047607,-0.271,-0.51514,-0.43823,-0.19775,0.080566,0.39063,0.5249,0.41748,0.13428,-0.15747,-0.37476,-0.52368,-0.30396,-0.13428,0.16479,0.45654,0.48096,0.34912,0.031738,-0.25757,-0.49316,-0.47363,-0.26245,0.0354,0.31128,0.54443,0.46021,0.17944,-0.13428,-0.38818,-0.58105,-0.32349,-0.075684,0.15381,0.42236,0.49438,0.29541,-0.0048828,-0.25146,-0.49805,-0.49927,-0.27832,0.040283,0.35156,0.46997,0.42847,0.20142,-0.083008,-0.32471,-0.51514,-0.36865,-0.19409,0.095215,0.40405,0.5127,0.36133,0.042725,-0.24414,-0.46265,-0.50049,-0.24902,0.045166,0.32227,0.56152,0.47852,0.2063,-0.10254,-0.39307,-0.52612,-0.42725,-0.14893,0.084229,0.39063,0.46875,0.38452,0.10498,-0.2002,-0.43457,-0.46143,-0.28198,-0.018311,0.3186,0.49561,0.42969,0.26001,-0.043945,-0.33813,-0.49072,-0.3894,-0.18921,0.1709,0.43701,0.46509,0.37598,0.061035,-0.23315,-0.40527,-0.50537,-0.31372,-0.036621,0.30273,0.4834,0.44922,0.22339,-0.064697,-0.30762,-0.51392,-0.37354,-0.25024,0.045166,0.32837,0.48462,0.34668,0.096436,-0.19287,-0.4541,-0.54199,-0.20508,-0.023193,0.27588,0.53467,0.49805,0.26001,-0.021973,-0.32471,-0.55786,-0.35645,-0.19897,0.070801,0.38086,0.4834,0.35278,0.10254,-0.15869,-0.40527,-0.5249,-0.33325,-0.05127,0.18799,0.49805,0.50903,0.27588,0.024414,-0.27222,-0.54443,-0.38452,-0.21973,0.078125,0.41016,0.48096,0.36743,0.10498,-0.19165,-0.39063,-0.48706,-0.36133,-0.024414,0.23804,0.50293,0.5188,0.29053,-0.024414,-0.25146,-0.43457,-0.43457,-0.23315,0.041504,0.26367,0.46265,0.42236,0.13306,-0.15259,-0.32715,-0.48706,-0.34424,-0.015869,0.19775,0.44922,0.50049,0.29663,0,-0.19653,-0.51392,-0.42603,-0.23071,0.064697,0.37109,0.41748,0.37476,0.1355,-0.14404,-0.37231,-0.50537,-0.39795,-0.10132,0.17944,0.39917,0.48096,0.3064,0.015869,-0.24536,-0.44189,-0.47363,-0.27466,0.01709,0.33081,0.45898,0.44189,0.18677,-0.16113,-0.36011,-0.47974,-0.36133,-0.12451,0.1709,0.44678,0.5127,0.37354,0.06958,-0.2478,-0.48706,-0.4248,-0.25024,0,0.24048,0.42236,0.35034,0.17212,-0.1062,-0.36255,-0.50659,-0.36621,-0.12939,0.16357,0.42114,0.47241,0.36743,0.079346,-0.23193,-0.44434,-0.4895,-0.23193,-0.021973,0.36377,0.46143,0.41138,0.18311,-0.096436,-0.33447,-0.48828,-0.3894,-0.15137,0.10498,0.43213,0.52002,0.3418,0.06958,-0.17334,-0.39429,-0.46143,-0.25146,-0.024414,0.27222,0.46387,0.44189,0.20264,-0.093994,-0.32349,-0.53223,-0.31982,-0.15747,0.097656,0.41748,0.4895,0.34302,0.13184,-0.13184,-0.44922,-0.46875,-0.25024,-0.024414,0.2417,0.49316,0.4248,0.19043,-0.029297,-0.29785,-0.53711,-0.30762,-0.15381,0.13428,0.38574,0.48218,0.35889,0.085449,-0.17456,-0.39429,-0.49072,-0.36621,-0.024414,0.27344,0.448,0.44434,0.20752,-0.10986,-0.3125,-0.50293,-0.45044,-0.20752,0.024414,0.28809,0.51025,0.41504,0.063477,-0.15991,-0.34546,-0.46997,-0.28076,-0.013428,0.19287,0.4126,0.4895,0.23438,-0.063477,-0.29541,-0.51147,-0.38208,-0.2002,0.085449,0.41016,0.47852,0.43823,0.20508,-0.10132,-0.36133,-0.47852,-0.36621,-0.043945,0.25146,0.45166,0.43945,0.22949,0.0024414,-0.25513,-0.47241,-0.45654,-0.24902,0.085449,0.38086,0.45898,0.39307,0.16357,-0.13794,-0.28442,-0.41016,-0.36377,-0.1416,0.15259,0.4248,0.49561,0.30396,-0.0048828,-0.30029,-0.51636,-0.37842,-0.23193,0.054932,0.30518,0.45532,0.40039,0.17334,-0.15015,-0.40039,-0.49561,-0.22339,-0.073242,0.19775,0.41504,0.41626,0.29175,0.072021,-0.26367,-0.5127,-0.44556,-0.29541,-0.0024414,0.36987,0.48462,0.41748,0.19165,-0.053711,-0.31494,-0.48584,-0.40039,-0.12207,0.1709,0.44434,0.51514,0.30884,0.0036621,-0.23926,-0.4541,-0.43335,-0.22461,-0.010986,0.32104,0.52612,0.4187,0.1709,-0.097656,-0.3418,-0.47363,-0.30884,-0.1123,0.064697,0.41504,0.50049,0.36499,0.054932,-0.21606,-0.52124,-0.54199,-0.19531,-0.029297,0.25391,0.44067,0.3894,0.19531,-0.056152,-0.30518,-0.46265,-0.35645,-0.10742,0.19409,0.43457,0.45898,0.30518,0.036621,-0.18555,-0.3894,-0.50171,-0.33447,-0.029297,0.26489,0.4834,0.50049,0.22949,-0.05127,-0.22095,-0.43579,-0.4126,-0.14648,0.11963,0.33203,0.54688,0.4187,0.062256,-0.22827,-0.4126,-0.49072,-0.28809,0.0085449,0.24414,0.46509,0.46753,0.21973,-0.083008,-0.34668,-0.5957,-0.41748,-0.17822,0.15869,0.56152,0.65063,0.52368,0.2002,-0.22949,-0.5249,-0.55908,-0.40039,-0.062256,0.39795,0.65063,0.63477,0.39307,-0.043945,-0.39551,-0.56763,-0.46631,-0.22583,0.21851,0.54199,0.56519,0.4895,0.17212,-0.26001,-0.44434,-0.56152,-0.354,-0.061035,0.28076,0.55176,0.62744,0.38452,0.0024414,-0.3772,-0.60547,-0.46387,-0.23804,0.12329,0.47485,0.68115,0.52856,0.20508,-0.21851,-0.53467,-0.61279,-0.33936,-0.043945,0.35889,0.63232,0.61279,0.34668,-0.021973,-0.38086,-0.58228,-0.51147,-0.21484,0.092773,0.50659,0.62988,0.53101,0.22705,-0.16724,-0.46509,-0.58838,-0.41992,-0.18066,0.24536,0.5542,0.60425,0.36621,-0.0024414,-0.39307,-0.59937,-0.53101,-0.18555,0.1123,0.49072,0.63965,0.5127,0.20508,-0.17212,-0.45898,-0.61768,-0.40039,-0.12207,0.22827,0.54443,0.59082,0.39795,0.054932,-0.30029,-0.60913,-0.5127,-0.21362,0.054932,0.4248,0.6665,0.53955,0.26489,-0.10254,-0.46387,-0.61401,-0.38086,-0.14648,0.26855,0.58228,0.59448,0.40649,0.024414,-0.36377,-0.56885,-0.5603,-0.23193,0.043945,0.40527,0.59937,0.57861,0.23682,-0.13184,-0.40405,-0.61401,-0.45898,-0.18433,0.21118,0.48462,0.60547,0.45166,0.08667,-0.31494,-0.61035,-0.60913,-0.25024,-0.028076,0.39551,0.63965,0.50659,0.25757,-0.12207,-0.43335,-0.62622,-0.43091,-0.15137,0.22583,0.54321,0.64087,0.46387,0.050049,-0.31738,-0.55908,-0.57373,-0.3064,-0.043945,0.34668,0.57861,0.57129,0.34668,-0.041504,-0.40527,-0.56885,-0.34668,-0.1416,0.21362,0.55908,0.67383,0.49561,0.12207,-0.2832,-0.58105,-0.66528,-0.32715,-0.013428,0.37842,0.61523,0.58105,0.33325,-0.061035,-0.38818,-0.59937,-0.47241,-0.24902,0.14648,0.4248,0.56763,0.43457,0.095215,-0.29053,-0.56641,-0.64697,-0.37231,-0.10132,0.26489,0.57373,0.56885,0.271,-0.098877,-0.41138,-0.62866,-0.52734,-0.15747,0.12207,0.50049,0.61523,0.5249,0.17578,-0.23926,-0.52124,-0.57861,-0.37964,-0.1001,0.27588,0.55908,0.56885,0.30518,-0.05249,-0.39063,-0.62256,-0.49805,-0.14282,0.18311,0.52734,0.6665,0.53223,0.17944,-0.18188,-0.43213,-0.56519,-0.38574,-0.061035,0.29785,0.58105,0.59082,0.35278,-0.018311,-0.38086,-0.64575,-0.53101,-0.25757,0.029297,0.38696,0.60181,0.46387,0.14282,-0.23682,-0.49316,-0.60303,-0.32837,-0.085449,0.27222,0.55664,0.58472,0.38574,0.01709,-0.40771,-0.63599,-0.60059,-0.30762,0.029297,0.42114,0.54932,0.52246,0.2124,-0.20386,-0.49561,-0.66284,-0.45166,-0.13306,0.25757,0.52368,0.61646,0.39307,0.036621,-0.3418,-0.57129,-0.49438,-0.25879,0.10254,0.46509,0.65918,0.55908,0.26978,-0.1001,-0.46875,-0.64941,-0.47485,-0.097656,0.21484,0.54932,0.61035,0.448,0.026855,-0.33325,-0.57129,-0.50659,-0.23926,0.061035,0.40161,0.62134,0.55786,0.28809,-0.12695,-0.39795,-0.59204,-0.38086,-0.13672,0.22217,0.50293,0.59692,0.40894,0.03418,-0.34912,-0.62744,-0.68359,-0.31494,-0.024414,0.3833,0.63477,0.5542,0.29053,-0.074463,-0.3833,-0.58105,-0.41748,-0.18799,0.22827,0.50537,0.60303,0.44922,0.087891,-0.29175,-0.50415,-0.55542,-0.26611,-0.0024414,0.36621,0.61035,0.56885,0.29053,-0.10254,-0.44922,-0.63965,-0.4895,-0.22705,0.14648,0.55054,0.67139,0.5249,0.13062,-0.26611,-0.53955,-0.56396,-0.29541,-0.0024414,0.36621,0.61401,0.54443,0.28442,-0.1001,-0.4541,-0.65308,-0.48218,-0.16602,0.11475,0.42236,0.52246,0.40161,0.10986,-0.27344,-0.53711,-0.66772,-0.46753,-0.11475,0.31128,0.59814,0.62378,0.34912,-0.053711,-0.39063,-0.62622,-0.53345,-0.27832,0.10498,0.41504,0.60913,0.47119,0.12695,-0.26611,-0.54565,-0.63599,-0.3418,-0.057373,0.31006,0.59326,0.58472,0.32349,-0.0048828,-0.3833,-0.60791,-0.47363,-0.26245,0.15259,0.5249,0.6189,0.55786,0.2417,-0.1709,-0.46387,-0.60669,-0.40894,-0.12451,0.27344,0.57739,0.6311,0.34424,-0.031738,-0.41626,-0.6543,-0.54443,-0.21362,0.085449,0.42969,0.5835,0.47241,0.16113,-0.2002,-0.46875,-0.58594,-0.39917,-0.12207,0.24902,0.55786,0.61035,0.43823,0.070801,-0.32715,-0.62134,-0.56641,-0.27588,0.053711,0.40771,0.63721,0.54932,0.22949,-0.18066,-0.44189,-0.62256,-0.35645,-0.075684,0.31616,0.59448,0.64331,0.4248,0.026855,-0.32959,-0.54688,-0.48828,-0.1709,0.070801,0.47607,0.6311,0.57129,0.29175,-0.080566,-0.4126,-0.59082,-0.43091,-0.16235,0.21484,0.50781,0.60425,0.448,0.047607,-0.34912,-0.60913,-0.61646,-0.22949,0.046387,0.40894,0.59814,0.49194,0.24414,-0.14648,-0.47852,-0.63843,-0.50293,-0.19409,0.2124,0.52979,0.60791,0.44556,0.063477,-0.29541,-0.5603,-0.6311,-0.28564,-0.010986,0.35767,0.62622,0.57373,0.26367,-0.11353,-0.43701,-0.62256,-0.39429,-0.16113,0.21851,0.54565,0.64575,0.47363,0.12939,-0.28198,-0.57495,-0.58105,-0.31494,-0.01709,0.37476,0.62378,0.58472,0.32104,-0.080566,-0.41504,-0.60669,-0.46387,-0.19775,0.2002,0.46387,0.5835,0.46509,0.11475,-0.24658,-0.47119,-0.625,-0.31372,-0.043945,0.33081,0.57739,0.55298,0.25513,-0.1001,-0.47485,-0.69458,-0.51758,-0.1709,0.090332,0.51025,0.60059,0.46509,0.14771,-0.26367,-0.54199,-0.5896,-0.39551,-0.06958,0.30762,0.57373,0.58594,0.35156,-0.040283,-0.40161,-0.62622,-0.49438,-0.1709,0.15625,0.50659,0.64209,0.5249,0.15137,-0.26367,-0.51392,-0.61035,-0.37598,-0.05249,0.28809,0.58105,0.63599,0.37842,0.025635,-0.33447,-0.62134,-0.52368,-0.17578,0.10498,0.43213,0.63354,0.49194,0.18921
