# Supplementary material for: Removal of Dust Microelectric Signal Based on Empirical Mode Decomposition and Multifractal Detrended Fluctuation Analysis
Source: Comput Intell Neurosci. 2021 Aug 6;2021:5468514. doi: 10.1155/2021/5468514 (PMC8367589; doi:10.1155/2021/5468514)
Supplement: Supplementary Materials — “Measured_noise_data.docx” includes the measured noise used in this paper. “Measured_signal_data.docx” includes the measured signal used in this paper. [file 5468514.f1.zip › 5468514.f1/measured_signal_data.docx]

The following data is the measured signal in this paper, the sampling rate is 500Hz, and the unit is Voltage (V).

0.025635,0.078125,0.081787,0.045166,-0.037842,-0.0073242,-0.10376,-0.13672,-0.11719,-0.19043,-0.1062,-0.067139,-0.0354,-0.036621,-0.045166,0.05249,0.029297,0.081787,0.059814,0.15137,0.23071,0.27222,0.24658,0.16968,0.11841,0.17822,0.17212,0.13672,0.20752,0.32227,0.42847,0.44434,0.40405,0.37231,0.46631,0.46143,0.51758,0.46509,0.5188,0.60181,0.61768,0.58838,0.53955,0.48462,0.5481,0.54688,0.48828,0.53833,0.57495,0.59814,0.57617,0.49683,0.39673,0.44434,0.37231,0.39307,0.33691,0.40894,0.45654,0.4834,0.46997,0.40894,0.34424,0.39551,0.33936,0.36499,0.30518,0.3479,0.36743,0.33081,0.22827,0.091553,0.089111,-0.031738,-0.056152,-0.13672,-0.068359,0.0036621,0.042725,0.031738,-0.023193,-0.041504,0.076904,0.056152,0.098877,0.040283,0.070801,0.084229,0.025635,-0.10376,-0.25513,-0.24902,-0.32959,-0.33936,-0.28442,-0.32593,-0.22217,-0.16968,-0.1355,-0.1416,-0.042725,-0.056152,-0.029297,0.057373,0.061035,0.18311,0.26611,0.2832,0.24048,0.16602,0.19897,0.12695,0.10986,0.13062,0.025635,0.020752,-0.036621,-0.097656,-0.21362,-0.21362,-0.3186,-0.40161,-0.46753,-0.51514,-0.67749,-0.74951,-0.85327,-0.95459,-0.9436,-0.9729,-0.94971,-0.87158,-0.78125,-0.66528,-0.62744,-0.5542,-0.54321,-0.47119,-0.51514,-0.54932,-0.5542,-0.51758,-0.44312,-0.45898,-0.36865,-0.32837,-0.34058,-0.25146,-0.27466,-0.29297,-0.24414,-0.18555,-0.12451,-0.19409,-0.17334,-0.16479,-0.17456,-0.061035,-0.064697,-0.048828,0.018311,0.11597,0.18921,0.12695,0.10742,0.032959,0.084229,0.019531,-0.048828,-0.0036621,0.070801,0.11597,0.065918,0.079346,0.029297,0.1001,0.075684,0.079346,0.095215,0.16724,0.25391,0.30151,0.19409,0.15259,0.041504,0.0073242,-0.14648,-0.26611,-0.31982,-0.30151,-0.25391,-0.30273,-0.22705,-0.17578,-0.15747,-0.032959,0.0036621,0.045166,0.15259,0.28076,0.26489,0.33325,0.36255,0.31982,0.35034,0.26733,0.19165,0.21729,0.32593,0.29663,0.38696,0.41748,0.39185,0.31738,0.34912,0.26733,0.19653,0.18188,0.22217,0.13428,0.1416,0.13794,0.062256,0.084229,0.012207,-0.048828,-0.05249,0.013428,-0.0354,0.080566,0.1416,0.15869,0.12695,0.18799,0.14038,0.095215,0.10132,0.14282,0.06958,0.12085,0.13916,0.11841,0.05249,0.11475,0.065918,0.056152,0.11597,0.091553,0.19897,0.28687,0.32715,0.31982,0.27222,0.36255,0.35034,0.40283,0.48706,0.45288,0.52124,0.53345,0.52002,0.46997,0.52368,0.46509,0.45532,0.4834,0.43335,0.52368,0.55908,0.5481,0.50537,0.41992,0.44189,0.37109,0.32471,0.32227,0.20508,0.19775,0.15015,0.029297,-0.12207,-0.13428,-0.25635,-0.30518,-0.28198,-0.34424,-0.24902,-0.16724,-0.13672,-0.15869,-0.19775,-0.12329,-0.13428,-0.081787,-0.12939,-0.040283,0.03418,0.037842,-0.0061035,-0.076904,-0.17212,-0.15747,-0.18799,-0.15381,-0.22339,-0.16357,-0.10376,-0.095215,-0.13306,-0.17578,-0.12207,-0.19043,-0.16968,-0.11108,-0.16846,-0.046387,0.05249,0.081787,0.11108,0.25024,0.2478,0.2832,0.34546,0.29175,0.36377,0.4126,0.40039,0.35522,0.28931,0.34912,0.31372,0.32959,0.38818,0.32959,0.36987,0.41016,0.39429,0.32227,0.37476,0.32471,0.29297,0.34546,0.30884,0.35889,0.39795,0.40405,0.33569,0.25269,0.30273,0.21729,0.21362,0.26611,0.2063,0.26733,0.2832,0.26611,0.22461,0.29297,0.24414,0.21484,0.25391,0.33325,0.29175,0.33936,0.33325,0.26245,0.29175,0.19653,0.11597,0.091553,0.11353,0.16602,0.072021,0.090332,0.048828,-0.026855,0.021973,-0.042725,-0.058594,0,0.079346,0.042725,0.12085,0.1416,0.11963,0.15747,0.061035,-0.013428,-0.037842,-0.021973,-0.1001,-0.074463,-0.065918,-0.1001,-0.16724,-0.12695,-0.18433,-0.21606,-0.17334,-0.085449,-0.11719,-0.039063,-0.025635,-0.032959,-0.074463,-0.0085449,-0.058594,-0.050049,0,0.06958,0.031738,0.059814,0.05127,0.010986,0.080566,0.0354,0.0048828,0.036621,0.097656,0.0354,0.083008,0.072021,0.014648,0.050049,-0.025635,-0.041504,0.013428,0.10864,0.22827,0.23071,0.31616,0.32837,0.29663,0.33447,0.22705,0.15015,0.13672,0.13916,0.041504,0.080566,0.068359,0.013428,0.05127,-0.0048828,-0.058594,-0.040283,0.070801,0.16602,0.16602,0.28564,0.34302,0.35645,0.46265,0.43579,0.41626,0.46509,0.52368,0.47852,0.54077,0.53101,0.47852,0.38452,0.40039,0.29419,0.22461,0.20752,0.19409,0.1062,0.10498,0.05249,-0.024414,-0.068359,-0.0061035,-0.025635,0.046387,0.13062,0.087891,0.16235,0.21851,0.19409,0.12695,0.17334,0.1001,0.042725,0.057373,-0.046387,-0.023193,-0.013428,-0.019531,-0.064697,-0.10742,-0.043945,-0.085449,-0.065918,-0.015869,-0.089111,-0.042725,-0.036621,-0.063477,-0.12329,-0.17578,-0.087891,-0.092773,-0.025635,-0.036621,0.05249,0.11719,0.13428,0.097656,0.050049,0.15503,0.14404,0.19165,0.27954,0.23804,0.29297,0.30396,0.26367,0.17334,0.20142,0.13428,0.076904,0.087891,0.030518,0.085449,0.13672,0.15137,0.12817,0.090332,0.18677,0.18188,0.22949,0.30762,0.26978,0.33936,0.38208,0.36255,0.29907,0.3418,0.27954,0.23071,0.23926,0.15869,0.17578,0.19287,0.1709,0.11108,0.036621,0.097656,0.068359,0.11475,0.18677,0.14038,0.20508,0.20142,0.1416,0.048828,0.081787,0.0085449,-0.025635,-0.0061035,-0.084229,-0.0354,0.028076,0.013428,-0.032959,-0.072021,-0.029297,-0.080566,-0.062256,-0.023193,-0.098877,-0.029297,-0.0061035,-0.021973,-0.064697,0.0073242,-0.036621,-0.028076,0.03418,0.10254,0.062256,0.13794,0.14648,0.096436,0.0073242,0.015869,-0.075684,-0.078125,-0.040283,-0.10132,-0.037842,0.0097656,0.0036621,-0.019531,0.078125,0.032959,0.012207,0.068359,0.14893,0.084229,0.13428,0.1416,0.084229,0.13428,0.062256,0.0097656,0.0073242,0.065918,0.13672,0.10132,0.13428,0.13306,0.06958,0.11841,0.046387,-0.0354,-0.0354,0.0012207,-0.11597,-0.093994,-0.11475,-0.1709,-0.14038,-0.21484,-0.2832,-0.29297,-0.20508,-0.098877,-0.087891,-0.0024414,0.037842,0.023193,0.12573,0.12451,0.12939,0.20752,0.31006,0.27588,0.34424,0.36133,0.3186,0.2356,0.2771,0.22095,0.20264,0.24414,0.30029,0.24902,0.30029,0.29785,0.25513,0.30273,0.2478,0.20386,0.2063,0.2417,0.15869,0.15991,0.10864,0.01709,0.0073242,-0.12085,-0.19287,-0.21118,-0.16113,-0.076904,-0.11353,-0.068359,-0.075684,-0.11841,-0.084229,-0.14648,-0.19165,-0.16357,-0.083008,-0.11353,-0.040283,0.025635,0.026855,-0.010986,0.079346,0.05249,0.05249,0.087891,0.15381,0.091553,0.12695,0.10986,0.058594,0.095215,0.041504,0.0012207,0.014648,0.045166,-0.03418,0.021973,0.0012207,-0.045166,-0.10376,-0.054932,-0.11475,-0.11841,-0.041504,-0.062256,0.015869,0.075684,0.056152,-0.01709,-0.090332,-0.068359,-0.14893,-0.14282,-0.13428,-0.21606,-0.15625,-0.1355,-0.14526,-0.15381,-0.064697,-0.08667,-0.054932,0.014648,0.10864,0.10986,0.20508,0.22827,0.20264,0.16602,0.22095,0.18555,0.19897,0.22827,0.15991,0.22217,0.2478,0.21973,0.14404,0.18311,0.087891,0.024414,0.041504,-0.057373,-0.0354,-0.021973,-0.068359,-0.18677,-0.30273,-0.3064,-0.40527,-0.40161,-0.32227,-0.33081,-0.177,-0.074463,-0.019531,-0.0036621,-0.015869,0.083008,0.05127,0.10742,0.19897,0.14282,0.21729,0.21851,0.1355,0.040283,0.053711,-0.067139,-0.08667,-0.063477,-0.14404,-0.1062,-0.072021,-0.11597,-0.20264,-0.29419,-0.28076,-0.354,-0.37354,-0.33813,-0.4187,-0.35278,-0.30884,-0.32104,-0.3418,-0.25391,-0.24658,-0.19287,-0.063477,-0.057373,0.054932,0.12085,0.13916,0.095215,0.18188,0.16968,0.16113,0.21729,0.30518,0.25024,0.3125,0.33936,0.31372,0.2478,0.31616,0.27222,0.27222,0.34546,0.31982,0.40894,0.44922,0.45288,0.3772,0.39185,0.31128,0.2356,0.21729,0.25269,0.16235,0.18555,0.17944,0.12817,0.0354,0.080566,0.023193,0.020752,0.075684,0.018311,0.10132,0.14771,0.1355,0.091553,0.13672,0.061035,-0.0061035,-0.014648,0.023193,-0.063477,0.0048828,0.0024414,-0.028076,-0.093994,-0.042725,-0.098877,-0.1001,-0.068359,-0.013428,-0.070801,-0.045166,-0.061035,-0.10376,-0.062256,-0.12939,-0.15869,-0.13062,-0.054932,-0.078125,0.031738,0.074463,0.091553,0.072021,0.16846,0.13672,0.15381,0.21362,0.27954,0.23193,0.25757,0.21851,0.14771,0.177,0.068359,-0.0097656,-0.013428,0.026855,-0.072021,-0.01709,-0.0073242,-0.068359,-0.15625,-0.13306,-0.23193,-0.27954,-0.26367,-0.25513,-0.33325,-0.33813,-0.37598,-0.44922,-0.38086,-0.41748,-0.41016,-0.33325,-0.22339,-0.23682,-0.11963,-0.070801,-0.093994,-0.15015,-0.075684,-0.12085,-0.1355,-0.074463,-0.024414,-0.1001,-0.059814,-0.079346,-0.15137,-0.23315,-0.18921,-0.26611,-0.27588,-0.25635,-0.37354,-0.37598,-0.42725,-0.52002,-0.61523,-0.57129,-0.59814,-0.58472,-0.50171,-0.40283,-0.41626,-0.34546,-0.32471,-0.35767,-0.40161,-0.3479,-0.39917,-0.39185,-0.32471,-0.35767,-0.29175,-0.23193,-0.23193,-0.27832,-0.19897,-0.21118,-0.20264,-0.11963,-0.11108,-0.015869,0.075684,0.12939,0.11108,0.072021,0.16846,0.15259,0.17578,0.24536,0.19531,0.25269,0.26367,0.22827,0.15015,0.1709,0.087891,0.036621,0.058594,0.14404,0.13062,0.2417,0.31006,0.32349,0.29541,0.38086,0.35889,0.36377,0.41992,0.36987,0.41138,0.42725,0.40771,0.34546,0.37964,0.31128,0.25391,0.25269,0.29297,0.22461,0.25146,0.22461,0.17456,0.11108,0.16235,0.11597,0.14771,0.21851,0.20264,0.31372,0.39795,0.42603,0.42603,0.51147,0.48218,0.47485,0.50537,0.44556,0.48706,0.50903,0.48218,0.42114,0.33569,0.3833,0.32593,0.3186,0.36133,0.30273,0.3833,0.41382,0.41016,0.37354,0.32349,0.37842,0.35889,0.38696,0.4541,0.40771,0.47241,0.4834,0.448,0.3833,0.43579,0.37598,0.36621,0.42358,0.36743,0.41382,0.44922,0.4126,0.33447,0.36987,0.28442,0.21851,0.22949,0.26367,0.17334,0.21362,0.19287,0.10986,-0.012207,0.0036621,-0.10986,-0.14648,-0.1001,-0.17822,-0.10376,-0.041504,-0.028076,-0.040283,0.062256,0.045166,0.061035,0.14771,0.25757,0.22949,0.3064,0.32837,0.30273,0.22217,0.25269,0.17944,0.13062,0.14038,0.15625,0.05127,0.029297,-0.040283,-0.12695,-0.10986,-0.18555,-0.22095,-0.18311,-0.098877,-0.11475,-0.029297,0.048828,0.061035,0.015869,0.087891,0.062256,0.047607,0.1001,0.16357,0.097656,0.12695,0.1001,0.014648,0.030518,-0.06958,-0.13794,-0.15625,-0.12939,-0.19287,-0.13062,-0.097656,-0.093994,-0.13306,-0.058594,-0.087891,-0.079346,-0.0085449,-0.029297,0.06958,0.12695,0.13062,0.084229,0.13184,0.058594,0.0036621,0.014648,0.06958,0.03418,0.11597,0.16602,0.17944,0.14404,0.20752,0.16846,0.15625,0.18799,0.26123,0.21851,0.28076,0.2832,0.23071,0.16602,0.2063,0.14526,0.14648,0.17212,0.097656,0.13794,0.15137,0.10376,0.031738,0.061035,-0.013428,-0.03418,0.0012207,0.064697,0.023193,0.11597,0.15015,0.13672,0.10376,0.1709,0.13672,0.16968,0.22461,0.17334,0.25269,0.25879,0.23804,0.16357,0.2063,0.13916,0.10742,0.15259,0.22949,0.15869,0.20874,0.21484,0.16357,0.083008,0.12329,0.030518,0.0097656,0.037842,-0.041504,0.025635,0.054932,0.063477,0.01709,0.093994,0.050049,0.025635,0.079346,0.15259,0.098877,0.17334,0.17944,0.12939,0.06958,0.13306,0.081787,0.097656,0.15991,0.10376,0.17822,0.24292,0.23926,0.19165,0.26611,0.21851,0.19409,0.24414,0.20142,0.2771,0.34546,0.35522,0.30273,0.20874,0.22705,0.13672,0.076904,0.081787,-0.018311,0.020752,-0.0024414,-0.045166,-0.15137,-0.15869,-0.24292,-0.30273,-0.29175,-0.20996,-0.25146,-0.19897,-0.16357,-0.19165,-0.27588,-0.20386,-0.24048,-0.2356,-0.13672,-0.13794,-0.015869,0.085449,0.14282,0.1355,0.23193,0.19409,0.16968,0.18555,0.23804,0.15991,0.20142,0.19043,0.14648,0.048828,0.068359,0.0036621,-0.0024414,0.053711,0.12085,0.080566,0.13672,0.14893,0.12085,0.16968,0.10742,0.062256,0.062256,0.089111,0.0024414,0.013428,-0.019531,-0.063477,-0.17456,-0.17456,-0.27832,-0.33447,-0.30151,-0.35278,-0.25391,-0.17578,-0.12451,-0.098877,-0.093994,0.019531,0.0354,0.10132,0.18677,0.15991,0.2356,0.26733,0.24414,0.18311,0.23438,0.18555,0.17334,0.20874,0.26489,0.20508,0.22705,0.18921,0.097656,0.10132,-0.029297,-0.1355,-0.17334,-0.21118,-0.35889,-0.40161,-0.51758,-0.67749,-0.8606,-0.979,-1.1682,-1.2585,-1.3464,-1.3818,-1.4368,-1.4221,-1.416,-1.3672,-1.2659,-1.1719,-1.0596,-0.87524,-0.6897,-0.60181,-0.43213,-0.33936,-0.31128,-0.3125,-0.21362,-0.22705,-0.19287,-0.091553,0.056152,0.076904,0.22095,0.28442,0.29541,0.40405,0.37109,0.36133,0.42358,0.51025,0.50537,0.59326,0.64087,0.6189,0.55664,0.5957,0.53223,0.48218,0.49805,0.52734,0.43701,0.45166,0.43945,0.37231,0.42725,0.36743,0.32227,0.35156,0.41992,0.37476,0.42847,0.43701,0.39063,0.28564,0.31982,0.22827,0.15381,0.15259,0.19775,0.11841,0.1709,0.18799,0.13672,0.17944,0.11963,0.05249,0.067139,0.12451,0.070801,0.12817,0.15015,0.10498,0,0.012207,-0.084229,-0.15503,-0.17334,-0.27832,-0.2771,-0.2832,-0.3418,-0.40039,-0.46631,-0.41748,-0.44678,-0.41992,-0.36011,-0.39673,-0.32593,-0.27466,-0.2478,-0.26367,-0.19287,-0.19653,-0.19287,-0.12573,-0.13306,-0.029297,0.058594,0.097656,0.08667,0.018311,0.062256,-0.0097656,-0.025635,0.014648,-0.048828,0.061035,0.10498,0.12207,0.080566,0.14648,0.11108,0.085449,0.13062,0.21851,0.18188,0.26855,0.29053,0.25269,0.16602,0.177,0.091553,0.073242,0.13916,0.11108,0.21851,0.2832,0.28809,0.25024,0.29907,0.23193,0.18799,0.19775,0.23804,0.16968,0.21851,0.21362,0.177,0.1123,0.17578,0.13062,0.14404,0.2063,0.15015,0.20752,0.23804,0.19043,0.11108,0.14404,0.058594,-0.015869,0.012207,0.056152,0.0061035,0.078125,0.10742,0.096436,0.048828,0.10986,0.046387,0.0012207,0.0073242,0.037842,-0.039063,0.010986,0.019531,0.0036621,0.093994,0.054932,0.047607,0.10376,0.19775,0.15991,0.24048,0.29541,0.28931,0.23315,0.29175,0.23682,0.20264,0.23071,0.15625,0.18799,0.20508,0.17578,0.083008,0.11841,0.053711,-0.015869,0.030518,0.1123,0.072021,0.15625,0.17334,0.12451,0.026855,0.042725,-0.056152,-0.11841,-0.11841,-0.078125,-0.14648,-0.11963,-0.17212,-0.29907,-0.3479,-0.50903,-0.63721,-0.70435,-0.71899,-0.78247,-0.71533,-0.65063,-0.60669,-0.59204,-0.47119,-0.44556,-0.40039,-0.30273,-0.15747,-0.16235,-0.056152,0.0024414,-0.012207,-0.028076,0.063477,0.050049,0.097656,0.15991,0.12695,0.19165,0.22461,0.21729,0.16113,0.20508,0.13672,0.10742,0.12817,0.18677,0.10742,0.13306,0.10376,0.012207,0.019531,-0.1001,-0.1709,-0.19653,-0.16602,-0.23926,-0.19775,-0.17578,-0.17944,-0.19775,-0.1062,-0.084229,-0.048828,0.076904,0.19775,0.21973,0.34668,0.40894,0.40405,0.36499,0.43579,0.40039,0.43213,0.47974,0.41016,0.46387,0.46875,0.40771,0.31006,0.29419,0.16602,0.079346,0.056152,0.081787,-0.0073242,0.03418,0.012207,-0.048828,-0.13428,-0.12329,-0.22095,-0.26123,-0.24414,-0.30396,-0.25024,-0.19897,-0.18799,-0.20142,-0.095215,-0.10254,-0.097656,-0.021973,0.048828,-0.0085449,0.059814,0.039063,-0.026855,-0.1123,-0.093994,-0.18188,-0.22217,-0.20874,-0.28687,-0.22705,-0.20142,-0.24292,-0.33203,-0.45288,-0.47119,-0.5835,-0.61523,-0.57373,-0.61646,-0.55664,-0.53223,-0.54321,-0.57983,-0.4834,-0.48828,-0.43945,-0.33569,-0.33813,-0.21973,-0.12085,-0.029297,-0.0061035,-0.0097656,0.098877,0.072021,0.10742,0.16846,0.10376,0.14282,0.10986,0.0073242,-0.15869,-0.18921,-0.33203,-0.40039,-0.35034,-0.35156,-0.23315,-0.083008,-0.026855,-0.046387,-0.081787,0.0024414,-0.056152,-0.05127,-0.025635,-0.087891,-0.024414,0.015869,-0.012207,-0.065918,0.0097656,-0.021973,0.015869,0.084229,0.079346,0.2063,0.29541,0.33691,0.32471,0.27222,0.31982,0.25269,0.25757,0.29053,0.20874,0.22461,0.21606,0.16235,0.073242,0.091553,-0.019531,-0.10132,-0.13306,-0.25635,-0.26123,-0.2771,-0.32837,-0.38696,-0.30518,-0.29907,-0.25024,-0.14038,-0.0073242,0.012207,0.13062,0.19409,0.20508,0.15381,0.18555,0.095215,0.0354,-0.0061035,-0.12695,-0.12695,-0.13916,-0.2002,-0.3064,-0.40039,-0.37109,-0.39307,-0.32471,-0.22461,-0.23071,-0.11841,-0.019531,0.020752,0.015869,0.11597,0.1123,0.11597,0.177,0.26733,0.21484,0.26489,0.24414,0.177,0.06958,0.048828,-0.087891,-0.14648,-0.1709,-0.24536,-0.15625,-0.090332,-0.10376,-0.11597,-0.0073242,-0.013428,-0.0085449,0.10742,0.23315,0.26733,0.41992,0.50415,0.5127,0.49316,0.55786,0.53101,0.55664,0.59937,0.54932,0.59692,0.58472,0.51758,0.40039,0.35522,0.21484,0.10498,0.058594,0.059814,-0.058594,-0.0097656,-0.0012207,-0.045166,-0.1001,-0.03418,-0.072021,-0.03418,0.05249,0.029297,0.12451,0.21484,0.2478,0.25391,0.38208,0.37964,0.37598,0.43335,0.51758,0.46875,0.53833,0.53955,0.46997,0.36255,0.36499,0.25146,0.18066,0.15625,0.041504,0.058594,0.081787,0.083008,0.058594,0.14526,0.13794,0.15381,0.22949,0.3418,0.35522,0.47119,0.52979,0.54199,0.49805,0.5542,0.50415,0.48828,0.51758,0.44189,0.47363,0.45532,0.41748,0.31494,0.30273,0.19775,0.1001,0.063477,0.081787,-0.026855,-0.014648,-0.041504,-0.11841,-0.24414,-0.26001,-0.36011,-0.41016,-0.38086,-0.44922,-0.42603,-0.42358,-0.43823,-0.47852,-0.43335,-0.47241,-0.47852,-0.41016,-0.29907,-0.29785,-0.19165,-0.15259,-0.14404,-0.18066,-0.11719,-0.16235,-0.16479,-0.1062,-0.11475,-0.0048828,0.10376,0.1355,0.12085,0.15991,0.075684,0.0024414,-0.020752,-0.0012207,-0.084229,-0.05249,-0.054932,-0.068359,-0.095215,-0.029297,-0.030518,0.0354,0.12451,0.24414,0.2771,0.36499,0.38818,0.37842,0.32715,0.37109,0.34302,0.37598,0.43091,0.40527,0.46509,0.50171,0.49927,0.44067,0.45288,0.37842,0.33813,0.37598,0.3418,0.42603,0.48218,0.49072,0.46631,0.43335,0.49805,0.46997,0.49194,0.53955,0.49438,0.53833,0.53345,0.47119,0.38452,0.39429,0.2832,0.21973,0.21606,0.24658,0.16846,0.2124,0.20264,0.14648,0.20752,0.16235,0.13062,0.19409,0.30273,0.29541,0.39429,0.42847,0.39429,0.33203,0.36743,0.29907,0.26123,0.27832,0.34302,0.26611,0.29419,0.26367,0.19409,0.1123,0.14648,0.091553,0.13062,0.2124,0.18066,0.26367,0.32227,0.30029,0.2478,0.29297,0.23193,0.20508,0.23193,0.3064,0.2417,0.29419,0.31372,0.28076,0.22827,0.30273,0.26611,0.27222,0.31372,0.26733,0.32471,0.33936,0.33936,0.29053,0.34546,0.30518,0.28809,0.31616,0.40039,0.35522,0.41016,0.42236,0.37231,0.2832,0.30396,0.22095,0.20874,0.24414,0.17822,0.22705,0.2478,0.22461,0.16357,0.21484,0.15991,0.12695,0.18188,0.26978,0.21851,0.2832,0.25513,0.16968,0.041504,0.021973,-0.084229,-0.10742,-0.078125,-0.12085,-0.018311,0.062256,0.079346,0.079346,0.1709,0.15869,0.17822,0.22827,0.29175,0.24658,0.31006,0.29907,0.24414,0.13672,0.14038,0.043945,0.015869,0.046387,0.1001,0.05249,0.092773,0.089111,0.046387,0.10132,0.042725,0.0061035,0.028076,0.091553,0.037842,0.097656,0.12329,0.087891,0.018311,0.072021,0.012207,0.0036621,0.064697,0.12329,0.08667,0.13062,0.11108,0.063477,0.10498,0.026855,-0.062256,-0.045166,-0.021973,-0.10742,-0.048828,-0.020752,-0.045166,-0.090332,0.0036621,-0.046387,-0.030518,0.036621,0.098877,0.046387,0.12573,0.12939,0.095215,0.16602,0.10986,0.064697,0.087891,0.12085,0.043945,0.1001,0.090332,0.0354,-0.064697,-0.019531,-0.10254,-0.13672,-0.091553,-0.11353,-0.013428,0.087891,0.11353,0.10498,0.19897,0.17578,0.14648,0.17578,0.25024,0.19409,0.24048,0.26367,0.22827,0.12695,0.16602,0.079346,0.01709,0.031738,0.087891,0.031738,0.091553,0.11353,0.073242,0.13062,0.087891,0.053711,0.078125,0.14282,0.096436,0.177,0.20386,0.2002,0.1355,0.17456,0.10132,0.072021,0.087891,0.13672,0.057373,0.097656,0.095215,0.041504,0.079346,-0.0073242,-0.076904,-0.084229,-0.058594,-0.12329,-0.068359,-0.040283,-0.043945,-0.083008,-0.028076,-0.056152,-0.061035,-0.012207,0.085449,0.039063,0.1123,0.11475,0.050049,0.070801,-0.046387,-0.12085,-0.11475,-0.081787,-0.14893,-0.10376,-0.095215,-0.13794,-0.19775,-0.1355,-0.17578,-0.20142,-0.17334,-0.12085,-0.16357,-0.098877,-0.075684,-0.1123,-0.18799,-0.16602,-0.26489,-0.31372,-0.32471,-0.44556,-0.43945,-0.47485,-0.52124,-0.5835,-0.51514,-0.51147,-0.46509,-0.354,-0.22949,-0.22339,-0.097656,-0.041504,-0.05249,-0.078125,-0.0024414,-0.036621,-0.0012207,0.084229,0.15015,0.13062,0.17578,0.15259,0.087891,0.12207,0.021973,-0.074463,-0.10742,-0.12817,-0.27466,-0.29663,-0.35034,-0.42725,-0.50293,-0.448,-0.45776,-0.39185,-0.271,-0.24048,-0.097656,0.036621,0.068359,0.058594,0.12695,0.070801,0.019531,0.041504,0.085449,0.015869,0.054932,0.064697,0.020752,0.048828,-0.021973,-0.064697,-0.072021,0.024414,0.12329,0.11353,0.19409,0.22461,0.18921,0.25269,0.20508,0.15625,0.15259,0.19897,0.1355,0.17944,0.19531,0.15625,0.083008,0.15259,0.10742,0.092773,0.14893,0.11841,0.20874,0.27832,0.28076,0.23682,0.28564,0.22583,0.17334,0.17944,0.22949,0.16846,0.20508,0.2002,0.15259,0.056152,0.075684,-0.0073242,-0.043945,-0.031738,-0.093994,-0.054932,-0.029297,-0.057373,-0.11475,-0.20508,-0.14404,-0.17334,-0.15259,-0.067139,-0.076904,0.010986,0.05249,0.072021,0.030518,0.097656,0.05127,0.037842,0.072021,0.12939,0.081787,0.12817,0.12085,0.080566,0.11597,0.040283,-0.019531,-0.028076,-0.018311,-0.10498,-0.076904,-0.058594,-0.073242,-0.12207,-0.075684,-0.11353,-0.11597,-0.061035,-0.084229,0.010986,0.081787,0.11963,0.1123,0.080566,0.13916,0.095215,0.091553,0.084229,-0.036621,-0.048828,-0.15015,-0.32471,-0.55908,-0.67993,-0.86304,-0.96313,-0.97046,-0.93262,-0.93628,-0.83862,-0.77515,-0.73608,-0.69092,-0.54688,-0.48706,-0.35522,-0.21851,-0.19043,-0.063477,-0.0036621,0.0073242,-0.031738,0.029297,-0.020752,-0.013428,0.072021,0.17334,0.15747,0.25635,0.26123,0.2124,0.12085,0.11108,-0.015869,-0.092773,-0.16968,-0.33813,-0.36133,-0.39673,-0.44312,-0.54688,-0.55542,-0.67993,-0.80322,-0.86792,-0.93384,-1.0754,-1.167,-1.3184,-1.5283,-1.6736,-1.6772,-1.8616,-1.9153,-1.9641,-2.0923,-2.0569,-1.3623,-1.0522,-0.81787,-0.61401,-0.49805,-0.41626,-0.3064,-0.18555,-0.15259,-0.045166,0,-0.023193,-0.081787,-0.061035,-0.13306,-0.15991,-0.11475,-0.13672,-0.065918,-0.0036621,0.019531,-0.026855,0.036621,0,-0.020752,0.050049,0.15381,0.16602,0.26611,0.31372,0.32227,0.28564,0.35156,0.34546,0.36987,0.4126,0.37842,0.44067,0.50293,0.54688,0.55664,0.61768,0.5835,0.55054,0.55664,0.57617,0.49805,0.4834,0.42358,0.33569,0.22705,0.19897,0.10742,0.046387,0.059814,0.025635,0.091553,0.13672,0.14404,0.11597,0.18433,0.16235,0.15747,0.19165,0.26489,0.25391,0.32227,0.36865,0.38696,0.37109,0.43335,0.40527,0.40039,0.43579,0.38208,0.41504,0.39795,0.31982,0.18921,0.11108,-0.029297,-0.13794,-0.18188,-0.15625,-0.17944,-0.067139,0.018311,0.080566,0.11475,0.22339,0.2478,0.29541,0.36743,0.46265,0.46997,0.52856,0.53833,0.51147,0.46143,0.49316,0.43945,0.43457,0.44312,0.37354,0.39307,0.39307,0.36133,0.31494,0.35522,0.33203,0.31372,0.33203,0.35889,0.29663,0.3064,0.28687,0.23438,0.18799,0.2478,0.2478,0.30518,0.39307,0.39795,0.48828,0.5249,0.49561,0.45166,0.47363,0.40283,0.34668,0.32227,0.21729,0.19165,0.13428,0.0048828,-0.14404,-0.2771,-0.25635,-0.25146,-0.16235,-0.026855,0.013428,0.14648,0.23438,0.27588,0.26367,0.35767,0.36377,0.37598,0.43213,0.42603,0.46021,0.50659,0.48462,0.39673,0.28931,0.27466,0.16846,0.14038,0.17456,0.12085,0.17822,0.21729,0.22583,0.19775,0.25391,0.23193,0.22827,0.28931,0.38086,0.3772,0.45044,0.46631,0.42236,0.34424,0.34546,0.26733,0.25269,0.25757,0.18799,0.21362,0.22705,0.18921,0.12695,0.14282,0.096436,0.06958,0.080566,0.13672,0.083008,0.1062,0.092773,0.021973,-0.087891,-0.087891,-0.15869,-0.17212,-0.1355,-0.16113,-0.068359,-0.013428,0.036621,0.048828,0.16479,0.18921,0.22461,0.29785,0.40405,0.42114,0.49805,0.5188,0.50659,0.45654,0.49194,0.46143,0.46021,0.50537,0.48218,0.5481,0.58472,0.57617,0.53345,0.55664,0.50659,0.47485,0.49805,0.53345,0.50171,0.53711,0.52368,0.45898,0.354,0.34424,0.24414,0.20752,0.21118,0.14648,0.18555,0.21729,0.17822,0.12817,0.12939,0.053711,-0.0073242,0.0097656,0.0085449,-0.067139,-0.045166,-0.080566,-0.14648,-0.22705,-0.23682,-0.31982,-0.36133,-0.35767,-0.33447,-0.3479,-0.30273,-0.29175,-0.29053,-0.21362,-0.20264,-0.17822,-0.10742,-0.018311,-0.025635,0.059814,0.10864,0.1123,0.078125,0.12451,0.067139,0.0354,0.075684,0.10376,0.053711,0.065918,0.0354,-0.018311,0.023193,-0.028076,-0.028076,0.029297,0.12817,0.14771,0.25879,0.32593,0.3479,0.32593,0.40283,0.37842,0.38208,0.44434,0.42847,0.48096,0.52368,0.50537,0.44189,0.44067,0.37476,0.3125,0.3064,0.33447,0.25757,0.271,0.24658,0.16968,0.072021,0.080566,0.015869,-0.0048828,0.042725,0.10742,0.10132,0.17822,0.20386,0.18921,0.2417,0.2124,0.20508,0.23071,0.28564,0.25757,0.29541,0.30518,0.26367,0.18311,0.18799,0.13428,0.074463,0.085449,0.048828,0.097656,0.15625,0.17334,0.14648,0.21362,0.18188,0.15625,0.17578,0.23438,0.21729,0.29053,0.31616,0.30518,0.26733,0.31006,0.2832,0.30029,0.3479,0.42725,0.41016,0.45654,0.45166,0.40283,0.33203,0.34546,0.2832,0.27222,0.28564,0.21851,0.26733,0.271,0.24658,0.18921,0.20508,0.14038,0.11475,0.11475,0.14648,0.084229,0.10132,0.072021,0.0073242,-0.080566,-0.079346,-0.15015,-0.15015,-0.11841,-0.15503,-0.1001,-0.074463,-0.087891,-0.13306,-0.11963,-0.16602,-0.19165,-0.14282,-0.17822,-0.11963,-0.059814,-0.059814,-0.10498,-0.15381,-0.12573,-0.17334,-0.15991,-0.097656,-0.1062,-0.0073242,0.059814,0.070801,0.056152,0.12939,0.10376,0.097656,0.12817,0.177,0.12085,0.14648,0.11353,0.0085449,-0.026855,-0.13428,-0.22461,-0.26367,-0.25635,-0.33813,-0.30273,-0.26855,-0.25391,-0.26367,-0.18555,-0.21118,-0.22705,-0.18433,-0.11353,-0.14404,-0.08667,-0.074463,-0.10132,-0.16479,-0.11963,-0.17944,-0.18799,-0.14893,-0.19775,-0.15381,-0.1355,-0.17212,-0.23438,-0.21851,-0.28198,-0.32837,-0.31494,-0.26978,-0.30518,-0.26611,-0.27588,-0.33569,-0.42969,-0.44434,-0.50415,-0.54077,-0.53101,-0.52612,-0.56396,-0.59326,-0.65186,-0.73853,-0.76782,-0.77637,-0.76172,-0.69946,-0.57007,-0.48462,-0.3418,-0.20142,-0.11719,-0.076904,0.05249,0.074463,0.13062,0.23193,0.23315,0.3064,0.32959,0.28442,0.20142,0.17334,0.061035,-0.023193,-0.047607,-0.048828,-0.10376,-0.068359,-0.065918,-0.075684,-0.12695,-0.11108,-0.15869,-0.16602,-0.14404,-0.16602,-0.08667,-0.025635,-0.0048828,-0.023193,0.019531,-0.012207,0.010986,0.078125,0.18188,0.18311,0.271,0.32837,0.34424,0.32227,0.38086,0.36133,0.36987,0.40649,0.45288,0.42236,0.44922,0.40161,0.32227,0.21362,0.19165,0.11597,0.093994,0.096436,0.050049,0.11108,0.15381,0.16846,0.15991,0.21484,0.18311,0.17944,0.23071,0.29175,0.2771,0.31982,0.30273,0.23804,0.15137,0.15015,0.05249,-0.0097656,-0.058594,-0.19653,-0.22095,-0.28687,-0.35645,-0.41382,-0.39673,-0.41748,-0.42236,-0.37354,-0.30518,-0.28198,-0.16602,-0.074463,-0.010986,0.039063,0.15869,0.18311,0.27466,0.38818,0.39429,0.49194,0.53955,0.51147,0.44556,0.45654,0.37354,0.30518,0.29419,0.3064,0.22461,0.24048,0.19531,0.11108,0.012207,-0.015869,-0.095215,-0.10742,-0.087891,-0.13794,-0.084229,-0.031738,-0.019531,-0.028076,0.050049,0.030518,0.040283,0.11108,0.19409,0.15991,0.19775,0.20264,0.14648,0.054932,0.072021,0.0036621,-0.048828,-0.05249,-0.14282,-0.16235,-0.19287,-0.25024,-0.3479,-0.36011,-0.4248,-0.46387,-0.43091,-0.35278,-0.30273,-0.177,-0.085449,-0.045166,-0.05249,0.023193,0.0048828,0.03418,0.093994,0.070801,0.12817,0.16846,0.15747,0.1123,0.14771,0.10864,0.075684,0.087891,0.12695,0.073242,0.098877,0.090332,0.063477,0,0.047607,0.031738,0.037842,0.092773,0.075684,0.13672,0.16602,0.12695,0.036621,0.026855,-0.053711,-0.10986,-0.10376,-0.087891,-0.13306,-0.054932,-0.0073242,0.0024414,-0.031738,0.036621,-0.0012207,0.014648,0.06958,0.054932,0.13794,0.16968,0.16846,0.1123,0.054932,0.085449,0.046387,0.054932,0.084229,0.021973,0.048828,0.032959,-0.0036621,-0.054932,-0.018311,-0.05249,-0.037842,0.030518,0.10986,0.11841,0.19897,0.24658,0.24414,0.32104,0.28442,0.24902,0.26367,0.28687,0.22949,0.26489,0.27588,0.24658,0.18799,0.22583,0.17578,0.15381,0.20874,0.2832,0.25757,0.32227,0.31616,0.27588,0.22827,0.27344,0.23926,0.25391,0.30029,0.26855,0.33203,0.33691,0.31006,0.26123,0.2832,0.22827,0.19531,0.21606,0.26367,0.23804,0.29297,0.30029,0.25513,0.19287,0.21851,0.16602,0.17578,0.21484,0.16846,0.19287,0.19287,0.13184,0.013428,-0.013428,-0.097656,-0.15625,-0.13062,-0.14648,-0.067139,0.023193,0.081787,0.10132,0.18921,0.177,0.15869,0.17456,0.23682,0.19043,0.23193,0.21973,0.16846,0.050049,0.0073242,-0.087891,-0.15869,-0.17822,-0.24048,-0.2063,-0.16846,-0.15625,-0.18799,-0.22217,-0.15259,-0.14526,-0.11108,-0.047607,-0.070801,-0.025635,-0.03418,-0.090332,-0.19653,-0.271,-0.40283,-0.50415,-0.54565,-0.53345,-0.52368,-0.45776,-0.40649,-0.39063,-0.40283,-0.33813,-0.32837,-0.271,-0.18555,-0.17456,-0.078125,-0.013428,0.0097656,0,0.064697,0.048828,0.068359,0.1416,0.25757,0.26733,0.34302,0.37964,0.36133,0.3064,0.33569,0.29297,0.29175,0.31738,0.27466,0.3418,0.33936,0.32471,0.27954,0.3186,0.25635,0.23438,0.24902,0.29541,0.25757,0.31006,0.30884,0.26367,0.19775,0.22827,0.17456,0.17456,0.2063,0.1416,0.18677,0.18677,0.14282,0.048828,0.026855,-0.08667,-0.16479,-0.17822,-0.14282,-0.1709,-0.078125,-0.026855,0.0061035,-0.0024414,0.098877,0.090332,0.13672,0.22949,0.23682,0.32227,0.38818,0.37109,0.31616,0.33569,0.24536,0.16846,0.15259,0.15991,0.091553,0.12085,0.12329,0.06958,0.012207,0.042725,-0.013428,-0.020752,0.036621,0.089111,0.075684,0.11108,0.10498,0.037842,0.05127,-0.0097656,-0.045166,-0.012207,0.041504,0.0024414,0.056152,0.093994,0.072021,0.0097656,0.06958,0.056152,0.068359,0.16602,0.16479,0.23926,0.29419,0.2832,0.21851,0.22949,0.15381,0.076904,0.057373,0.090332,0.028076,0.053711,0.05127,0.010986,-0.078125,-0.041504,-0.10132,-0.13916,-0.12085,-0.15991,-0.12451,-0.092773,-0.1062,-0.17456,-0.18188,-0.25024,-0.29419,-0.3064,-0.29297,-0.32227,-0.27222,-0.24658,-0.23071,-0.25269,-0.17334,-0.16602,-0.13306,-0.045166,-0.025635,0.083008,0.14038,0.16113,0.12085,0.14893,0.075684,0.019531,0.010986,0.032959,-0.0097656,0.025635,0.0061035,-0.043945,-0.11963,-0.14648,-0.22217,-0.26001,-0.2417,-0.24658,-0.15259,-0.076904,-0.036621,-0.025635,-0.047607,0.0097656,-0.0061035,0.039063,0.090332,0.078125,0.15625,0.17212,0.16968,0.14038,0.19287,0.15015,0.13794,0.177,0.24902,0.2124,0.24902,0.23804,0.17578,0.10132,0.13062,0.078125,0.089111,0.10742,0.056152,0.12329,0.13428,0.11597,0.078125,0.11963,0.076904,0.072021,0.10132,0.14893,0.10498,0.16357,0.14038,0.079346,-0.0061035,-0.021973,-0.11597,-0.11353,-0.06958,-0.084229,0.013428,0.072021,0.079346,0.032959,0.056152,-0.039063,-0.13062,-0.18188,-0.20386,-0.27954,-0.24292,-0.22461,-0.24048,-0.25391,-0.17212,-0.17212,-0.11963,-0.053711,-0.087891,-0.0061035,0.019531,0.015869,-0.01709,-0.046387,0.015869,0.0036621,0.05249,0.12939,0.097656,0.15503,0.17212,0.13062,0.040283,0.037842,-0.064697,-0.10986,-0.095215,-0.16846,-0.15869,-0.14893,-0.15137,-0.21118,-0.18677,-0.22095,-0.2417,-0.18311,-0.079346,-0.085449,-0.0036621,0.032959,0.0085449,-0.072021,-0.048828,-0.13062,-0.15869,-0.13916,-0.21118,-0.17578,-0.15137,-0.16602,-0.22827,-0.21484,-0.25635,-0.27954,-0.25757,-0.2124,-0.25146,-0.22827,-0.26123,-0.35889,-0.49805,-0.59692,-0.80811,-1.0229,-1.2769,-1.4807,-1.5698,-1.6309,-1.6101,-1.5369,-1.4355,-1.2207,-1.0291,-0.85083,-0.67383,-0.57007,-0.44189,-0.34912,-0.29907,-0.30273,-0.23804,-0.24048,-0.20874,-0.11597,-0.10498,-0.0061035,0.05249,0.090332,0.095215,0.1709,0.16602,0.14648,0.15503,0.18066,0.11108,0.13916,0.11719,0.057373,-0.015869,0.020752,-0.020752,-0.0061035,0.041504,0.12207,0.14038,0.23682,0.29541,0.32959,0.42603,0.43579,0.46265,0.54077,0.63599,0.67383,0.78125,0.82275,0.79346,0.72754,0.69336,0.58838,0.51514,0.47852,0.47607,0.39917,0.40283,0.37109,0.31494,0.2417,0.26733,0.22827,0.22827,0.25635,0.2124,0.25757,0.29419,0.28076,0.23682,0.28442,0.24414,0.2417,0.29297,0.34424,0.30273,0.33569,0.3186,0.26001,0.28564,0.20874,0.14771,0.13306,0.15991,0.089111,0.14893,0.14771,0.11108,0.062256,0.092773,0.047607,0.023193,0.050049,0.098877,0.056152,0.090332,0.081787,0.037842,0.073242,-0.0097656,-0.061035,-0.068359,-0.029297,-0.067139,-0.0048828,0.037842,0.040283,0.015869,0.090332,0.070801,0.050049,0.092773,0.15991,0.12329,0.17578,0.18188,0.15625,0.11353,0.17212,0.13306,0.15625,0.22095,0.20752,0.28442,0.3479,0.34424,0.3125,0.3479,0.30518,0.28076,0.29663,0.33813,0.3064,0.354,0.36255,0.32959,0.26001,0.28076,0.23071,0.20996,0.21729,0.16235,0.19165,0.18311,0.16602,0.10498,0.12451,0.061035,0.025635,0.032959,0.057373,-0.024414,-0.029297,-0.093994,-0.21729,-0.39185,-0.50903,-0.67993,-0.80322,-0.84229,-0.84595,-0.78613,-0.72266,-0.67139,-0.64087,-0.57983,-0.53711,-0.46265,-0.3479,-0.21729,-0.16479,-0.03418,0.032959,0.045166,0.012207,0.059814,0.021973,0.013428,0.042725,0.059814,0.012207,0.042725,0.0061035,-0.075684,-0.15381,-0.14282,-0.16724,-0.12329,-0.047607,-0.030518,0.085449,0.15625,0.19653,0.19531,0.27222,0.26367,0.31128,0.40649,0.41016,0.4834,0.52368,0.51758,0.47119,0.41382,0.42236,0.37231,0.37476,0.39429,0.32227,0.34302,0.31982,0.24536,0.15991,0.17212,0.087891,0.043945,0.072021,0.1001,0.056152,0.072021,0.037842,-0.026855,-0.093994,-0.058594,-0.1062,-0.10986,-0.075684,-0.1123,-0.057373,-0.036621,-0.05249,-0.12939,-0.098877,-0.13062,-0.13794,-0.064697,-0.072021,0.0012207,0.092773,0.13916,0.13062,0.19409,0.16968,0.14648,0.18188,0.27466,0.26245,0.32471,0.36133,0.33325,0.25757,0.2832,0.22095,0.177,0.19653,0.23438,0.16602,0.17944,0.15869,0.1001,0.11841,0.059814,0.021973,0.026855,0.075684,0.043945,0.091553,0.10864,0.091553,0.023193,0.05249,0.012207,-0.0024414,0.048828,0.10742,0.078125,0.12085,0.11963,0.070801,0.081787,0.0024414,-0.054932,-0.05127,-0.015869,-0.054932,0.0085449,0.0024414,-0.015869,-0.08667,-0.087891,-0.16357,-0.21729,-0.24414,-0.20752,-0.24048,-0.18677,-0.18555,-0.23193,-0.29419,-0.27344,-0.31616,-0.30151,-0.25146,-0.25513,-0.16602,-0.12451,-0.11841,-0.15869,-0.1355,-0.18188,-0.21118,-0.177,-0.10132,-0.093994,-0.0036621,0.020752,-0.0012207,-0.0354,-0.0097656,-0.046387,0.012207,0.1001,0.12695,0.23071,0.28809,0.28076,0.21729,0.21851,0.13428,0.070801,0.062256,0.087891,0.021973,0.032959,0.0048828,-0.057373,-0.12329,-0.10254,-0.13672,-0.10376,-0.020752,-0.024414,0.08667,0.13672,0.13672,0.10498,0.14648,0.078125,0.018311,0.0097656,0.045166,-0.015869,0.042725,0.0354,-0.01709,-0.070801,-0.05127,-0.11841,-0.11108,-0.045166,-0.057373,0.040283,0.10376,0.13062,0.14404,0.22827,0.21606,0.22461,0.28076,0.34424,0.32227,0.3894,0.39795,0.37109,0.30273,0.33203,0.271,0.26978,0.33203,0.3064,0.38818,0.47119,0.50171,0.48218,0.45166,0.48462,0.43701,0.43945,0.46509,0.3833,0.38696,0.36499,0.29297,0.17822,0.16357,0.058594,-0.0012207,0.01709,0.062256,0.040283,0.081787,0.080566,0.03418,0.068359,0.0061035,-0.036621,-0.01709,0.056152,0.05249,0.14648,0.22705,0.23438,0.17578,0.21484,0.15747,0.12817,0.15869,0.10376,0.14648,0.17578,0.16113,0.085449,0.054932,-0.045166,-0.10986,-0.11841,-0.058594,-0.065918,0.018311,0.081787,0.12329,0.13184,0.24414,0.26855,0.32593,0.41138,0.44067,0.53833,0.61646,0.63843,0.61523,0.54565,0.54077,0.49194,0.47852,0.4895,0.43213,0.44067,0.4187,0.36865,0.27466,0.26611,0.19287,0.15747,0.15381,0.18555,0.12329,0.1416,0.12085,0.090332,0.023193,0.054932,0.012207,0,0.031738,-0.01709,0.026855,0.01709,-0.029297,-0.095215,-0.072021,-0.10742,-0.10376,-0.01709,0.0012207,0.096436,0.17822,0.18311,0.13184,0.14771,0.054932,-0.029297,-0.042725,-0.032959,-0.092773,-0.043945,-0.046387,-0.072021,-0.11475,-0.090332,-0.14282,-0.14404,-0.10376,-0.03418,-0.045166,0.042725,0.06958,0.062256,0.042725,0.11108,0.081787,0.11475,0.16602,0.12695,0.17578,0.1709,0.10376,0,-0.0024414,-0.087891,-0.15381,-0.14404,-0.11963,-0.17212,-0.13306,-0.13794,-0.17578,-0.20752,-0.13916,-0.15991,-0.12695,-0.059814,-0.074463,0.013428,0.076904,0.078125,0.036621,0.072021,0.019531,-0.015869,0.010986,0.048828,-0.026855,0.0048828,-0.018311,-0.074463,-0.15625,-0.14404,-0.21973,-0.2417,-0.21606,-0.26978,-0.21118,-0.17822,-0.17334,-0.19653,-0.14526,-0.16235,-0.16846,-0.11597,-0.031738,-0.046387,0.0097656,0.029297,0.0012207,-0.062256,-0.028076,-0.097656,-0.15625,-0.14038,-0.18188,-0.11597,-0.065918,-0.070801,-0.12085,-0.090332,-0.12695,-0.15991,-0.14038,-0.085449,-0.10132,-0.0097656,0.046387,0.053711,0.032959,0.11108,0.1062,0.12939,0.19409,0.16479,0.23193,0.26367,0.23193,0.15747,0.15869,0.081787,0.032959,0.041504,0.091553,0.075684,0.14526,0.16724,0.17456,0.14648,0.19409,0.18066,0.18799,0.23438,0.19775,0.26001,0.28687,0.26489,0.19775,0.18677,0.090332,0.013428,0.0012207,0.031738,0.0024414,0.062256,0.1001,0.087891,0.048828,0.085449,0.048828,0.054932,0.10742,0.18311,0.16357,0.19287,0.16846,0.078125,0.062256,-0.065918,-0.18555,-0.28687,-0.36865,-0.48828,-0.51636,-0.56274,-0.61279,-0.66162,-0.65308,-0.64331,-0.57373,-0.43701,-0.30151,-0.24048,-0.10986,-0.042725,-0.026855,-0.036621,0.036621,0.019531,0.057373,0.1123,0.05249,0.057373,0.0024414,-0.12817,-0.30518,-0.43945,-0.62256,-0.74341,-0.77026,-0.72632,-0.68237,-0.60425,-0.54443,-0.51147,-0.51025,-0.44312,-0.43091,-0.36377,-0.27466,-0.25513,-0.15503,-0.098877,-0.11108,-0.15991,-0.12695,-0.17822,-0.22095,-0.22949,-0.31372,-0.29419,-0.26245,-0.2478,-0.27588,-0.22461,-0.22095,-0.19897,-0.14893,-0.072021,-0.090332,-0.0036621,0.040283,0.05249,0.018311,0.072021,0.029297,0.0036621,0.025635,0.075684,0.0073242,0.021973,-0.0012207,-0.084229,-0.17578,-0.18433,-0.25757,-0.25146,-0.18066,-0.17334,-0.081787,-0.019531,-0.0048828,-0.018311,0.054932,0.043945,0.068359,0.13184,0.22095,0.18311,0.21362,0.18921,0.13428,0.17578,0.15991,0.18799,0.26978,0.37842,0.41748,0.52368,0.58838,0.59204,0.57007,0.58105,0.52124,0.49805,0.50049,0.54688,0.52124,0.5603,0.57129,0.55542,0.49561,0.51392,0.45898,0.43091,0.45166,0.40161,0.44434,0.43945,0.41016,0.35278,0.37109,0.29541,0.26367,0.27832,0.31982,0.30029,0.35767,0.36621,0.33569,0.28931,0.30518,0.2356,0.23071,0.26367,0.2124,0.2771,0.3125,0.30029,0.25635,0.29541,0.22705,0.177,0.18188,0.21118,0.15259,0.18311,0.16602,0.092773,-0.029297,-0.025635,-0.10742,-0.13428,-0.10376,-0.15747,-0.12573,-0.097656,-0.14648,-0.23804,-0.27344,-0.41626,-0.5603,-0.66895,-0.75562,-0.84229,-0.87769,-0.92163,-0.94727,-0.94238,-0.89966,-0.82764,-0.75195,-0.63599,-0.53101,-0.48096,-0.40649,-0.37354,-0.38696,-0.34546,-0.35278,-0.36865,-0.36011,-0.34058,-0.39673,-0.4126,-0.45654,-0.52368,-0.60181,-0.58716,-0.59326,-0.57617,-0.52734,-0.48584,-0.51147,-0.51392,-0.54077,-0.55298,-0.53711,-0.52002,-0.50903,-0.45044,-0.32227,-0.26367,-0.12695,-0.028076,0.048828,0.041504,0.13306,0.13062,0.14893,0.21851,0.22949,0.29907,0.35889,0.36865,0.3418,0.30273,0.35278,0.33691,0.37598,0.43945,0.42725,0.49561,0.51392,0.48706,0.40405,0.39063,0.2832,0.18677,0.12817,0.11841,0.037842,0.046387,0.010986,-0.048828,-0.12085,-0.080566,-0.1001,-0.072021,0.0036621,0.013428,0.11353,0.17944,0.19897,0.16357,0.21606,0.19897,0.21851,0.28076,0.35522,0.32715,0.35889,0.35156,0.30151,0.23682,0.24536,0.18921,0.20264,0.24292,0.22705,0.30518,0.34668,0.36743,0.3479,0.37476,0.33203,0.32104,0.37476,0.46265,0.46875,0.5481,0.59448,0.57007,0.5249,0.52124,0.44678,0.40771,0.38452,0.28809,0.30884,0.31006,0.26367,0.18799,0.17822,0.078125,-0.0048828,-0.029297,-0.046387,-0.14648,-0.13794,-0.16357,-0.20508,-0.24536,-0.20752,-0.23438,-0.19653,-0.13672,-0.14648,-0.075684,-0.0354,-0.029297,-0.032959,-0.05127,0.031738,0.040283,0.10986,0.21851,0.23071,0.32471,0.37109,0.35034,0.30273,0.33325,0.25146,0.19653,0.19653,0.11353,0.13916,0.14282,0.091553,-0.01709,-0.1001,-0.10376,-0.16602,-0.15869,-0.13428,-0.18799,-0.14404,-0.13184,-0.16602,-0.21484,-0.18188,-0.22705,-0.23193,-0.17822,-0.22217,-0.18188,-0.17212,-0.19287,-0.25757,-0.22705,-0.26001,-0.30151,-0.29297,-0.26611,-0.33447,-0.32471,-0.35767,-0.4126,-0.49927,-0.51392,-0.54688,-0.52124,-0.43945,-0.40771,-0.29907,-0.20264,-0.14282,-0.14648,-0.19531,-0.15503,-0.19653,-0.17944,-0.11841,-0.15259,-0.095215,-0.078125,-0.10376,-0.16602,-0.11475,-0.12451,-0.090332,-0.015869,-0.015869,0.06958,0.10498,0.11475,0.068359,0.083008,0.029297,-0.0036621,0.029297,0.081787,0.028076,0.062256,0.074463,0.070801,0.029297,0.079346,0.065918,0.091553,0.17578,0.18677,0.30151,0.38086,0.41382,0.42236,0.41992,0.49805,0.50171,0.53345,0.5835,0.55664,0.60059,0.59937,0.58105,0.53955,0.54932,0.4895,0.448,0.44067,0.46265,0.40039,0.40771,0.36621,0.2832,0.19775,0.22461,0.19653,0.22705,0.2771,0.25146,0.3186,0.35645,0.3479,0.32349,0.36865,0.32959,0.32227,0.35278,0.39185,0.33081,0.34912,0.31006,0.21362,0.10254,0.078125,0.0097656,0.025635,0.073242,0.050049,0.11841,0.14893,0.13672,0.090332,0.12207,0.058594,0.03418,0.068359,0.13672,0.11841,0.18311,0.21606,0.22217,0.2002,0.27954,0.25146,0.26123,0.29785,0.2478,0.31006,0.31616,0.271,0.18555,0.2063,0.13916,0.10376,0.12085,0.17944,0.13428,0.18555,0.19897,0.15625,0.087891,0.10986,0.05127,0.056152,0.11719,0.093994,0.16724,0.20142,0.19775,0.14038,0.17578,0.12573,0.11108,0.14404,0.22827,0.20508,0.24536,0.23682,0.16357,0.059814,0.050049,-0.057373,-0.10742,-0.097656,-0.15381,-0.093994,-0.042725,-0.041504,-0.075684,-0.01709,-0.042725,-0.054932,-0.013428,0.042725,-0.0012207,0.05249,0.048828,0.010986,-0.048828,0.0061035,-0.025635,-0.0097656,0.032959,0.084229,0.048828,0.050049,0.0073242,-0.078125,-0.081787,-0.16602,-0.19775,-0.16846,-0.08667,-0.080566,0.013428,0.074463,0.12695,0.14404,0.24414,0.25757,0.28442,0.36133,0.3418,0.40283,0.41016,0.37109,0.28931,0.26733,0.15747,0.059814,0.0097656,0.0036621,-0.080566,-0.054932,-0.062256,-0.080566,-0.11353,-0.040283,-0.023193,0.032959,0.11108,0.21118,0.20996,0.27954,0.28809,0.24414,0.25757,0.16602,0.075684,0.026855,-0.028076,-0.14526,-0.16113,-0.19775,-0.25391,-0.3186,-0.27466,-0.2771,-0.24048,-0.14771,-0.13916,-0.048828,0.01709,0.032959,0.029297,-0.0061035,0.064697,0.05249,0.091553,0.16968,0.15381,0.21362,0.23682,0.20508,0.15137,0.17334,0.098877,0.056152,0.057373,-0.014648,0.0354,0.063477,0.05127,0.0085449,0.065918,0.040283,0.019531,0.05249,0.11108,0.078125,0.14404,0.15015,0.11475,0.056152,0.10864,0.072021,0.080566,0.13062,0.19897,0.15381,0.19653,0.18066,0.12695,0.15015,0.083008,0.037842,0.063477,0.13672,0.10742,0.16724,0.18555,0.16113,0.097656,0.13062,0.080566,0.048828,0.046387,0.079346,-0.01709,-0.031738,-0.10376,-0.21606,-0.34302,-0.3772,-0.47607,-0.53711,-0.56641,-0.61157,-0.60181,-0.5957,-0.59326,-0.61279,-0.57373,-0.52612,-0.49438,-0.43457,-0.36743,-0.35767,-0.30029,-0.30518,-0.32593,-0.37476,-0.31982,-0.29175,-0.19653,-0.065918,-0.012207,0.13672,0.20142,0.23438,0.21484,0.24048,0.18555,0.14648,0.13916,0.15747,0.090332,0.070801,0.029297,-0.065918,-0.18188,-0.17944,-0.23315,-0.21484,-0.1416,-0.12939,-0.025635,0.048828,0.085449,0.10742,0.19653,0.20142,0.21729,0.26001,0.31006,0.26489,0.30396,0.2771,0.19775,0.079346,0.058594,-0.05127,-0.098877,-0.087891,-0.15381,-0.12573,-0.11597,-0.15625,-0.21118,-0.17822,-0.2002,-0.19653,-0.13428,-0.047607,-0.046387,0.045166,0.085449,0.095215,0.074463,0.14038,0.12451,0.16968,0.25391,0.32593,0.3186,0.35522,0.33813,0.27222,0.17334,0.11475,-0.0024414,-0.023193,-0.0061035,-0.063477,-0.023193,-0.0024414,-0.032959,-0.087891,-0.021973,-0.064697,-0.058594,-0.0012207,-0.040283,0.030518,0.079346,0.080566,0.057373,0.11475,0.079346,0.045166,0.085449,0.17456,0.18921,0.29297,0.33936,0.32227,0.25635,0.29663,0.23926,0.22339,0.26611,0.32349,0.30273,0.36743,0.3894,0.36743,0.41382,0.38086,0.36133,0.3772,0.42603,0.38452,0.43579,0.44312,0.40161,0.32959,0.3479,0.28564,0.25391,0.28442,0.25269,0.29297,0.30273,0.27588,0.2063,0.10376,0.10132,0.037842,0.045166,0.10498,0.076904,0.12817,0.16846,0.15259,0.089111,0.11353,0.030518,-0.047607,-0.056152,-0.14648,-0.14282,-0.13062,-0.14404,-0.20142,-0.18433,-0.20752,-0.21851,-0.18188,-0.12329,-0.12207,-0.054932,-0.026855,-0.028076,-0.078125,-0.0354,-0.061035,-0.068359,-0.030518,-0.057373,0.014648,0.070801,0.089111,0.054932,0.085449,0.050049,0.042725,0.098877,0.18555,0.18921,0.271,0.3064,0.29907,0.25635,0.271,0.20142,0.13672,0.11719,0.12573,0.032959,0.032959,-0.021973,-0.075684,-0.15259,-0.15991,-0.24414,-0.29541,-0.33936,-0.44312,-0.49438,-0.56641,-0.66528,-0.74829,-0.76538,-0.75806,-0.71167,-0.6189,-0.47974,-0.38086,-0.2417,-0.15381,-0.11475,-0.10132,0,0.030518,0.11475,0.21851,0.25391,0.36499,0.42725,0.45898,0.4248,0.46143,0.40039,0.3418,0.34424,0.35889,0.28198,0.29297,0.26001,0.17944,0.095215,0.10254,0.023193,0.013428,0.032959,-0.025635,0.021973,0.026855,-0.0061035,-0.05127,-0.11353,-0.076904,-0.11475,-0.092773,-0.031738,-0.047607,0.05249,0.074463,0.062256,0.0073242,0.030518,-0.053711,-0.092773,-0.10864,-0.19775,-0.19897,-0.21484,-0.28564,-0.39673,-0.47119,-0.58472,-0.66406,-0.65674,-0.58472,-0.50537,-0.36865,-0.27222,-0.22705,-0.23071,-0.13916,-0.15259,-0.12085,-0.031738,-0.013428,0.12085,0.19897,0.24414,0.23438,0.30151,0.2771,0.25513,0.2832,0.33691,0.29175,0.33813,0.3418,0.30396,0.22705,0.24048,0.17944,0.16602,0.21851,0.18921,0.26367,0.30518,0.29663,0.24414,0.26123,0.18555,0.12085,0.10498,0.097656,-0.019531,-0.032959,-0.093994,-0.15869,-0.25757,-0.26978,-0.3186,-0.31128,-0.23315,-0.22095,-0.12817,-0.056152,-0.021973,-0.028076,-0.072021,-0.012207,-0.021973,0.026855,0.11108,0.11108,0.17456,0.19653,0.16602,0.10132,0.11353,0.05249,0.026855,0.037842,0.10742,0.090332,0.15015,0.17822,0.16724,0.22583,0.19165,0.17578,0.20996,0.25757,0.22461,0.28687,0.29663,0.25269,0.18311,0.19897,0.13672,0.11353,0.14893,0.13306,0.21118,0.29907,0.33325,0.33325,0.31494,0.38208,0.36499,0.40039,0.45044,0.4248,0.4895,0.50049,0.47852,0.41016,0.41992,0.34424,0.30029,0.30273,0.33569,0.28564,0.32227,0.31372,0.25024,0.17944,0.20386,0.14648,0.14771,0.19409,0.15259,0.22705,0.25513,0.23682,0.18555,0.21729,0.18677,0.16724,0.18066,0.23926,0.17944,0.21851,0.20996,0.14893,0.074463,0.10132,0.062256,0.08667,0.1416,0.11353,0.16235,0.20264,0.19653,0.15991,0.22217,0.20264,0.19409,0.26367,0.34424,0.3125,0.354,0.33447,0.271,0.19043,0.22461,0.1709,0.15381,0.19165,0.17456,0.24048,0.25635,0.24414,0.18921,0.22705,0.177,0.13428,0.13916,0.17456,0.10498,0.12085,0.10864,0.03418,0.023193,-0.072021,-0.12695,-0.12695,-0.08667,-0.11597,-0.061035,-0.047607,-0.064697,-0.12085,-0.1062,-0.15503,-0.16968,-0.13672,-0.068359,-0.080566,-0.028076,-0.012207,-0.019531,-0.074463,-0.040283,-0.064697,-0.050049,0.013428,0.0036621,0.087891,0.13184,0.13306,0.10132,0.15137,0.11963,0.10742,0.13428,0.19165,0.16968,0.23682,0.2478,0.21118,0.14893,0.17822,0.13306,0.12085,0.15991,0.11597,0.18799,0.22461,0.21484,0.16357,0.17944,0.10742,0.06958,0.090332,0.14404,0.11597,0.18555,0.22461,0.21973,0.17578,0.21362,0.15625,0.12939,0.14648,0.15625,0.076904,0.075684,0.032959,-0.021973,-0.074463,-0.041504,-0.075684,-0.042725,0.023193,0.0012207,0.090332,0.1355,0.13428,0.10376,0.15259,0.093994,0.064697,0.095215,0.12451,0.079346,0.10132,0.074463,0.0097656,0.014648,-0.084229,-0.15625,-0.19165,-0.20386,-0.28564,-0.27222,-0.29175,-0.35034,-0.42603,-0.41992,-0.46631,-0.47852,-0.41992,-0.32959,-0.30273,-0.20996,-0.16602,-0.18188,-0.20752,-0.15015,-0.18921,-0.16846,-0.10254,-0.12329,-0.040283,0.032959,0.039063,0.013428,0.080566,0.057373,0.054932,0.11963,0.19897,0.177,0.23315,0.24048,0.18677,0.11719,0.14648,0.073242,0.046387,0.045166,-0.025635,0.021973,0.032959,0.029297,-0.0097656,0.061035,0.042725,0.043945,0.079346,0.13794,0.096436,0.14404,0.14893,0.090332,0.11597,0.061035,0.019531,0.023193,0.068359,0.0354,0.081787,0.11353,0.12085,0.078125,0.11108,0.072021,0.053711,0.083008,0.15747,0.11475,0.17578,0.17334,0.10864,0.0012207,-0.0097656,-0.087891,-0.11719,-0.087891,-0.10498,-0.037842,0.0073242,-0.0097656,-0.047607,-0.018311,-0.064697,-0.11108,-0.1062,-0.075684,-0.15259,-0.15137,-0.18188,-0.25024,-0.33447,-0.33569,-0.39795,-0.42236,-0.4126,-0.45532,-0.42725,-0.45532,-0.51514,-0.59204,-0.63477,-0.67749,-0.67993,-0.63599,-0.57983,-0.57007,-0.54321,-0.53711,-0.53467,-0.55054,-0.48584,-0.44556,-0.36011,-0.23438,-0.19653,-0.063477,-0.0012207,0.0024414,-0.048828,-0.036621,-0.10742,-0.15503,-0.16602,-0.14771,-0.19409,-0.16113,-0.177,-0.24414,-0.3479,-0.41992,-0.5835,-0.72632,-0.91309,-1.1316,-1.2317,-1.2659,-1.2061,-1.1267,-1.0901,-1.0193,-0.92529,-0.77515,-0.57617,-0.41382,-0.19043,-0.041504,0.041504,0.078125,0.2002,0.21729,0.27222,0.36255,0.42603,0.40283,0.44189,0.43213,0.3894,0.43579,0.40039,0.38086,0.4126,0.47241,0.46509,0.54443,0.57739,0.53833,0.47241,0.47852,0.38818,0.33447,0.34302,0.26611,0.29053,0.30029,0.2832,0.22583,0.25024,0.17944,0.12329,0.11597,0.15259,0.1123,0.16602,0.18311,0.17212,0.13062,0.19409,0.16846,0.16846,0.2478,0.26367,0.34058,0.42969,0.45288,0.43457,0.47485,0.41626,0.35767,0.35034,0.36987,0.31616,0.34912,0.36865,0.33569,0.28076,0.32349,0.2771,0.26611,0.30273,0.26733,0.30396,0.32227,0.29175,0.22461,0.12451,0.12085,0.057373,0.043945,0.075684,0.037842,0.092773,0.12817,0.1355,0.10986,0.16968,0.15625,0.17822,0.23926,0.33203,0.31982,0.36255,0.35522,0.29541,0.29785,0.19775,0.10742,0.040283,0.023193,-0.084229,-0.068359,-0.10254,-0.16357,-0.2478,-0.25635,-0.32837,-0.36377,-0.3479,-0.31494,-0.35645,-0.35645,-0.40649,-0.49805,-0.57495,-0.60181,-0.67017,-0.69702,-0.67505,-0.65186,-0.55298,-0.45776,-0.38086,-0.35278,-0.26978,-0.25146,-0.20508,-0.11841,-0.015869,-0.019531,0.020752,-0.014648,-0.085449,-0.20508,-0.25024,-0.37354,-0.45288,-0.46387,-0.48218,-0.35889,-0.21484,-0.13062,-0.10498,-0.019531,-0.021973,-0.037842,-0.0061035,0.030518,-0.058594,-0.040283,-0.097656,-0.20508,-0.33813,-0.36133,-0.4187,-0.40283,-0.31128,-0.28442,-0.14893,-0.059814,-0.020752,-0.015869,-0.025635,0.05127,0.045166,0.091553,0.16602,0.15991,0.24414,0.28687,0.3064,0.27588,0.34546,0.31494,0.30884,0.35278,0.32227,0.38452,0.42847,0.43457,0.40405,0.4541,0.42114,0.39429,0.40649,0.42358,0.35645,0.36133,0.32471,0.25635,0.14282,0.15137,0.075684,0.031738,0.032959,0.029297,-0.067139,-0.074463,-0.11963,-0.20264,-0.20874,-0.24292,-0.2417,-0.16602,-0.059814,-0.042725,0.054932,0.1001,0.11353,0.080566,0.14038,0.10742,0.074463,0.081787,0.021973,0.061035,0.081787,0.063477,-0.0024414,-0.084229,-0.084229,-0.13672,-0.10864,-0.048828,-0.048828,0.046387,0.093994,0.1001,0.068359,0.11475,0.078125,0.062256,0.10986,0.090332,0.15747,0.19287,0.18799,0.1416,0.18311,0.12207,0.093994,0.098877,0.12329,0.063477,0.10986,0.097656,0.073242,0.023193,0.057373,0.0097656,-0.0012207,0.057373,0.14893,0.14893,0.21362,0.21851,0.15869,0.16846,0.073242,-0.0097656,-0.015869,-0.0061035,-0.080566,-0.045166,-0.05127,-0.068359,-0.098877,-0.026855,-0.0061035,0.040283,0.12695,0.24414,0.24414,0.30518,0.29175,0.22705,0.14038,0.13306,0.03418,-0.0097656,-0.028076,-0.13672,-0.14893,-0.17822,-0.26855,-0.38696,-0.43579,-0.53345,-0.59448,-0.58228,-0.52979,-0.50537,-0.38086,-0.2771,-0.21362,-0.15015,0.0061035,0.06958,0.18677,0.32227,0.35645,0.46509,0.53711,0.56152,0.5481,0.60547,0.56274,0.52856,0.53467,0.54932,0.46143,0.47363,0.41992,0.3125,0.19897,0.19897,0.11108,0.093994,0.11963,0.070801,0.11597,0.13428,0.10498,0.043945,0.10132,0.064697,0.024414,0.046387,0.11475,0.068359,0.1416,0.16968,0.16113,0.11597,0.17456,0.14648,0.16846,0.2478,0.2478,0.32227,0.3772,0.3833,0.33447,0.3772,0.32227,0.2771,0.29785,0.26245,0.33691,0.41016,0.44434,0.45166,0.40894,0.45288,0.41992,0.40649,0.44556,0.41626,0.45898,0.47363,0.44556,0.35889,0.36133,0.27222,0.19897,0.15869,0.18555,0.11963,0.15259,0.1709,0.14893,0.085449,0.13184,0.093994,0.098877,0.14771,0.10132,0.15747,0.18066,0.15991,0.11108,0.14404,0.089111,0.05249,0.072021,0.11597,0.075684,0.1355,0.13672,0.12695,0.087891,0.15259,0.14648,0.19775,0.29541,0.31372,0.41992,0.48218,0.47974,0.448,0.46875,0.38208,0.31372,0.2832,0.27832,0.20508,0.21851,0.19287,0.15381,0.079346,0.10986,0.05249,0.032959,0.070801,0.12329,0.089111,0.13672,0.14282,0.11475,0.18799,0.14893,0.13672,0.18555,0.25024,0.21729,0.28076,0.30762,0.26978,0.2063,0.23926,0.16479,0.15381,0.19409,0.24414,0.18311,0.19165,0.14771,0.045166,0.018311,-0.11353,-0.21851,-0.26001,-0.29663,-0.40405,-0.40894,-0.43823,-0.47485,-0.51025,-0.45288,-0.46143,-0.43335,-0.31494,-0.2832,-0.17212,-0.061035,-0.019531,-0.029297,-0.048828,0.025635,-0.0073242,0.031738,0.091553,0.064697,0.10986,0.11475,0.050049,-0.0354,-0.01709,-0.10498,-0.16724,-0.15259,-0.074463,-0.10376,-0.023193,0.012207,-0.0097656,-0.029297,0.047607,0.01709,0.056152,0.13428,0.11353,0.19531,0.25757,0.26123,0.20264,0.24414,0.18311,0.13794,0.14282,0.18921,0.11719,0.14404,0.15625,0.1355,0.080566,0.12329,0.075684,0.065918,0.090332,0.0354,0.078125,0.032959,-0.025635,-0.11963,-0.10742,-0.16968,-0.19531,-0.14648,-0.045166,-0.047607,0.031738,0.064697,0.046387,0.010986,0.065918,0.041504,0.05127,0.092773,0.062256,0.11475,0.1355,0.12939,0.075684,0.10376,0.050049,0.019531,0.041504,0.10864,0.078125,0.12085,0.12085,0.068359,-0.024414,-0.0036621,-0.061035,-0.054932,-0.025635,-0.072021,-0.021973,0.020752,0.025635,-0.0048828,-0.056152,0,-0.040283,-0.0097656,0.036621,-0.026855,0.029297,0.030518,0.0097656,-0.036621,0.031738,-0.015869,0.010986,0.046387,0.10742,0.085449,0.10864,0.087891,0.021973,0.046387,-0.0354,-0.074463,-0.054932,-0.0073242,-0.062256,0.0036621,0.0073242,-0.01709,-0.056152,-0.015869,-0.080566,-0.090332,-0.040283,-0.098877,-0.01709,0.014648,-0.031738,-0.084229,-0.14648,-0.12451,-0.17578,-0.17456,-0.1416,-0.20508,-0.15503,-0.16113,-0.21606,-0.31738,-0.32104,-0.41138,-0.43945,-0.4126,-0.37476,-0.41504,-0.36621,-0.354,-0.37598,-0.41748,-0.36377,-0.41138,-0.39673,-0.35522,-0.41016,-0.36133,-0.32837,-0.33203,-0.36011,-0.27954,-0.30029,-0.31006,-0.26001,-0.30518,-0.25513,-0.20752,-0.22583,-0.29419,-0.2771,-0.36133,-0.44312,-0.47852,-0.47607,-0.58716,-0.60669,-0.62134,-0.65186,-0.70923,-0.61279,-0.56396,-0.46143,-0.29297,-0.22583,-0.075684,0.025635,0.072021,0.045166,0.096436,0.031738,-0.024414,-0.021973,0.015869,-0.057373,-0.0097656,-0.0012207,-0.021973,-0.087891,-0.029297,-0.074463,-0.08667,-0.053711,-0.079346,-0.0061035,0.054932,0.058594,-0.012207,-0.10376,-0.092773,-0.16724,-0.16357,-0.079346,-0.058594,0.070801,0.15015,0.177,0.16602,0.22095,0.19653,0.17944,0.19409,0.22827,0.14648,0.17578,0.15991,0.13794,0.10742,0.1709,0.16235,0.21851,0.29907,0.32227,0.42236,0.49438,0.49927,0.47241,0.52124,0.48462,0.44556,0.46021,0.51025,0.45898,0.50049,0.50659,0.46875,0.41504,0.45288,0.38818,0.36499,0.38452,0.30884,0.3479,0.32959,0.24902,0.12451,0.074463,-0.078125,-0.1709,-0.20508,-0.22461,-0.32837,-0.31372,-0.32837,-0.36743,-0.39917,-0.3125,-0.31494,-0.25391,-0.13672,-0.10132,0.048828,0.14893,0.19897,0.19409,0.28564,0.26367,0.24414,0.27222,0.32227,0.26001,0.27832,0.24414,0.16846,0.037842,0.031738,-0.08667,-0.15259,-0.15991,-0.26001,-0.24292,-0.21484,-0.23438,-0.26855,-0.16602,-0.16235,-0.15015,-0.076904,-0.096436,-0.021973,0.050049,0.068359,0.032959,-0.056152,-0.0061035,-0.068359,-0.10742,-0.078125,-0.15259,-0.10864,-0.084229,-0.085449,-0.091553,-0.1123,-0.0073242,0.013428,0.080566,0.19897,0.20508,0.29785,0.36865,0.38086,0.33081,0.40039,0.36255,0.35889,0.41992,0.39307,0.48096,0.55298,0.5835,0.57983,0.63477,0.60059,0.5896,0.62256,0.69092,0.64697,0.69092,0.68604,0.66772,0.56885,0.57007,0.47241,0.40649,0.39795,0.30396,0.31494,0.30273,0.26367,0.18555,0.2124,0.14893,0.11597,0.15625,0.22583,0.19775,0.24414,0.22705,0.15747,0.026855,-0.0097656,-0.12695,-0.17578,-0.16113,-0.10132,-0.13428,-0.070801,-0.048828,-0.064697,-0.095215,-0.0073242,-0.0097656,0.062256,0.14893,0.1416,0.25269,0.32349,0.33691,0.3064,0.354,0.3125,0.30518,0.3418,0.29541,0.36499,0.4126,0.40283,0.35889,0.30029,0.34302,0.30518,0.3125,0.34302,0.2832,0.35278,0.3479,0.30518,0.22827,0.25391,0.15991,0.095215,0.097656,0.14282,0.091553,0.17212,0.19775,0.17944,0.15015,0.23438,0.20752,0.24414,0.29663,0.25269,0.31982,0.34302,0.31494,0.2356,0.26367,0.17822,0.12817,0.14038,0.18921,0.10986,0.15015,0.14038,0.096436,0.042725,0.10986,0.062256,0.070801,0.13062,0.073242,0.12817,0.16846,0.13672,0.053711,0.10498,0.043945,-0.010986,0.029297,0.062256,-0.014648,0.041504,0.032959,-0.015869,-0.054932,0.013428,-0.037842,-0.0073242,0.061035,0.13062,0.10986,0.16113,0.16968,0.12085,0.18921,0.15625,0.13062,0.16846,0.23804,0.19043,0.2478,0.26978,0.22705,0.14526,0.19653,0.13794,0.12451,0.17944,0.13428,0.19165,0.22095,0.2124,0.15503,0.20874,0.15503,0.10986,0.12695,0.19165,0.12695,0.16724,0.17212,0.13916,0.042725,0.073242,-0.0061035,-0.023193,0.018311,-0.057373,0.021973,0.064697,0.064697,0.018311,0.05249,-0.0024414,-0.040283,-0.012207,0.041504,-0.0354,0.024414,0.018311,-0.0073242,-0.064697,0.018311,0.0061035,0.046387,0.13062,0.25757,0.26855,0.32593,0.35522,0.34424,0.40161,0.36377,0.34424,0.36743,0.42847,0.38574,0.42236,0.41504,0.37109,0.26733,0.26733,0.16602,0.12329,0.15259,0.070801,0.11963,0.1355,0.10376,0.042725,0.091553,0.023193,-0.015869,0.012207,0.053711,0.014648,0.10376,0.12085,0.08667,0.015869,0.032959,-0.036621,-0.036621,-0.0036621,0.068359,0.029297,0.1001,0.11475,0.089111,0.16357,0.12939,0.093994,0.12329,0.16968,0.10864,0.18188,0.20874,0.18188,0.12451,0.17822,0.12329,0.10132,0.14038,0.20996,0.14893,0.19897,0.19897,0.1355,0.05127,0.070801,-0.01709,-0.020752,0.0073242,-0.095215,-0.0354,-0.0085449,-0.037842,-0.085449,-0.0012207,-0.0354,-0.01709,0.087891,0.20264,0.19653,0.29541,0.32959,0.28564,0.35522,0.29907,0.24536,0.2417,0.27954,0.20508,0.27344,0.27832,0.24048,0.14893,0.16357,0.079346,-0.0061035,-0.013428,-0.11963,-0.11353,-0.098877,-0.11597,-0.19653,-0.28076,-0.25024,-0.30884,-0.29785,-0.23804,-0.29419,-0.26367,-0.22461,-0.24048,-0.27954,-0.19653,-0.20386,-0.18433,-0.093994,0.01709,-0.015869,0.074463,0.078125,0.036621,0.06958,-0.0036621,-0.073242,-0.10498,-0.076904,-0.12573,-0.080566,-0.05127,-0.075684,-0.15381,-0.11108,-0.15625,-0.17822,-0.13184,-0.045166,-0.080566,-0.0097656,0.018311,-0.025635,-0.081787,-0.046387,-0.12329,-0.16479,-0.18799,-0.30151,-0.29907,-0.33936,-0.3772,-0.43945,-0.39185,-0.42358,-0.41138,-0.34912,-0.26001,-0.27832,-0.19165,-0.15259,-0.14771,-0.17822,-0.08667,-0.11475,-0.084229,-0.0085449,-0.03418,0.058594,0.096436,0.11353,0.10132,0.18555,0.15503,0.15015,0.19409,0.27466,0.23071,0.30029,0.30029,0.27222,0.19897,0.25024,0.18555,0.17944,0.22461,0.2832,0.23804,0.29175,0.26733,0.20874,0.24658,0.177,0.12573,0.13306,0.15625,0.059814,0.084229,0.058594,-0.024414,-0.12695,-0.10498,-0.17334,-0.18555,-0.12085,-0.17212,-0.092773,-0.042725,-0.047607,-0.080566,-0.14282,-0.084229,-0.10498,-0.05127,0.0354,-0.0073242,0.11719,0.17822,0.18921,0.15503,0.23682,0.18677,0.17822,0.23804,0.17944,0.24536,0.27588,0.271,0.20874,0.26367,0.20264,0.15869,0.15625,0.2063,0.14038,0.20996,0.22217,0.17944,0.10742,0.15381,0.092773,0.080566,0.12329,0.19165,0.12939,0.17334,0.16602,0.078125,0.10498,0.013428,-0.070801,-0.074463,-0.056152,-0.15137,-0.13794,-0.14526,-0.17822,-0.23682,-0.18188,-0.22339,-0.23926,-0.19653,-0.21729,-0.12939,-0.054932,-0.041504,-0.074463,-0.15625,-0.12695,-0.21362,-0.24048,-0.22583,-0.29907,-0.27954,-0.27954,-0.31982,-0.3772,-0.3186,-0.36377,-0.3772,-0.3418,-0.37109,-0.28564,-0.22217,-0.19287,-0.21484,-0.28442,-0.22583,-0.28564,-0.3125,-0.29175,-0.34912,-0.26489,-0.21362,-0.20996,-0.25269,-0.18311,-0.21729,-0.2124,-0.15259,-0.074463,-0.12695,-0.090332,-0.097656,-0.12695,-0.20264,-0.16602,-0.22095,-0.19409,-0.12451,-0.14404,-0.05249,-0.028076,-0.031738,-0.064697,-0.0061035,-0.057373,-0.080566,-0.059814,0.012207,-0.046387,0.031738,0.037842,0.0024414,-0.074463,-0.015869,-0.070801,-0.078125,-0.036621,-0.10376,-0.048828,-0.021973,-0.058594,-0.13306,-0.08667,-0.13428,-0.1416,-0.059814,0.059814,0.056152,0.18799,0.24414,0.23071,0.18799,0.2478,0.17212,0.16602,0.19165,0.21606,0.16724,0.20752,0.19653,0.15625,0.20996,0.14038,0.12695,0.17578,0.24902,0.20752,0.27832,0.29663,0.25513,0.17456,0.20508,0.11597,0.070801,0.085449,-0.029297,0.0097656,0,-0.063477,-0.14648,-0.11719,-0.20508,-0.25391,-0.22583,-0.14526,-0.17578,-0.064697,0.0097656,0.0097656,-0.01709,0.11597,0.096436,0.13062,0.21851,0.17578,0.24414,0.3064,0.29907,0.20752,0.22217,0.12695,0.046387,0.05249,0.11597,0.079346,0.16235,0.21484,0.22461,0.18555,0.2771,0.26733,0.2832,0.36987,0.35034,0.43945,0.50659,0.51147,0.46631,0.40039,0.44922,0.40039,0.40161,0.45654,0.40283,0.4248,0.42358,0.36377,0.2478,0.26001,0.16602,0.090332,0.087891,0.14038,0.068359,0.097656,0.1001,0.048828,0.10986,0.05127,0.0036621,0.019531,0.062256,0.0024414,0.062256,0.075684,0.037842,-0.045166,0.012207,-0.065918,-0.067139,-0.015869,-0.061035,0.013428,0.062256,0.06958,0.048828,0.11963,0.053711,0.024414,0.037842,0.079346,0.028076,0.087891,0.10132,0.089111,0.023193,0.070801,0.01709,-0.0097656,0.01709,0.065918,0.0024414,0.041504,0.025635,-0.046387,-0.13306,-0.091553,-0.16602,-0.18433,-0.15381,-0.22095,-0.15625,-0.089111,-0.08667,-0.11963,-0.01709,-0.041504,-0.043945,0.029297,0.097656,0.037842,0.10986,0.10986,0.075684,0.029297,0.092773,0.058594,0.10132,0.18555,0.17212,0.3064,0.37354,0.39063,0.354,0.42603,0.36987,0.33936,0.36987,0.41992,0.34424,0.38086,0.36255,0.28931,0.19775,0.22461,0.14038,0.11353,0.15381,0.06958,0.12207,0.1416,0.10498,0.020752,0.054932,-0.025635,-0.076904,-0.056152,-0.015869,-0.1062,-0.062256,-0.064697,-0.11475,-0.18555,-0.12085,-0.17334,-0.15503,-0.087891,-0.12329,-0.0097656,0.073242,0.084229,0.062256,0.16235,0.13672,0.11108,0.15259,0.22583,0.14893,0.19043,0.17578,0.10864,-0.0061035,0.025635,-0.087891,-0.14648,-0.13916,-0.24658,-0.21484,-0.22095,-0.23438,-0.27588,-0.19897,-0.20264,-0.19653,-0.12329,-0.0061035,-0.023193,0.068359,0.11963,0.11353,0.05127,0.11841,0.068359,0.037842,0.091553,0.14648,0.072021,0.10132,0.068359,-0.023193,0.021973,-0.074463,-0.12451,-0.10986,-0.070801,-0.13306,-0.072021,-0.080566,-0.11353,-0.18311,-0.13062,-0.18188,-0.19287,-0.15991,-0.2124,-0.1416,-0.11353,-0.13306,-0.19775,-0.17456,-0.23315,-0.27466,-0.25391,-0.20142,-0.25146,-0.19165,-0.18311,-0.19653,-0.25879,-0.18921,-0.24292,-0.26001,-0.23193,-0.28809,-0.22705,-0.18311,-0.17578,-0.20874,-0.2478,-0.15259,-0.16724,-0.10986,-0.026855,-0.084229,-0.0036621,0.012207,-0.061035,-0.15503,-0.15991,-0.26611,-0.30396,-0.26978,-0.20142,-0.21606,-0.14648,-0.12085,-0.13306,-0.15747,-0.076904,-0.084229,-0.0073242,0.1001,0.065918,0.17456,0.20142,0.20142,0.16357,0.24414,0.20508,0.20752,0.27222,0.36499,0.3125,0.38086,0.39063,0.32227,0.24292,0.26611,0.16357,0.11475,0.11475,0.0012207,0.032959,0.048828,0.0085449,-0.05127,0.037842,-0.013428,-0.0048828,0.073242,0.16235,0.12695,0.23193,0.28076,0.25757,0.21851,0.30273,0.26855,0.3064,0.38696,0.34668,0.41504,0.47485,0.46875,0.41138,0.4541,0.37842,0.3125,0.30884,0.33691,0.2124,0.20874,0.16602,0.046387,0.042725,-0.041504,-0.08667,-0.048828,0.062256,0.042725,0.16968,0.24658,0.271,0.24048,0.32837,0.28564,0.25391,0.28931,0.35645,0.29785,0.35522,0.35889,0.3125,0.22217,0.26367,0.20264,0.17456,0.2124,0.14648,0.19165,0.21362,0.21362,0.15869,0.21484,0.17212,0.15869,0.19409,0.26489,0.22583,0.27832,0.27832,0.25269,0.17822,0.22827,0.18311,0.17944,0.23804,0.18921,0.24048,0.27222,0.24048,0.15747,0.18555,0.098877,0.015869,0.0073242,0.026855,-0.085449,-0.068359,-0.10986,-0.17578,-0.2771,-0.21362,-0.25635,-0.23804,-0.14771,-0.16602,-0.047607,0.029297,0.029297,0.018311,0.095215,0.040283,0.043945,0.084229,0.1416,0.10132,0.18921,0.23071,0.21729,0.17822,0.24414,0.19653,0.2002,0.25391,0.30884,0.25879,0.28687,0.26733,0.19775,0.22461,0.11597,0.053711,0.05127,0.087891,0.01709,0.083008,0.081787,0.056152,-0.014648,0.045166,-0.040283,-0.041504,0.0036621,0.046387,-0.015869,0.046387,0.03418,-0.0085449,-0.042725,0.048828,-0.0024414,0.048828,0.10986,0.048828,0.12817,0.17822,0.13672,0.081787,0.15259,0.10376,0.097656,0.14893,0.22827,0.17944,0.24292,0.23193,0.18188,0.12695,0.2063,0.18433,0.23438,0.3125,0.27344,0.36499,0.41992,0.41626,0.36865,0.42725,0.36133,0.32349,0.35889,0.3064,0.34546,0.3772,0.38086,0.3186,0.24658,0.29053,0.2063,0.19409,0.22339,0.13672,0.18311,0.18311,0.14282,0.072021,0.12939,0.075684,0.048828,0.083008,0.15625,0.11108,0.17944,0.21851,0.17212,0.11597,0.19409,0.16846,0.19287,0.25879,0.22461,0.30273,0.3418,0.32837,0.2478,0.29541,0.23315,0.18799,0.19775,0.25757,0.17334,0.21362,0.21484,0.15869,0.062256,0.10864,0.045166,0.0354,0.089111,0.05249,0.11841,0.16724,0.16602,0.10132,0.13428,0.072021,0.021973,0.032959,0.080566,0.0097656,0.050049,0.041504,-0.012207,-0.087891,-0.045166,-0.10498,-0.097656,-0.05127,-0.10742,-0.028076,-0.0024414,-0.021973,-0.05249,-0.10132,-0.012207,-0.014648,0.032959,0.10498,0.078125,0.14404,0.17822,0.16602,0.096436,0.15259,0.10132,0.096436,0.14648,0.078125,0.14771,0.14526,0.097656,0.0048828,-0.0061035,-0.10986,-0.18433,-0.22339,-0.22705,-0.33447,-0.31494,-0.3479,-0.4248,-0.54932,-0.53711,-0.59692,-0.59204,-0.51025,-0.41138,-0.43213,-0.38208,-0.3833,-0.42358,-0.36011,-0.39917,-0.41992,-0.37842,-0.32471,-0.36011,-0.29175,-0.25879,-0.27588,-0.32471,-0.24048,-0.2832,-0.28198,-0.20386,-0.25757,-0.15869,-0.090332,-0.070801,-0.093994,-0.11475,-0.025635,-0.045166,-0.0061035,0.075684,0.019531,0.081787,0.11719,0.081787,0.0036621,0.057373,-0.015869,-0.05127,-0.029297,-0.13306,-0.093994,-0.070801,-0.081787,-0.11475,-0.16113,-0.062256,-0.05127,0.0097656,0.10132,0.087891,0.18433,0.23193,0.23193,0.17822,0.25513,0.20508,0.17212,0.2124,0.27466,0.18555,0.22583,0.21362,0.14893,0.19775,0.11841,0.046387,0.032959,0.068359,-0.023193,0.019531,0.013428,-0.03418,-0.11963,-0.061035,-0.1001,-0.091553,-0.018311,-0.013428,0.11597,0.21118,0.26733,0.271,0.21729,0.27588,0.23438,0.21118,0.22461,0.14282,0.16357,0.17578,0.15137,0.079346,0.1355,0.074463,0.026855,0.042725,0.093994,0.028076,0.078125,0.098877,0.081787,0.041504,0.11963,0.080566,0.091553,0.16357,0.12085,0.21484,0.23438,0.21973,0.15381,0.19897,0.11597,0.075684,0.081787,0.11963,0.05249,0.11841,0.12451,0.097656,0.037842,0.089111,0.040283,0.057373,0.11108,0.072021,0.1709,0.23193,0.22949,0.18677,0.25391,0.2002,0.17212,0.21118,0.2832,0.23682,0.3125,0.31616,0.27466,0.19531,0.22217,0.13672,0.097656,0.10132,-0.0048828,0.0036621,-0.026855,-0.095215,-0.19287,-0.17212,-0.24048,-0.26367,-0.18677,-0.075684,-0.080566,0.056152,0.087891,0.076904,0.019531,0.078125,0.013428,-0.0073242,0.047607,0.081787,0.032959,0.056152,0.036621,-0.045166,0.029297,-0.029297,-0.05127,-0.0024414,0.063477,-0.018311,0.056152,0.063477,0.01709,-0.05249,0.01709,-0.047607,-0.05127,0.018311,0.056152,0.0048828,0.041504,0.029297,-0.028076,-0.072021,0,-0.053711,-0.0073242,0.067139,-0.012207,0.048828,0.064697,0.032959,-0.0354,0.059814,-0.0012207,-0.0085449,0.062256,0.0061035,0.081787,0.15259,0.17944,0.14038,0.21484,0.177,0.13184,0.14526,0.19287,0.12939,0.15625,0.14648,0.064697,-0.048828,-0.015869,-0.078125,-0.1123,-0.080566,-0.12695,-0.043945,0.030518,0.048828,0.029297,0.11108,0.074463,0.042725,0.070801,0.12939,0.089111,0.15625,0.20874,0.22217,0.16602,0.21851,0.177,0.14038,0.177,0.13916,0.20874,0.25635,0.26978,0.23438,0.18311,0.25513,0.22217,0.25757,0.32227,0.28442,0.37354,0.37476,0.33813,0.26855,0.29053,0.18677,0.11963,0.093994,0.12329,0.041504,0.089111,0.085449,0.026855,0.074463,-0.0012207,-0.042725,-0.026855,0.029297,-0.029297,0.05127,0.075684,0.068359,0.0036621,0.043945,-0.010986,-0.023193,0.012207,0.078125,0.0354,0.10376,0.10742,0.06958,0.012207,0.059814,0.0073242,0.023193,0.079346,0.15625,0.11108,0.16724,0.15625,0.11597,0.19287,0.13672,0.11108,0.16113,0.22827,0.16724,0.2124,0.18188,0.10498,0.018311,0.0354,-0.054932,-0.065918,-0.050049,-0.14282,-0.076904,-0.068359,-0.090332,-0.13916,-0.19531,-0.14038,-0.16968,-0.13306,-0.065918,-0.12329,-0.054932,-0.020752,-0.058594,-0.1062,-0.024414,-0.046387,-0.023193,0.068359,0.019531,0.08667,0.12085,0.096436,0.0073242,0.05127,-0.0073242,-0.046387,-0.0061035,0.068359,0.0097656,0.076904,0.10864,0.076904,-0.0012207,0.062256,-0.032959,-0.054932,-0.013428,-0.081787,-0.019531,0.025635,0.0097656,-0.042725,0.042725,-0.0036621,-0.019531,0.05249,0.12085,0.075684,0.14038,0.15137,0.10864,0.023193,0.072021,0.0097656,-0.0097656,0.048828,0.092773,0.0354,0.076904,0.079346,0.043945,0.10864,0.067139,0.050049,0.091553,0.17578,0.15991,0.24658,0.29663,0.29419,0.2356,0.28076,0.22217,0.19775,0.22461,0.16235,0.23315,0.27954,0.2771,0.22949,0.25757,0.19531,0.16235,0.177,0.23438,0.17578,0.2478,0.25757,0.2417,0.17212,0.2063,0.13428,0.10498,0.11963,0.16968,0.097656,0.12817,0.1123,0.059814,0.11841,0.061035,0.029297,0.05127,0.1001,0.050049,0.12329,0.13306,0.090332,0.0097656,0.042725,-0.020752,-0.048828,-0.018311,-0.084229,-0.025635,0.025635,0.0061035,-0.05127,-0.080566,-0.028076,-0.067139,-0.029297,0.030518,-0.0354,0.061035,0.093994,0.070801,0.01709,0.064697,-0.021973,-0.056152,-0.040283,-0.13306,-0.10864,-0.095215,-0.13306,-0.21606,-0.29297,-0.26001,-0.30762,-0.28442,-0.23682,-0.29053,-0.2124,-0.19287,-0.20752,-0.2771,-0.2478,-0.32104,-0.3772,-0.3418,-0.27954,-0.34424,-0.28076,-0.25024,-0.27588,-0.3125,-0.21606,-0.24292,-0.19897,-0.10498,-0.13062,-0.05249,-0.0036621,-0.021973,-0.093994,-0.048828,-0.11597,-0.15015,-0.093994,-0.0036621,-0.032959,0.057373,0.074463,0.041504,-0.020752,0.040283,-0.0354,-0.029297,0.010986,-0.065918,-0.0036621,0.030518,-0.0024414,-0.074463,-0.05249,-0.12573,-0.20508,-0.18311,-0.13306,-0.18555,-0.12451,-0.10132,-0.13794,-0.20508,-0.14526,-0.19409,-0.18188,-0.1123,-0.1416,-0.054932,-0.013428,-0.0024414,-0.029297,-0.085449,-0.0061035,-0.0354,0,0.062256,0.023193,0.097656,0.13428,0.11597,0.059814,0.11841,0.065918,0.059814,0.10498,0.043945,0.11108,0.12939,0.091553,0.015869,0.040283,-0.0354,-0.065918,-0.068359,-0.020752,-0.078125,-0.0073242,0.01709,-0.0036621,-0.045166,0.029297,-0.013428,0.026855,0.11475,0.23071,0.22461,0.30396,0.31494,0.28687,0.34912,0.29297,0.25391,0.27832,0.33691,0.271,0.32349,0.33325,0.30029,0.23193,0.28442,0.22339,0.2124,0.25269,0.3064,0.27954,0.34058,0.33203,0.29541,0.2356,0.27466,0.23193,0.26123,0.32227,0.26245,0.33203,0.35278,0.31006,0.24536,0.28076,0.20752,0.177,0.22217,0.16968,0.25757,0.3186,0.32227,0.28564,0.21851,0.28076,0.23804,0.25513,0.31494,0.2356,0.29297,0.30029,0.23193,0.12817,0.12939,0.021973,-0.040283,-0.014648,-0.091553,-0.029297,0.048828,0.072021,0.031738,0.10742,0.061035,-0.012207,0.014648,0.06958,-0.0036621,0.05127,0.056152,0.012207,-0.080566,-0.023193,-0.10254,-0.15869,-0.12939,-0.177,-0.10376,-0.021973,0,-0.025635,-0.061035,0.031738,-0.013428,0.023193,0.090332,0.053711,0.12451,0.16846,0.13916,0.05249,0.081787,0.0012207,-0.058594,-0.048828,-0.11475,-0.068359,-0.047607,-0.045166,-0.10864,-0.21118,-0.16235,-0.21606,-0.21362,-0.15503,-0.19165,-0.10376,-0.059814,-0.048828,-0.093994,-0.023193,-0.058594,-0.076904,-0.041504,0.046387,0.0097656,0.078125,0.10742,0.095215,0.029297,0.076904,0.013428,-0.015869,0.024414,-0.061035,-0.01709,-0.025635,-0.057373,-0.13184,-0.098877,-0.17334,-0.23315,-0.22827,-0.16357,-0.2002,-0.12817,-0.10498,-0.10986,-0.14893,-0.048828,-0.05249,-0.01709,0.072021,0.037842,0.14771,0.22461,0.22827,0.20752,0.27588,0.22461,0.22095,0.26855,0.33813,0.29663,0.36133,0.36133,0.31982,0.2417,0.28076,0.21362,0.2063,0.23926,0.2832,0.22827,0.26245,0.24658,0.21484,0.28076,0.2063,0.17578,0.19775,0.24536,0.18799,0.24414,0.25146,0.20874,0.11841,0.14038,0.065918,0.032959,0.080566,0.13672,0.092773,0.13672,0.12817,0.080566,0.15259,0.11108,0.084229,0.13794,0.22827,0.19775,0.30396,0.35645,0.3418,0.30151,0.37109,0.30273,0.27222,0.30029,0.31616,0.20996,0.21606,0.16113,0.05249,0.079346,-0.014648,-0.068359,-0.05249,0.0061035,-0.067139,0.0097656,0.019531,0.0061035,-0.036621,0.053711,-0.0085449,-0.015869,0.041504,-0.032959,0.020752,0.046387,0.030518,-0.048828,-0.12207,-0.083008,-0.16846,-0.17578,-0.13062,-0.18555,-0.15259,-0.13062,-0.16357,-0.25757,-0.20508,-0.2478,-0.25757,-0.18555,-0.18311,-0.078125,0.014648,0.045166,0.0085449,0.070801,0.010986,-0.050049,-0.056152,-0.048828,-0.13916,-0.074463,-0.059814,-0.070801,-0.12695,-0.067139,-0.1062,-0.13306,-0.1062,-0.16602,-0.12329,-0.091553,-0.11353,-0.17944,-0.2771,-0.26367,-0.32837,-0.31616,-0.26001,-0.28564,-0.20874,-0.15381,-0.11597,-0.12085,-0.01709,-0.0073242,0.0024414,0.046387,0.13916,0.1001,0.15259,0.15381,0.10742,0.047607,0.10498,0.058594,0.083008,0.1416,0.097656,0.18555,0.2417,0.22827,0.18677,0.23438,0.1709,0.1355,0.16968,0.2356,0.17944,0.23438,0.22583,0.17212,0.068359,0.084229,-0.0061035,-0.020752,-0.0048828,-0.084229,-0.021973,-0.0012207,-0.018311,-0.064697,-0.11719,-0.057373,-0.084229,-0.037842,0.042725,-0.0048828,0.091553,0.10498,0.079346,0.019531,0.065918,0.0073242,0.0036621,0.072021,0.01709,0.078125,0.10498,0.081787,0,0.036621,-0.05127,-0.10742,-0.081787,-0.021973,-0.067139,0.023193,0.057373,0.054932,0.018311,0.1062,0.059814,0.063477,0.12939,0.19287,0.15015,0.21729,0.20874,0.15869,0.24414,0.21362,0.19775,0.25513,0.33203,0.28809,0.36987,0.39429,0.35522,0.27222,0.30396,0.22827,0.18677,0.2063,0.2417,0.15991,0.16968,0.14648,0.075684,0.10986,0.05249,0.015869,0.042725,0.11475,0.063477,0.10742,0.12695,0.090332,0.026855,0.095215,0.050049,0.036621,0.11597,0.20874,0.16357,0.23315,0.2478,0.20874,0.27466,0.23315,0.19287,0.2063,0.25757,0.19165,0.22827,0.22461,0.16602,0.058594,0.065918,-0.032959,-0.080566,-0.10132,-0.18188,-0.14893,-0.11353,-0.12207,-0.17578,-0.26001,-0.20752,-0.2478,-0.22705,-0.16235,-0.20264,-0.15137,-0.15015,-0.19165,-0.27466,-0.23682,-0.28564,-0.29297,-0.22461,-0.10742,-0.11841,-0.029297,0.0097656,-0.0061035,-0.012207,0.081787,0.062256,0.10498,0.16968,0.13916,0.21606,0.26123,0.25879,0.22827,0.29297,0.25757,0.26733,0.3064,0.36377,0.32837,0.39673,0.40283,0.37231,0.3186,0.35767,0.3125,0.32959,0.36499,0.31616,0.38452,0.38818,0.35767,0.29053,0.31006,0.22461,0.15259,0.15747,0.2002,0.1123,0.15381,0.14893,0.10376,0.05249,0.11108,0.054932,0.073242,0.13062,0.072021,0.15259,0.16724,0.13672,0.083008,0.13672,0.059814,0.023193,0.070801,0.1355,0.06958,0.16602,0.17456,0.14648,0.1001,0.16724,0.11108,0.12451,0.18188,0.11353,0.17334,0.19897,0.15991,0.074463,0.11475,0.040283,-0.0097656,0.015869,0.054932,-0.0085449,0.075684,0.097656,0.061035,-0.0048828,0.063477,-0.020752,-0.037842,-0.0073242,-0.095215,-0.05249,-0.05249,-0.11353,-0.22827,-0.34424,-0.34424,-0.44922,-0.45776,-0.42725,-0.49683,-0.45898,-0.43823,-0.46509,-0.52612,-0.46143,-0.46875,-0.44678,-0.34424,-0.33813,-0.22217,-0.1355,-0.090332,-0.11108,-0.046387,-0.070801,-0.098877,-0.057373,0.0354,-0.0012207,0.10742,0.15259,0.15991,0.12939,0.20874,0.17578,0.16479,0.20752,0.15991,0.2063,0.24658,0.23438,0.14771,0.17212,0.089111,0.0024414,-0.042725,-0.025635,-0.1355,-0.12329,-0.1416,-0.21484,-0.31738,-0.30273,-0.36621,-0.37598,-0.33569,-0.26123,-0.2832,-0.23804,-0.22949,-0.26611,-0.33813,-0.30029,-0.33936,-0.32471,-0.26978,-0.28198,-0.17944,-0.11597,-0.091553,-0.10498,-0.041504,-0.061035,-0.048828,0.015869,0.10864,0.092773,0.14648,0.17578,0.16235,0.23193,0.20874,0.20264,0.23682,0.31982,0.31006,0.41626,0.46143,0.46509,0.41748,0.4541,0.37842,0.3418,0.3479,0.38574,0.29663,0.31616,0.2771,0.19287,0.11108,0.15137,0.089111,0.1001,0.14893,0.080566,0.15259,0.17334,0.14648,0.10376,0.16357,0.11841,0.10498,0.13672,0.19775,0.13062,0.18555,0.17456,0.12207,0.040283,0.062256,0.0012207,0.026855,0.080566,0.041504,0.13794,0.19897,0.19653,0.15869,0.23804,0.20508,0.19287,0.26367,0.3418,0.27588,0.32471,0.3064,0.22827,0.13184,0.16968,0.087891,0.062256,0.10498,0.046387,0.13062,0.16602,0.17578,0.12939,0.19775,0.16602,0.15869,0.21484,0.3186,0.30029,0.37354,0.41382,0.38208,0.30273,0.36377,0.30273,0.28687,0.33081,0.24414,0.26367,0.26367,0.21118,0.12085,0.14038,0.057373,-0.0061035,0.0097656,0.05249,0.0036621,0.080566,0.1123,0.091553,0.031738,0.10498,0.068359,0.075684,0.12695,0.074463,0.12695,0.15747,0.14038,0.079346,0.12085,0.056152,0.01709,0.048828,0.10742,0.065918,0.12451,0.13306,0.11475,0.036621,0.095215,0.053711,0.058594,0.11475,0.075684,0.16235,0.20508,0.20142,0.14648,0.16968,0.091553,0.05249,0.06958,0.13794,0.10864,0.177,0.21729,0.21362,0.15625,0.19653,0.14038,0.10986,0.11475,0.01709,0.032959,0.026855,-0.01709,-0.061035,-0.12085,-0.059814,-0.078125,-0.036621,0.037842,-0.0097656,0.097656,0.11963,0.11475,0.062256,0.10986,0.0354,0.010986,0.056152,0.10864,0.067139,0.12085,0.11475,0.062256,0.0073242,0.046387,-0.0097656,0.0024414,0.0097656,-0.054932,0.014648,0.0036621,-0.040283,-0.1001,-0.067139,-0.13916,-0.18433,-0.16602,-0.14404,-0.20874,-0.14648,-0.13794,-0.17334,-0.21118,-0.13306,-0.14648,-0.1001,-0.029297,-0.068359,-0.0012207,0.053711,0.053711,0.0061035,0.06958,0.028076,-0.0024414,0.058594,0.11841,0.048828,0.092773,0.081787,0.032959,-0.0036621,0.072021,0.023193,0.054932,0.12695,0.096436,0.18066,0.2063,0.18921,0.12085,0.177,0.11475,0.062256,0.095215,0.18311,0.14648,0.22461,0.25757,0.20874,0.25635,0.20508,0.14648,0.13428,0.16113,0.087891,0.11475,0.11841,0.081787,-0.0073242,0.032959,-0.032959,-0.046387,0,0.079346,0.05127,0.14282,0.18555,0.16968,0.12085,0.18066,0.12817,0.12451,0.16846,0.12451,0.17944,0.2124,0.19775,0.14038,0.18311,0.11963,0.081787,0.097656,0.15259,0.10376,0.14893,0.14282,0.10254,0.010986,0.032959,-0.029297,-0.05127,-0.030518,-0.087891,-0.023193,-0.012207,-0.029297,-0.10376,-0.084229,-0.16602,-0.22095,-0.22339,-0.18311,-0.23438,-0.15503,-0.11475,-0.10376,-0.13428,-0.045166,-0.074463,-0.048828,0.018311,0.068359,0.020752,0.064697,0.045166,0.0048828,0.054932,-0.019531,-0.03418,-0.0061035,0.062256,0.020752,0.11963,0.14648,0.13672,0.098877,0.15625,0.1001,0.097656,0.1355,0.18311,0.12695,0.16968,0.15991,0.10742,0.15747,0.095215,0.059814,0.087891,0.14404,0.089111,0.15747,0.18799,0.15747,0.096436,0.14648,0.084229,0.064697,0.095215,0.13428,0.089111,0.15747,0.16357,0.12207,0.18921,0.13428,0.10742,0.13428,0.18188,0.10132,0.14404,0.15991,0.11963,0.037842,0.091553,0.023193,0,0.048828,0.12695,0.087891,0.15625,0.17578,0.14038,0.20508,0.18555,0.14648,0.16968,0.2478,0.19897,0.26123,0.29175,0.271,0.20264,0.25269,0.18433,0.13428,0.16602,0.22217,0.14893,0.19165,0.17578,0.1062,0.13428,0.043945,-0.028076,-0.015869,0.03418,-0.040283,0.023193,0.048828,0.029297,-0.05249,0.0073242,-0.056152,-0.070801,-0.025635,-0.063477,0.012207,0.062256,0.080566,0.05127,-0.018311,0.045166,-0.0061035,0.010986,0.062256,0.039063,0.11597,0.16602,0.177,0.13916,0.20752,0.17456,0.16602,0.19409,0.26733,0.2124,0.25269,0.25024,0.18921,0.21362,0.12695,0.062256,0.029297,0.046387,-0.0061035,0.062256,0.064697,0.059814,0.0073242,0.046387,0.0024414,-0.013428,0.048828,0.12695,0.10742,0.19897,0.21362,0.19653,0.13794,0.16357,0.093994,0.06958,0.087891,-0.0061035,0.03418,0.0073242,-0.040283,-0.1062,-0.081787,-0.16968,-0.20996,-0.19043,-0.12695,-0.15625,-0.061035,-0.029297,-0.029297,-0.058594,0.014648,-0.018311,0.021973,0.078125,0.16602,0.14038,0.2124,0.20874,0.16724,0.22583,0.15869,0.11597,0.15015,0.19287,0.12207,0.18799,0.18555,0.13428,0.061035,0.093994,0.029297,0.023193,0.074463,0.1355,0.10132,0.16602,0.16968,0.13428,0.20264,0.14038,0.10498,0.13428,0.18921,0.12207,0.17822,0.19043,0.13916,0.062256,0.10498,0.057373,0.059814,0.12695,0.19531,0.16235,0.22339,0.2356,0.16968,0.22705,0.17944,0.12817,0.15015,0.20874,0.1355,0.20142,0.21484,0.1709,0.087891,0.13306,0.063477,0.032959,0.072021,0.13428,0.073242,0.14038,0.13672,0.075684,0.12817,0.068359,0.010986,0.031738,0.096436,0.05249,0.13062,0.16357,0.14648,0.095215,0.14648,0.089111,0.057373,0.075684,0.14282,0.06958,0.091553,0.084229,0.0073242,0.019531,-0.076904,-0.16235,-0.2002,-0.17578,-0.24414,-0.20142,-0.16113,-0.15503,-0.19897,-0.11597,-0.12451,-0.10742,-0.029297,-0.041504,0.058594,0.12085,0.15381,0.12695,0.18921,0.13428,0.10132,0.12939,0.18555,0.14282,0.21973,0.22583,0.20752,0.14771,0.18433,0.12695,0.093994,0.10498,0.15625,0.073242,0.098877,0.097656,0.029297,-0.05249,-0.0061035,-0.078125,-0.075684,-0.014648,-0.073242,-0.0097656,0.0012207,-0.058594,-0.13306,-0.10498,-0.17212,-0.2002,-0.16357,-0.10376,-0.12329,-0.015869,0.01709,-0.0097656,-0.026855,0.042725,-0.0073242,0.023193,0.046387,0.093994,0.023193,0.025635,-0.036621,-0.13794,-0.15747,-0.28931,-0.38818,-0.4126,-0.40527,-0.47485,-0.40039,-0.35767,-0.32959,-0.33569,-0.2356,-0.23438,-0.18311,-0.079346,-0.10132,0.0048828,0.048828,0.042725,0.0012207,0.056152,-0.012207,-0.067139,-0.047607,-0.0036621,-0.087891,-0.046387,-0.045166,-0.092773,-0.15869,-0.090332,-0.13794,-0.12451,-0.018311,-0.041504,0.045166,0.10254,0.097656,0.05249,0.10986,0.059814,0.0024414,0.0061035,0.048828,-0.042725,0.0036621,-0.0048828,-0.042725,-0.12451,-0.089111,-0.14648,-0.15259,-0.087891,-0.14526,-0.073242,-0.0085449,0.0024414,-0.046387,-0.092773,-0.026855,-0.064697,-0.021973,0.062256,0.032959,0.12451,0.18555,0.19287,0.14404,0.22217,0.18921,0.17456,0.21606,0.17944,0.25513,0.29541,0.30029,0.25391,0.28076,0.20874,0.16235,0.15381,0.19897,0.13916,0.18433,0.2124,0.18555,0.10254,0.14771,0.092773,0.06958,0.12695,0.091553,0.13794,0.19287,0.19409,0.14648,0.19775,0.14893,0.12451,0.15625,0.2356,0.2002,0.28198,0.30273,0.29663,0.23438,0.28076,0.20874,0.18921,0.19531,0.25757,0.20142,0.2478,0.2356,0.18921,0.12695,0.16724,0.13672,0.15625,0.20264,0.14648,0.21851,0.21973,0.177,0.097656,0.10986,0.029297,-0.031738,-0.036621,-0.0073242,-0.08667,-0.05249,-0.05249,-0.084229,-0.13672,-0.076904,-0.092773,-0.056152,0.019531,-0.0085449,0.091553,0.13428,0.12085,0.068359,0.10864,0.031738,-0.019531,0.0036621,0.01709,-0.070801,-0.028076,-0.045166,-0.1062,-0.177,-0.14282,-0.19775,-0.18188,-0.12329,-0.16479,-0.068359,-0.028076,-0.01709,-0.042725,0.041504,0.019531,0.025635,0.096436,0.20752,0.17578,0.25513,0.28687,0.23438,0.15503,0.17334,0.070801,0.025635,0.05127,-0.045166,0.0097656,0.037842,0.019531,-0.0354,0.042725,-0.012207,-0.031738,0.023193,0.085449,0.03418,0.11353,0.13428,0.10132,0.03418,0.092773,0.030518,0.019531,0.072021,0.013428,0.065918,0.097656,0.076904,0.021973,0.075684,0.020752,-0.018311,0.0073242,0.039063,-0.040283,-0.0024414,-0.0061035,-0.031738,-0.10376,-0.029297,-0.064697,-0.057373,0.031738,0.0012207,0.085449,0.15015,0.1709,0.11963,0.16846,0.10742,0.046387,0.040283,0.087891,0.021973,0.062256,0.080566,0.042725,-0.039063,0.032959,-0.028076,-0.041504,0.0097656,-0.045166,0.036621,0.065918,0.059814,0.010986,0.037842,-0.045166,-0.10376,-0.12695,-0.11597,-0.22095,-0.20264,-0.24292,-0.27954,-0.34912,-0.29419,-0.31982,-0.31372,-0.22949,-0.22461,-0.093994,0.025635,0.074463,0.072021,0.14526,0.097656,0.070801,0.093994,0.15381,0.11353,0.18921,0.21362,0.18555,0.11475,0.12329,0.05249,0.023193,0.029297,0.075684,0.0048828,0.031738,-0.0012207,-0.068359,-0.17212,-0.17212,-0.26001,-0.271,-0.23682,-0.28198,-0.20508,-0.13916,-0.11841,-0.12573,-0.047607,-0.062256,-0.063477,0.0073242,0.081787,0.05249,0.13062,0.15015,0.11597,0.18921,0.14282,0.097656,0.10376,0.14038,0.063477,0.12329,0.11597,0.070801,-0.0048828,-0.080566,-0.048828,-0.091553,-0.056152,-0.010986,-0.075684,-0.015869,0.0097656,-0.020752,-0.040283,0.05127,0.0048828,0.0354,0.10742,0.06958,0.14648,0.18311,0.17212,0.12207,0.17944,0.10986,0.062256,0.062256,0.076904,-0.0354,-0.023193,-0.072021,-0.14526,-0.23438,-0.18311,-0.23682,-0.21118,-0.11963,-0.14404,-0.056152,0.0073242,0.0036621,-0.064697,0.0012207,-0.061035,-0.081787,-0.040283,-0.065918,0.0073242,0.06958,0.10986,0.097656,0.036621,0.10742,0.06958,0.072021,0.13062,0.080566,0.14771,0.177,0.16357,0.085449,0.10376,0.014648,-0.050049,-0.068359,-0.058594,-0.15381,-0.15381,-0.19287,-0.28076,-0.3833,-0.34912,-0.37842,-0.35889,-0.25269,-0.21851,-0.062256,0.05249,0.10132,0.11475,0.20874,0.2002,0.21118,0.26733,0.3418,0.32715,0.39307,0.41016,0.39063,0.31616,0.36133,0.3064,0.28442,0.32227,0.40405,0.3772,0.43091,0.43945,0.39795,0.32593,0.36255,0.31616,0.32715,0.36865,0.31616,0.33569,0.31982,0.2417,0.14282,0.15869,0.091553,0.074463,0.097656,0.15991,0.11108,0.1709,0.18921,0.16968,0.12207,0.18311,0.14893,0.17944,0.22827,0.17944,0.23926,0.25391,0.22583,0.16357,0.19287,0.11841,0.097656,0.12329,0.16846,0.097656,0.13062,0.10498,0.023193,0.056152,-0.041504,-0.10986,-0.10376,-0.057373,-0.11475,-0.043945,-0.036621,-0.05127,-0.096436,-0.040283,-0.091553,-0.10986,-0.06958,-0.0061035,-0.081787,-0.031738,-0.053711,-0.12939,-0.20386,-0.16357,-0.21118,-0.18799,-0.13916,-0.20752,-0.14282,-0.12085,-0.15625,-0.23193,-0.19409,-0.24048,-0.2771,-0.24658,-0.18433,-0.2417,-0.18555,-0.177,-0.2356,-0.32471,-0.28931,-0.35767,-0.36133,-0.31616,-0.33813,-0.26855,-0.2478,-0.30762,-0.42847,-0.46265,-0.57251,-0.64575,-0.60547,-0.51514,-0.52979,-0.44312,-0.4126,-0.44312,-0.51025,-0.47485,-0.53589,-0.55054,-0.48706,-0.49316,-0.38818,-0.31616,-0.26611,-0.29541,-0.22705,-0.25391,-0.2832,-0.25146,-0.16602,-0.18799,-0.1001,-0.048828,-0.041504,-0.078125,0.0061035,-0.020752,0.015869,0.087891,0.059814,0.14648,0.19531,0.2124,0.19043,0.25879,0.23071,0.22461,0.271,0.36255,0.34058,0.41748,0.4541,0.45288,0.40649,0.45532,0.41626,0.41992,0.45776,0.42603,0.49194,0.51758,0.5127,0.48096,0.50049,0.43091,0.3833,0.38696,0.4248,0.36499,0.40283,0.38208,0.3418,0.25513,0.26611,0.18555,0.15625,0.17578,0.22217,0.17212,0.20752,0.2002,0.1709,0.11841,0.18188,0.16602,0.19653,0.25146,0.20752,0.27588,0.29541,0.27222,0.22461,0.271,0.22217,0.21851,0.28687,0.25146,0.32837,0.38696,0.38452,0.3418,0.28564,0.32837,0.28687,0.3125,0.36865,0.31128,0.39063,0.41016,0.36621,0.30884,0.36865,0.29663,0.27222,0.31006,0.36499,0.30273,0.35767,0.33569,0.28076,0.20874,0.25635,0.22461,0.25391,0.31372,0.2771,0.34546,0.37354,0.34912,0.30396,0.33936,0.28564,0.24658,0.25757,0.3125,0.22217,0.23926,0.21851,0.11841,0.0073242,0.023193,-0.083008,-0.11597,-0.093994,-0.18921,-0.15869,-0.14282,-0.20752,-0.32227,-0.32227,-0.42236,-0.50415,-0.49805,-0.47974,-0.54077,-0.48584,-0.45654,-0.4895,-0.52246,-0.42358,-0.41016,-0.3418,-0.23438,-0.21118,-0.075684,0.0097656,0.050049,0.032959,0.1123,0.083008,0.050049,0.062256,0.10864,0.015869,0.03418,0.014648,-0.037842,-0.085449,-0.0097656,-0.045166,-0.0354,0.042725,0.0024414,0.06958,0.096436,0.097656,0.031738,0.074463,0.019531,-0.013428,0.0073242,0.058594,0.018311,0.095215,0.14771,0.16602,0.13672,0.21851,0.2063,0.22827,0.29053,0.24658,0.30396,0.33569,0.31616,0.25269,0.2771,0.20874,0.15869,0.17212,0.22095,0.14648,0.17578,0.15137,0.083008,-0.026855,-0.019531,-0.12329,-0.16724,-0.15259,-0.21484,-0.14038,-0.08667,-0.046387,-0.045166,0.054932,0.021973,0.023193,0.06958,0.14404,0.10986,0.17944,0.18433,0.13794,0.065918,0.11353,0.05127,0.029297,0.058594,0.089111,0.021973,0.054932,0.054932,0.013428,0.075684,0.020752,-0.0048828,0.048828,0.10864,0.06958,0.15991,0.17822,0.16968,0.12695,0.17944,0.10864,0.10132,0.15381,0.21729,0.16846,0.20874,0.177,0.10742,0.12085,0.029297,-0.042725,-0.012207,0.048828,-0.019531,0.058594,0.067139,0.041504,-0.019531,0.030518,-0.05127,-0.084229,-0.048828,-0.10742,-0.03418,0.032959,0.039063,-0.0061035,-0.054932,0.026855,-0.013428,0.037842,0.11475,0.087891,0.16357,0.20508,0.17944,0.10132,0.13672,0.054932,-0.0085449,0.01709,0.036621,-0.059814,-0.05249,-0.089111,-0.19043,-0.28564,-0.26123,-0.31128,-0.26001,-0.18311,-0.19653,-0.085449,-0.042725,-0.041504,-0.087891,-0.05127,-0.11963,-0.16602,-0.16357,-0.21729,-0.15747,-0.10864,-0.092773,-0.12939,-0.18311,-0.10132,-0.12451,-0.097656,-0.01709,-0.0354,0.048828,0.083008,0.095215,0.05249,0.10498,0.061035,0.036621,0.048828,0.10864,0.039063,0.068359,0.046387,0,-0.097656,-0.054932,-0.10132,-0.10132,-0.032959,-0.064697,0.024414,0.073242,0.095215,0.063477,0.14648,0.10986,0.091553,0.12695,0.19531,0.14038,0.19043,0.19897,0.15869,0.097656,0.15259,0.11963,0.1416,0.2063,0.16846,0.25513,0.28442,0.26367,0.19897,0.23438,0.13184,0.059814,0.050049,0.05249,-0.028076,0.031738,0.013428,-0.029297,-0.10498,-0.058594,-0.11475,-0.11108,-0.059814,-0.1123,-0.031738,0.010986,0,-0.029297,0.040283,0.0012207,-0.0073242,0.058594,0.12085,0.085449,0.17578,0.19043,0.16602,0.097656,0.14038,0.070801,0.06958,0.12573,0.17822,0.12939,0.18066,0.15869,0.1062,0.14893,0.081787,0.031738,0.076904,0.15991,0.13306,0.21729,0.26367,0.23804,0.18799,0.25024,0.19653,0.18433,0.21729,0.26367,0.19775,0.22461,0.20874,0.15381,0.19775,0.12939,0.080566,0.087891,0.14648,0.070801,0.12329,0.14893,0.10254,0.014648,0.064697,-0.0097656,-0.042725,0.0097656,0.067139,0.021973,0.090332,0.11841,0.089111,0.15503,0.11108,0.072021,0.078125,0.13184,0.075684,0.14404,0.15991,0.1355,0.061035,0.1001,0.025635,-0.013428,0.018311,0.059814,0.0097656,0.070801,0.06958,0.025635,0.072021,0.0097656,-0.028076,-0.020752,0.010986,-0.078125,-0.056152,-0.072021,-0.11841,-0.22217,-0.19531,-0.22827,-0.23438,-0.17944,-0.067139,-0.078125,0.021973,0.062256,0.058594,0.019531,0.083008,0.036621,0.026855,0.047607,-0.039063,-0.0085449,-0.01709,-0.053711,-0.11475,-0.074463,-0.13672,-0.177,-0.15259,-0.085449,-0.12939,-0.062256,-0.05249,-0.091553,-0.13916,-0.065918,-0.068359,-0.0024414,0.080566,0.062256,0.17822,0.21484,0.22339,0.18555,0.25513,0.22705,0.22705,0.26978,0.34912,0.3186,0.37598,0.39063,0.35278,0.2771,0.3064,0.23682,0.20752,0.23193,0.1416,0.17578,0.16846,0.11597,0.05249,0.080566,0.0036621,-0.045166,-0.024414,0.01709,-0.073242,-0.018311,-0.032959,-0.085449,-0.14648,-0.10742,-0.15869,-0.13062,-0.05127,-0.075684,0.040283,0.13062,0.18066,0.18799,0.2832,0.23804,0.21851,0.25757,0.30273,0.25024,0.3125,0.31738,0.2832,0.21606,0.26001,0.20142,0.19043,0.2478,0.18921,0.25269,0.31372,0.3064,0.23682,0.28687,0.21729,0.16724,0.18799,0.27466,0.21729,0.28076,0.3125,0.26367,0.18799,0.22827,0.16113,0.14771,0.20752,0.15259,0.2356,0.2832,0.28687,0.2478,0.19165,0.24658,0.20752,0.22705,0.28442,0.2356,0.29663,0.31128,0.271,0.18921,0.22095,0.15381,0.10986,0.13184,0.19775,0.13062,0.16357,0.15869,0.087891,0.11108,0.048828,-0.010986,0.0024414,0.032959,-0.021973,0.05249,0.079346,0.079346,0.030518,0.095215,0.074463,0.065918,0.11841,0.20508,0.16479,0.22583,0.22461,0.17578,0.21729,0.15381,0.092773,0.083008,0.12451,0.040283,0.070801,0.081787,0.037842,-0.045166,-0.01709,-0.090332,-0.13916,-0.11475,-0.06958,-0.13306,-0.096436,-0.10376,-0.1355,-0.18188,-0.12085,-0.15015,-0.12451,-0.078125,-0.11841,-0.031738,-0.019531,-0.029297,-0.084229,-0.048828,-0.1062,-0.12085,-0.084229,-0.0073242,-0.061035,0.0097656,0.026855,0.0024414,-0.041504,0.0354,-0.0024414,0.032959,0.091553,0.048828,0.11841,0.15625,0.13672,0.075684,0.12329,0.061035,0.031738,0.075684,0.16602,0.13184,0.2124,0.21851,0.17822,0.10376,0.14038,0.085449,0.10132,0.16968,0.12695,0.22217,0.2832,0.28442,0.22705,0.2832,0.22461,0.17334,0.20508,0.26978,0.19897,0.25146,0.26611,0.20874,0.13306,0.17944,0.10376,0.079346,0.13672,0.062256,0.12695,0.15625,0.13916,0.062256,0.095215,0.0036621,-0.063477,-0.047607,-0.01709,-0.097656,-0.061035,-0.062256,-0.10742,-0.17334,-0.12939,-0.17578,-0.17212,-0.10742,-0.15747,-0.093994,-0.057373,-0.064697,-0.11597,-0.062256,-0.1062,-0.11475,-0.075684,0.013428,-0.015869,0.070801,0.081787,0.064697,-0.026855,0.020752,-0.03418,-0.063477,-0.025635,-0.067139,-0.021973,0.01709,-0.0085449,-0.057373,-0.021973,-0.072021,-0.10254,-0.064697,0,-0.056152,0.0354,0.068359,0.056152,0.0012207,0.06958,0.020752,0.020752,0.078125,0.15869,0.12085,0.17578,0.18799,0.16602,0.22217,0.16479,0.14771,0.18066,0.25146,0.23438,0.3125,0.32959,0.30273,0.24658,0.26001,0.16846,0.12329,0.13062,0.17456,0.13794,0.18555,0.17944,0.12451,0.15991,0.079346,0.010986,0.0048828,0.023193,-0.041504,0.036621,0.042725,0.0036621,-0.062256,-0.046387,-0.13794,-0.18188,-0.18799,-0.15991,-0.22705,-0.18921,-0.21851,-0.26367,-0.33081,-0.30884,-0.36743,-0.35278,-0.30884,-0.35767,-0.28076,-0.25513,-0.26367,-0.29785,-0.24292,-0.32227,-0.35889,-0.32227,-0.28687,-0.35522,-0.31372,-0.3479,-0.42236,-0.51392,-0.5249,-0.625,-0.6543,-0.62988,-0.67017,-0.57861,-0.49561,-0.44189,-0.4248,-0.30029,-0.26733,-0.21729,-0.095215,-0.075684,0.048828,0.15747,0.2124,0.17944,0.12817,0.18799,0.11597,0.10986,0.15015,0.17578,0.11963,0.14893,0.11963,0.059814,0.13916,0.1001,0.095215,0.15259,0.24414,0.19775,0.25635,0.28687,0.23804,0.14893,0.1709,0.097656,0.068359,0.10376,0.031738,0.080566,0.087891,0.05249,-0.036621,-0.0036621,-0.072021,-0.12695,-0.10864,-0.032959,-0.085449,-0.015869,0.043945,0.039063,-0.0024414,0.10742,0.054932,0.050049,0.12085,0.080566,0.15137,0.20508,0.19775,0.15137,0.19897,0.14893,0.11597,0.13794,0.20508,0.16846,0.24292,0.27588,0.25269,0.18921,0.22705,0.17822,0.15259,0.16968,0.2063,0.13794,0.14404,0.12451,0.068359,0.090332,0.0073242,-0.043945,-0.037842,0.0048828,-0.070801,-0.026855,-0.01709,-0.054932,-0.12817,-0.1001,-0.17212,-0.2124,-0.18799,-0.24414,-0.18311,-0.16113,-0.18066,-0.25269,-0.3479,-0.33936,-0.41504,-0.46387,-0.46631,-0.52734,-0.47363,-0.48706,-0.50903,-0.5481,-0.46753,-0.44556,-0.38696,-0.29053,-0.18555,-0.17212,-0.073242,-0.048828,-0.059814,-0.084229,0.0097656,0.0012207,0.050049,0.12939,0.065918,0.11597,0.11597,0.050049,-0.056152,-0.072021,-0.19897,-0.28564,-0.30151,-0.26978,-0.28564,-0.17334,-0.11353,-0.081787,-0.087891,0.0012207,-0.031738,0.0097656,0.057373,-0.0061035,0.054932,0.061035,0.0036621,-0.072021,-0.12695,-0.070801,-0.1062,-0.057373,-0.074463,0.019531,0.097656,0.12451,0.079346,0.13794,0.075684,0.0036621,-0.021973,0.013428,-0.074463,-0.010986,-0.0036621,-0.043945,-0.1355,-0.11353,-0.19775,-0.24048,-0.19775,-0.10376,-0.11475,0.01709,0.1001,0.13916,0.15991,0.27954,0.28931,0.34912,0.45898,0.45288,0.54321,0.61157,0.625,0.57617,0.62256,0.55664,0.50415,0.51025,0.56396,0.48828,0.52368,0.52734,0.49561,0.42236,0.47852,0.42847,0.41992,0.47119,0.42358,0.46997,0.50171,0.47119,0.40161,0.43701,0.36377,0.31006,0.32227,0.36377,0.29663,0.3418,0.33813,0.28687,0.19653,0.22827,0.15625,0.12817,0.16113,0.097656,0.15747,0.177,0.15625,0.080566,0.098877,0.015869,-0.045166,-0.048828,-0.0097656,-0.076904,-0.040283,-0.021973,-0.040283,-0.089111,-0.040283,-0.083008,-0.078125,-0.012207,-0.048828,0.032959,0.074463,0.084229,0.048828,0.12451,0.080566,0.064697,0.11597,0.19897,0.15991,0.25757,0.27588,0.2478,0.18066,0.21851,0.12085,0.062256,0.029297,0.0061035,-0.12451,-0.13428,-0.19409,-0.26855,-0.23193,-0.2832,-0.31006,-0.26978,-0.18799,-0.20874,-0.093994,-0.01709,-0.0097656,-0.040283,0.028076,-0.023193,-0.021973,0.054932,0.10742,0.043945,0.12329,0.13916,0.10376,0.062256,0.12817,0.089111,0.12817,0.20752,0.16357,0.23438,0.27954,0.25391,0.20264,0.25024,0.17334,0.13184,0.15381,0.20874,0.14282,0.19653,0.19409,0.14404,0.068359,0.14404,0.092773,0.1123,0.17822,0.13794,0.22217,0.26367,0.24414,0.16846,0.20752,0.13428,0.080566,0.10986,0.18921,0.12695,0.19531,0.21973,0.18311,0.23438,0.18188,0.13672,0.14771,0.2063,0.15991,0.2063,0.22217,0.21729,0.15747,0.2124,0.16968,0.14282,0.18555,0.2832,0.23315,0.3064,0.33203,0.29175,0.22461,0.26001,0.20142,0.19897,0.26367,0.2356,0.30762,0.354,0.3418,0.27954,0.32593,0.26611,0.22583,0.23926,0.29663,0.23071,0.271,0.26855,0.22705,0.13306,0.16846,0.13062,0.1416,0.19287,0.15381,0.24658,0.271,0.26123,0.21118,0.23804,0.15869,0.10864,0.11108,0.14771,0.097656,0.13062,0.12817,0.091553,0.0061035,0.045166,-0.0073242,-0.020752,0.026855,-0.031738,0.048828,0.083008,0.081787,0.046387,0.10376,0.040283,-0.0061035,0.021973,0.050049,-0.0061035,0.084229,0.074463,0.050049,-0.0097656,0.058594,-0.0097656,0.013428,0.046387,0.1001,0.054932,0.10254,0.080566,0.037842,0.090332,0.01709,-0.010986,0.040283,0.080566,0.0354,0.10742,0.12329,0.10132,0.032959,0.057373,-0.021973,-0.062256,-0.074463,-0.080566,-0.2002,-0.23071,-0.30273,-0.38574,-0.47485,-0.44922,-0.48462,-0.41748,-0.31494,-0.31128,-0.18433,-0.1001,-0.087891,-0.11108,-0.048828,-0.11475,-0.14648,-0.11597,-0.18799,-0.13916,-0.074463,-0.036621,-0.057373,-0.080566,0.014648,-0.020752,0.025635,0.081787,0.05127,0.1355,0.17212,0.14648,0.079346,0.13062,0.061035,0.015869,0.050049,0.10498,0.030518,0.10254,0.12085,0.081787,0.028076,0.084229,0.030518,0.050049,0.11841,0.057373,0.11108,0.16235,0.16235,0.12451,0.19165,0.16357,0.14038,0.18921,0.28076,0.22705,0.28442,0.28442,0.22339,0.12451,0.15747,0.084229,0.064697,0.10254,0.048828,0.11353,0.16113,0.14893,0.083008,0.14648,0.11841,0.093994,0.12207,0.18555,0.11597,0.15259,0.16846,0.10742,0.042725,0.096436,0.05127,0.06958,0.13428,0.10742,0.19897,0.24536,0.26733,0.22949,0.2771,0.22705,0.19775,0.21606,0.28564,0.22095,0.27344,0.28442,0.25757,0.18799,0.20996,0.13794,0.11108,0.14771,0.068359,0.11108,0.12695,0.10498,0.0354,0.087891,0.05127,0.032959,0.074463,0.16602,0.12329,0.21118,0.23071,0.19531,0.12939,0.177,0.11475,0.10986,0.14893,0.20874,0.15747,0.19409,0.18921,0.15381,0.19653,0.13672,0.095215,0.13916,0.19409,0.1355,0.2002,0.20752,0.18066,0.12085,0.16357,0.080566,0.056152,0.090332,0.13062,0.068359,0.089111,0.050049,-0.020752,-0.093994,-0.063477,-0.1355,-0.1355,-0.1001,-0.18066,-0.14526,-0.14648,-0.20508,-0.29419,-0.26367,-0.34302,-0.36743,-0.33813,-0.39673,-0.33569,-0.29541,-0.29297,-0.33081,-0.26367,-0.28564,-0.29785,-0.24414,-0.14771,-0.19165,-0.11353,-0.081787,-0.11353,-0.21118,-0.19409,-0.29907,-0.3772,-0.39307,-0.49438,-0.47852,-0.42847,-0.40039,-0.41992,-0.3479,-0.36865,-0.3833,-0.34424,-0.27222,-0.31494,-0.25146,-0.22095,-0.23682,-0.29297,-0.23804,-0.29663,-0.35645,-0.35889,-0.32104,-0.37842,-0.32593,-0.28442,-0.28442,-0.30151,-0.19165,-0.17822,-0.11597,-0.0024414,0.0085449,0.091553,0.15869,0.16602,0.11108,0.15625,0.10132,0.054932,0.059814,0.10498,0.032959,0.075684,0.083008,0.068359,0.023193,0.10498,0.078125,0.10376,0.16846,0.14038,0.23193,0.25391,0.25757,0.20752,0.2478,0.17456,0.13672,0.14771,0.21851,0.17578,0.23804,0.26001,0.24414,0.20264,0.26367,0.22461,0.2356,0.27954,0.22461,0.2771,0.26733,0.21851,0.13916,0.15137,0.065918,0.01709,0.021973,0.039063,-0.026855,0.026855,0.0036621,-0.031738,-0.076904,-0.021973,-0.059814,-0.026855,0.053711,0.12695,0.10986,0.17456,0.17212,0.12085,0.15137,0.074463,0.015869,0.010986,0.029297,-0.05249,-0.0036621,-0.0024414,-0.025635,-0.075684,-0.0024414,-0.032959,-0.0073242,0.06958,0.14404,0.12817,0.17944,0.16968,0.12939,0.18311,0.091553,0.019531,0.0061035,0.0024414,-0.11108,-0.085449,-0.085449,-0.14038,-0.20508,-0.13306,-0.15503,-0.12085,-0.015869,-0.024414,0.074463,0.13062,0.1355,0.068359,0.10864,0.042725,-0.025635,-0.014648,0.013428,-0.068359,0.0073242,0.024414,0.015869,-0.031738,0.062256,0.024414,0.036621,0.12939,0.10986,0.19775,0.28442,0.29663,0.25635,0.2002,0.24536,0.19409,0.2063,0.25879,0.18921,0.22827,0.24414,0.21118,0.13916,0.18555,0.10864,0.061035,0.068359,0.11475,0.041504,0.062256,0.0354,-0.046387,-0.1355,-0.10376,-0.16357,-0.15991,-0.093994,-0.13672,-0.056152,0,-0.023193,-0.062256,-0.01709,-0.075684,-0.1062,-0.075684,-0.015869,-0.064697,-0.025635,-0.0048828,-0.026855,-0.058594,0.050049,0.054932,0.11475,0.20874,0.20508,0.29541,0.32837,0.3418,0.29297,0.33813,0.27832,0.25391,0.271,0.33447,0.26733,0.30273,0.29541,0.22461,0.13428,0.16357,0.092773,0.10132,0.15625,0.11597,0.18677,0.21729,0.20752,0.16724,0.2356,0.2063,0.18555,0.22949,0.3064,0.27222,0.34424,0.34302,0.27832,0.18188,0.18677,0.098877,0.078125,0.095215,0.031738,0.10986,0.16602,0.15625,0.13184,0.20508,0.16968,0.16968,0.22583,0.29663,0.25146,0.3186,0.3125,0.26367,0.17578,0.2002,0.12817,0.11841,0.17212,0.13672,0.22339,0.26489,0.26367,0.23438,0.19531,0.25635,0.22949,0.25635,0.31738,0.2771,0.33569,0.3418,0.28442,0.19653,0.22461,0.12085,0.068359,0.070801,-0.062256,-0.042725,-0.056152,-0.098877,-0.15991,-0.22949,-0.15625,-0.19409,-0.16479,-0.091553,-0.12329,-0.040283,0.0024414,-0.012207,-0.057373,0.023193,-0.03418,-0.023193,0.043945,0.093994,0.041504,0.089111,0.068359,-0.0012207,-0.091553,-0.061035,-0.12085,-0.10376,-0.048828,-0.098877,-0.041504,-0.025635,-0.054932,-0.11475,-0.059814,-0.13306,-0.15381,-0.10376,-0.14038,-0.054932,-0.0012207,0.021973,-0.015869,-0.045166,0.025635,-0.018311,0,0.05127,0.0036621,0.042725,0.065918,0.031738,-0.05249,-0.0036621,-0.074463,-0.10864,-0.073242,-0.12329,-0.048828,0.0024414,0.0085449,-0.013428,-0.05249,0.013428,-0.01709,0.020752,0.070801,0.020752,0.092773,0.11841,0.10986,0.05249,0.10132,0.050049,0.0097656,0.0354,0.095215,0.036621,0.087891,0.084229,0.029297,0.065918,-0.019531,-0.084229,-0.085449,-0.045166,-0.098877,-0.039063,-0.015869,-0.040283,-0.08667,-0.028076,-0.057373,-0.064697,-0.021973,0.059814,0.013428,0.076904,0.062256,0.018311,-0.063477,-0.046387,-0.12939,-0.13428,-0.10254,-0.14038,-0.058594,0.0085449,0.013428,0.0024414,0.10498,0.1001,0.11475,0.177,0.26733,0.23071,0.3125,0.32227,0.2832,0.20508,0.22705,0.15625,0.13672,0.15015,0.061035,0.12451,0.12939,0.11963,0.067139,0.12939,0.068359,0.032959,0.068359,0.1355,0.081787,0.177,0.21851,0.19531,0.15625,0.21851,0.16968,0.17334,0.2417,0.18921,0.25269,0.27344,0.2478,0.16357,0.20874,0.14282,0.096436,0.10376,0.13062,0.062256,0.12207,0.13672,0.1001,0.037842,0.091553,0.041504,0.048828,0.11841,0.18311,0.12695,0.17822,0.15869,0.072021,0.10132,0.010986,-0.061035,-0.05127,-0.0085449,-0.072021,-0.032959,-0.019531,-0.042725,-0.10132,-0.056152,-0.1001,-0.11475,-0.075684,-0.13428,-0.084229,-0.070801,-0.12817,-0.23193,-0.24292,-0.3418,-0.40894,-0.42236,-0.41748,-0.49805,-0.46997,-0.47241,-0.52368,-0.60181,-0.53345,-0.54077,-0.52856,-0.46509,-0.48584,-0.41748,-0.37109,-0.36743,-0.40649,-0.36987,-0.41748,-0.43091,-0.39063,-0.31616,-0.34058,-0.26123,-0.22705,-0.21851,-0.25024,-0.16602,-0.18066,-0.15259,-0.070801,-0.064697,0.043945,0.1355,0.16846,0.15015,0.11719,0.18677,0.15381,0.19653,0.25146,0.19287,0.24414,0.23315,0.16479,0.070801,0.083008,0.0097656,-0.026855,0.0073242,0.059814,0.020752,0.091553,0.10986,0.096436,0.072021,0.16357,0.15869,0.21484,0.28442,0.25391,0.32227,0.34546,0.30396,0.24414,0.27222,0.18921,0.15381,0.18066,0.22461,0.16235,0.23315,0.2417,0.21484,0.17212,0.22217,0.16357,0.16357,0.19409,0.11841,0.16968,0.16602,0.13672,0.085449,0.16357,0.13672,0.13062,0.18921,0.28564,0.25146,0.32593,0.34302,0.29541,0.23315,0.27832,0.21851,0.21973,0.271,0.19897,0.25391,0.2832,0.25757,0.19165,0.25391,0.2063,0.1709,0.22583,0.29785,0.24414,0.31494,0.31982,0.2771,0.20142,0.24048,0.17456,0.16724,0.23193,0.18677,0.25024,0.29175,0.26733,0.20508,0.23315,0.15991,0.091553,0.081787,0.1001,0.0097656,0.0354,0.032959,-0.0048828,-0.046387,0.031738,0.0024414,0.015869,0.096436,0.074463,0.16479,0.22095,0.23682,0.19043,0.2417,0.19165,0.15381,0.16724,0.22949,0.18066,0.24414,0.28442,0.26123,0.18799,0.24536,0.21606,0.20264,0.26123,0.20996,0.2771,0.32715,0.31494,0.26855,0.30396,0.22339,0.1709,0.17578,0.20142,0.12939,0.16479,0.14648,0.089111,-0.0061035,0.026855,-0.061035,-0.080566,-0.068359,-0.13428,-0.087891,-0.054932,-0.075684,-0.13062,-0.10254,-0.16602,-0.21484,-0.21118,-0.16479,-0.20142,-0.12695,-0.12451,-0.14282,-0.23071,-0.19653,-0.26367,-0.27832,-0.2417,-0.17822,-0.22339,-0.16357,-0.1416,-0.16846,-0.20142,-0.12329,-0.13062,-0.068359,0.010986,-0.048828,0.037842,0.057373,0.024414,-0.058594,-0.023193,-0.11963,-0.18066,-0.17578,-0.24414,-0.18311,-0.12207,-0.076904,-0.072021,-0.074463,0.013428,-0.0061035,0.057373,0.14038,0.12085,0.23682,0.27954,0.2832,0.24902,0.31372,0.26367,0.23804,0.27466,0.32715,0.2478,0.29663,0.29053,0.20996,0.12573,0.14893,0.064697,0.056152,0.089111,0.020752,0.068359,0.10376,0.091553,0.03418,0.087891,0.046387,0.018311,0.05249,0.12085,0.065918,0.13428,0.13428,0.08667,0.020752,0.068359,0.0085449,0.015869,0.070801,0.021973,0.081787,0.12695,0.12329,0.089111,0.15869,0.11353,0.084229,0.11719,0.18921,0.13428,0.19165,0.21729,0.18311,0.1001,0.1355,0.06958,0.042725,0.074463,0.013428,0.068359,0.068359,0.058594,-0.0061035,0.042725,-0.019531,-0.057373,-0.046387,0.01709,-0.050049,0.029297,0.053711,0.032959,-0.021973,0.06958,0.019531,0.036621,0.11475,0.068359,0.13672,0.16968,0.15381,0.098877,0.15381,0.10742,0.081787,0.11353,0.19165,0.15625,0.22461,0.22705,0.19409,0.13062,0.16968,0.10864,0.097656,0.11353,0.16479,0.11475,0.12329,0.097656,0.030518,0.045166,-0.042725,-0.12695,-0.16357,-0.15991,-0.26123,-0.25024,-0.27588,-0.33447,-0.39917,-0.33447,-0.36133,-0.33447,-0.24902,-0.14893,-0.14893,-0.072021,-0.048828,-0.059814,-0.10864,-0.047607,-0.063477,-0.019531,0.048828,-0.0024414,0.079346,0.087891,0.046387,-0.024414,0.0073242,-0.087891,-0.10986,-0.080566,-0.15015,-0.085449,-0.028076,-0.021973,-0.045166,-0.078125,-0.0036621,-0.029297,0.01709,0.080566,0.032959,0.13306,0.16602,0.15625,0.11597,0.17944,0.12939,0.10742,0.12817,0.17212,0.098877,0.1355,0.095215,0.0012207,-0.084229,-0.048828,-0.12085,-0.10498,-0.070801,-0.13062,-0.048828,0.012207,0.012207,-0.0036621,0.10254,0.068359,0.070801,0.14038,0.21484,0.15015,0.21362,0.22461,0.16235,0.097656,0.15259,0.095215,0.10864,0.17944,0.13306,0.18799,0.21362,0.19653,0.14404,0.20264,0.15015,0.12695,0.16968,0.2478,0.20752,0.27588,0.29297,0.2478,0.19897,0.25879,0.20264,0.19531,0.2478,0.19653,0.25635,0.27222,0.24658,0.1709,0.21484,0.15869,0.10986,0.12939,0.20508,0.15015,0.20142,0.21362,0.15991,0.06958,0.10742,0.043945,0.023193,0.059814,0.0061035,0.070801,0.11597,0.12085,0.083008,0.15015,0.1123,0.10132,0.14404,0.22095,0.17944,0.26245,0.27222,0.25635,0.17578,0.22217,0.17212,0.15259,0.18799,0.12451,0.1709,0.20508,0.19897,0.14771,0.18555,0.12207,0.065918,0.062256,0.091553,0.013428,0.058594,0.068359,0.042725,-0.029297,0.032959,-0.01709,-0.012207,0.041504,0.096436,0.074463,0.11108,0.10132,0.045166,0.063477,-0.040283,-0.098877,-0.12085,-0.11108,-0.18311,-0.15503,-0.15259,-0.19775,-0.26367,-0.20508,-0.23071,-0.21606,-0.14648,-0.17578,-0.098877,-0.054932,-0.059814,-0.10376,-0.18433,-0.14893,-0.2124,-0.21973,-0.22217,-0.31616,-0.30029,-0.33325,-0.39673,-0.47241,-0.4541,-0.50903,-0.50171,-0.43213,-0.34912,-0.36865,-0.29297,-0.26733,-0.26367,-0.27588,-0.17578,-0.18555,-0.15747,-0.098877,-0.15991,-0.11841,-0.10132,-0.14893,-0.23438,-0.2002,-0.26367,-0.28198,-0.23926,-0.16602,-0.19165,-0.096436,-0.045166,-0.040283,-0.065918,-0.0048828,-0.0354,-0.0024414,0.064697,0.0036621,0.070801,0.10864,0.081787,0.013428,0.050049,-0.019531,-0.03418,0.032959,0.0024414,0.072021,0.12939,0.14648,0.11108,0.048828,0.098877,0.031738,0.0036621,0.0354,-0.030518,0.023193,0.015869,-0.0012207,-0.046387,-0.1062,-0.023193,-0.045166,-0.0097656,-0.054932,0.015869,0.050049,0.054932,-0.0061035,-0.076904,-0.029297,-0.091553,-0.08667,-0.025635,-0.058594,0.029297,0.079346,0.087891,0.029297,0.065918,-0.0048828,-0.019531,-0.0073242,-0.042725,0.021973,0.074463,0.085449,0.061035,-0.015869,0.025635,-0.059814,-0.068359,-0.053711,-0.13794,-0.10498,-0.091553,-0.11353,-0.15869,-0.090332,-0.10376,-0.090332,-0.019531,-0.028076,0.079346,0.14648,0.16968,0.15137,0.090332,0.14648,0.090332,0.06958,0.10254,0.13306,0.074463,0.081787,0.06958,0.041504,0.12451,0.10986,0.1355,0.2063,0.29785,0.29541,0.37231,0.40039,0.36499,0.27588,0.29907,0.22827,0.19287,0.21362,0.11597,0.14038,0.15137,0.11597,0.056152,0.097656,0.037842,0.012207,0.058594,0.12451,0.080566,0.16602,0.18677,0.17334,0.12085,0.15991,0.081787,0.032959,0.05249,0.092773,0.019531,0.057373,0.032959,-0.026855,-0.084229,-0.025635,-0.062256,-0.020752,0.054932,0.023193,0.10742,0.15015,0.12939,0.078125,0.15991,0.11353,0.10742,0.15625,0.21851,0.17212,0.21973,0.21362,0.14282,0.17944,0.10132,0.039063,0.05127,0.1001,0.029297,0.095215,0.1355,0.12695,0.056152,0.087891,0.013428,-0.025635,0.013428,0.064697,0.028076,0.098877,0.13428,0.10742,0.17944,0.13428,0.1001,0.12329,0.2002,0.16602,0.24048,0.26611,0.25635,0.19409,0.25146,0.19165,0.14893,0.17944,0.25879,0.20752,0.271,0.2832,0.23804,0.28564,0.21484,0.16602,0.15381,0.19043,0.14648,0.21729,0.25757,0.271,0.22827,0.29541,0.25879,0.24414,0.27954,0.33691,0.28076,0.34302,0.34668,0.31006,0.22705,0.25391,0.19165,0.17578,0.20874,0.15869,0.20752,0.23804,0.2478,0.19287,0.23438,0.17334,0.11963,0.13794,0.16968,0.096436,0.11963,0.087891,0.023193,-0.070801,-0.037842,-0.092773,-0.090332,-0.05127,-0.087891,0.014648,0.045166,0.05249,0.010986,0.050049,0,-0.046387,-0.018311,0.025635,-0.045166,0.031738,0.039063,0.0024414,-0.05249,0.010986,-0.036621,-0.012207,0.058594,0.0097656,0.083008,0.10498,0.075684,0.010986,0.046387,-0.031738,-0.059814,-0.013428,0.05127,-0.0073242,0.08667,0.11108,0.090332,0.036621,0.079346,0.010986,0.010986,0.06958,0.12085,0.068359,0.10132,0.081787,0.031738,0.08667,0.025635,-0.021973,0.013428,0.05249,-0.015869,0.040283,0.021973,-0.046387,-0.13672,-0.12329,-0.24292,-0.33081,-0.37231,-0.39063,-0.49194,-0.49194,-0.53467,-0.61523,-0.57617,-0.61523,-0.6311,-0.58105,-0.49072,-0.48462,-0.33691,-0.19043,-0.13062,-0.1123,0.018311,0.023193,0.054932,0.14893,0.14648,0.22949,0.29297,0.3125,0.26978,0.20996,0.26611,0.20508,0.20996,0.26245,0.20996,0.27466,0.27588,0.2478,0.16113,0.18799,0.10986,0.05249,0.058594,0.10742,0.024414,0.048828,0.03418,-0.042725,-0.11841,-0.057373,-0.11597,-0.10132,-0.026855,-0.067139,0.018311,0.048828,0.037842,-0.024414,0.037842,-0.020752,-0.031738,-0.0012207,-0.045166,0.029297,0.093994,0.13306,0.12817,0.079346,0.12939,0.090332,0.095215,0.15015,0.10376,0.15991,0.19409,0.18799,0.12817,0.16846,0.12085,0.091553,0.13672,0.22461,0.19775,0.26489,0.29297,0.26855,0.21973,0.28931,0.25391,0.27832,0.35645,0.33936,0.43457,0.46143,0.44434,0.39307,0.41016,0.30762,0.24658,0.22461,0.24414,0.17944,0.23193,0.24292,0.21851,0.15015,0.20142,0.16968,0.18921,0.2478,0.20874,0.26733,0.30029,0.2771,0.20508,0.22461,0.12939,0.068359,0.070801,0.10376,0.026855,0.061035,0.037842,-0.020752,-0.10498,-0.093994,-0.18188,-0.20996,-0.18066,-0.25146,-0.19653,-0.17456,-0.19897,-0.25269,-0.19531,-0.23193,-0.21973,-0.12939,-0.0024414,0.01709,0.11719,0.18188,0.17334,0.12329,0.18677,0.11841,0.091553,0.12817,0.15625,0.078125,0.081787,0.042725,-0.043945,-0.13306,-0.10864,-0.15503,-0.13306,-0.048828,-0.074463,0,0.037842,0.0354,-0.013428,0.063477,0.012207,0.0061035,0.062256,0.036621,0.1123,0.15869,0.17578,0.13062,0.18311,0.14893,0.12451,0.15991,0.23804,0.19897,0.26489,0.29053,0.2771,0.21729,0.26367,0.21729,0.18677,0.22217,0.19043,0.25513,0.3125,0.31616,0.26123,0.3064,0.2478,0.19897,0.21362,0.24902,0.19287,0.22949,0.21484,0.16846,0.063477,0.056152,-0.01709,-0.062256,-0.039063,-0.085449,-0.018311,0.041504,0.045166,0.019531,0.078125,0.019531,-0.012207,0.019531,0.070801,0.047607,0.11597,0.13916,0.11597,0.032959,0.0354,-0.05249,-0.10376,-0.10376,-0.16846,-0.11841,-0.090332,-0.10376,-0.14893,-0.21118,-0.16846,-0.21484,-0.18311,-0.12207,-0.14893,-0.054932,-0.021973,-0.0097656,-0.041504,0.026855,-0.0097656,0.0012207,0.037842,0.12329,0.083008,0.15747,0.16846,0.12451,0.062256,0.10132,0.050049,0.070801,0.12695,0.084229,0.17334,0.2063,0.20142,0.17212,0.23804,0.17456,0.14648,0.18188,0.24048,0.18677,0.25146,0.24658,0.19897,0.12939,0.16357,0.075684,0.067139,0.11597,0.16968,0.12695,0.16968,0.14038,0.078125,0.12085,0.048828,0.012207,0.046387,0.11475,0.06958,0.13794,0.15869,0.12451,0.046387,0.1001,0.031738,0.015869,0.083008,0.14038,0.10498,0.16357,0.15747,0.10498,0.15991,0.097656,0.054932,0.081787,0.14282,0.079346,0.11841,0.14526,0.089111,0,0.05249,-0.01709,-0.0354,0.032959,0.078125,0.023193,0.062256,0.05127,0.0012207,0.029297,-0.03418,-0.081787,-0.070801,-0.014648,-0.061035,0.0012207,0.039063,0.015869,-0.036621,0.036621,-0.026855,-0.0354,0.012207,0.054932,0.0024414,0.042725,0.05127,0.023193,0.074463,0.05249,0.041504,0.075684,0.15381,0.12939,0.20142,0.2478,0.23804,0.15869,0.19531,0.15381,0.12817,0.14648,0.20996,0.15747,0.19043,0.18555,0.14648,0.17578,0.10742,0.057373,0.065918,0.12085,0.074463,0.12939,0.16602,0.14893,0.097656,0.15625,0.093994,0.087891,0.12695,0.20874,0.16846,0.21118,0.20508,0.16968,0.097656,0.13672,0.083008,0.087891,0.11597,0.068359,0.14648,0.15747,0.14893,0.10742,0.16357,0.11108,0.10742,0.14282,0.21362,0.17578,0.24048,0.24048,0.18677,0.12207,0.15625,0.092773,0.095215,0.12695,0.042725,0.10132,0.10864,0.058594,-0.028076,-0.0024414,-0.1001,-0.15747,-0.13672,-0.096436,-0.15869,-0.098877,-0.098877,-0.14038,-0.19409,-0.17456,-0.22705,-0.20508,-0.12085,-0.13916,-0.048828,0.0024414,-0.028076,-0.064697,-0.11597,-0.062256,-0.097656,-0.059814,-0.0073242,-0.057373,-0.0097656,-0.012207,-0.073242,-0.15259,-0.12451,-0.20142,-0.2417,-0.22095,-0.29907,-0.26733,-0.26489,-0.32227,-0.42358,-0.43213,-0.53101,-0.59692,-0.59326,-0.5249,-0.54199,-0.4541,-0.38818,-0.38086,-0.40649,-0.32227,-0.34668,-0.33203,-0.23926,-0.271,-0.19165,-0.12695,-0.10498,-0.12329,-0.15381,-0.084229,-0.12207,-0.10254,-0.05249,-0.11108,-0.062256,-0.056152,-0.087891,-0.16724,-0.13306,-0.18555,-0.20874,-0.16479,-0.2002,-0.1416,-0.087891,-0.081787,-0.1062,-0.01709,-0.029297,-0.018311,0.032959,0.095215,0.050049,0.10132,0.10132,0.057373,-0.032959,0.0097656,-0.054932,-0.057373,-0.0024414,-0.026855,0.068359,0.14648,0.18555,0.17212,0.13184,0.21118,0.19775,0.22217,0.27344,0.21729,0.26611,0.2832,0.26367,0.18555,0.22095,0.16846,0.15015,0.18188,0.13916,0.20386,0.2356,0.22217,0.17212,0.096436,0.14038,0.11108,0.13916,0.21362,0.18677,0.28076,0.31616,0.30273,0.24414,0.29663,0.2356,0.20264,0.21729,0.25391,0.15015,0.15625,0.096436,-0.0024414,-0.084229,-0.053711,-0.095215,-0.070801,-0.025635,-0.057373,0.030518,0.068359,0.065918,0.039063,0.10254,0.06958,0.065918,0.12939,0.20508,0.17822,0.25879,0.27222,0.22827,0.16113,0.17944,0.091553,0.10132,0.14038,0.090332,0.15625,0.19409,0.20508,0.1709,0.23682,0.20752,0.19409,0.24658,0.3418,0.31616,0.39185,0.41138,0.37598,0.31006,0.354,0.29053,0.26123,0.28564,0.20508,0.24414,0.25269,0.20142,0.12451,0.16357,0.1062,0.06958,0.10742,0.17944,0.14038,0.20752,0.24292,0.20142,0.12695,0.16968,0.097656,0.065918,0.083008,-0.013428,0.01709,-0.021973,-0.080566,-0.19897,-0.22217,-0.32715,-0.41992,-0.45532,-0.39673,-0.41138,-0.30029,-0.2417,-0.21118,-0.24292,-0.15991,-0.20142,-0.19897,-0.14282,-0.18677,-0.11963,-0.093994,-0.12329,-0.21362,-0.20386,-0.29175,-0.36377,-0.37354,-0.32715,-0.36133,-0.31128,-0.31006,-0.36743,-0.47607,-0.48096,-0.57251,-0.64453,-0.68359,-0.69336,-0.75439,-0.78003,-0.82764,-0.8606,-0.81909,-0.81665,-0.76904,-0.67017,-0.52124,-0.45166,-0.28931,-0.15503,-0.075684,-0.056152,0.041504,0.015869,0.024414,0.079346,0.15137,0.11475,0.15747,0.17578,0.16724,0.2417,0.20752,0.18311,0.21118,0.27832,0.24414,0.31738,0.3418,0.31616,0.25269,0.29297,0.22949,0.21118,0.25757,0.32593,0.29297,0.36255,0.36621,0.33203,0.30029,0.32837,0.28931,0.31738,0.354,0.30518,0.37842,0.39795,0.36255,0.29907,0.32471,0.23926,0.19287,0.21484,0.26001,0.20874,0.271,0.27222,0.22583,0.15625,0.18799,0.11841,0.10742,0.12329,0.041504,0.070801,0.068359,-0.0073242,-0.098877,-0.081787,-0.16235,-0.19897,-0.13428,-0.15625,-0.074463,0.036621,0.068359,0.050049,0.040283,0.12695,0.091553,0.12695,0.19287,0.15259,0.22705,0.25269,0.22461,0.12939,0.13428,0.020752,-0.078125,-0.10742,-0.12207,-0.25635,-0.27588,-0.32104,-0.4248,-0.52856,-0.49438,-0.49683,-0.41382,-0.2832,-0.2478,-0.096436,0.028076,0.087891,0.10376,0.21729,0.23315,0.2478,0.31738,0.31128,0.36499,0.42725,0.43213,0.36987,0.26367,0.27588,0.16846,0.11108,0.12695,0.054932,0.093994,0.13062,0.12695,0.078125,0.14038,0.095215,0.050049,0.065918,0.096436,0.021973,0.042725,-0.012207,-0.091553,-0.20264,-0.19165,-0.26978,-0.29053,-0.25146,-0.17578,-0.20386,-0.16846,-0.15869,-0.20142,-0.14282,-0.15991,-0.15015,-0.074463,0.05249,0.040283,0.12817,0.17822,0.1709,0.10742,0.14404,0.096436,0.080566,0.11108,0.15381,0.090332,0.10864,0.091553,0.032959,0.072021,-0.0061035,-0.047607,-0.0354,0.013428,-0.0354,0.05249,0.072021,0.062256,0.021973,0.06958,0.01709,-0.020752,0.013428,0.05249,-0.0048828,0.05127,0.041504,-0.0097656,-0.074463,-0.025635,-0.075684,-0.05249,-0.0024414,-0.048828,0.028076,0.037842,0.0097656,-0.0354,0.037842,-0.0061035,0.028076,0.081787,0.15869,0.13306,0.19897,0.21606,0.18311,0.12573,0.19409,0.14404,0.1709,0.22461,0.16846,0.22827,0.25513,0.2124,0.14038,0.18188,0.10986,0.081787,0.12329,0.081787,0.14771,0.21729,0.22949,0.19409,0.14648,0.20508,0.15625,0.18555,0.24902,0.20508,0.28687,0.31372,0.29419,0.23438,0.28076,0.2063,0.15869,0.16968,0.22461,0.16846,0.22461,0.25146,0.19653,0.23926,0.18921,0.13062,0.11963,0.18555,0.12451,0.18433,0.2124,0.16968,0.059814,0.056152,-0.053711,-0.13062,-0.1355,-0.20142,-0.14648,-0.084229,-0.068359,-0.093994,-0.14893,-0.084229,-0.12573,-0.10376,-0.028076,-0.073242,-0.0085449,0.048828,0.025635,0.0024414,0.076904,0.042725,0.020752,0.074463,0.16968,0.14038,0.22461,0.24658,0.21362,0.1416,0.18066,0.1123,0.078125,0.087891,0.0036621,0.041504,0.018311,-0.036621,-0.11597,-0.12451,-0.22095,-0.28076,-0.27344,-0.2124,-0.24048,-0.16968,-0.10742,-0.1062,-0.15503,-0.078125,-0.12207,-0.11963,-0.058594,-0.093994,-0.020752,0.020752,-0.0073242,-0.046387,-0.12451,-0.074463,-0.12085,-0.097656,-0.047607,-0.10498,-0.061035,-0.064697,-0.087891,-0.15015,-0.11353,-0.17212,-0.18799,-0.13306,-0.061035,-0.084229,-0.015869,-0.010986,-0.0354,-0.084229,-0.026855,-0.067139,-0.05127,-0.014648,-0.084229,-0.03418,-0.036621,-0.090332,-0.16357,-0.11353,-0.15503,-0.15015,-0.068359,-0.087891,0.01709,0.087891,0.11108,0.089111,0.042725,0.10742,0.075684,0.089111,0.13794,0.091553,0.14648,0.19653,0.19287,0.15991,0.24536,0.23193,0.25513,0.34424,0.44556,0.43945,0.52734,0.5481,0.50049,0.44067,0.4895,0.44312,0.44922,0.47974,0.41382,0.44556,0.42725,0.36133,0.24414,0.23438,0.12207,0.037842,0.026855,0.073242,0.0036621,0.064697,0.095215,0.068359,0.14282,0.1123,0.084229,0.12695,0.20508,0.177,0.2478,0.28442,0.25879,0.19287,0.2478,0.19775,0.16357,0.19775,0.26367,0.20142,0.25024,0.2417,0.17822,0.19775,0.11108,0.029297,-0.0097656,0.019531,-0.083008,-0.068359,-0.084229,-0.15381,-0.26733,-0.27588,-0.38696,-0.46753,-0.4895,-0.45288,-0.47607,-0.38696,-0.31372,-0.26367,-0.22461,-0.093994,-0.075684,-0.015869,0.070801,0.036621,0.11841,0.1355,0.12939,0.074463,0.13062,0.10254,0.085449,0.11841,0.16968,0.12085,0.16602,0.14648,0.095215,0.0097656,0.036621,-0.040283,-0.05249,-0.025635,-0.078125,-0.0024414,0.03418,0.018311,-0.031738,0.0073242,-0.097656,-0.14648,-0.14404,-0.12329,-0.19043,-0.13306,-0.14648,-0.18066,-0.24292,-0.19653,-0.21362,-0.17212,-0.074463,-0.074463,0.046387,0.10986,0.11963,0.087891,0.13916,0.059814,0.019531,0.041504,0.076904,0.01709,0.090332,0.11475,0.10132,0.072021,0.15015,0.12939,0.16602,0.24658,0.2124,0.30518,0.35034,0.32837,0.26123,0.18066,0.19653,0.14038,0.15015,0.19897,0.13184,0.18555,0.22095,0.18555,0.15381,0.24292,0.2063,0.21606,0.30762,0.26611,0.3418,0.39429,0.37476,0.29663,0.2124,0.2356,0.15991,0.15015,0.18555,0.11108,0.17578,0.20264,0.19653,0.15625,0.22217,0.17334,0.16968,0.2124,0.27832,0.21606,0.26733,0.27588,0.21484,0.13794,0.19531,0.13916,0.15381,0.22583,0.19653,0.26123,0.3186,0.30884,0.25513,0.32227,0.28809,0.27222,0.31616,0.29541,0.35034,0.42358,0.43335,0.37476,0.30273,0.33569,0.26855,0.25391,0.28809,0.21851,0.26733,0.29053,0.25269,0.18921,0.23804,0.18555,0.15259,0.18677,0.25757,0.21851,0.26367,0.26733,0.2124,0.10742,0.12939,0.053711,0,0.012207,-0.067139,-0.0354,-0.048828,-0.097656,-0.20508,-0.21484,-0.28931,-0.31982,-0.28076,-0.20752,-0.24414,-0.17578,-0.12695,-0.12329,-0.13306,-0.041504,-0.048828,0.0073242,0.079346,0.075684,0.17456,0.23804,0.25269,0.22705,0.28442,0.23438,0.22095,0.24048,0.28687,0.23438,0.26367,0.23804,0.18799,0.087891,0.10254,0.03418,0.018311,0.053711,0.13428,0.087891,0.14038,0.14526,0.098877,0.16113,0.097656,0.05249,0.087891,0.14648,0.1001,0.17822,0.18799,0.15259,0.097656,0.14038,0.078125,0.087891,0.14648,0.21118,0.18799,0.23804,0.22827,0.19043,0.24048,0.16968,0.13306,0.16235,0.22217,0.16846,0.22949,0.23071,0.19653,0.12695,0.15381,0.087891,0.058594,0.096436,0.14038,0.087891,0.12085,0.098877,0.03418,0.095215,0.037842,-0.0012207,0.039063,0.089111,0.036621,0.11597,0.1355,0.10376,0.042725,0.089111,0.031738,0.0012207,0.042725,0.10864,0.070801,0.13062,0.13062,0.087891,0.13916,0.076904,0.031738,0.063477,0.11963,0.064697,0.1355,0.16357,0.13306,0.075684,0.12085,0.068359,0.0354,0.079346,0.14404,0.081787,0.14038,0.15625,0.10132,0.15015,0.087891,0.029297,0.0354,0.087891,0.028076,0.078125,0.10498,0.092773,0.032959,0.070801,0.031738,-0.0036621,0.046387,0.098877,0.03418,0.093994,0.10498,0.057373,0.12207,0.06958,0.031738,0.058594,0.12085,0.08667,0.17822,0.2124,0.21484,0.16357,0.21851,0.17578,0.15137,0.17578,0.24902,0.19287,0.23682,0.24414,0.18555,0.11597,0.16113,0.10132,0.12085,0.16846,0.13062,0.19409,0.21606,0.20508,0.14893,0.19287,0.14038,0.11841,0.1416,0.20508,0.15015,0.20874,0.21118,0.17212,0.1001,0.13062,0.056152,0.032959,0.048828,-0.028076,0.021973,0.023193,-0.0073242,-0.061035,-0.028076,-0.08667,-0.12207,-0.079346,-0.12695,-0.048828,0.037842,0.057373,0.039063,-0.018311,0.039063,-0.03418,-0.026855,0.0097656,0.041504,0.0024414,0.018311,-0.029297,-0.093994,-0.061035,-0.13672,-0.15991,-0.093994,-0.0024414,-0.013428,0.090332,0.12939,0.11597,0.065918,0.1355,0.072021,0.062256,0.12573,0.17212,0.11475,0.14038,0.11475,0.057373,0.10132,0.032959,-0.018311,0.0036621,0.039063,-0.0354,0.032959,0.023193,-0.025635,-0.087891,-0.042725,-0.11597,-0.1416,-0.083008,-0.14282,-0.080566,-0.025635,-0.031738,-0.10132,-0.059814,-0.11353,-0.16846,-0.15259,-0.097656,-0.17456,-0.12939,-0.10742,-0.14893,-0.22095,-0.16602,-0.19653,-0.18799,-0.095215,-0.10742,-0.0061035,0.072021,0.093994,0.068359,0.13306,0.089111,0.05249,0.076904,0.15747,0.1123,0.17212,0.20752,0.17456,0.10742,0.16235,0.10864,0.073242,0.11597,0.18555,0.13062,0.16724,0.15991,0.096436,0.12085,0.06958,0.01709,0.03418,0.10132,0.075684,0.16602,0.20874,0.20996,0.15625,0.20752,0.16357,0.14404,0.16357,0.22095,0.15991,0.20264,0.20508,0.14893,0.17944,0.1062,0.050049,0.062256,0.10864,0.054932,0.11597,0.14771,0.14648,0.096436,0.14404,0.1001,0.076904,0.11353,0.19409,0.14648,0.21606,0.22095,0.18799,0.13062,0.16602,0.10132,0.10254,0.13062,0.074463,0.14771,0.16968,0.14282,0.083008,0.12817,0.06958,0.032959,0.056152,0.10132,0.0354,0.076904,0.056152,-0.0097656,-0.076904,-0.047607,-0.10864,-0.10376,-0.046387,-0.097656,0.0012207,0.032959,0.030518,-0.021973,0.028076,-0.064697,-0.098877,-0.079346,-0.030518,-0.089111,-0.03418,-0.01709,-0.045166,-0.091553,-0.039063,-0.080566,-0.057373,0.0097656,-0.056152,0.030518,0.054932,0.0354,-0.012207,-0.074463,-0.029297,-0.085449,-0.068359,-0.0073242,-0.061035,-0.0012207,0.018311,-0.025635,-0.073242,-0.028076,-0.10498,-0.13916,-0.097656,-0.15625,-0.10132,-0.05249,-0.073242,-0.13672,-0.10498,-0.16235,-0.19897,-0.16602,-0.096436,-0.12329,-0.020752,0.042725,0.041504,0.0024414,0.090332,0.057373,0.062256,0.13672,0.2063,0.16968,0.22583,0.23804,0.18555,0.22949,0.15991,0.11108,0.12207,0.19165,0.13062,0.17944,0.20508,0.19287,0.13672,0.19043,0.14648,0.12451,0.16602,0.12085,0.18799,0.20752,0.18066,0.10986,0.13062,0.048828,-0.020752,-0.026855,0.0097656,-0.048828,0.042725,0.065918,0.064697,0.0073242,0.070801,0.039063,0.029297,0.059814,0.1416,0.096436,0.14771,0.16113,0.11841,0.15625,0.093994,0.042725,0.059814,0.10132,0.0354,0.090332,0.097656,0.058594,0.0012207,0.031738,-0.048828,-0.058594,-0.031738,-0.081787,-0.0097656,0.031738,0.020752,-0.0012207,-0.029297,0.032959,-0.0097656,0.026855,0.085449,0.061035,0.13428,0.17212,0.14893,0.093994,0.12817,0.056152,0.021973,0.043945,0.1001,0.042725,0.087891,0.097656,0.046387,-0.025635,0.026855,-0.041504,-0.023193,0.030518,0.06958,0.042725,0.067139,0.041504,-0.0097656,0.053711,-0.0354,-0.056152,-0.0012207,0.054932,0.0012207,0.06958,0.091553,0.062256,0.021973,0.080566,0.042725,0.048828,0.11963,0.070801,0.14282,0.177,0.15869,0.10986,0.15869,0.085449,0.036621,0.050049,0.096436,0.0354,0.084229,0.090332,0.03418,-0.040283,0.014648,-0.068359,-0.070801,-0.0097656,-0.058594,0.028076,0.087891,0.083008,0.039063,0.10986,0.068359,0.036621,0.083008,0.15015,0.097656,0.16113,0.18188,0.14038,0.050049,0.092773,0.021973,-0.0024414,0.05249,0.10498,0.072021,0.11597,0.10986,0.070801,0.11963,0.062256,0.026855,0.050049,0.12085,0.080566,0.14038,0.16357,0.13916,0.058594,0.1123,0.048828,0.031738,0.089111,0.046387,0.12695,0.18677,0.19775,0.16235,0.21851,0.16968,0.12817,0.13428,0.2063,0.15869,0.22461,0.24658,0.23804,0.15991,0.2124,0.16113,0.14404,0.18311,0.25513,0.21362,0.26367,0.271,0.22217,0.26489,0.20142,0.16724,0.17578,0.21851,0.15991,0.19653,0.21118,0.17822,0.1001,0.11963,0.05127,0.012207,0.0354,0.090332,0.0354,0.080566,0.076904,0.031738,0.072021,0.012207,-0.015869,0.025635,0.076904,0.057373,0.14038,0.18066,0.17456,0.12817,0.18799,0.14038,0.12695,0.15991,0.22705,0.18188,0.23926,0.23193,0.17578,0.10498,0.14648,0.087891,0.095215,0.13672,0.087891,0.16724,0.18677,0.15259,0.078125,0.1062,0.032959,-0.0061035,0.046387,0.090332,0.029297,0.093994,0.091553,0.05249,-0.0097656,0.045166,-0.0097656,0.024414,0.079346,0.042725,0.12329,0.16602,0.15015,0.096436,0.15747,0.10132,0.068359,0.097656,0.17212,0.10742,0.16602,0.16113,0.10986,0.0354,0.087891,0.023193,0.010986,0.06958,0.0097656,0.075684,0.12573,0.096436,0.025635,0.062256,-0.0024414,-0.040283,-0.0061035,0.048828,-0.029297,0.03418,0.029297,-0.0048828,-0.061035,-0.012207,-0.059814,-0.058594,0.0036621,-0.03418,0.062256,0.095215,0.090332,0.0354,0.080566,0.023193,-0.030518,-0.0061035,-0.062256,-0.031738,0.013428,0.025635,0.0036621,-0.076904,-0.013428,-0.058594,-0.067139,-0.0048828,-0.041504,0.029297,0.061035,0.064697,0.018311,0.070801,0.018311,-0.0073242,0.028076,0.081787,0.036621,0.10132,0.10132,0.054932,-0.0354,0.0097656,-0.05127,-0.059814,-0.015869,-0.048828,0.028076,0.078125,0.084229,0.042725,0.095215,0.037842,0.0024414,0.015869,0.068359,0.020752,0.085449,0.10742,0.1001,0.048828,0.10742,0.075684,0.084229,0.13794,0.22827,0.19653,0.24414,0.24414,0.19165,0.22705,0.14404,0.079346,0.079346,0.11963,0.059814,0.10986,0.12695,0.10376,0.05249,0.11841,0.078125,0.062256,0.1123,0.17578,0.14282,0.19287,0.19531,0.13916,0.19165,0.11597,0.080566,0.10132,0.14648,0.10132,0.17578,0.18555,0.15503,0.076904,0.10132,0.019531,-0.015869,0.013428,0.048828,-0.023193,0.042725,0.032959,-0.018311,-0.070801,-0.023193,-0.08667,-0.05127,0.0073242,-0.05249,0.026855,0.045166,0.020752,-0.025635,0.043945,-0.029297,-0.045166,-0.0024414,0.081787,0.021973,0.091553,0.095215,0.054932,-0.014648,0.046387,-0.0354,-0.026855,0.01709,0.057373,0.01709,0.070801,0.05249,-0.014648,-0.064697,-0.015869,-0.05127,-0.0061035,-0.067139,-0.0097656,0.05249,0.042725,-0.013428,-0.091553,-0.047607,-0.11353,-0.11353,-0.056152,-0.098877,-0.025635,0.019531,0.023193,-0.024414,0.05127,-0.0024414,-0.029297,0.0036621,0.068359,0.023193,0.081787,0.087891,0.032959,0.058594,-0.028076,-0.10132,-0.12817,-0.075684,-0.13306,-0.074463,-0.040283,-0.065918,-0.13794,-0.10132,-0.15259,-0.177,-0.14526,-0.063477,-0.084229,-0.015869,0.021973,0,-0.064697,0.0012207,-0.056152,-0.063477,-0.018311,-0.065918,-0.0073242,0.031738,0.023193,-0.028076,0.019531,-0.05127,-0.080566,-0.054932,-0.015869,-0.070801,-0.031738,-0.0354,-0.081787,-0.16724,-0.14038,-0.20874,-0.21484,-0.15625,-0.18188,-0.074463,-0.0012207,0.037842,0.012207,0.084229,0.036621,0.026855,0.065918,0.13428,0.10376,0.17578,0.20142,0.17822,0.11108,0.13794,0.076904,0.058594,0.10498,0.17944,0.13916,0.19897,0.19653,0.16968,0.12695,0.19653,0.1709,0.20508,0.26123,0.20874,0.27832,0.29053,0.25391,0.18799,0.22461,0.15503,0.13062,0.15991,0.22583,0.17822,0.23804,0.25146,0.20874,0.14648,0.19531,0.13794,0.15869,0.20386,0.14404,0.20874,0.2124,0.16602,0.098877,0.12939,0.05249,0.012207,0.029297,0.083008,0.0097656,0.072021,0.072021,0.01709,-0.043945,0.020752,-0.047607,-0.030518,0.021973,-0.048828,0.032959,0.068359,0.057373,0.0024414,0.059814,0.021973,0.0097656,0.039063,0.10864,0.023193,0.046387,0.023193,-0.048828,-0.10742,-0.05127,-0.10376,-0.085449,-0.025635,-0.067139,-0.0024414,0,-0.0354,-0.12085,-0.10742,-0.18188,-0.23315,-0.19897,-0.21606,-0.15015,-0.081787,-0.05127,-0.093994,-0.16357,-0.11108,-0.1709,-0.15625,-0.075684,-0.097656,-0.0061035,0.045166,0.048828,-0.0024414,0.061035,0.0097656,-0.020752,0.0097656,0.062256,0.021973,0.081787,0.096436,0.062256,-0.025635,0.014648,-0.058594,-0.062256,-0.015869,-0.05127,0.024414,0.070801,0.087891,0.062256,0.13062,0.12573,0.11719,0.17212,0.26001,0.22461,0.28564,0.29663,0.271,0.20264,0.25146,0.20142,0.18677,0.22583,0.17822,0.2478,0.27954,0.26123,0.20508,0.23682,0.14648,0.11108,0.12329,0.16602,0.10132,0.15747,0.17212,0.1355,0.063477,0.098877,0.024414,0.012207,0.05249,-0.0085449,0.065918,0.092773,0.087891,0.042725,0.085449,0.032959,0.0048828,0.032959,0.096436,0.053711,0.13672,0.15869,0.13428,0.072021,0.12329,0.050049,0.031738,0.061035,0.12085,0.074463,0.12695,0.10742,0.045166,0.087891,0.026855,-0.015869,0.031738,0.080566,0.029297,0.11353,0.15381,0.14648,0.10376,0.16724,0.1123,0.089111,0.12817,0.18433,0.14038,0.19165,0.18921,0.13672,0.17334,0.097656,0.05127,0.057373,0.11475,0.068359,0.12451,0.16602,0.1355,0.075684,0.13184,0.078125,0.058594,0.11597,0.059814,0.12817,0.17944,0.17334,0.11108,0.14648,0.068359,-0.010986,-0.023193,0.021973,-0.05249,0.012207,0.015869,-0.01709,-0.091553,-0.072021,-0.15503,-0.19775,-0.16724,-0.19653,-0.11963,-0.057373,-0.028076,-0.056152,-0.11475,-0.054932,-0.11108,-0.10986,-0.059814,-0.11353,-0.063477,-0.057373,-0.081787,-0.1416,-0.097656,-0.16357,-0.19653,-0.1709,-0.096436,-0.11841,-0.041504,0,-0.015869,-0.040283,0.032959,-0.0097656,0.028076,0.078125,0.054932,0.13306,0.16968,0.15991,0.10742,0.14648,0.091553,0.076904,0.10376,0.16602,0.12939,0.18188,0.18799,0.15869,0.083008,0.12085,0.05249,0.046387,0.084229,0.15991,0.13062,0.16602,0.16846,0.12329,0.18188,0.13062,0.10742,0.1355,0.2002,0.15747,0.21118,0.22827,0.19409,0.11963,0.15137,0.089111,0.078125,0.11963,0.16602,0.11841,0.15747,0.12939,0.087891,0.13428,0.064697,0.041504,0.078125,0.13794,0.10132,0.19287,0.21118,0.18677,0.11597,0.16968,0.10498,0.090332,0.13306,0.19409,0.1355,0.18555,0.17578,0.11353,0.15747,0.072021,0.01709,0.01709,0.05249,-0.019531,0.050049,0.032959,-0.0061035,-0.063477,-0.019531,-0.084229,-0.10132,-0.054932,0.014648,-0.0036621,0.079346,0.096436,0.065918,0.12939,0.072021,0.031738,0.042725,0.10986,0.054932,0.11963,0.15991,0.13306,0.064697,0.11475,0.056152,0.015869,0.05249,0.12085,0.068359,0.11841,0.13184,0.065918,0.11841,0.064697,0.018311,0.041504,0.11108,0.063477,0.1416,0.15991,0.13062,0.056152,0.072021,-0.0024414,-0.03418,-0.0085449,-0.061035,-0.0024414,0.048828,0.059814,0.013428,0.057373,0.0012207,-0.054932,-0.036621,0.029297,-0.0097656,0.062256,0.080566,0.075684,0.014648,0.079346,0.041504,0.026855,0.059814,0.1416,0.097656,0.16357,0.17944,0.12695,0.050049,0.1001,0.048828,0.046387,0.097656,0.05127,0.10986,0.13306,0.10498,0.029297,0.054932,-0.013428,-0.040283,-0.0097656,0.064697,0.013428,0.089111,0.10864,0.090332,0.041504,0.089111,0.046387,0.050049,0.084229,0.030518,0.10376,0.10498,0.073242,-0.0085449,-0.10742,-0.11353,-0.19897,-0.2356,-0.2417,-0.32715,-0.33081,-0.37354,-0.46021,-0.59326,-0.61401,-0.71045,-0.75439,-0.74707,-0.80811,-0.7605,-0.73608,-0.74951,-0.76294,-0.68115,-0.66528,-0.62134,-0.51636,-0.39307,-0.36133,-0.24658,-0.18188,-0.17822,-0.20264,-0.13794,-0.17944,-0.17822,-0.13428,-0.21851,-0.20386,-0.21851,-0.32227,-0.47485,-0.53467,-0.68481,-0.79712,-0.84595,-0.88989,-1.0193,-1.0754,-1.1584,-1.2341,-1.2561,-1.2622,-1.2524,-1.2012,-1.0754,-0.9436,-0.89722,-0.79956,-0.73975,-0.73853,-0.68115,-0.70557,-0.67993,-0.58716,-0.45776,-0.40283,-0.26123,-0.15625,-0.11353,-0.13062,-0.029297,-0.05249,-0.065918,-0.015869,-0.08667,-0.043945,-0.024414,-0.042725,-0.087891,-0.14404,-0.080566,-0.12695,-0.12329,-0.079346,-0.14771,-0.10864,-0.089111,-0.089111,-0.12939,-0.03418,-0.026855,0.0097656,0.097656,0.23926,0.2356,0.32959,0.36865,0.33081,0.37476,0.29175,0.20874,0.17944,0.16113,0.040283,0.019531,-0.023193,-0.076904,-0.14282,-0.084229,-0.075684,-0.028076,0.062256,0.18066,0.17944,0.28076,0.3064,0.29663,0.25146,0.3064,0.2771,0.27954,0.3064,0.25757,0.29541,0.28442,0.21851,0.10742,0.076904,-0.026855,-0.11719,-0.15137,-0.16357,-0.26123,-0.23315,-0.22827,-0.27832,-0.36499,-0.35889,-0.44434,-0.48462,-0.51147,-0.60303,-0.57251,-0.57129,-0.59082,-0.65063,-0.62134,-0.67993,-0.68604,-0.62622,-0.53467,-0.53589,-0.42969,-0.33691,-0.26855,-0.22339,-0.096436,-0.047607,0.061035,0.20142,0.25513,0.41016,0.50659,0.52979,0.5188,0.5835,0.52368,0.49316,0.50903,0.5481,0.50415,0.56763,0.56519,0.51147,0.44556,0.47852,0.41626,0.4126,0.45166,0.39185,0.43213,0.46387,0.43579,0.35767,0.3894,0.32104,0.28442,0.31616,0.36621,0.30396,0.36133,0.35767,0.32227,0.24658,0.28442,0.21851,0.20996,0.25391,0.20264,0.26001,0.2832,0.25269,0.18188,0.21729,0.15259,0.11353,0.14771,0.23193,0.20508,0.28076,0.3418,0.33936,0.32227,0.42358,0.42358,0.45044,0.53345,0.52124,0.60181,0.66284,0.6604,0.60669,0.64331,0.57739,0.52612,0.52734,0.55054,0.4834,0.53467,0.53711,0.49072,0.41016,0.43091,0.34546,0.31372,0.34424,0.3064,0.36865,0.40649,0.40405,0.34302,0.36499,0.28564,0.22827,0.23804,0.29053,0.23804,0.29663,0.3125,0.30273,0.23926,0.28931,0.2478,0.22949,0.26001,0.33569,0.29053,0.32715,0.3186,0.26733,0.18799,0.23438,0.17944,0.17944,0.22461,0.17578,0.23438,0.25513,0.23071,0.16357,0.19897,0.14893,0.13428,0.177,0.25146,0.20874,0.25269,0.26978,0.21362,0.14648,0.18555,0.13428,0.13062,0.16602,0.096436,0.15015,0.15137,0.12695,0.05249,0.061035,-0.019531,-0.054932,-0.0354,-0.10376,-0.045166,-0.0061035,-0.021973,-0.078125,-0.13428,-0.065918,-0.097656,-0.057373,0.0354,0.013428,0.11353,0.16846,0.16602,0.14038,0.21729,0.177,0.15381,0.18921,0.26001,0.21484,0.2832,0.28198,0.23926,0.16968,0.19409,0.13306,0.12329,0.16724,0.12085,0.19409,0.25024,0.26123,0.2063,0.271,0.21729,0.17944,0.2124,0.25635,0.17944,0.22095,0.21484,0.15259,0.057373,0.087891,0.015869,0.0036621,0.079346,0.041504,0.11841,0.16602,0.16724,0.13428,0.19043,0.13428,0.090332,0.10986,0.16602,0.11963,0.17578,0.17578,0.11475,0.015869,0.036621,-0.03418,-0.040283,0.032959,0.10498,0.085449,0.15869,0.18555,0.14893,0.21484,0.177,0.15137,0.18799,0.271,0.22217,0.28687,0.30762,0.2832,0.19409,0.22461,0.1416,0.090332,0.11108,0.15015,0.078125,0.10376,0.093994,0.026855,0.056152,-0.013428,-0.047607,-0.020752,0.048828,0.0097656,0.095215,0.12939,0.12085,0.072021,0.1416,0.10376,0.096436,0.14893,0.23682,0.2063,0.26123,0.27588,0.2356,0.28076,0.22583,0.19287,0.19775,0.24414,0.19897,0.25024,0.26978,0.2478,0.177,0.22217,0.17456,0.15747,0.19775,0.26123,0.2124,0.25635,0.26001,0.21973,0.27832,0.2063,0.16602,0.18555,0.23682,0.19287,0.26489,0.26978,0.24536,0.16968,0.18921,0.10986,0.072021,0.081787,0.14038,0.096436,0.14648,0.15503,0.1062,0.15747,0.097656,0.048828,0.058594,0.10986,0.05249,0.12695,0.14038,0.11108,0.05249,0.087891,0.019531,-0.0024414,0.059814,0.11719,0.084229,0.16968,0.19043,0.15625,0.1001,0.14648,0.089111,0.093994,0.13672,0.078125,0.1355,0.16357,0.13794,0.072021,0.11841,0.057373,0.023193,0.043945,0.10376,0.053711,0.12207,0.12939,0.089111,0.021973,0.058594,-0.0036621,0.0073242,0.048828,-0.013428,0.068359,0.080566,0.047607,-0.019531,-0.089111,-0.057373,-0.096436,-0.064697,0.0036621,-0.05127,0.021973,0.032959,0.0061035,0.058594,0.015869,-0.020752,0.0024414,0.075684,0.048828,0.14404,0.19409,0.2063,0.16357,0.20874,0.15991,0.12329,0.13794,0.19409,0.1355,0.19653,0.2063,0.177,0.10742,0.14526,0.085449,0.06958,0.12329,0.070801,0.13306,0.17944,0.177,0.11841,0.15381,0.091553,0.050049,0.076904,0.14771,0.10742,0.16968,0.19653,0.16357,0.093994,0.13916,0.080566,0.068359,0.098877,0.16724,0.12085,0.15625,0.1355,0.095215,0.12329,0.05249,0.013428,0.018311,0.074463,0.031738,0.085449,0.11353,0.087891,0.029297,0.075684,0.024414,0.013428,0.058594,0.11963,0.06958,0.11108,0.11108,0.068359,0.12329,0.072021,0.043945,0.072021,0.11597,0.075684,0.15991,0.16724,0.14404,0.089111,0.12695,0.058594,0.05127,0.078125,0.13794,0.1001,0.15625,0.14893,0.10498,0.15625,0.097656,0.064697,0.087891,0.1355,0.087891,0.14893,0.1709,0.13916,0.080566,0.12207,0.050049,0.024414,0.054932,0.11108,0.062256,0.11597,0.12451,0.068359,0.013428,0.041504,-0.023193,-0.012207,0.029297,-0.054932,0.012207,0.024414,-0.0097656,-0.054932,-0.0012207,-0.059814,-0.087891,-0.019531,-0.046387,0.031738,0.10742,0.13062,0.089111,0.036621,0.10864,0.05249,0.061035,0.12939,0.079346,0.14282,0.17578,0.13916,0.068359,0.097656,0.025635,-0.015869,0.014648,0.064697,0.0073242,0.062256,0.079346,0.043945,-0.042725,0.015869,-0.061035,-0.056152,0.025635,-0.018311,0.059814,0.10498,0.090332,0.041504,0.10132,0.05127,0.0085449,0.041504,0.11353,0.068359,0.13916,0.15381,0.13306,0.063477,0.12207,0.079346,0.054932,0.091553,0.032959,0.068359,0.079346,0.042725,-0.0354,-0.015869,-0.087891,-0.14038,-0.12573,-0.059814,-0.095215,-0.029297,0.0036621,-0.015869,-0.063477,-0.0036621,-0.046387,-0.050049,0.0073242,0.06958,0.031738,0.068359,0.074463,0.019531,0.050049,-0.015869,-0.056152,-0.040283,0.012207,-0.045166,0.028076,0.037842,0.023193,-0.048828,0.0097656,-0.047607,-0.058594,-0.0061035,-0.042725,0.037842,0.08667,0.087891,0.048828,0.10742,0.045166,0.0012207,0.021973,0.075684,0.037842,0.12085,0.14282,0.12451,0.075684,0.12695,0.064697,0.05249,0.087891,0.15869,0.10986,0.15381,0.14648,0.095215,0.11841,0.048828,0.0036621,0.015869,0.065918,0.032959,0.11108,0.15015,0.14038,0.1001,0.15625,0.1001,0.091553,0.13062,0.18799,0.12939,0.18555,0.17334,0.11353,0.048828,0.083008,0.031738,0.048828,0.10864,0.070801,0.15381,0.20264,0.18677,0.14282,0.19043,0.13428,0.11719,0.16113,0.22827,0.18188,0.23438,0.22095,0.15625,0.080566,0.12329,0.062256,0.061035,0.097656,0.040283,0.10986,0.12939,0.090332,0.032959,0.075684,0.010986,-0.025635,0.023193,0.070801,0.015869,0.073242,0.075684,0.018311,-0.064697,-0.024414,-0.098877,-0.11963,-0.076904,-0.12695,-0.074463,-0.043945,-0.061035,-0.11597,-0.065918,-0.10376,-0.11475,-0.056152,0.0354,-0.0036621,0.081787,0.087891,0.048828,-0.021973,0.040283,-0.021973,-0.023193,0.029297,0.085449,0.039063,0.095215,0.097656,0.037842,0.081787,0.041504,0.0073242,0.036621,0.096436,0.043945,0.085449,0.098877,0.064697,-0.026855,0.01709,-0.058594,-0.072021,-0.028076,-0.083008,-0.018311,0.01709,0.014648,-0.015869,-0.054932,0.010986,-0.013428,0.036621,0.097656,0.062256,0.12451,0.14038,0.11597,0.042725,0.076904,0.018311,0.0036621,0.05127,-0.0048828,0.072021,0.10254,0.1001,0.058594,0.11108,0.064697,0.042725,0.068359,0.11841,0.079346,0.13916,0.14648,0.12695,0.05127,0.081787,0.015869,0.0024414,0.039063,0.10132,0.078125,0.1355,0.12939,0.095215,0.1416,0.074463,0.041504,0.072021,0.13916,0.10742,0.16846,0.18555,0.15747,0.10498,0.15747,0.098877,0.073242,0.11475,0.18066,0.12695,0.16357,0.14893,0.089111,0.13916,0.068359,0.013428,0.023193,0.054932,-0.0024414,0.078125,0.1001,0.064697,0,0.036621,-0.040283,-0.058594,-0.0061035,-0.056152,0.0036621,0.061035,0.064697,0.023193,0.080566,0.023193,-0.021973,0.013428,0.062256,0.0097656,0.089111,0.1001,0.078125,0.0097656,0.048828,-0.024414,-0.048828,-0.030518,0.031738,-0.026855,0.042725,0.0354,-0.0073242,-0.05249,-0.0061035,-0.053711,-0.037842,0.015869,-0.037842,0.048828,0.080566,0.070801,0.0061035,0.072021,0.026855,-0.015869,0.029297,0.098877,0.046387,0.10132,0.10132,0.036621,0.058594,-0.03418,-0.091553,-0.10986,-0.072021,-0.11719,-0.058594,-0.029297,-0.031738,-0.080566,-0.032959,-0.072021,-0.087891,-0.032959,-0.059814,0.026855,0.091553,0.11597,0.089111,0.01709,0.059814,0.0036621,-0.0012207,0.062256,0.0036621,0.1001,0.13794,0.1355,0.1001,0.15015,0.10254,0.06958,0.084229,0.13428,0.074463,0.11353,0.10864,0.068359,-0.012207,0.031738,-0.030518,-0.025635,0.010986,0.064697,0.03418,0.072021,0.068359,0.0354,0.095215,0.043945,0.019531,0.042725,0.098877,0.043945,0.12085,0.11841,0.080566,0.010986,0.042725,-0.013428,0.0036621,0.056152,0.13428,0.10498,0.16357,0.1709,0.1355,0.18188,0.12695,0.093994,0.12207,0.16968,0.11108,0.16968,0.15747,0.10986,0.040283,0.075684,0.013428,0.018311,0.087891,0.15991,0.15015,0.21484,0.20752,0.15015,0.10132,0.13916,0.081787,0.10864,0.15381,0.098877,0.16602,0.18921,0.14771,0.081787,0.11841,0.037842,0.012207,0.043945,0.096436,0.045166,0.076904,0.058594,-0.013428,-0.093994,-0.064697,-0.12573,-0.1062,-0.056152,-0.1062,-0.018311,0.025635,0.014648,-0.01709,0.057373,0.012207,0,0.05249,0.13916,0.074463,0.13306,0.14282,0.080566,0.13672,0.090332,0.032959,0.043945,0.097656,0.037842,0.095215,0.1355,0.1123,0.05127,0.1001,0.048828,0.028076,0.058594,0.13306,0.091553,0.16968,0.18799,0.15259,0.19653,0.13184,0.079346,0.085449,0.14282,0.093994,0.1355,0.17456,0.14282,0.073242,0.098877,0.0354,-0.0061035,0.025635,0.089111,0.043945,0.1001,0.12817,0.090332,0.032959,0.089111,0.040283,0.043945,0.089111,0.059814,0.13794,0.16235,0.15381,0.097656,0.13916,0.078125,0.050049,0.074463,0.13306,0.091553,0.14893,0.15503,0.12085,0.043945,0.087891,0.032959,0.029297,0.080566,0.03418,0.091553,0.12207,0.097656,0.037842,0.073242,0.015869,-0.0097656,0.019531,0.064697,0.018311,0.068359,0.065918,0.036621,-0.0354,0.018311,-0.048828,-0.031738,0.013428,0.059814,0.015869,0.062256,0.037842,-0.020752,-0.081787,-0.039063,-0.074463,-0.023193,0.046387,-0.0012207,0.091553,0.1123,0.095215,0.0354,0.089111,0.026855,0.012207,0.061035,0.11475,0.070801,0.087891,0.067139,0.0036621,0.048828,-0.023193,-0.054932,-0.025635,0.040283,-0.0061035,0.08667,0.11108,0.085449,0.032959,0.097656,0.053711,0.046387,0.11108,0.17334,0.15015,0.21118,0.20874,0.15137,0.2002,0.13672,0.084229,0.093994,0.12939,0.057373,0.11108,0.12085,0.096436,0.025635,0.063477,0.0012207,-0.0061035,0.061035,0.14038,0.093994,0.14893,0.15381,0.10498,0.15869,0.11353,0.061035,0.079346,0.14648,0.10132,0.16724,0.19043,0.16357,0.084229,0.13916,0.089111,0.053711,0.087891,0.15991,0.11108,0.16724,0.17944,0.12695,0.16846,0.1123,0.064697,0.068359,0.11963,0.080566,0.14038,0.17334,0.16724,0.096436,0.13916,0.087891,0.058594,0.080566,0.15869,0.10742,0.15381,0.177,0.12695,0.057373,0.1001,0.048828,0.05249,0.098877,0.039063,0.10742,0.10986,0.090332,0.0036621,0.032959,-0.037842,-0.074463,-0.054932,-0.0036621,-0.057373,-0.012207,-0.0073242,-0.037842,-0.095215,-0.041504,-0.070801,-0.048828,0.018311,-0.018311,0.076904,0.093994,0.087891,0.039063,0.076904,0.021973,-0.0097656,0.0097656,0.058594,0.01709,0.078125,0.095215,0.054932,-0.01709,0.042725,-0.019531,-0.0061035,0.062256,0.01709,0.078125,0.12695,0.10986,0.056152,0.091553,0.025635,-0.018311,0.023193,0.062256,0.0097656,0.068359,0.080566,0.042725,-0.0097656,0.054932,-0.015869,0.0097656,0.064697,0.13794,0.11719,0.16968,0.15869,0.12695,0.17578,0.10742,0.075684,0.10254,0.15625,0.10376,0.15991,0.17822,0.14771,0.063477,0.11597,0.06958,0.063477,0.11475,0.18188,0.13672,0.18555,0.16846,0.11353,0.14893,0.089111,0.048828,0.064697,0.12329,0.075684,0.14893,0.16846,0.14893,0.080566,0.13062,0.068359,0.03418,0.078125,0.01709,0.054932,0.1001,0.090332,0.021973,0.061035,0.0012207,-0.05127,-0.026855,0.03418,-0.018311,0.048828,0.070801,0.058594,-0.0024414,0.087891,0.025635,0.0097656,0.042725,0.10986,0.061035,0.10864,0.090332,0.014648,0.032959,-0.041504,-0.10376,-0.10376,-0.054932,-0.11353,-0.054932,-0.013428,-0.021973,-0.090332,-0.021973,-0.046387,-0.043945,0.013428,0.096436,0.059814,0.11841,0.13916,0.091553,0.12451,0.048828,-0.018311,-0.01709,0.010986,-0.063477,0,-0.0073242,-0.025635,-0.08667,-0.045166,-0.10498,-0.12939,-0.092773,-0.12573,-0.05249,0.0085449,0.0073242,-0.019531,-0.062256,-0.0036621,-0.028076,-0.0073242,0.054932,0.018311,0.093994,0.11841,0.11597,0.068359,0.11108,0.065918,0.041504,0.056152,0.12085,0.068359,0.11597,0.10742,0.043945,0.072021,-0.018311,-0.089111,-0.1123,-0.065918,-0.12207,-0.048828,-0.012207,-0.029297,-0.078125,-0.013428,-0.054932,-0.062256,-0.0061035,0.062256,0.0097656,0.079346,0.091553,0.054932,-0.0073242,0.03418,-0.042725,-0.042725,-0.0012207,-0.048828,0.032959,0.068359,0.057373,0.019531,0.074463,0.010986,-0.015869,0.046387,0.093994,0.068359,0.14526,0.15991,0.13306,0.070801,0.12939,0.078125,0.072021,0.13428,0.085449,0.14893,0.18921,0.16968,0.11353,0.16479,0.091553,0.0354,0.045166,0.093994,0.03418,0.10498,0.11597,0.067139,-0.019531,0.029297,-0.05249,-0.070801,-0.031738,-0.097656,-0.045166,0.0097656,-0.0061035,-0.046387,-0.098877,-0.048828,-0.06958,-0.019531,0.056152,0.01709,0.091553,0.13916,0.14038,0.089111,0.15869,0.12329,0.10986,0.16602,0.25024,0.20874,0.24414,0.25391,0.19409,0.2417,0.17456,0.12939,0.13306,0.19409,0.14648,0.18799,0.19775,0.15625,0.06958,0.093994,0.032959,0.0012207,0.041504,0.10498,0.072021,0.12085,0.14038,0.097656,0.1416,0.076904,0.039063,0.05249,0.1001,0.054932,0.12817,0.14648,0.12939,0.058594,0.090332,0.043945,0.021973,0.056152,0.12329,0.087891,0.1355,0.14648,0.12695,0.17456,0.12451,0.092773,0.11353,0.16846,0.12939,0.19531,0.20508,0.177,0.11597,0.1416,0.075684,0.046387,0.070801,0.13916,0.095215,0.15015,0.15625,0.11719,0.048828,0.093994,0.040283,0.05249,0.1062,0.06958,0.14404,0.17212,0.14038,0.085449,0.11353,0.043945,0.019531,0.045166,0.1001,0.062256,0.13062,0.1355,0.10864,0.068359,0.11597,0.068359,0.087891,0.1355,0.091553,0.18311,0.2063,0.18799,0.13062,0.17578,0.11353,0.085449,0.11597,0.18311,0.12695,0.18555,0.19653,0.14648,0.078125,0.11963,0.057373,0.032959,0.078125,0.014648,0.063477,0.092773,0.062256,-0.0036621,-0.059814,-0.019531,-0.074463,-0.042725,0.029297,-0.043945,0.031738,0.0354,-0.0097656,-0.067139,-0.026855,-0.092773,-0.10986,-0.059814,-0.11108,-0.054932,0.0061035,-0.0036621,-0.030518,-0.097656,-0.0354,-0.065918,-0.025635,0.0354,-0.013428,0.050049,0.076904,0.048828,-0.0354,0.0097656,-0.064697,-0.072021,-0.019531,-0.062256,-0.0036621,0.036621,0.026855,-0.012207,-0.062256,-0.0036621,-0.040283,-0.023193,0.05249,0.0012207,0.059814,0.096436,0.072021,0.018311,0.075684,0.047607,0.046387,0.10742,0.19287,0.16602,0.20142,0.2063,0.16602,0.20508,0.15381,0.12939,0.1355,0.18555,0.15625,0.2124,0.21851,0.20264,0.12695,0.15991,0.10742,0.081787,0.11963,0.18311,0.13672,0.17456,0.16968,0.13672,0.17578,0.10498,0.050049,0.05249,0.1001,0.05249,0.10376,0.11108,0.081787,0.015869,0.050049,-0.0097656,-0.0354,-0.0097656,-0.048828,0.039063,0.080566,0.068359,0.026855,-0.036621,0.01709,-0.036621,-0.0061035,0.037842,-0.0085449,0.091553,0.11963,0.10986,0.063477,0.12207,0.083008,0.091553,0.13672,0.2063,0.15869,0.22095,0.21851,0.17578,0.11108,0.1416,0.095215,0.1123,0.15015,0.10132,0.17212,0.177,0.16602,0.10498,0.14648,0.084229,0.045166,0.070801,0.12939,0.081787,0.1355,0.14282,0.085449,0.029297,0.070801,0.010986,0.015869,0.065918,0.0024414,0.079346,0.10742,0.084229,0.029297,0.068359,0.01709,-0.0073242,0.03418,0.1001,0.053711,0.1123,0.13306,0.096436,0.036621,0.087891,0.024414,0.015869,0.061035,0.10864,0.059814,0.1001,0.087891,0.032959,0.087891,0.029297,-0.021973,0.023193,0.075684,0.030518,0.095215,0.11963,0.078125,0.0097656,0.063477,0.01709,0.0048828,0.042725,-0.021973,0.05127,0.058594,0.041504,-0.021973,-0.090332,-0.046387,-0.1001,-0.087891,-0.029297,-0.081787,-0.028076,0.0061035,-0.023193,-0.059814,0.0085449,-0.029297,-0.013428,0.056152,0.013428,0.093994,0.13672,0.14038,0.093994,0.12939,0.081787,0.039063,0.057373,0.11597,0.074463,0.14038,0.15869,0.13916,0.059814,0.10498,0.024414,-0.0036621,0.036621,0.085449,0.032959,0.072021,0.065918,0.025635,0.062256,0.0024414,-0.020752,0.023193,0.081787,0.045166,0.12329,0.14648,0.12817,0.078125,0.11841,0.064697,0.058594,0.091553,0.14893,0.11475,0.14648,0.12939,0.058594,0.078125,-0.01709,-0.075684,-0.087891,-0.047607,-0.097656,-0.040283,-0.012207,-0.025635,-0.072021,-0.019531,-0.065918,-0.062256,-0.012207,-0.05249,0.0097656,0.048828,0.031738,-0.013428,-0.068359,-0.023193,-0.081787,-0.056152,0.0024414,-0.042725,0.048828,0.072021,0.053711,-0.0012207,0.063477,-0.013428,-0.024414,0.021973,0.090332,0.045166,0.11108,0.12085,0.081787,0.029297,0.074463,0.023193,0.032959,0.068359,0.015869,0.068359,0.078125,0.040283,-0.029297,0.013428,-0.058594,-0.10132,-0.074463,-0.12695,-0.074463,0.0012207,0.0036621,-0.023193,-0.063477,0.0012207,-0.054932,-0.025635,0.063477,0.023193,0.097656,0.16602,0.16357,0.10742,0.16357,0.10864,0.070801,0.10498,0.16602,0.11597,0.16724,0.16724,0.11597,0.0354,0.070801,0.0073242,0.0048828,0.043945,0.12085,0.08667,0.12817,0.13184,0.083008,0.13306,0.096436,0.067139,0.087891,0.15991,0.11108,0.15137,0.17334,0.1355,0.050049,0.090332,0.031738,0.014648,0.061035,0.014648,0.068359,0.096436,0.087891,0.040283,0.075684,0.013428,-0.040283,-0.01709,0.036621,-0.0073242,0.06958,0.076904,0.070801,0.012207,0.068359,0.026855,0.021973,0.068359,0.13428,0.091553,0.1416,0.13428,0.087891,0.13062,0.075684,0.046387,0.06958,0.13062,0.080566,0.13062,0.14404,0.11597,0.040283,0.073242,0.0073242,-0.0097656,0.042725,0.090332,0.05127,0.087891,0.078125,0.030518,0.078125,-0.0024414,-0.032959,-0.0036621,0.056152,0.0048828,0.075684,0.10132,0.087891,0.041504,0.091553,0.043945,0.031738,0.080566,0.14648,0.11841,0.16968,0.17212,0.12817,0.17212,0.11475,0.078125,0.090332,0.13672,0.089111,0.15625,0.17334,0.14771,0.081787,0.12329,0.064697,0.030518,0.078125,0.13184,0.080566,0.13428,0.12695,0.072021,0.12451,0.070801,0.0048828,0.029297,0.090332,0.032959,0.1001,0.13184,0.10742,0.058594,0.10986,0.048828,0.012207,0.050049,0.12085,0.062256,0.12695,0.12329,0.067139,0.10376,0.030518,-0.01709,-0.0061035,0.048828,-0.018311,0.056152,0.091553,0.068359,0.010986,0.061035,0.0036621,-0.020752,0.026855,0.089111,0.032959,0.095215,0.10986,0.068359,0.012207,0.06958,0.020752,0.018311,0.079346,0.05127,0.13306,0.16846,0.16113,0.089111,0.13672,0.081787,0.040283,0.05249,0.1123,0.05249,0.1001,0.10132,0.05249,0.085449,0.019531,-0.026855,-0.023193,0.041504,-0.013428,0.059814,0.089111,0.080566,0.031738,0.084229,0.025635,-0.010986,0.024414,0.072021,0.030518,0.084229,0.095215,0.074463,0.0012207,0.032959,-0.019531,-0.019531,0.029297,-0.01709,0.048828,0.073242,0.064697,0.025635,0.058594,0.0024414,-0.021973,0.019531,0.06958,0.031738,0.10742,0.12451,0.097656,0.037842,0.089111,0.046387,0.042725,0.091553,0.15625,0.12451,0.16357,0.15381,0.11108,0.15747,0.1001,0.073242,0.091553,0.15259,0.11108,0.17334,0.18799,0.16602,0.10132,0.14648,0.081787,0.078125,0.11719,0.17456,0.1416,0.1709,0.15503,0.10498,0.14038,0.056152,-0.0036621,0.010986,0.032959,-0.025635,0.048828,0.05127,0.021973,-0.058594,-0.0012207,-0.064697,-0.092773,-0.042725,-0.093994,-0.039063,0.024414,0.0024414,-0.054932,-0.11108,-0.080566,-0.14404,-0.14038,-0.11353,-0.17334,-0.11841,-0.08667,-0.11475,-0.15991,-0.085449,-0.13306,-0.13672,-0.078125,-0.11719,-0.03418,0.040283,0.05127,0.012207,0.059814,-0.0036621,-0.040283,-0.028076,0.01709,-0.054932,-0.0085449,0.0061035,-0.040283,-0.10986,-0.067139,-0.11719,-0.13794,-0.084229,-0.11353,-0.061035,-0.0012207,0.015869,-0.021973,-0.061035,0.0036621,-0.058594,-0.0354,0.025635,-0.018311,0.065918,0.072021,0.05249,-0.021973,0.01709,-0.061035,-0.093994,-0.065918,-0.1062,-0.045166,0.028076,0.0354,-0.0012207,-0.043945,0.014648,-0.021973,-0.0073242,0.040283,-0.0061035,0.068359,0.091553,0.087891,0.037842,0.095215,0.059814,0.056152,0.096436,0.17578,0.14648,0.2124,0.23315,0.22095,0.17456,0.21118,0.177,0.18188,0.22949,0.19531,0.25391,0.29175,0.2771,0.22461,0.26001,0.20264,0.15625,0.17822,0.23682,0.19775,0.24902,0.2417,0.20264,0.13306,0.16113,0.11597,0.097656,0.12085,0.06958,0.13916,0.15015,0.13306,0.070801,0.10742,0.048828,0.013428,0.041504,0.093994,0.048828,0.11108,0.13428,0.10986,0.046387,0.078125,0.020752,0.012207,0.056152,0.0036621,0.059814,0.098877,0.083008,0.031738,0.075684,0.021973,-0.0097656,0.045166,0.097656,0.064697,0.13428,0.15259,0.12695,0.068359,0.10376,0.031738,0.015869,0.048828,0.10376,0.054932,0.10498,0.089111,0.037842,0.090332,0.041504,0.0024414,0.03418,0.097656,0.048828,0.12695,0.15259,0.12695,0.068359,0.11597,0.058594,0.040283,0.084229,0.13794,0.091553,0.13672,0.14038,0.079346,0.12329,0.056152,0.0061035,0.023193,0.084229,0.041504,0.11353,0.15991,0.14038,0.079346,0.11841,0.070801,0.050049,0.093994,0.05249,0.10986,0.14648,0.15381,0.087891,0.11841,0.061035,0.0048828,0.0048828,0.054932,0.0012207,0.062256,0.074463,0.061035,-0.0048828,0.043945,-0.0085449,-0.028076,0.0036621,0.072021,0.029297,0.081787,0.087891,0.036621,0.081787,0.014648,-0.025635,-0.0061035,0.05249,0.018311,0.10132,0.13184,0.11597,0.068359,0.12085,0.072021,0.05249,0.072021,0.13184,0.084229,0.12329,0.13306,0.091553,0.0073242,0.036621,-0.012207,-0.0061035,0.037842,-0.013428,0.054932,0.085449,0.093994,0.048828,0.093994,0.036621,0.014648,0.050049,0.10742,0.058594,0.10742,0.10498,0.068359,0.019531,0.053711,0.012207,0.023193,0.079346,0.14282,0.12451,0.14648,0.13428,0.087891,0.13428,0.084229,0.062256,0.081787,0.14282,0.11841,0.17578,0.18921,0.14893,0.078125,0.10742,0.05249,0.045166,0.091553,0.031738,0.092773,0.12085,0.096436,0.046387,0.081787,0.01709,-0.019531,0.023193,0.068359,0.020752,0.10132,0.11963,0.089111,0.036621,0.080566,0.01709,0.01709,0.06958,0.12939,0.087891,0.1355,0.11963,0.070801,0.11475,0.045166,0.010986,0.048828,0.10742,0.070801,0.13672,0.14893,0.11597,0.056152,0.10498,0.042725,0.032959,0.078125,0.13306,0.093994,0.13062,0.11475,0.056152,0.095215,0.029297,-0.01709,0.01709,0.075684,0.026855,0.091553,0.13306,0.10132,0.032959,0.079346,0.025635,-0.0012207,0.043945,0.083008,0.019531,0.05249,0.053711,-0.0012207,-0.046387,0.0097656,-0.029297,0.0085449,0.064697,0.015869,0.093994,0.11108,0.095215,0.046387,0.087891,0.028076,0.0012207,0.042725,0.11353,0.070801,0.1123,0.11475,0.058594,0.089111,0.030518,-0.0085449,0,0.043945,0.0024414,0.062256,0.085449,0.068359,0.0097656,0.046387,0,-0.018311,0.018311,0.074463,0.0354,0.091553,0.093994,0.056152,-0.0024414,0.050049,0.0036621,0.019531,0.070801,0.029297,0.087891,0.12451,0.11108,0.056152,0.1001,0.05249,0.0354,0.078125,0.15259,0.11475,0.15625,0.16235,0.11963,0.064697,0.096436,0.037842,0.048828,0.083008,0.0354,0.11719,0.11841,0.091553,0.046387,0.079346,0.0085449,-0.021973,0,0.045166,0.0012207,0.063477,0.068359,0.026855,0.073242,0.0097656,-0.029297,-0.0024414,0.05249,0.0073242,0.068359,0.10376,0.075684,0.0073242,0.0354,-0.0354,-0.079346,-0.061035,-0.031738,-0.10376,-0.056152,-0.065918,-0.10986,-0.17456,-0.13672,-0.18677,-0.16113,-0.090332,-0.12451,-0.032959,0.023193,0.0097656,-0.0097656,-0.048828,0.0024414,-0.0354,0.0097656,0.05249,-0.0061035,0.045166,0.030518,-0.043945,-0.12451,-0.087891,-0.15259,-0.15381,-0.081787,-0.10132,-0.015869,0.05127,0.062256,0.036621,0.10864,0.068359,0.040283,0.080566,0.14648,0.1001,0.16113,0.18921,0.15625,0.092773,0.13672,0.062256,0.056152,0.11841,0.065918,0.12329,0.15625,0.15259,0.10132,0.14648,0.084229,0.029297,0.041504,0.10132,0.062256,0.11353,0.1355,0.11597,0.048828,0.093994,0.031738,0.0073242,0.032959,0.092773,0.042725,0.074463,0.06958,0.015869,0.0354,-0.01709,-0.040283,-0.025635,0.0354,-0.0085449,0.058594,0.081787,0.062256,-0.0061035,0.05127,-0.0085449,-0.028076,0.0061035,0.064697,0.023193,0.064697,0.078125,0.041504,0.087891,0.037842,-0.0073242,0.013428,0.053711,0.020752,0.061035,0.091553,0.061035,0.0097656,0.045166,0,-0.013428,0.03418,0.096436,0.06958,0.11841,0.12329,0.093994,0.023193,0.062256,0.020752,0.030518,0.062256,0.014648,0.076904,0.087891,0.056152,0.0012207,0.030518,-0.03418,-0.047607,-0.015869,0.056152,0.010986,0.06958,0.068359,0.0354,-0.020752,0.0354,-0.0085449,0.029297,0.074463,0.032959,0.11108,0.15015,0.1355,0.097656,0.15869,0.10132,0.080566,0.12207,0.17578,0.1355,0.21484,0.21851,0.18311,0.12695,0.15503,0.090332,0.093994,0.14893,0.085449,0.14404,0.17456,0.14038,0.081787,0.11841,0.05127,0.01709,0.048828,0.097656,0.05127,0.10742,0.12695,0.10132,0.036621,0.089111,0.032959,0.020752,0.079346,0.025635,0.096436,0.13428,0.12085,0.062256,0.11719,0.056152,0.020752,0.062256,0.12451,0.072021,0.13672,0.17212,0.13184,0.063477,0.11963,0.059814,0.046387,0.11108,0.058594,0.11475,0.14893,0.13306,0.068359,0.10864,0.037842,0.0048828,0.036621,0.087891,0.040283,0.096436,0.11108,0.091553,0.024414,0.06958,0.01709,0.0024414,0.058594,0.032959,0.096436,0.14648,0.14648,0.090332,0.13428,0.087891,0.046387,0.068359,0.12329,0.080566,0.12695,0.13428,0.10742,0.030518,0.068359,0.021973,-0.0061035,0.036621,0.090332,0.045166,0.081787,0.079346,0.028076,0.062256,-0.0024414,-0.0354,-0.0097656,0.041504,-0.012207,0.053711,0.063477,0.042725,-0.030518,0.0097656,-0.057373,-0.085449,-0.040283,-0.080566,-0.0073242,0.036621,0.032959,0,0.05249,0.012207,-0.021973,0.019531,0.062256,0.036621,0.12085,0.1355,0.12085,0.063477,0.097656,0.025635,0.0036621,0.023193,0.079346,0.040283,0.096436,0.087891,0.046387,-0.010986,0.029297,-0.019531,0.013428,0.045166,-0.0024414,0.068359,0.075684,0.042725,-0.020752,0.025635,-0.05249,-0.068359,-0.041504,-0.097656,-0.036621,0.032959,0.031738,0.018311,-0.021973,0.025635,-0.013428,0.023193,0.059814,0.023193,0.10742,0.13184,0.12329,0.070801,0.11597,0.048828,0.01709,0.05127,0.10986,0.054932,0.11475,0.10498,0.042725,-0.028076,0.013428,-0.065918,-0.061035,-0.015869,-0.068359,0.0024414,0.015869,0.0097656,-0.050049,0.0061035,-0.063477,-0.087891,-0.048828,0.030518,-0.0061035,0.087891,0.11108,0.079346,0.023193,0.072021,0.029297,0.036621,0.098877,0.058594,0.11963,0.15747,0.14771,0.097656,0.14648,0.10864,0.072021,0.10498,0.17578,0.12939,0.17822,0.19775,0.15869,0.081787,0.12939,0.067139,0.048828,0.091553,0.15869,0.11597,0.16235,0.15259,0.098877,0.1355,0.087891,0.064697,0.093994,0.14648,0.1123,0.16602,0.177,0.14038,0.061035,0.092773,0.042725,0.020752,0.050049,0.11597,0.072021,0.11597,0.11841,0.085449,0.11841,0.058594,0.024414,0.032959,0.085449,0.040283,0.087891,0.096436,0.074463,-0.0048828,0.030518,-0.041504,-0.059814,-0.024414,-0.064697,0.0073242,0.042725,0.041504,0.0097656,0.061035,0.014648,-0.0097656,0.023193,0.068359,0.032959,0.1123,0.12451,0.10132,0.0354,0.079346,0.013428,-0.0097656,0.023193,0.073242,0.032959,0.095215,0.087891,0.042725,0.091553,0.025635,0.0036621,0.032959,0.084229,0.042725,0.10864,0.13062,0.10864,0.062256,0.097656,0.037842,0.023193,0.063477,0.11597,0.073242,0.12939,0.12085,0.075684,0.029297,0.061035,0.018311,0.042725,0.098877,0.062256,0.13672,0.18311,0.15869,0.10742,0.15625,0.089111,0.050049,0.075684,0.12817,0.063477,0.098877,0.083008,0.01709,0.072021,0.0073242,-0.045166,-0.013428,0.050049,-0.0085449,0.079346,0.097656,0.062256,-0.0012207,-0.039063,0.0097656,-0.042725,0.0048828,0.061035,0.0012207,0.06958,0.091553,0.032959,0.087891,0.029297,-0.0097656,0.018311,0.073242,0.042725,0.10498,0.14038,0.12451,0.068359,0.11475,0.062256,0.025635,0.048828,0.11353,0.05127,0.10376,0.10742,0.06958,0.0036621,0.05249,0.0085449,0.013428,0.078125,0.056152,0.12085,0.15869,0.14282,0.073242,0.1062,0.050049,0.0024414,0.0048828,0.045166,-0.023193,0.021973,0.01709,-0.013428,-0.074463,-0.014648,-0.048828,-0.041504,0.014648,0.084229,0.054932,0.087891,0.092773,0.046387,0.078125,0.013428,-0.018311,0.0097656,0.059814,0.012207,0.062256,0.073242,0.040283,-0.046387,0.0012207,-0.057373,-0.061035,-0.0073242,-0.048828,0.019531,0.036621,0.040283,0.0061035,0.048828,-0.0024414,-0.028076,-0.018311,0.029297,-0.028076,0.041504,0.030518,0.010986,-0.054932,0.0061035,-0.043945,-0.025635,0.023193,0.08667,0.070801,0.14282,0.14282,0.093994,0.1355,0.070801,0.013428,0.0354,0.059814,-0.015869,0.029297,-0.0061035,-0.054932,-0.14648,-0.13306,-0.18921,-0.17944,-0.1062,-0.11475,-0.0036621,0.061035,0.090332,0.068359,0.032959,0.087891,0.053711,0.081787,0.14282,0.1001,0.16724,0.18921,0.15381,0.097656,0.12451,0.050049,0.0036621,0.031738,0.078125,0.028076,0.070801,0.075684,0.010986,0.05249,0.0036621,-0.061035,-0.03418,0.028076,-0.026855,0.062256,0.087891,0.059814,-0.0097656,0.037842,-0.040283,-0.067139,-0.041504,0.021973,-0.0354,0.040283,0.048828,0.0012207,0.057373,0.01709,-0.0354,-0.0097656,0.059814,-0.0036621,0.057373,0.068359,0.046387,-0.025635,0.023193,-0.037842,-0.058594,-0.015869,-0.0354,0.031738,0.091553,0.11841,0.081787,0.12329,0.076904,0.031738,0.043945,0.10498,0.074463,0.14404,0.18799,0.17822,0.12085,0.16968,0.11963,0.087891,0.10498,0.16235,0.1062,0.14404,0.15381,0.10376,0.13916,0.084229,0.039063,0.037842,0.083008,0.039063,0.11597,0.14648,0.14038,0.075684,0.10864,0.040283,0.0048828,0.023193,0.078125,0.037842,0.096436,0.11108,0.085449,0.0097656,0.025635,-0.031738,-0.03418,0.0048828,-0.064697,0.0024414,0.010986,-0.014648,-0.05249,-0.12695,-0.092773,-0.1355,-0.12207,-0.064697,-0.096436,-0.026855,0.0024414,-0.021973,-0.053711,-0.0036621,-0.061035,-0.073242,-0.046387,-0.1123,-0.067139,-0.062256,-0.12207,-0.20996,-0.22461,-0.32837,-0.39917,-0.4126,-0.39063,-0.43579,-0.38452,-0.3772,-0.40161,-0.46143,-0.40649,-0.448,-0.43579,-0.36987,-0.30029,-0.31616,-0.24048,-0.21118,-0.21606,-0.2417,-0.16602,-0.17822,-0.11841,-0.023193,-0.048828,0.041504,0.083008,0.070801,0.024414,0.081787,0.031738,0.012207,0.058594,0.11963,0.064697,0.10864,0.10376,0.048828,0.0048828,0.065918,0.015869,0.05249,0.12085,0.091553,0.18433,0.22461,0.20142,0.14038,0.19653,0.14038,0.11108,0.16602,0.22949,0.18188,0.24658,0.25635,0.19409,0.23682,0.17822,0.13672,0.14771,0.19897,0.15259,0.20996,0.23682,0.22827,0.16724,0.20142,0.15747,0.11597,0.14648,0.22583,0.1709,0.21729,0.22461,0.17822,0.10742,0.15381,0.096436,0.081787,0.13428,0.084229,0.12695,0.16479,0.16357,0.10742,0.16235,0.12695,0.1001,0.14282,0.12817,0.19043,0.25024,0.26123,0.20874,0.1416,0.18311,0.13184,0.13062,0.17334,0.13062,0.20874,0.23193,0.22705,0.16724,0.2124,0.16113,0.14771,0.17822,0.23438,0.19897,0.25391,0.26367,0.22705,0.15015,0.16968,0.12085,0.11108,0.14038,0.080566,0.12939,0.13916,0.11597,0.054932,0.081787,0.01709,-0.019531,0.0024414,0.048828,0,0.057373,0.072021,0.056152,0.010986,0.061035,0.018311,0.03418,0.097656,0.16968,0.16724,0.22461,0.21851,0.17822,0.23682,0.177,0.1416,0.16846,0.21973,0.1709,0.22705,0.22461,0.16846,0.091553,0.10986,0.042725,0.029297,0.061035,0.10376,0.062256,0.091553,0.070801,0.029297,0.074463,0.0097656,-0.031738,0,0.032959,-0.024414,0.029297,0.021973,-0.057373,-0.13916,-0.14282,-0.2478,-0.27466,-0.20142,-0.23438,-0.12329,-0.045166,-0.029297,-0.067139,-0.11108,-0.040283,-0.061035,-0.026855,0.05127,0.013428,0.1001,0.14404,0.12329,0.065918,0.12695,0.068359,0.053711,0.089111,0.14648,0.10376,0.15625,0.15503,0.1001,0.14893,0.087891,0.053711,0.080566,0.15503,0.11841,0.177,0.21484,0.19897,0.12329,0.17944,0.11353,0.068359,0.081787,0.12939,0.053711,0.063477,0.05249,-0.025635,-0.11841,-0.1001,-0.16846,-0.18066,-0.11353,-0.14404,-0.080566,-0.031738,-0.040283,-0.087891,-0.029297,-0.054932,-0.070801,-0.023193,-0.048828,0.010986,0.058594,0.065918,0.029297,0.068359,0.018311,-0.015869,0.013428,0.080566,0.072021,0.16235,0.20142,0.2124,0.16846,0.21118,0.16968,0.1416,0.15625,0.20752,0.15259,0.19043,0.19775,0.15869,0.087891,0.12695,0.084229,0.090332,0.1355,0.097656,0.16357,0.19653,0.17334,0.10498,0.12939,0.067139,0.029297,0.037842,0.087891,0.040283,0.096436,0.10376,0.076904,0.01709,0.061035,0.012207,0.019531,0.072021,0.03418,0.10742,0.14404,0.14038,0.1001,0.15625,0.1001,0.093994,0.15015,0.2124,0.17456,0.24048,0.24414,0.21362,0.15259,0.2002,0.14038,0.13062,0.17822,0.11963,0.20142,0.22827,0.20874,0.15747,0.20142,0.13184,0.095215,0.11475,0.15747,0.11108,0.16846,0.17822,0.14038,0.06958,0.10132,0.041504,0.030518,0.075684,0.12451,0.073242,0.10254,0.075684,0.01709,0.062256,0.0036621,-0.029297,0.025635,0.081787,0.041504,0.11597,0.14038,0.11719,0.061035,0.11963,0.062256,0.040283,0.10376,0.064697,0.11841,0.17578,0.17212,0.10864,0.14404,0.087891,0.0354,0.046387,0.10376,0.040283,0.083008,0.098877,0.056152,-0.023193,0.029297,-0.045166,-0.068359,-0.029297,-0.075684,-0.018311,0.026855,0.026855,-0.0354,-0.084229,-0.021973,-0.059814,-0.042725,0.030518,-0.015869,0.062256,0.10132,0.091553,0.029297,0.079346,0.046387,0.0354,0.085449,0.16846,0.13916,0.18555,0.19409,0.14893,0.18555,0.12329,0.091553,0.10498,0.15015,0.10986,0.17334,0.18555,0.17822,0.12085,0.14771,0.1001,0.074463,0.10132,0.17212,0.11597,0.15259,0.14526,0.087891,0.12939,0.070801,0.037842,0.061035,0.11841,0.087891,0.15015,0.17944,0.16602,0.11475,0.15381,0.1001,0.068359,0.087891,0.13306,0.089111,0.14282,0.13062,0.081787,0.020752,0.048828,0.0048828,0.021973,0.065918,0.036621,0.11597,0.15015,0.13672,0.081787,0.12451,0.068359,0.037842,0.06958,0.13062,0.078125,0.12695,0.13184,0.096436,0.036621,0.070801,0.020752,0.03418,0.081787,0.037842,0.12939,0.15381,0.14404,0.096436,0.14038,0.074463,0.032959,0.06958,0.11353,0.054932,0.12817,0.14526,0.098877,0.042725,0.078125,0.025635,0.030518,0.085449,0.026855,0.090332,0.11597,0.095215,0.043945,0.087891,0.024414,-0.0012207,0.010986,0.05249,0.018311,0.075684,0.092773,0.068359,0.012207,0.059814,0.0073242,0.0061035,0.064697,0.13428,0.087891,0.13306,0.12451,0.062256,0.090332,0.030518,-0.025635,-0.0012207,-0.054932,-0.013428,0.048828,0.068359,0.039063,-0.023193,0.042725,-0.015869,-0.019531,0.031738,-0.023193,0.05249,0.081787,0.078125,0.018311,0.056152,-0.025635,-0.080566,-0.084229,-0.041504,-0.095215,-0.058594,-0.043945,-0.081787,-0.15381,-0.11108,-0.16846,-0.19165,-0.13916,-0.16846,-0.075684,-0.015869,-0.0097656,-0.048828,-0.12329,-0.076904,-0.12695,-0.13916,-0.10498,-0.16235,-0.12573,-0.13062,-0.16602,-0.25513,-0.22705,-0.28564,-0.29907,-0.25146,-0.2771,-0.19165,-0.11353,-0.078125,-0.079346,-0.11475,-0.041504,-0.062256,-0.020752,0.046387,-0.0024414,0.058594,0.061035,0.024414,-0.0354,0.023193,-0.026855,-0.019531,0.030518,0.10132,0.081787,0.16113,0.16846,0.14038,0.093994,0.13428,0.096436,0.1123,0.15015,0.11597,0.17334,0.19287,0.16968,0.1001,0.12451,0.064697,0.037842,0.06958,0.12817,0.091553,0.16235,0.17212,0.13794,0.089111,0.12695,0.087891,0.11108,0.15869,0.1123,0.17822,0.2063,0.18921,0.13916,0.177,0.10254,0.061035,0.075684,0.12085,0.043945,0.073242,0.050049,-0.018311,-0.090332,-0.070801,-0.14404,-0.14648,-0.11841,-0.15503,-0.083008,-0.061035,-0.070801,-0.11597,-0.054932,-0.095215,-0.11597,-0.067139,-0.097656,-0.026855,0.057373,0.084229,0.065918,0.0097656,0.062256,0.0073242,-0.0048828,0.062256,0.0085449,0.05249,0.091553,0.068359,-0.01709,0.018311,-0.054932,-0.10132,-0.090332,-0.0354,-0.091553,-0.03418,-0.0097656,-0.036621,-0.087891,-0.0354,-0.068359,-0.064697,-0.0061035,-0.037842,0.0354,0.068359,0.065918,0.014648,0.058594,0.0048828,-0.03418,-0.013428,0.050049,-0.018311,0.042725,0.056152,0.021973,-0.05249,-0.014648,-0.067139,-0.1001,-0.058594,-0.10132,-0.040283,-0.0036621,0.0048828,-0.026855,0.039063,-0.0012207,-0.0012207,0.029297,0.095215,0.072021,0.1416,0.14771,0.12817,0.053711,0.08667,0.01709,-0.012207,0.023193,0.070801,0.010986,0.048828,0.029297,-0.025635,-0.078125,-0.026855,-0.061035,-0.029297,0.032959,-0.0073242,0.076904,0.10132,0.11597,0.093994,0.16113,0.1355,0.14282,0.19531,0.27344,0.26367,0.31738,0.31494,0.27954,0.21973,0.25024,0.20752,0.21851,0.26733,0.22217,0.2832,0.29297,0.25269,0.19287,0.22705,0.15991,0.1416,0.16846,0.22217,0.17456,0.22095,0.21362,0.16357,0.098877,0.12817,0.081787,0.10254,0.1416,0.1001,0.17334,0.19287,0.18188,0.14893,0.18555,0.12451,0.10498,0.14648,0.20996,0.17578,0.2478,0.24902,0.19409,0.13916,0.17456,0.10498,0.10742,0.14648,0.074463,0.12817,0.14282,0.10132,0.041504,0.087891,0.024414,-0.0073242,0.020752,0.059814,0.0097656,0.062256,0.072021,0.023193,-0.029297,0.030518,-0.028076,-0.024414,0.030518,-0.036621,0.020752,0.050049,0.019531,-0.064697,-0.14404,-0.12695,-0.19775,-0.18433,-0.24048,-0.21118,-0.18188,-0.18188,-0.22705,-0.29053,-0.22705,-0.26367,-0.24048,-0.14404,-0.15015,-0.031738,0.026855,0.045166,0.0061035,0.059814,0.021973,0.0061035,0.029297,0.10742,0.068359,0.1123,0.13916,0.11108,0.032959,0.072021,0.025635,0.013428,0.076904,0.045166,0.1123,0.14648,0.14282,0.090332,0.12451,0.074463,0.031738,0.045166,0.11108,0.062256,0.12817,0.15015,0.12085,0.05249,0.097656,0.053711,0.05249,0.093994,0.16113,0.12207,0.15259,0.1416,0.080566,0.10254,0.032959,-0.015869,-0.0048828,0.021973,-0.043945,0.010986,0.013428,-0.015869,-0.05127,0.0073242,-0.046387,-0.045166,0.0036621,0.058594,0.030518,0.076904,0.078125,0.05249,0.1123,0.053711,0.026855,0.059814,0.12085,0.1001,0.17334,0.20752,0.18921,0.14648,0.21362,0.16235,0.16235,0.21484,0.27588,0.25146,0.30029,0.29053,0.24292,0.29419,0.22217,0.17578,0.19409,0.22705,0.16846,0.22705,0.22949,0.18677,0.1062,0.14648,0.079346,0.057373,0.1001,0.16602,0.11841,0.18799,0.20264,0.16602,0.23071,0.17578,0.13062,0.15381,0.21484,0.16968,0.22461,0.25024,0.22095,0.16602,0.21606,0.15137,0.11475,0.15015,0.21118,0.15381,0.20752,0.20508,0.15015,0.19043,0.11841,0.054932,0.056152,0.1001,0.045166,0.093994,0.12329,0.090332,0.019531,0.046387,0.0024414,-0.041504,0.0073242,0.072021,0.024414,0.080566,0.10132,0.062256,0.0012207,0.058594,0.018311,0.03418,0.092773,0.062256,0.13794,0.16724,0.15991,0.091553,0.13794,0.076904,0.032959,0.05127,0.11108,0.05249,0.097656,0.10498,0.059814,0.087891,0.021973,-0.040283,-0.05249,-0.012207,-0.05249,-0.0012207,0.019531,0.012207,-0.054932,-0.013428,-0.059814,-0.08667,-0.076904,-0.0036621,-0.053711,0.0073242,0.0097656,-0.0085449,-0.059814,-0.015869,-0.057373,-0.059814,-0.0048828,-0.043945,0.026855,0.065918,0.061035,0.012207,0.048828,-0.0085449,-0.032959,-0.012207,0.042725,-0.0097656,0.048828,0.061035,0.036621,-0.015869,0.040283,0.0073242,0.0036621,0.046387,0.10986,0.095215,0.12817,0.13306,0.11475,0.16602,0.11108,0.092773,0.13062,0.18555,0.15503,0.21973,0.23071,0.19165,0.12451,0.15381,0.091553,0.075684,0.11475,0.05249,0.10498,0.13062,0.11353,0.053711,0.093994,0.025635,-0.023193,0.010986,0.048828,0,0.081787,0.10864,0.087891,0.050049,0.10742,0.05127,0.053711,0.090332,0.1355,0.097656,0.14893,0.1355,0.080566,0.12939,0.061035,0.013428,0.041504,0.097656,0.036621,0.093994,0.11475,0.074463,0.0097656,0.058594,0.0024414,-0.021973,0.025635,0.06958,0.020752,0.057373,0.045166,-0.0036621,-0.05127,0.0061035,-0.046387,-0.0073242,0.058594,0.01709,0.089111,0.14038,0.12451,0.074463,0.12329,0.078125,0.05127,0.096436,0.15625,0.11108,0.15625,0.15869,0.096436,0.1355,0.075684,0.029297,0.032959,0.085449,0.042725,0.10986,0.13184,0.12695,0.062256,0.097656,0.058594,0.029297,0.050049,0.12695,0.090332,0.14404,0.15625,0.11597,0.15015,0.092773,0.037842,0.021973,0.065918,0.014648,0.053711,0.054932,0.030518,-0.050049,-0.015869,-0.065918,-0.10376,-0.064697,-0.084229,-0.0061035,0.065918,0.087891,0.070801,0.021973,0.065918,0.025635,0.023193,0.068359,0.031738,0.084229,0.10864,0.093994,0.031738,0.072021,0.012207,-0.013428,0.028076,0.074463,0.041504,0.10864,0.11841,0.095215,0.041504,0.083008,0.045166,0.048828,0.087891,0.14893,0.11353,0.15015,0.13306,0.074463,0.11475,0.048828,0.014648,0.031738,0.079346,0.050049,0.10742,0.13794,0.12085,0.06958,0.11963,0.078125,0.068359,0.12085,0.068359,0.1355,0.16357,0.14648,0.095215,0.13916,0.079346,0.0354,0.065918,0.12207,0.070801,0.1416,0.15625,0.12695,0.072021,0.12085,0.059814,0.041504,0.074463,0.11597,0.059814,0.10864,0.083008,0.023193,0.056152,-0.015869,-0.047607,-0.025635,0.020752,-0.030518,0.043945,0.061035,0.031738,-0.028076,0.037842,-0.026855,-0.024414,0.021973,0.064697,0.01709,0.061035,0.058594,0.013428,0.064697,0.0073242,-0.028076,0,0.050049,-0.0012207,0.068359,0.081787,0.05127,-0.015869,0.031738,-0.054932,-0.083008,-0.06958,-0.11841,-0.081787,-0.036621,-0.0354,-0.079346,-0.12939,-0.068359,-0.10986,-0.090332,-0.045166,-0.090332,-0.021973,-0.015869,-0.015869,-0.084229,-0.040283,-0.076904,-0.10132,-0.058594,-0.081787,-0.020752,0.045166,0.062256,0.0097656,0.05249,0.012207,-0.020752,0.010986,0.068359,0.037842,0.11108,0.14038,0.12695,0.068359,0.11841,0.080566,0.048828,0.080566,0.15381,0.10742,0.16724,0.16724,0.12329,0.063477,0.098877,0.036621,0.031738,0.059814,-0.013428,0.042725,0.029297,-0.0085449,-0.064697,-0.041504,-0.097656,-0.1062,-0.068359,-0.091553,-0.0024414,0.058594,0.06958,0.042725,-0.01709,0.031738,-0.020752,-0.0073242,0.0354,-0.023193,0.061035,0.068359,0.050049,0.0048828,0.039063,-0.021973,-0.041504,-0.023193,0.032959,-0.020752,0.054932,0.058594,0.032959,-0.018311,0.042725,-0.015869,0.0024414,0.050049,0.0061035,0.070801,0.10742,0.092773,0.036621,0.078125,0.021973,-0.015869,0.032959,0.076904,0.032959,0.098877,0.10986,0.078125,0.026855,0.06958,0.012207,0.010986,0.043945,0.084229,0.048828,0.090332,0.062256,0.025635,0.075684,0.025635,-0.0073242,0.031738,0.074463,0.026855,0.096436,0.11108,0.064697,-0.0097656,0.040283,-0.036621,-0.0354,0.014648,0.065918,0.045166,0.10376,0.10498,0.072021,0.12085,0.068359,0.030518,0.050049,0.11841,0.074463,0.13916,0.16968,0.14771,0.090332,0.15015,0.098877,0.091553,0.15015,0.22217,0.19653,0.24048,0.2478,0.20752,0.25391,0.20508,0.16235,0.17578,0.22827,0.17944,0.23438,0.24414,0.20874,0.11963,0.14648,0.074463,0.023193,0.041504,0.093994,0.042725,0.085449,0.091553,0.050049,0.084229,0.03418,-0.01709,-0.0061035,0.050049,-0.024414,0.029297,0.029297,0.0024414,-0.064697,-0.014648,-0.067139,-0.091553,-0.041504,-0.075684,-0.0012207,0.068359,0.074463,0.039063,0.08667,0.029297,-0.015869,0.0097656,0.03418,-0.0024414,0.062256,0.078125,0.065918,0.0012207,0.037842,-0.013428,-0.025635,-0.0036621,-0.054932,0.0012207,0.029297,0.029297,-0.020752,-0.078125,-0.040283,-0.087891,-0.085449,-0.0354,-0.076904,0,0.030518,0.037842,0.012207,0.080566,0.040283,0.037842,0.090332,0.15625,0.11475,0.18066,0.17944,0.14038,0.074463,0.11597,0.059814,0.070801,0.097656,0.041504,0.096436,0.11597,0.097656,0.054932,0.10742,0.048828,0.028076,0.058594,0.11597,0.075684,0.14282,0.15381,0.11963,0.053711,0.093994,0.039063,0.031738,0.074463,0.032959,0.084229,0.12329,0.10864,0.059814,0.10864,0.063477,0.0354,0.064697,0.13062,0.087891,0.1416,0.14648,0.10498,0.0354,0.084229,0.0354,0.031738,0.085449,0.14648,0.11597,0.15747,0.15381,0.093994,0.1416,0.087891,0.048828,0.078125,0.12695,0.070801,0.12085,0.13672,0.098877,0.031738,0.070801,0.029297,0.013428,0.075684,0.040283,0.10742,0.14771,0.16113,0.11597,0.15991,0.10864,0.062256,0.059814,0.11475,0.056152,0.095215,0.093994,0.056152,-0.031738,0.021973,-0.046387,-0.059814,-0.015869,-0.05249,0.01709,0.075684,0.096436,0.065918,0.12207,0.096436,0.062256,0.097656,0.15747,0.11475,0.17334,0.19653,0.17334,0.10864,0.14771,0.098877,0.078125,0.11108,0.1709,0.13916,0.18188,0.17334,0.13672,0.16846,0.10132,0.061035,0.070801,0.10742,0.062256,0.11353,0.12207,0.096436,0.048828,0.074463,0.023193,0.012207,0.031738,0.097656,0.062256,0.087891,0.087891,0.05249,0.090332,0.0354,-0.0061035,0.014648,0.042725,-0.0024414,0.070801,0.072021,0.058594,0.0097656,0.045166,-0.019531,-0.031738,0.0024414,0.043945,-0.0048828,0.05127,0.031738,-0.020752,-0.065918,-0.032959,-0.093994,-0.085449,-0.056152,-0.12329,-0.065918,-0.058594,-0.090332,-0.14282,-0.10864,-0.16357,-0.19165,-0.16235,-0.10132,-0.14038,-0.064697,-0.042725,-0.057373,-0.1062,-0.061035,-0.11841,-0.11353,-0.067139,-0.12695,-0.068359,-0.025635,-0.042725,-0.087891,-0.040283,-0.096436,-0.12207,-0.059814,0.032959,-0.0024414,0.089111,0.11597,0.096436,0.059814,0.12085,0.085449,0.098877,0.15991,0.12451,0.19409,0.23193,0.21729,0.16968,0.21362,0.15259,0.11108,0.12695,0.17578,0.12085,0.16602,0.18555,0.15015,0.070801,0.12573,0.091553,0.10132,0.18555,0.14893,0.20752,0.2478,0.23315,0.18555,0.22461,0.17212,0.13428,0.14771,0.20996,0.16235,0.21606,0.2478,0.21973,0.15625,0.20508,0.14648,0.13306,0.17944,0.1355,0.19653,0.23193,0.21973,0.16968,0.19653,0.12939,0.089111,0.10132,0.14038,0.093994,0.1416,0.13672,0.097656,0.029297,0.059814,0.018311,0.0012207,0.040283,0.11963,0.089111,0.12695,0.12695,0.080566,0.11597,0.05249,0.024414,0.032959,0.085449,0.054932,0.11597,0.14404,0.1355,0.079346,0.12695,0.087891,0.074463,0.11475,0.065918,0.12817,0.16846,0.15015,0.1001,0.13062,0.054932,0.0061035,0.013428,0.050049,-0.0048828,0.062256,0.057373,0.048828,-0.015869,0.043945,-0.029297,-0.05127,-0.026855,-0.095215,-0.053711,-0.031738,-0.065918,-0.16479,-0.29663,-0.35034,-0.51514,-0.65552,-0.82153,-1.0876,-1.344,-1.6919,-1.7419,-1.7407,-1.7456,-1.7493,-1.748,-1.7493,-1.7395,-1.7383,-1.7395,-1.7407,-1.7358,-1.7444,-1.0071,-0.80322,-0.58228,-0.36133,-0.25024,-0.063477,0.081787,0.15625,0.18921,0.29663,0.28809,0.29297,0.34546,0.30273,0.35034,0.40527,0.40649,0.37476,0.32471,0.36499,0.32227,0.32837,0.37231,0.33569,0.40405,0.42725,0.41748,0.36865,0.4126,0.36133,0.32227,0.32959,0.38086,0.3125,0.36377,0.37476,0.33203,0.26611,0.31738,0.27466,0.2832,0.34668,0.32715,0.40039,0.44189,0.45044,0.42114,0.47852,0.45044,0.42603,0.4541,0.51514,0.46631,0.5188,0.5249,0.4895,0.4126,0.44678,0.37476,0.354,0.39307,0.34424,0.39185,0.42236,0.40771,0.34546,0.3772,0.32227,0.28076,0.30029,0.35034,0.3064,0.35278,0.35034,0.32227,0.25391,0.29053,0.24414,0.23438,0.26367,0.23438,0.31616,0.3479,0.34912,0.29907,0.32959,0.26367,0.22461,0.22705,0.26733,0.2124,0.26001,0.2771,0.25269,0.18311,0.21729,0.16235,0.15381,0.19287,0.14893,0.20874,0.23682,0.23438,0.18311,0.21606,0.15503,0.1123,0.12939,0.18921,0.15625,0.2356,0.25269,0.23438,0.177,0.21118,0.14771,0.12451,0.15625,0.20142,0.15137,0.19653,0.18555,0.13672,0.17944,0.11597,0.080566,0.10986,0.17212,0.12817,0.19775,0.22461,0.19897,0.14771,0.18799,0.13428,0.11353,0.13672,0.18921,0.14648,0.19287,0.17944,0.11963,0.15869,0.083008,0.031738,0.046387,0.090332,0.040283,0.10864,0.13672,0.098877,0.03418,0.065918,-0.0024414,-0.032959,0.0061035,0.048828,0.023193,0.085449,0.097656,0.068359,0.12329,0.078125,0.029297,0.042725,0.1001,0.062256,0.13794,0.1709,0.16724,0.11597,0.17334,0.11841,0.092773,0.12451,0.19409,0.14893,0.19775,0.2063,0.14648,0.17944,0.11597,0.05127,0.058594,0.091553,0.05127,0.10498,0.13672,0.11353,0.047607,0.087891,0.032959,-0.012207,0.023193,0.084229,0.024414,0.093994,0.11597,0.079346,0.0073242,0.05127,0.0061035,0.0073242,0.061035,0.021973,0.081787,0.10864,0.1001,0.042725,0.078125,0.020752,-0.018311,0.023193,0.06958,0.023193,0.067139,0.079346,0.048828,-0.013428,0.0354,-0.020752,-0.014648,0.040283,-0.0048828,0.085449,0.11475,0.12085,0.076904,0.13184,0.091553,0.06958,0.11597,0.17334,0.14038,0.20264,0.21729,0.18555,0.12451,0.16235,0.10254,0.098877,0.14038,0.090332,0.15991,0.20508,0.19165,0.15015,0.19165,0.13062,0.096436,0.12451,0.16968,0.12451,0.17944,0.18799,0.16235,0.091553,0.11597,0.057373,0.041504,0.074463,0.12695,0.079346,0.12207,0.11475,0.06958,0.12085,0.056152,0.025635,0.068359,0.1123,0.075684,0.14893,0.15259,0.11841,0.063477,0.10742,0.046387,0.03418,0.072021,0.1355,0.10132,0.14038,0.12939,0.079346,0.11597,0.056152,0.0097656,0.020752,0.080566,0.0354,0.095215,0.12939,0.10132,0.036621,0.081787,0.025635,0.014648,0.057373,0.12085,0.075684,0.12573,0.13428,0.076904,0.11475,0.061035,0.0012207,0.012207,0.061035,0,0.068359,0.072021,0.040283,-0.026855,0.013428,-0.064697,-0.090332,-0.057373,-0.097656,-0.040283,0.0073242,0.010986,-0.042725,-0.087891,-0.032959,-0.078125,-0.058594,0.012207,-0.023193,0.059814,0.089111,0.070801,0.0073242,0.042725,-0.0024414,-0.012207,0.029297,0.091553,0.05249,0.12695,0.14893,0.10986,0.042725,0.081787,0.036621,0.05127,0.090332,0.037842,0.091553,0.12329,0.10742,0.045166,0.075684,0.012207,-0.015869,0.023193,0.079346,0.045166,0.087891,0.098877,0.06958,0.0036621,0.041504,-0.0085449,0.0024414,0.030518,-0.0061035,0.072021,0.070801,0.041504,-0.018311,0.018311,-0.056152,-0.084229,-0.070801,0,-0.040283,0.023193,0.040283,0.0024414,-0.048828,0.023193,-0.019531,0,0.048828,0.0097656,0.091553,0.12207,0.10986,0.065918,0.096436,0.037842,0.010986,0.037842,0.097656,0.059814,0.12085,0.12939,0.10864,0.039063,0.073242,0.0061035,0.0024414,0.047607,0.093994,0.05127,0.085449,0.058594,0.010986,0.0354,-0.040283,-0.074463,-0.037842,0.026855,-0.019531,0.056152,0.072021,0.040283,-0.012207,0.050049,0.0061035,0.0097656,0.062256,0.13428,0.11108,0.15259,0.14771,0.10132,0.1416,0.072021,0.0354,0.056152,0.10742,0.042725,0.098877,0.12939,0.087891,0.024414,0.084229,0.029297,0.021973,0.091553,0.050049,0.12451,0.18555,0.19165,0.13916,0.17944,0.12085,0.081787,0.10254,0.15991,0.11597,0.18555,0.19775,0.17334,0.098877,0.13916,0.068359,0.046387,0.073242,0.11963,0.063477,0.1001,0.079346,0.01709,0.048828,-0.020752,-0.058594,-0.046387,0.018311,-0.028076,0.048828,0.081787,0.076904,0.023193,0.075684,0.036621,0.0354,0.072021,0.15503,0.11108,0.15625,0.16846,0.12817,0.16235,0.10132,0.062256,0.067139,0.10986,0.065918,0.11597,0.13428,0.11353,0.046387,0.068359,-0.0024414,-0.032959,-0.0012207,-0.048828,0.013428,0.046387,0.045166,0.021973,-0.045166,0.021973,-0.020752,-0.015869,0.048828,0.012207,0.084229,0.11108,0.10376,0.046387,0.090332,0.037842,0.020752,0.046387,0.11597,0.078125,0.13916,0.14893,0.10742,0.042725,0.085449,0.036621,0.041504,0.087891,0.032959,0.091553,0.11841,0.093994,0.032959,0.076904,0.019531,-0.0061035,0.037842,0.084229,0.042725,0.11841,0.12207,0.091553,0.0354,0.061035,0.012207,0.015869,0.062256,0.021973,0.095215,0.13428,0.11353,0.072021,0.12695,0.062256,0.037842,0.070801,0.12695,0.083008,0.1416,0.15259,0.10742,0.05127,0.093994,0.029297,0.021973,0.062256,0.11597,0.081787,0.10498,0.090332,0.046387,0.095215,0.040283,0.010986,0.047607,0.10742,0.058594,0.12207,0.14648,0.12573,0.068359,0.11108,0.056152,0.05127,0.10864,0.048828,0.10864,0.14404,0.12207,0.064697,0.095215,0.040283,-0.0061035,0.021973,0.085449,0.05249,0.12085,0.14648,0.11597,0.048828,0.10254,0.046387,0.023193,0.072021,0.031738,0.083008,0.12695,0.12817,0.079346,0.12329,0.080566,0.029297,0.042725,0.1001,0.05249,0.11475,0.13672,0.11475,0.041504,0.081787,0.029297,0.0048828,0.045166,0.10254,0.064697,0.11719,0.12451,0.079346,0.12085,0.068359,0.023193,0.032959,0.089111,0.045166,0.085449,0.10742,0.083008,0.010986,0.05249,-0.0012207,-0.029297,0.014648,0.075684,0.039063,0.081787,0.079346,0.0354,0.072021,0.0097656,-0.040283,-0.036621,0.020752,-0.0354,0.045166,0.05127,0.015869,-0.05127,-0.0061035,-0.067139,-0.092773,-0.058594,0.0097656,-0.03418,0.045166,0.058594,0.024414,0.080566,0.032959,0.0097656,0.037842,0.095215,0.061035,0.12817,0.15625,0.13916,0.091553,0.12085,0.068359,0.05249,0.081787,0.1416,0.083008,0.13428,0.12207,0.072021,0.0012207,0.01709,-0.040283,-0.037842,0.0012207,-0.05127,0.0024414,0.020752,0.0024414,-0.047607,0.01709,-0.031738,-0.048828,-0.0036621,-0.037842,0.0354,0.080566,0.097656,0.06958,0.1123,0.065918,0.025635,0.032959,0.090332,0.057373,0.13306,0.15991,0.14526,0.081787,0.12695,0.065918,0.032959,0.072021,0.12085,0.065918,0.12207,0.14038,0.093994,0.01709,0.059814,0.0024414,-0.0097656,0.065918,0.0097656,0.064697,0.098877,0.084229,0.032959,0.075684,0.0061035,-0.039063,-0.013428,0.031738,-0.029297,0.024414,0.014648,-0.025635,-0.083008,-0.03418,-0.079346,-0.084229,-0.015869,-0.05249,0.024414,0.075684,0.070801,0.013428,0.072021,0.020752,-0.0012207,0.043945,0.095215,0.058594,0.12695,0.14282,0.11108,0.048828,0.080566,0.026855,0.013428,0.05249,0.11597,0.079346,0.10254,0.096436,0.058594,0.076904,-0.0061035,-0.037842,-0.0354,0.0097656,-0.045166,0.0036621,-0.0073242,-0.036621,-0.10254,-0.062256,-0.10742,-0.11841,-0.062256,-0.091553,-0.012207,0.039063,0.0354,-0.0012207,-0.05249,0.0024414,-0.050049,-0.025635,0.030518,-0.020752,0.05127,0.05127,0.021973,-0.045166,0.012207,-0.048828,-0.046387,-0.0012207,0.062256,0.026855,0.087891,0.084229,0.050049,0.10376,0.032959,0.0012207,0.040283,0.092773,0.05127,0.12207,0.13916,0.11597,0.047607,0.081787,0.019531,-0.0061035,0.037842,0.087891,0.046387,0.078125,0.083008,0.0354,0.068359,-0.0061035,-0.043945,-0.041504,0.0024414,-0.05249,0.0024414,0.018311,-0.012207,-0.057373,-0.015869,-0.073242,-0.11841,-0.067139,-0.0024414,-0.037842,0.025635,0.026855,-0.01709,-0.059814,-0.0073242,-0.068359,-0.061035,-0.015869,-0.070801,0.0024414,0.025635,0.021973,-0.019531,0.046387,0.0061035,-0.0097656,0.048828,0.12329,0.089111,0.17212,0.19897,0.16968,0.12085,0.16602,0.11108,0.12939,0.18555,0.13428,0.19531,0.21729,0.19531,0.12207,0.16602,0.095215,0.050049,0.068359,0.11108,0.048828,0.080566,0.063477,0.0085449,-0.074463,-0.040283,-0.10376,-0.12329,-0.089111,-0.12939,-0.062256,-0.021973,-0.025635,-0.074463,-0.028076,-0.067139,-0.10254,-0.072021,0.0012207,-0.043945,0.029297,0.019531,-0.031738,-0.081787,-0.061035,-0.096436,-0.087891,-0.045166,-0.076904,0.0073242,0.031738,0.05249,0.015869,0.054932,0.0012207,-0.025635,0.013428,0.081787,0.057373,0.11841,0.15259,0.12939,0.063477,0.10376,0.05249,0.048828,0.11108,0.078125,0.14038,0.17456,0.15625,0.10864,0.14282,0.081787,0.030518,0.048828,0.087891,0.03418,0.090332,0.08667,0.046387,-0.029297,0.023193,-0.046387,-0.045166,-0.0012207,-0.05249,-0.0061035,0.029297,-0.0097656,-0.061035,-0.15259,-0.14282,-0.20386,-0.19775,-0.16602,-0.21362,-0.15015,-0.13672,-0.15259,-0.20264,-0.15503,-0.19287,-0.17822,-0.12085,-0.15381,-0.064697,-0.01709,-0.010986,-0.039063,-0.1001,-0.056152,-0.1062,-0.08667,-0.029297,-0.090332,-0.036621,-0.0097656,-0.031738,-0.091553,-0.032959,-0.080566,-0.096436,-0.039063,-0.067139,0.0012207,0.046387,0.036621,-0.026855,-0.098877,-0.074463,-0.12573,-0.11841,-0.070801,-0.10376,-0.019531,0.025635,0.026855,-0.015869,0.065918,0.026855,0.020752,0.075684,0.15259,0.10742,0.177,0.18433,0.1355,0.19531,0.14038,0.098877,0.11353,0.17456,0.13062,0.18921,0.22217,0.21606,0.15015,0.19775,0.14282,0.11353,0.14038,0.20874,0.17212,0.22705,0.24536,0.20264,0.13306,0.18066,0.12085,0.12695,0.17334,0.12817,0.19043,0.22827,0.21729,0.16724,0.21606,0.17944,0.15747,0.19897,0.27832,0.24658,0.30273,0.32227,0.2832,0.22583,0.27466,0.23438,0.23438,0.26611,0.21729,0.28442,0.3064,0.29053,0.22217,0.24414,0.1709,0.12451,0.13306,0.18677,0.12695,0.17822,0.17822,0.13184,0.068359,0.10376,0.046387,0.048828,0.10132,0.05249,0.11719,0.15381,0.14282,0.10986,0.16968,0.1123,0.083008,0.11719,0.17944,0.14526,0.20996,0.21484,0.18188,0.11597,0.15137,0.087891,0.080566,0.10742,0.16235,0.12817,0.15991,0.13672,0.084229,0.11108,0.048828,0.015869,0.045166,0.089111,0.048828,0.10498,0.11597,0.093994,0.029297,0.087891,0.041504,0.039063,0.092773,0.15015,0.10742,0.14282,0.14404,0.091553,0.12695,0.054932,0.0097656,0.023193,0.076904,0.029297,0.083008,0.11475,0.083008,0.032959,0.085449,0.029297,0.029297,0.090332,0.15015,0.11963,0.17212,0.16602,0.11475,0.17456,0.11597,0.072021,0.090332,0.14038,0.087891,0.15259,0.16357,0.13306,0.068359,0.10986,0.048828,0.032959,0.074463,0.14282,0.10132,0.15625,0.15991,0.10498,0.14404,0.097656,0.062256,0.087891,0.15015,0.10132,0.17334,0.19287,0.17334,0.1123,0.14893,0.095215,0.065918,0.091553,0.15015,0.098877,0.14038,0.14526,0.095215,0.12817,0.062256,0.019531,0.031738,0.083008,0.053711,0.12085,0.14038,0.12939,0.075684,0.11841,0.079346,0.058594,0.085449,0.15991,0.12329,0.16235,0.15869,0.11108,0.15015,0.093994,0.045166,0.05249,0.1001,0.063477,0.14648,0.16846,0.14893,0.096436,0.13306,0.078125,0.061035,0.089111,0.15625,0.10742,0.16602,0.17334,0.12695,0.068359,0.10864,0.057373,0.081787,0.12939,0.087891,0.15259,0.17334,0.15503,0.10254,0.13916,0.076904,0.057373,0.084229,0.14648,0.092773,0.15869,0.15625,0.10986,0.05249,0.090332,0.03418,0.041504,0.091553,0.15015,0.11597,0.15625,0.13794,0.085449,0.1355,0.080566,0.058594,0.085449,0.15259,0.11597,0.17578,0.17944,0.13672,0.070801,0.11353,0.070801,0.065918,0.11108,0.16602,0.14038,0.177,0.16846,0.11841,0.15625,0.097656,0.056152,0.089111,0.15381,0.10498,0.16846,0.18311,0.13794,0.072021,0.11841,0.057373,0.043945,0.10742,0.058594,0.11597,0.15625,0.14648,0.090332,0.14404,0.081787,0.041504,0.058594,0.11597,0.083008,0.14893,0.15747,0.13306,0.058594,0.10254,0.042725,0.030518,0.080566,0.13428,0.10132,0.15625,0.14282,0.096436,0.12817,0.075684,0.037842,0.056152,0.12207,0.070801,0.12451,0.14893,0.12939,0.056152,0.10254,0.05127,0.031738,0.070801,0.13428,0.10376,0.13794,0.13428,0.087891,0.11841,0.065918,0.029297,0.040283,0.093994,0.050049,0.10498,0.13794,0.11963,0.050049,0.093994,0.043945,0.010986,0.054932,0.12207,0.080566,0.12817,0.13184,0.080566,0.11597,0.062256,0.012207,0.031738,0.078125,0.045166,0.11475,0.13306,0.11597,0.057373,0.090332,0.032959,0.019531,0.040283,0.10376,0.074463,0.12451,0.11597,0.080566,0.11597,0.057373,0.018311,0.029297,0.080566,0.040283,0.10864,0.1355,0.11963,0.065918,0.10986,0.048828,0.018311,0.041504,0.10742,0.070801,0.11597,0.11475,0.080566,0.019531,0.05249,0.015869,0.024414,0.073242,0.031738,0.092773,0.11597,0.10742,0.047607,0.090332,0.030518,0.0097656,0.042725,0.10132,0.05127,0.10742,0.11353,0.074463,0.11597,0.059814,0.0012207,0.013428,0.064697,0.01709,0.089111,0.12573,0.10986,0.05249,0.1001,0.039063,0.0061035,0.0354,0.097656,0.05127,0.10498,0.12085,0.087891,0.023193,0.065918,0.0085449,0.013428,0.068359,0.031738,0.10132,0.12939,0.10864,0.056152,0.096436,0.040283,0.0012207,0.026855,0.090332,0.041504,0.098877,0.11475,0.078125,0.0073242,0.056152,0.0036621,-0.0024414,0.065918,0.013428,0.06958,0.11597,0.11108,0.056152,0.097656,0.042725,0.0036621,0.029297,0.079346,0.040283,0.1001,0.11597,0.085449,0.020752,0.070801,0.01709,0.013428,0.057373,0.12085,0.097656,0.13062,0.11841,0.06958,0.10498,0.048828,0.014648,0.029297,0.087891,0.039063,0.093994,0.11597,0.091553,0.023193,0.061035,0.018311,-0.0036621,0.054932,0.10864,0.074463,0.11108,0.11108,0.064697,0.096436,0.032959,0.0024414,0.025635,0.078125,0.032959,0.10376,0.10986,0.090332,0.025635,0.070801,0.015869,0.0073242,0.036621,0.10742,0.06958,0.11108,0.11108,0.065918,0.10254,0.048828,0.013428,0.026855,0.078125,0.043945,0.1001,0.11475,0.091553,0.031738,0.062256,0.012207,-0.0048828,0.05127,0.092773,0.057373,0.10986,0.10986,0.058594,0.0073242,0.043945,-0.0036621,0.031738,0.065918,0.01709,0.081787,0.10986,0.084229,0.03418,0.072021,0.01709,-0.0036621,0.047607,0.096436,0.059814,0.10498,0.10986,0.064697,0.11108,0.057373,0.0097656,0.018311,0.062256,0.025635,0.097656,0.11963,0.096436,0.042725,0.08667,0.019531,-0.0012207,0.042725,0.095215,0.040283,0.1123,0.10742,0.054932,0.014648,0.048828,0,0.012207,0.065918,0.019531,0.084229,0.11597,0.10254,0.040283,0.083008,0.025635,-0.0024414,0.036621,0.089111,0.05127,0.10986,0.10986,0.079346,0.0097656,0.05127,0.0024414,0.0036621,0.054932,0.019531,0.075684,0.12085,0.10742,0.047607,0.090332,0.032959,-0.0073242,0.045166,0.093994,0.05127,0.10498,0.12207,0.080566,0.11597,0.058594,0.0012207,0.0012207,0.053711,0.012207,0.085449,0.10986,0.10132,0.048828,0.090332,0.032959,-0.0012207,0.036621,0.076904,0.037842,0.096436,0.11841,0.087891,0.020752,0.064697,0.014648,0.0061035,0.064697,0.021973,0.080566,0.12817,0.10742,0.05249,0.091553,0.03418,0.0073242,0.023193,0.078125,0.029297,0.093994,0.11108,0.092773,0.026855,0.061035,0.0097656,0.0036621,0.048828,0.10864,0.070801,0.11108,0.098877,0.058594,0.1001,0.036621,0.0061035,0.030518,0.074463,0.042725,0.10376,0.10986,0.090332,0.025635,0.068359,0.020752,0.0024414,0.037842,0.098877,0.075684,0.10986,0.10254,0.054932,0.087891,0.0354,-0.0061035,0.015869,0.053711,0.018311,0.073242,0.10132,0.079346,0.018311,0.058594,0.0024414,-0.0097656,0.054932,0.10376,0.064697,0.1123,0.10132,0.05249,0.10132,0.032959,-0.0061035,0.026855,0.06958,0.031738,0.1001,0.11597,0.1001,0.039063,0.087891,0.030518,0.0073242,0.041504,0.11108,0.063477,0.11963,0.12085,0.068359,0.010986,0.050049,-0.0024414,0.025635,0.068359,0.029297,0.097656,0.11597,0.092773,0.037842,0.080566,0.030518,0.015869,0.040283,0.10742,0.057373,0.11353,0.11841,0.072021,0.10986,0.05249,0.0061035,0.010986,0.057373,0.019531,0.083008,0.11353,0.10132,0.039063,0.078125,0.032959,0.0024414,0.026855,0.10254,0.05127,0.1001,0.11108,0.068359,-0.0012207,0.05127,-0.0061035,0.0085449,0.050049,0.01709,0.087891,0.10132,0.091553,0.041504,0.079346,0.031738,0.0036621,0.026855,0.089111,0.05127,0.096436,0.11108,0.079346,0.01709,0.058594,0.01709,0.010986,0.05249,0.018311,0.084229,0.1123,0.10498,0.05127,0.079346,0.040283,0.013428,0.03418,0.087891,0.041504,0.10132,0.11475,0.087891,0.023193,0.061035,0.0036621,0.010986,0.05249,0.0048828,0.058594,0.10132,0.093994,0.046387,0.089111,0.032959,0.0036621,0.030518,0.087891,0.040283,0.10132,0.11108,0.080566,0.021973,0.056152,0.0024414,-0.0048828,0.043945,0.097656,0.076904,0.10742,0.097656,0.048828,0.091553,0.036621,0.0024414,0.026855,0.080566,0.0354,0.1001,0.11963,0.079346,0.024414,0.070801,0.019531,0.0061035,0.05249,0.10986,0.064697,0.10742,0.097656,0.045166,0.087891,0.031738,-0.0097656,0.032959,0.078125,0.026855,0.095215,0.10742,0.079346,0.029297,0.081787,0.019531,0.0097656,0.061035,0.10864,0.062256,0.11597,0.10376,0.061035,0.10986,0.045166,-0.0012207,0.029297,0.073242,0.0354,0.093994,0.10986,0.087891,0.025635,0.076904,0.030518,0.0073242,0.041504,0.10986,0.068359,0.10864,0.10864,0.056152,0.093994,0.043945,0.0061035,0.023193,0.076904,0.023193,0.075684,0.12085,0.10132,0.032959,0.080566,0.029297,0,0.045166,0.10742,0.054932,0.1001,0.10742,0.05127,0.093994,0.040283,-0.0012207,0.021973,0.068359,0.029297,0.1001,0.10742,0.1062,0.050049,0.1001,0.043945,0.021973,0.041504,0.10132,0.058594,0.10986,0.10498,0.065918,0.0073242,0.05249,0.0097656,0.019531,0.065918,0.029297,0.098877,0.11353,0.097656,0.046387,0.085449,0.05127,0.021973,0.037842,0.081787,0.041504,0.097656,0.1062,0.065918,0.10986,0.042725,-0.0036621,0.019531,0.05249,0.013428,0.076904,0.10864,0.090332,0.043945,0.075684,0.025635,-0.0024414,0.042725,0.087891,0.045166,0.097656,0.095215,0.063477,0.015869,0.048828,-0.0085449,0.010986,0.048828,0.12085,0.087891,0.11597,0.10132,0.05249,0.10254,0.043945,0.0073242,0.025635,0.087891,0.041504,0.098877,0.11353,0.078125,0.0085449,0.059814,0.0097656,0.013428,0.061035,0.10742,0.075684,0.11475,0.10132,0.042725,0.089111,0.030518,0.0012207,0.031738,0.087891,0.024414,0.095215,0.11108,0.079346,0.010986,0.062256,0.0048828,-0.0036621,0.065918,0.10986,0.064697,0.10864,0.10132,0.058594,0.11108,0.05249,0.012207,0.032959,0.090332,0.05127,0.10498,0.11719,0.084229,0.025635,0.075684,0.020752,0.0036621,0.041504,0.11108,0.075684,0.11353,0.11108,0.058594,0.097656,0.057373,0.01709,0.031738,0.085449,0.036621,0.087891,0.12085,0.090332,0.024414,0.065918,0.019531,0.0097656,0.050049,0.0073242,0.065918,0.11353,0.11353,0.068359,0.10132,0.031738,-0.015869,0.0097656,0.068359,0.037842,0.12451,0.14648,0.11841,0.048828,0.054932,-0.0024414,-0.0085449,0.023193,0.097656,0.091553,0.14282,0.13916,0.090332,0.10742,0.026855,-0.0097656,0.012207,0.061035,0.057373,0.11963,0.12695,0.11597,0.042725,0.05127,0.0097656,-0.015869,0.020752,0.097656,0.068359,0.12451,0.13672,0.090332,0.0036621,0.024414,-0.025635,0.0012207,0.061035,0.030518,0.10376,0.14404,0.11475,0.042725,0.058594,-0.0036621,-0.025635,0.026855,0.098877,0.074463,0.13306,0.14404,0.080566,0.10742,0.023193,-0.019531,-0.0036621,0.053711,0.023193,0.11597,0.14038,0.12695,0.057373,0.072021,0.0048828,-0.021973,0.019531,0.080566,0.062256,0.12939,0.13672,0.084229,0.10986,0.031738,-0.025635,-0.0085449,0.05249,0.018311,0.10376,0.13428,0.10742,0.048828,0.067139,0,-0.031738,0.0061035,0.073242,0.05127,0.11841,0.12695,0.089111,0.013428,0.03418,-0.023193,-0.0048828,0.05249,0.023193,0.1001,0.14526,0.12451,0.064697,0.087891,0.0097656,-0.020752,0.020752,0.079346,0.067139,0.13062,0.1416,0.10132,0.021973,0.042725,-0.0061035,-0.0036621,0.056152,0.023193,0.10254,0.13916,0.13062,0.058594,0.076904,0.018311,-0.025635,0.0036621,0.06958,0.05127,0.10986,0.13306,0.10376,0.018311,0.036621,-0.025635,-0.028076,0.042725,0.1062,0.087891,0.13062,0.11353,0.054932,0.070801,0.0097656,-0.029297,0.0024414,0.068359,0.048828,0.11475,0.13062,0.10498,0.014648,0.039063,-0.014648,-0.019531,0.031738,0.10132,0.081787,0.12939,0.12451,0.072021,0.097656,0.021973,-0.015869,0.020752,0.06958,0.043945,0.10986,0.13062,0.10376,0.021973,0.040283,-0.01709,-0.0354,0.023193,0.1001,0.083008,0.13306,0.12329,0.072021,0.091553,0.018311,-0.019531,0,0.05249,0.032959,0.097656,0.12817,0.097656,0.024414,0.042725,-0.015869,-0.0354,0.019531,0.090332,0.070801,0.12817,0.12573,0.061035,0.083008,0.013428,-0.019531,-0.0061035,0.065918,0.025635,0.10986,0.12695,0.10376,0.03418,0.05127,-0.0073242,-0.01709,0.013428,0.081787,0.058594,0.11597,0.12695,0.079346,0.10132,0.020752,-0.026855,-0.0085449,0.057373,0.041504,0.11353,0.14038,0.11963,0.040283,0.059814,0.0073242,-0.019531,0.012207,0.087891,0.065918,0.12085,0.12451,0.074463,0.097656,0.015869,-0.036621,-0.018311,0.036621,0.0097656,0.087891,0.12939,0.10254,0.032959,0.054932,-0.0061035,-0.029297,0.025635,0.089111,0.061035,0.11963,0.12939,0.083008,0.0024414,0.018311,-0.029297,-0.024414,0.032959,0.0085449,0.090332,0.11475,0.10498,0.046387,0.062256,0.0024414,-0.040283,0.0048828,0.080566,0.059814,0.11841,0.13916,0.08667,0.018311,0.0354,-0.015869,0,0.05127,0.026855,0.10498,0.14038,0.12207,0.065918,0.087891,0.0073242,-0.029297,-0.0012207,0.072021,0.048828,0.12207,0.14038,0.10742,0.01709,0.036621,-0.023193,-0.012207,0.045166,0.013428,0.097656,0.1416,0.11963,0.064697,0.074463,0.012207,-0.021973,0.019531,0.079346,0.057373,0.11108,0.12207,0.084229,0.0036621,0.026855,-0.019531,-0.029297,0.032959,0.10132,0.083008,0.12817,0.11841,0.067139,0.091553,0.015869,-0.025635,0.0024414,0.057373,0.042725,0.10864,0.12695,0.097656,0.014648,0.0354,-0.0097656,-0.015869,0.039063,0.10742,0.079346,0.12817,0.11963,0.059814,0.078125,0.0024414,-0.025635,0.0024414,0.057373,0.032959,0.11108,0.13672,0.11108,0.0354,0.054932,-0.0024414,-0.015869,0.029297,0.1001,0.078125,0.13916,0.13306,0.074463,0.091553,0.0073242,-0.040283,-0.0061035,0.068359,0.041504,0.10742,0.12695,0.090332,0.021973,0.043945,-0.012207,-0.021973,0.018311,0.1001,0.080566,0.13062,0.13062,0.073242,0.095215,0.024414,-0.015869,0.0012207,0.05249,0.023193,0.087891,0.12573,0.097656,0.029297,0.054932,-0.0048828,-0.026855,0.018311,0.083008,0.062256,0.13062,0.12939,0.073242,0.10986,0.01709,-0.029297,0,0.045166,0.032959,0.11597,0.13672,0.12085,0.053711,0.070801,0,-0.023193,0.013428,0.085449,0.064697,0.12695,0.13428,0.076904,0.10132,0.015869,-0.031738,-0.012207,0.05127,0.026855,0.10132,0.13306,0.10742,0.036621,0.061035,-0.0012207,-0.020752,0.021973,0.087891,0.062256,0.12451,0.12939,0.085449,0.0097656,0.030518,-0.026855,-0.0097656,0.042725,0.0061035,0.075684,0.11963,0.10742,0.050049,0.068359,0.0073242,-0.028076,0.013428,0.081787,0.061035,0.11841,0.13062,0.093994,0.0097656,0.036621,-0.015869,-0.0085449,0.05127,0.01709,0.10376,0.1416,0.13184,0.068359,0.084229,0.0097656,-0.025635,0.0073242,0.076904,0.054932,0.11597,0.13672,0.093994,0.0085449,0.025635,-0.026855,-0.024414,0.05127,0.015869,0.090332,0.12939,0.10986,0.050049,0.076904,0.0097656,-0.021973,0.0024414,0.065918,0.048828,0.11597,0.13062,0.097656,0.019531,0.040283,-0.013428,-0.018311,0.041504,0.1123,0.083008,0.13428,0.11841,0.058594,0.081787,0.013428,-0.028076,0.019531,0.072021,0.050049,0.11597,0.13672,0.10254,0.032959,0.05127,-0.0097656,-0.019531,0.029297,0.1001,0.081787,0.13672,0.13306,0.076904,0.093994,0.019531,-0.020752,0.0024414,0.064697,0.050049,0.10742,0.13184,0.10986,0.031738,0.054932,0.0048828,-0.030518,0.029297,0.1001,0.074463,0.12329,0.12573,0.059814,0.083008,0.0036621,-0.040283,-0.0097656,0.063477,0.025635,0.10986,0.12817,0.10254,0.032959,0.042725,-0.010986,-0.021973,0.020752,0.093994,0.06958,0.12329,0.12939,0.075684,0.096436,0.023193,-0.021973,-0.0012207,0.062256,0.031738,0.10986,0.13306,0.1123,0.0354,0.05127,-0.0085449,-0.029297,0.01709,0.097656,0.063477,0.13062,0.13794,0.085449,0.012207,0.037842,-0.020752,-0.0048828,0.059814,0.026855,0.10132,0.1355,0.11475,0.046387
