# Supplementary material for: Removal of Dust Microelectric Signal Based on Empirical Mode Decomposition and Multifractal Detrended Fluctuation Analysis
Source: Comput Intell Neurosci. 2021 Aug 6;2021:5468514. doi: 10.1155/2021/5468514 (PMC8367589; doi:10.1155/2021/5468514)
Supplement: Supplementary Materials — “Measured_noise_data.docx” includes the measured noise used in this paper. “Measured_signal_data.docx” includes the measured signal used in this paper. [file 5468514.f1.zip › 5468514.f1/Suplementary description.docx]

**Supplementary Material:**

“measured_noise_data.docx” includes the measured noise used in this paper.

“measured_signal_data.docx” includes the measured signal used in this paper.
